# Mako: A Graph-based Pattern Growth Approach to Detect Complex Structural Variants

**DOI:** 10.1016/j.gpb.2021.03.007

**Published:** 2021-07-03

**Authors:** Jiadong Lin, Xiaofei Yang, Walter Kosters, Tun Xu, Yanyan Jia, Songbo Wang, Qihui Zhu, Mallory Ryan, Li Guo, Chengsheng Zhang, The Human Genome Structural Variation Consortium, Mark B. Gerstein, Ashley D. Sanders, Micheal C. Zody, Michael E. Talkowski, Ryan E. Mills, Jan O. Korbel, Tobias Marschall, Peter Ebert, Peter A. Audano, Bernardo Rodriguez-Martin, David Porubsky, Marc Jan Bonder, Arvis Sulovari, Jana Ebler, Weichen Zhou, Rebecca Serra Mari, Feyza Yilmaz, Xuefang Zhao, PingHsun Hsieh, Joyce Lee, Sushant Kumar, Tobias Rausch, Yu Chen, Zechen Chong, Katherine M. Munson, Mark J.P. Chaisson, Junjie Chen, Xinghua Shi, Aaron M. Wenger, William T. Harvey, Patrick Hansenfeld, Allison Regier, Ira M. Hall, Paul Flicek, Alex R. Hastie, Susan Fairely, Charles Lee, Scott E. Devine, Evan E. Eichler, Kai Ye

**Affiliations:** 1 Program in Computational Biology and Bioinformatics, Yale University, New Haven, CT 06520, USA; 2 European Molecular Biology Laboratory (EMBL), Genome Biology Unit, D-69117 Heidelberg, Germany; 3 New York Genome Center, New York, NY 10013, USA; 4 Center for Genomic Medicine, Massachusetts General Hospital, Department of Neurology, Harvard Medical School, Boston, MA 02114, USA; 5 Department of Computational Medicine & Bioinformatics, University of Michigan, Ann Arbor, MI 48109, USA; 6 Heinrich Heine University, Medical Faculty, Institute for Medical Biometry and Bioinformatics, D-40225 Düsseldorf, Germany; 7 Department of Genome Sciences, University of Washington School of Medicine, Seattle, WA 98195-5065, USA; 8 Division of Computational Genomics and Systems Genetics, German Cancer Research Center (DKFZ), D-69120 Heidelberg, Germany; 9 The Jackson Laboratory for Genomic Medicine, Farmington, CT 06030, USA; 10 Bionano Genomics, San Diego, CA 92121, USA; 11 Department of Genetics and Informatics Institute, School of Medicine, University of Alabama at Birmingham, Birmingham, AL 35294, USA; 12 Molecular and Computational Biology, University of Southern California, Los Angeles, CA 90089, USA; 13 Department of Computer & Information Sciences, Temple University, Philadelphia, PA 19122, USA; 14 Pacific Biosystems of California, Inc, Menlo Park, CA 94025, USA; 15 Washington University, St. Louis, MO 63108, USA; 16 European Molecular Biology Laboratory, European Bioinformatics Institute, Wellcome Genome Campus, Hinxton, Cambridge, CB10 1SD, United Kingdom; 1 School of Automation Science and Engineering, Faculty of Electronic and Information Engineering, Xi’an Jiaotong University, Xi’an 710049, China; 2 MOE Key Lab for Intelligent Networks & Networks Security, Faculty of Electronic and Information Engineering, Xi’an Jiaotong University, Xi’an 710049, China; 3 Genome Institute, the First Affiliated Hospital of Xi’an Jiaotong University, Xi’an 710061, China; 4 Leiden Institute of Advanced Computer Science, Faculty of Science, Leiden University, Leiden 2311EZ, Netherland; 5 School of Computer Science and Technology, Faculty of Electronic and Information Engineering, Xi’an Jiaotong University, Xi’an 710049, China; 6 The Jackson Laboratory for Genomic Medicine, Farmington, CT 06032, USA; 7 Precision Medicine Center, the First Affiliated Hospital of Xi’an Jiaotong University, Xi’an 710061, China; 8 Institute for Genome Sciences, University of Maryland School of Medicine, Baltimore, MD 21201, USA; 9 Department of Genome Sciences, University of Washington School of Medicine, Seattle, WA 98119, USA; 10 Howard Hughes Medical Institute, University of Washington, Seattle, WA 98195, USA; 11 The School of Life Science and Technology, Xi’an Jiaotong University, Xi’an 710049, China

**Keywords:** Next-generation sequencing, Complex structural variant, Pattern growth, Graph mining, Formation mechanism

## Abstract

**Complex structural variants** (CSVs) are genomic alterations that have more than two breakpoints and are considered as the simultaneous occurrence of simple structural variants. However, detecting the compounded mutational signals of CSVs is challenging through a commonly used model-match strategy. As a result, there has been limited progress for CSV discovery compared with simple structural variants. Here, we systematically analyzed the multi-breakpoint connection feature of CSVs, and proposed Mako, utilizing a bottom-up guided model-free strategy, to detect CSVs from paired-end short-read sequencing. Specifically, we implemented a graph-based **pattern growth** approach, where the graph depicts potential breakpoint connections, and pattern growth enables CSV detection without pre-defined models. Comprehensive evaluations on both simulated and real datasets revealed that Mako outperformed other algorithms. Notably, validation rates of CSVs on real data based on experimental and computational validations as well as manual inspections are around 70%, where the medians of experimental and computational breakpoint shift are 13 bp and 26 bp, respectively. Moreover, the Mako CSV subgraph effectively characterized the breakpoint connections of a CSV event and uncovered a total of 15 CSV types, including two novel types of adjacent segment swap and tandem dispersed duplication. Further analysis of these CSVs also revealed the impact of sequence homology on the formation of CSVs. Mako is publicly available at https://github.com/xjtu-omics/Mako.

## Introduction

**Figure 1:**
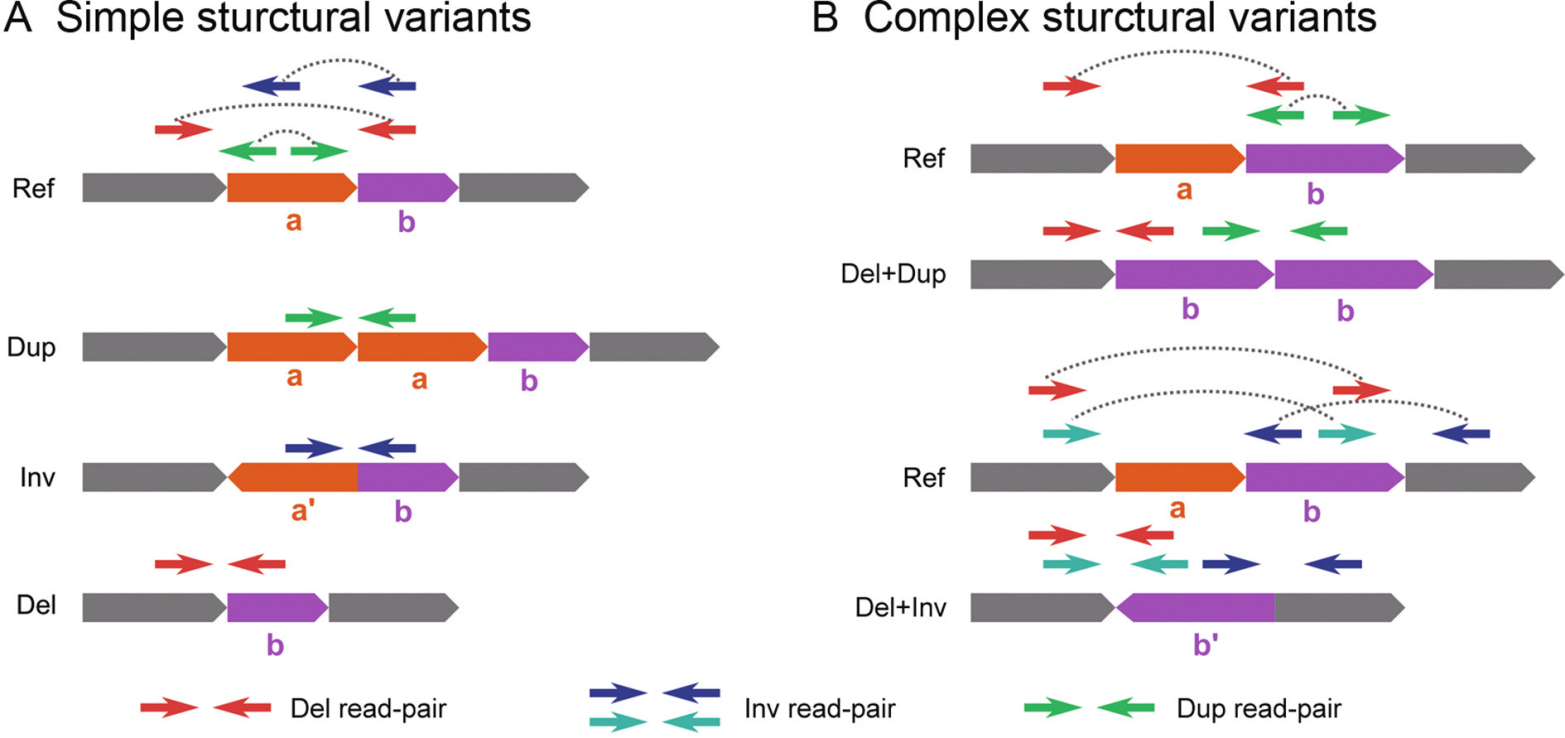
**Explanation of simple and complexSValignment models derived from abnormal read-pairs** **A.** Three common simple SVs and their corresponding abnormal read-pair alignments on the reference genome. **B.** The alignment signature of two CSVs. Each involves two types of signatures that can be matched by a simple SV alignment model. SV, structural variant; CSV, complex structural variant; Ref, reference; Dup, duplication; Inv, inversion; Del, deletion.

Computational methods based on next-generation sequencing (NGS) have provided an increasingly comprehensive discovery and catalog of simple structure variants (SVs) that usually have two breakpoints, such as deletions (Dels) and inversions (Invs) [1], [2], [3], [4], [5], [6], [7]. In general, these approaches follow a model-match strategy, where a specific SV model and its corresponding mutational signal model are proposed. Afterward, the mutational signal model is used to match observed signals for the detection (Figure 1A). This model-match strategy has been proved effective for detecting simple SVs, providing us with prominent opportunities to study and understand genome evaluation and disease progression [8], [9], [10], [11]. However, recent research has revealed that some rearrangements have multiple, compounded mutational signals and usually cannot fit into the simple SV models [8], [12], [13], [14], [15], [16] (Figure 1B). For example, in 2015, Sudmant et al. [8] systematically categorized 5 types of complex structural variants (CSVs) and found that a remarkable 80% of 229 Inv sites were complex events. Collins et al. [17] used long-insert size whole-genome sequencing (liWGS) on autism spectrum disease (ASD) and successfully resolved 16 classes of 9666 CSVs from 686 patients. In 2019, Lee et al. [16] revealed that 74% of known fusion oncogenes of lung adenocarcinomas were caused by complex genomic rearrangements, including *EML4-ALK* and *CD74-ROS1*. Though less frequently reported, compared with simple SVs, these multiple breakpoint rearrangements were considered as punctuated events, leading to severe genome alterations at once [10], [18], [19], [20], [21]. This dramatic change of genome provided distinctive evidence to study formation mechanisms of rearrangement and to understand cancer genome evolution [13], [14], [17], [18], [19], [21], [22], [23], [24].

However, due to the lack of effective CSV detection algorithms, most CSV-related studies screen these events from the “sea” of simple SVs through computational expensive contig assembly and realignment, clustering of incomplete breakpoints, or even targeted manual inspection [8], [12], [16]. In fact, many CSVs have already been neglected or misclassified in this “sea” because of the incompatibility between complicated mutational signals and existing SV models. Although the importance and challenge for CSV detection have been recognized, only a few dedicated algorithms have been proposed for CSV discovery, and they follow two major approaches guided by the model-match strategy. TARDIS and SVelter utilize the top-down approach, where they attempt to model all the mutational signals of a CSV event instead of modeling specific parts of signals. In particular, TARDIS [25] proposes sophisticated abnormal alignment models to depict the mutational signals reflected by dispersed duplication (Disdup) and inverted duplication (Invdup). The pre-defined models are then used to fit observed signals from alignments for the detection of the two specific CSV types. Indeed, this is complicated and greatly limited by the diverse types of CSVs. To solve this, SVelter [26] replaces the modeling process for specific CSVs with a randomly created virtual rearrangement. And CSVs are detected by minimizing the difference between the virtual rearrangement and the observed signals. However, GRIDSS [27] represents the assembly-based approach, which detects CSVs through extra breakpoints discovered from contig-assembly and realignment. Although the assembly-based approach is sensitive for breakpoint detection, it lacks certain regulations to constrain or classify these breakpoints and leaves them as independent events. As a result, these model-match-guided approaches would substantially break up or misinterpret the CSVs because of partially matched signals (Figure 1B). Moreover, the graph is another approach that has been widely used for simple [2], [28] and complex [19], [29] SV detection. Notably, ARC-SV [29] uses clustered discordant read-pairs to construct an adjacency graph and adopts a maximum likelihood model to detect CSVs, showing the great potential of using the graph to detect CSVs. Accordingly, there is an urgent demand for a new strategy, enabling CSV detection without pre-defined models as well as maintaining the completeness of a CSV event.

In this study, we proposed a bottom-up guided model-free strategy, implemented as Mako, to effectively discover CSVs all at once based on short-read sequencing. Specifically, Mako uses a graph to build connections of mutational signals derived from abnormal alignment, providing the potential breakpoint connections of CSVs. Meanwhile, Mako replaces model fitting with the detection of maximal subgraphs through a pattern growth approach. Pattern growth is a bottom-up approach, which captures the natural features of data without sophisticated model generation, allowing CSV detection without pre-defined models. We benchmarked Mako against five widely used tools on a series of simulated and real data. The results show that Mako is an effective and efficient algorithm for CSV discovery, which will provide more opportunities to study genome evolution and disease progression from large cohorts. Remarkably, the analysis of subgraphs detected by Mako highlights the unique strength of Mako, where Mako is able to effectively characterize the CSV breakpoint connections, confirming the completeness of a CSV event. Moreover, we systematically analyzed the CSVs detected by Mako on three healthy samples, revealing a novel role of sequence homology in CSV formation.

## Method

### Overview of Mako

**Figure 2:**
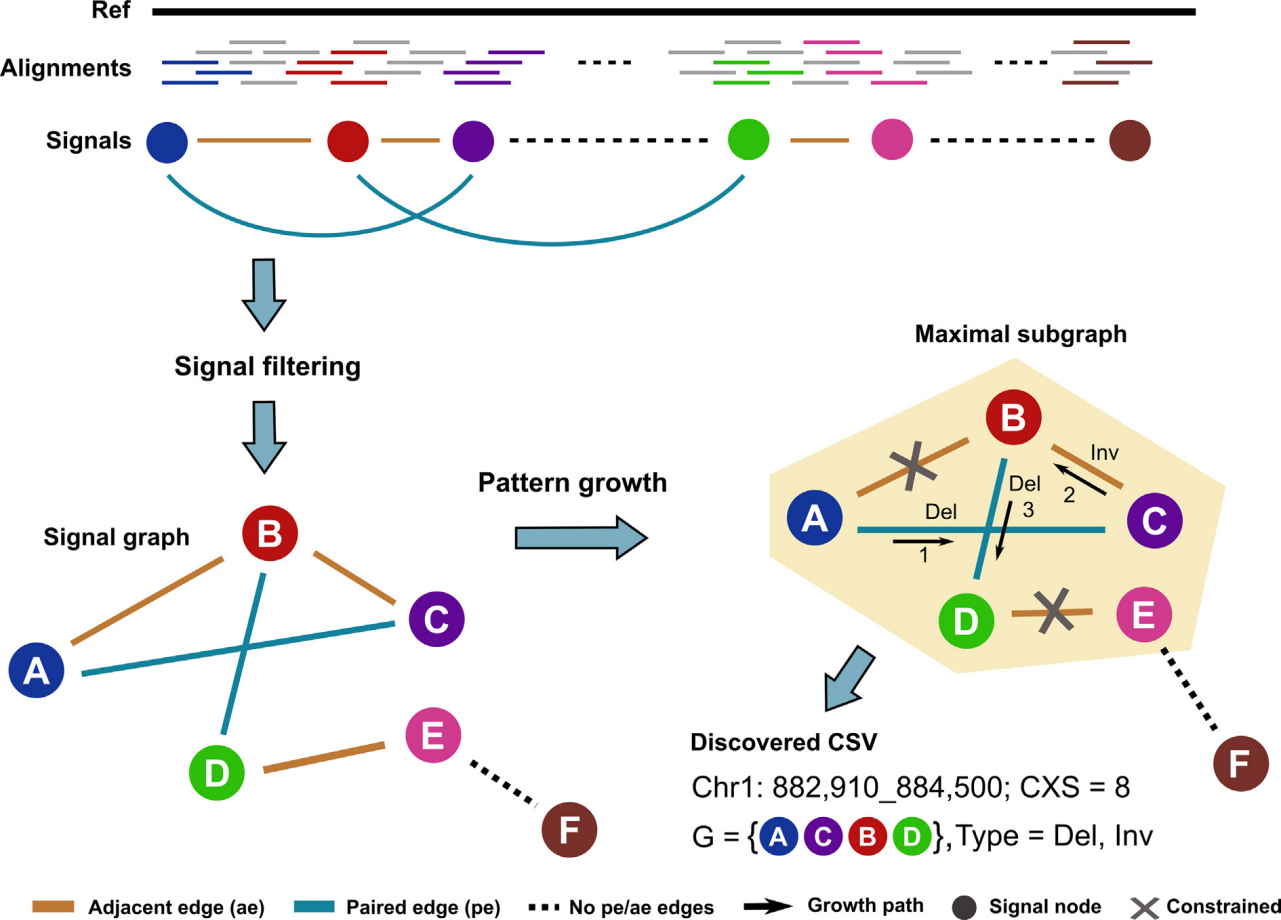
**Overview of Mako** Mako first builds a signal graph by collecting abnormally aligned reads as nodes, and their edge connections are provided by paired-end alignment and split alignment. Afterward, Mako utilizes the pattern growth approach to find a maximal subgraph as a potential CSV site. In the example output, the maximal subgraph G contains nodes A, B, C, and D, whereas F is not able to be appended because of no existing edge (dashed line). The CSV is derived from this subgraph with estimated breakpoints and CXS, where the discovered CSV subgraph contains four different nodes, one $E_{\mathrm{ae}}$ edge of type Inv, and two $E_{\mathrm{pe}}$ edges of type Del. CXS, complexity score.

Given that a CSV is a single event with multiple breakpoint connections, the breakpoints in the current CSV shall not connect with false-positive breakpoints or those from unrelated events. Thus, we formulate the discovery of CSVs as maximal subgraph pattern detection in a signal graph. Accordingly, Mako detects CSVs with NGS data in two major steps, *i.e.*, signal graph creation and subgraph detection (Figure 2). Firstly, Mako collects and clusters abnormally aligned reads as signal nodes and defines two types of edges to build the signal graph G=(V,E), with $V=\{v_{1},v_{2},...,v_{n}\}$ and $E=\{E_{\mathrm{pe}},E_{\mathrm{ae}}\}$. Each signal node v∈V is represented as v=(type,pos,weight), where *type*, *pos*, and *weight* denote the abnormal alignment type, node position, and the number of supporting abnormal reads, respectively. For the edge set, each edge in $E_{\mathrm{pe}}$ and $E_{\mathrm{ae}}$ is represented as $e_{\mathrm{pe}}=\left(v_{i},v_{j},rp\right)$ and $e_{\mathrm{ae}}=\left(v_{i},v_{j},dist\right)$, respectively, where $v_{i},v_{j}\in V$. Specifically, $E_{\mathrm{pe}}$ represents paired edges from a certain number of supporting paired-reads or split-reads (*sr*). $E_{\mathrm{ae}}$ indicates the adjacent edges induced from the reference genome, connecting two adjacent signal nodes of distance (*dist*). Secondly, Mako applies a pattern growth approach to detect the maximal subgraphs as potential CSVs at the whole-genome scale. Meanwhile, the attributes of the subgraph are used to measure the complexity, and CSV types are determined by the edge connection types of the corresponding subgraphs (Figure 2).

### Building signal graph

To create the signal graph, Mako collects abnormally aligned reads that satisfy one of the following criteria from the alignment file: 1) clipped portion with minimum 10% size fraction of the overall read length; 2) split reads with high mapping quality; 3) discordant read-pairs. As a result, one group of signal nodes is created by clustering clipped-reads or split-reads at the same position on the genome, which is filtered by *weight* and the ratio between *weight* and the coverage at *pos*. Another group of signal nodes is derived from clusters of discordant read-pairs, where the clustering distance is the estimated average insert size minus two-fold read length. It should be noted that a discordant alignment produces two nodes, and Mako separately clusters discordant alignments with multiple abnormally aligned types, such as abnormal insert size and incorrect mapping orientation. We adopt the procedure introduced by Chen [4] to avoid using randomly occurred discordant alignment (File S1). Additionally, edges are created alone with the signal nodes, where multiple types of edges might co-exist between two nodes.

### Detecting CSVs with pattern growth

Pattern growth has been widely used in many areas [30], [31], [32], [33], [34], [35], such as insertion/deletion (Indel) detection in DNA sequences [1], [23]. For CSV detection, the subgraph pattern starts at a single node and grows by adding one node each time until it cannot find a proper one (Algorithm I). Specifically, the subgraph is allowed to grow according to the increasing order of *pos* value for each node, and backtracking is only allowed for nodes involved in the current subgraph. Of note, pattern growth via adjacent edges is conditional to the distance constrain (*minDist*) because these edges are derived from the reference genome instead of alternatives. For example, Mako detects the maximal subgraph ACBD by visiting nodes A, C, B, and D, while the edge between D and E is constrained because of the larger distance (Figure 2).

Given that the signal graph contains millions of nodes at the whole-genome scale, we adopt the “seed-and-extension” [36], [37] strategy to accelerate subgraph detection. Moreover, the discovered subgraphs not only differ in edge connections but also in node *type* of the subgraph. Therefore, we propose an algorithm that starts at multiple signal nodes of the same *type* at the whole-genome scale, while extends locally for subgraph detection (Algorithm II). The parameter *minFreq* is used to measure the frequency of detected subgraphs, and Mako uses *minFreq =* 1 to avoid missing subgraphs of rare CSVs or incomplete ones. The detected CSV subgraph provides the connections between multiple breakpoints of a CSV, and the attributes of the subgraph are used to measure the complexity of CSVs. Accordingly, Mako defines the boundary of CSVs using the leftmost and rightmost *pos* values of the nodes and utilizes the number of identical node types multiplied by the number of $E_{\mathrm{pe}}$ edges as a complexity measurement score (CXS). For example, the discovered CSV subgraph ACBD has a CXS of 8 due to 4 different node types, *i.e.*, A, C, B, and D, and two paired edges (Figure 2). A toy example of excuting the algorithm is shown in Figure S1.

**Table:** 

**Algorithm I: Detect maximal subgraphs**
**Input:** Signal graph G=(V,E), **parameters**minFreq,minDist
**Output:** A set of CSV subgraphs $O=\{g_{1},g_{2},\cdots ,g_{n}\}$, with $freq\left(g_{i}\right)\geq minFreq$
1: **procedure**findMaximalSubgraph(G,minFreq,minDist)
2: Initialize freq_types equals to type frequency of node in V;
3: Build index-projection ${G|}_{\emptyset }$ of G;
4: **for**α**in**freq_types**do**:
5: Build index-projection ${G|}_{\alpha }$;
6: $g_{i}=\alpha$;
7: **if**$freq\left(g_{i}\right)>minFreq$**then**
8: $\mathrm{multiLocPatternGrowth}(O,g_{i},{G|}_{\alpha },minFreq,minDist)$;
9: **end if**
10: **end for**
11: **end procedure**

**Table:** 

**Algorithm II: Multi-location subgraph growth**
1: **procedure**$\mathrm{multiLocPatternGrowth}(O,g,{G|}_{g},minFreq,minDist)$
2: Initialize adj_list with adjacent node direct after g through E;
3: **for**node**in**adj_list**do**:
4: **if**nodeInRange(g,node)**then**
5: $g^{\text{'}}=g+node$;
6: $O.append(g^{\text{'}})$;
7: $\mathrm{multiLocPatternGrowth}(O,g\text{'},{G|}_{g\text{'}},minFreq,minDist)$;
8: **end if**
9: **end for**
10: **end procedure**
11: **procedure**nodeInRange(g,v)
12: Set the nodes in g with respect increasing order of pos value: $v_{0},v_{1},\cdots v_{n}$;
13: Set $v^{\text{'}}=v_{n}$;
14: **if**freq(v)>minFreq**then**
15: **if**$\mathrm{dist}(v^{\prime },v)<minDist$**then**
16: **return** True
17: **else**:
18: **for**i=n**to** 0 **do**
19: **if**$\exists e_{\mathrm{pe}}$ between v and $v_{i}$**then**
20: **return** True
21: **end if**
22: **end if**
23: **return** False
24: **end procedure**

### Performance evaluation

Since CSVs contain multiple breakpoints, we propose two tiers of stringency for their evaluation, *i.e.*, unique-interval match and all-breakpoint match. For a unique-interval match, the correct predicted breakpoints shall be within 500-bp distance to the leftmost and rightmost breakpoints of a benchmark CSV. For the all-breakpoint match initially proposed by Sniffles [38], a benchmark CSV is divided into separate subcomponents, and each of them should be correctly detected. For a CSV with an Inv flanked by two Dels containing three components, the correct prediction of all breakpoints for the three components is considered as an all-breakpoint match. Meanwhile, if only one prediction is close to the leftmost and rightmost breakpoints of the CSV, this prediction is considered as a unique-interval match. For simulated CSVs, true positives (TPs) are defined as predictions satisfying either match criterion, while predictions not in the benchmark are false positives (FPs). False negatives (FNs) are events in the benchmark set that are not matched by predictions. Whereas it is usually challenging to measure the FPs for real data due to the lack of a curated CSV set, we only consider the number of correct discoveries (File S1).

### Preparing CSV benchmarks for performance evaluation

In this study, we use both simulated and real CSVs to benchmark the performance of different callers. We follow the workflow introduced by the Sniffles [38] to create simulated CSVs (Figure S2). Firstly, VISOR [39] is used to create Del, Inv, Invdup, tandem duplication (Tandup), and Disdup. These events, termed as basic operations, are implanted and marked on the reference genome GRCh38 to generate an alternative genome. Secondly, CSVs are created by randomly adding basic operations to those marked operations, leading to a new genome harboring CSVs (CSV genome). Meanwhile, the purity parameter of VISOR is used to produce homozygous and heterozygous CSVs. Afterward, VISOR generates simulated paired-end reads based on the CSV genome with wgsim (https://github.com/lh3/wgsim) and aligns them to the reference genome with BWA-MEM [37]. According to the above-generalized simulation procedures, we create reported CSV types published by previous studies [8], [17] and randomized CSV types (File S1).

In terms of the real data, we are not aware of any public CSV benchmarks due to the breakpoint complexity and underdeveloped methods [8], [12], [26], [40], [41]. Fortunately, Pacific Biosciences (PacBio) reads could span multiple breakpoints of CSVs, providing direct evidence to validate CSVs through sequence Dotplot [42]. Thus, we curate the CSV benchmark from a simple SV callset by breakpoint clustering and manual inspection. For SV clustering, each of them is considered as an interval, and hierarchical clustering with the average method is used to find interval clusters (Figures S3 and S4). We then use the threshold that could produce the most clusters for merging clusters, which could potentially reduce the number of missed CSVs (Figures S5 and S6; Table S1). Given these simple SV clusters, we apply Gepard to create Dotplots based on PacBio high-fidelity (HiFi) reads and manually investigate each Dotplot. Since CSVs are rare and might appear at the minor allele, we create Dotplot for each long read that spans the corresponding region.

### Orthogonal validation of Mako-detected CSVs

To fully characterize Mako’s performance on real data, we use experimental and computational validations as well as manual inspections of CSVs from HG00733. The raw CSV calls from HG00733 are obtained by selecting events with more than one link type observed in the subgraph. For the experimental validation, Primer3 (https://github.com/primer3-org/primer3) is used to design PCR primers, where primers are selected within the extended distance but 200 bp outside of the boundaries of the breakpoints defined by Mako (Figure S7). BLAT (https://users.soe.ucsc.edu/~kent/) search is performed at the same time to ensure all primer candidates have only one hit in the human genome. Afterward, we select amplification products with the expected product size and bright electrophoretic bands for Sanger sequencing (Figure S8). The obtained Sanger sequences are aligned against the reference allele of the CSV site and visualized with Gepard for breakpoint inspection (File S1).

As for the computational validation, two orthogonal data obtained from Human Genome Structural Variant Consortium (HGSVC) are used, *i.e.*, Oxford Nanopore Technologies (ONT) sequencing and HiFi contigs. We first apply VaPoR [43] on the ONT reads to validate CSVs, referring as ONT validation. Additionally, we apply a *K*-mer-based breakpoint examination based on haplotype-aware HiFi contigs, from which we calculate the difference between the *K*-mer breakpoints and predicted breakpoints (Figure S9; File S1).

Furthermore, we manually curate detected CSVs via Dotplots created by Gepard (Figure S10), which is similar to the procedure of creating the benchmark CSVs for real data (File S1). For CSVs at highly repetitive regions, we further validate them according to specific patterns (Figures S11–S13).

## Results

### Mako effectively characterizes multiple breakpoints of CSVs

**Figure 3:**
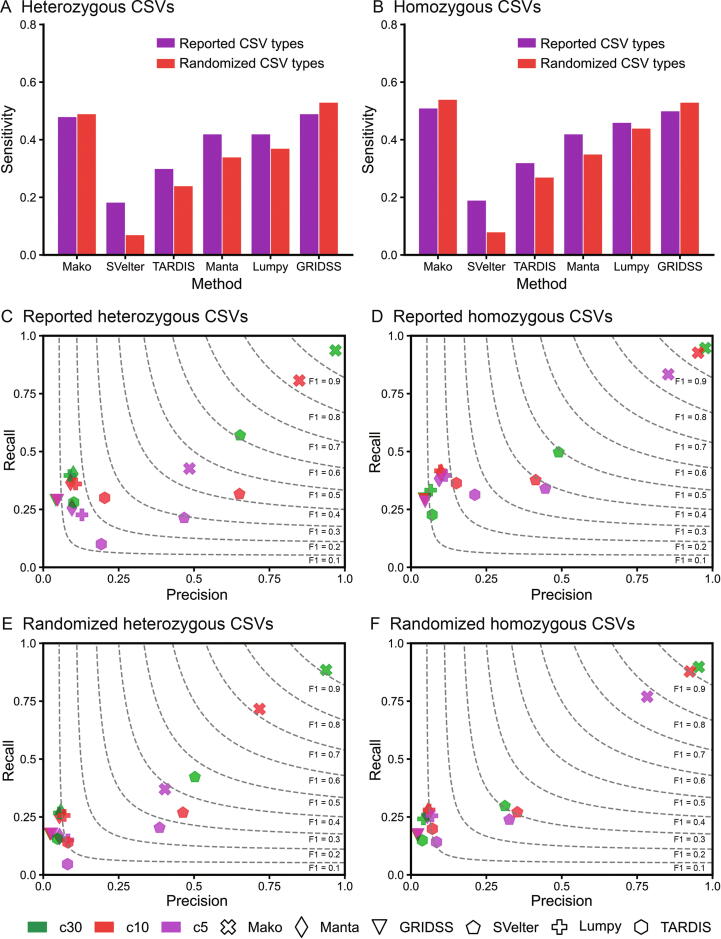
**Performance comparison on simulated CSVs with different match criteria** **A.** The sensitivity of detecting breakpoints of heterozygous CSVs. **B.** The sensitivity of detecting breakpoints of homozygous CSVs. **C.** Evaluation of reported heterozygous CSV simulation. **D.** Evaluation of reported homozygous CSV simulation. **E.** Evaluation of randomized heterozygous CSV simulation. **F.** Evaluation of randomized homozygous CSV simulation. The performances of selected tools for detecting simulated CSVs are evaluated according to the all-breakpoint match (A and B) and unique-interval match (C–F) criteria. In C–F, the performance is evaluated by recall (y-axis), precision (x-axis), and F1-score (dotted lines). The right top corner of the plot indicates better performance. The c5–c30 indicates coverage, *e.g.*, c5 indicates 5× coverage.

The most important feature for a CSV is the presence of multiple breakpoints in a single event. Thus, we first examined the performances of Mako, Lumpy, Manta, SVelter, TARDIS, and GRIDSS for detecting multiple breakpoints. The results were evaluated according to the all-breakpoint match criterion on both reported and randomized CSV-type simulations. Overall, for the heterozygous (Figure 3A) and homozygous (Figure 3B) simulations, Mako was comparable to GRIDSS, and these two methods outperformed other algorithms. For example, GRIDSS, Mako, and Lumpy detected 50%, 51%, and 46% of reported heterozygous CSV breakpoints, while they reported 53%, 54%, and 44% of randomized ones. Because the graph encoded both multiple breakpoints and their substantial connections for each CSV, Mako achieved better performance on randomized events, which included more subcomponents than the reported ones. Indeed, by comparing reported and randomized simulations, the breakpoint detection sensitivity (Figure 3A and B) of Mako for randomized simulation increased, while that of other algorithms dropped except for GRIDSS. Although the assembly-based method, GRIDSS, is as effective as Mako for breakpoint detection, it lacks a proper procedure to resolve the connections among breakpoints.

### Mako precisely discovers CSV unique-interval

CSV is considered as a single event consisting of connected breakpoints, and we have demonstrated that Mako is able to detect CSV breakpoints effectively. However, the breakpoint detection evaluation only assesses the discovery of basic components for a CSV and lacks examination for CSV completeness. We then investigated whether Mako could precisely capture the entire CSV interval even with missing breakpoints. According to the unique-interval match criterion, Mako consistently outperformed other algorithms for both reported and randomly created CSVs, while SVelter and GRIDSS ranked second and third, respectively. For the reported CSVs at 30× coverage (Figure 3C and D), the recalls of Mako were 92% and 94% for reported heterozygous and homozygous CSVs, respectively, which were significantly higher than those of SVelter (57% for reported heterozygous CSVs and 49% for reported homozygous CSVs). Due to the randomized top-down approach, SVelter was able to discover some complete CSV events, but it may not explore all possibilities. Remarkably, we noted that Mako’s sensitivity was even better for randomized simulation (Figure 3E and F), which was consistent with our previous observation (Figure 3A and B). In particular, at 30× coverage, Mako detected 203% more heterozygous CSVs than that of SVelter (Figure 3E), probably due to the complementary graph edges for accurate CSV site discovery.

### Performance on real data

**Figure 4:**
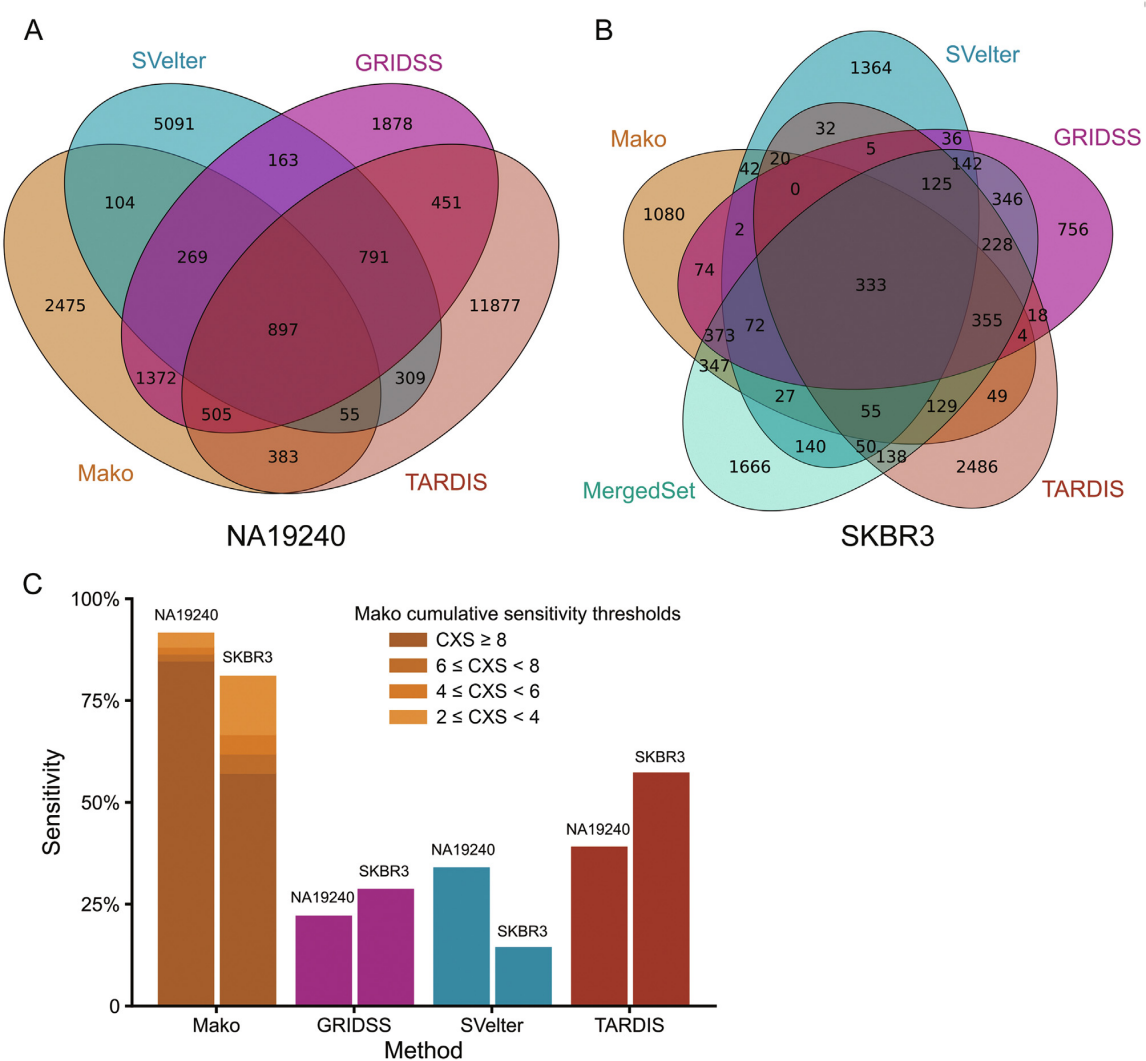
**Overview of performanceofMako, GRIDSS, SVelter, and TARDISon NA19240 and SKBR3** **A.** Venn diagram of callsets detected from NA19240 by four selected tools. **B.** Venn diagram of callsets detected from SKBR3 by four selected tools as well as MergedSet. The Venn diagrams are created by 50% reciprocal overlap via a publicly available tool Intervene with ‘‘–bedtools-options” enabled. The MergedSet is obtained from the original publication. **C.** The percentages of completely and uniquely discovered CSVs from the NA19240 and SKBR3 data, respectively. The results of Mako are shown according to different CXS thresholds.

We further compared Mako with SVelter, GRIDSS, and TARDIS on the whole-genome sequencing data of NA19240 and SKBR3. Firstly, we compared the callsets of different callers (Figures S14 and S15), and found that Mako shared most calls with GRIDSS (Figure 4A and B), which was consistent with our observation in simulated data (Figure 3). Furthermore, we examined the discovery completeness of 59 (NA19240) and 21 (SKBR3) benchmark CSVs (Table 1, Table S2; File S2). Because Manta and Lumpy contributed to the CSV benchmark sets, they were excluded from the comparison. The results showed that Mako performed the best for the two benchmark sets with different CXS thresholds, while TARDIS ranked second (Figure 4C). Given that Invdup and Disdup dominated the two benchmark sets (Table 1) and that TARDIS has designed specific models for these two types, TARDIS detected more events of these two duplication types than SVelter and GRIDSS. SVelter only detected three benchmark CSVs for SKBR3 because the randomized approach may not explore all combinations of CSVs. Based on the aforementioned observation, we concluded that the graph-based model-free strategy of Mako performed better than either randomized model (SVelter) or specific model (TARDIS) with few computational resources (Figure S16).

**Table 1:** **Summary of benchmark CSV**

**Type**	**Benchmark summary**		**Description**
	**NA19240**	**SKBR3**	
Disdup	15	12	Dispersed duplication
Invdup	18	–	Inverted duplication
DelInv	7	5	Deletion associated with inversion
DelDisdup	5	1	Deletion associated with dispersed duplication
DelInvdup	1	–	Deletion associated with inverted duplication
DisdupInvdup	2	2	Dispersed duplication with inverted duplication
InsInv	1	–	Insertion associated with inversion
Tantrans	1	–	Adjacent segment swap
DelSpaDel	8	1	Two deletions with inverted or non-inverted spacer
TanDisdup	1	–	Tandem dispersed duplication

### CSV subgraph illustrates breakpoint connections

**Figure 5:**
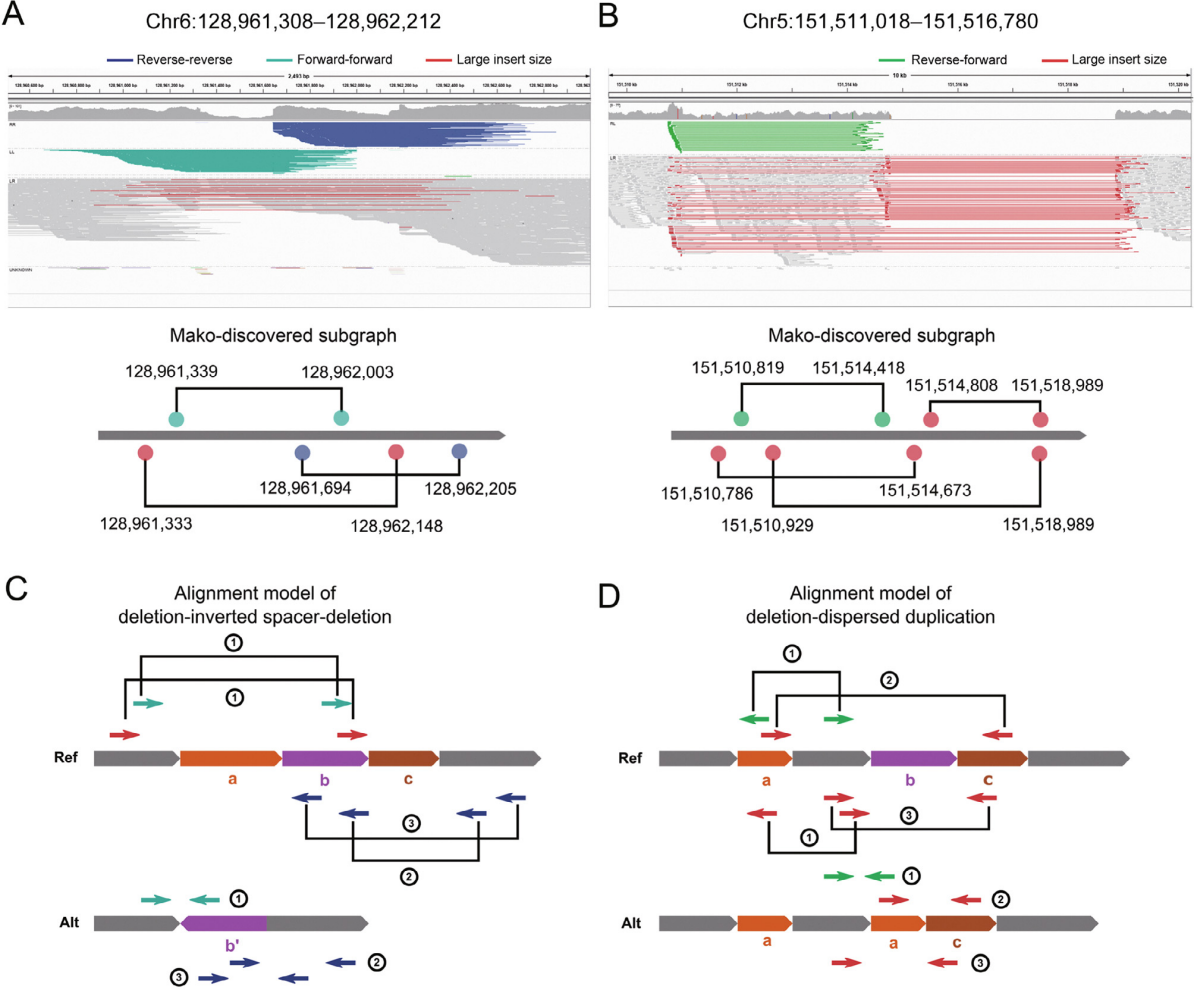
**Two representative CSV subgraphs identified by Mako** **A.** and **B.** Top: IGV views of the two representative CSV events. The alignments are grouped by read-pair orientation. Bottom: subgraph structures discovered by Mako. The colored circles and solid lines are nodes and edges in the subgraph. **C.** The alignment model of two deletions with an inverted spacer. **D.** The alignment model of deletion associated with dispersed duplication. In (C) and (D), short arrows are paired-end reads that span breakpoint junctions, and their alignments are shown on the Ref genome with the corresponding ID in the circle. Noted that a single ID may have more than one corresponding abnormal alignment types on the Ref genome. IGV, Itegrative Genomics Viewer.

Having demonstrated the performance of Mako on simulated and real data, we surveyed the landscape of CSVs from three individual genomes. Specifically, CSVs from autosomes were selected from Mako’s callset with more than one edge connection type observed in the subgraph, leading to 403, 609, and 556 events for HG00514, HG00733, and NA19240, respectively (Figure S17; Table S3). We systemically evaluated all CSV events in HG00733 via experimental and computational validations as well as manual inspections (File S3). For experimental validation, we successfully designed primers for 107 CSVs (Table S4), where 15 out of 21 (71%) CSVs were successfully amplified and validated by Sanger sequencing (Table 2, Tables S5 and S6; File S4). The computational validation showed up to 87% accuracy (Figure S4; Table 3, Tables S5, S7 and S8), indicating that a combination of methods and external data is necessary for comprehensive CSV validation. Further analysis showed that the medians of experimental and computational breakpoint shift were 13 bp and 26 bp, respectively (Figure S18). We observed that approximately 54% of CSVs were found in either short tandem repeat (STR) or variable number tandem repeat (VNTR) regions, contributing to 75% of all events inside the repetitive regions (Figure S17). For the connection types, more than half of the events contain Dup and Ins edges in the graph (Figure S17), indicating duplication-involved sequence insertion. Moreover, around 40% of the events contain Del edges (Figure S17), showing connections of two distant segments derived from either Dup or Inv events. We further examined whether the CSV subgraph depicts the connections for each CSV via discordant read-pairs. Interestingly, we observed two representative events with four breakpoints at chr6:128,961,308–128,962,212 and chr5:151,511,018–151,516,780 from NA19240 and SKBR3, respectively (Figure 5). Both events were correctly detected by Mako, but missed by SVelter and reported more than once by GRIDSS and TARDIS (Table S9). In particular, the CSV at chr6:128,961,308–128,962,212 that consists of two deletions and an inverted spacer (DelSpaDel) was reported twice and five times by GRIDSS and TARDIS. The event at chr5:151,511,018–151,516,780 that consists of Del and Disdup was reported four and three times by GRDISS and TARDIS. These redundant predictions complicated and misled downstream functional annotations. On the contrary, Mako was able to completely detect the aforementioned two CSV events and also capable of revealing the breakpoint connections of CSVs encoded in the subgraphs. The aforementioned observations suggest that Mako’s subgraph representation is interpretable, from which we can characterize the breakpoint connections for a given CSV event.

**Table 2:** **Summary of experimentally validatedCSVs**

**Chromosome**	**Start**	**End**	**Mako type**
Chr1	81,194,398	81,195,874	Del, Inv
Chr2	119,659,504	119,661,322	Del, Dup
Chr3	146,667,093	146,667,284	Del, Dup
Chr5	141,480,327	141,483,116	Del, Dup
Chr7	1,940,931	1,941,009	Dup, Ins
Chr9	29,591,409	29,593,057	Del, Inv
Chr10	14,568,488	14,568,677	Dup, Ins
Chr12	71,315,482	71,316,928	Del, Inv
Chr12	77,989,900	77,994,324	Del, Inv
Chr13	74,340,759	74,342,810	Del, Dup
Chr16	78,004,459	78,007,456	Del, Dup
Chr17	34,854,438	34,855,851	Del, Inv
Chr17	48,538,270	48,540,171	Del, Dup
Chr18	72,044,575	72,045,937	Del, Dup
Chr21	26,001,844	26,001,844	Del, Inv

*Note*: Del, deletion; Ins, insertion; Dup, duplication; Inv, inversion.

**Table 3:** **Summary of experimental and computational validations as well as manual inspections for CSVs**

**Validation strategy**		**Total**	**Valid**	**Invalid**	**Inconclusive**
Experimental (PCR succeeded)		21	15 (71%)	6 (29%)	–
Computational	ONT reads	609	256 (42%)	–	353 (58%)
	HiFi contigs		414 (68%)	195 (32%)	–
	ONT reads or HiFi contigs		533 (87%)	76 (13%)	–
Manual	HiFi reads	609	440 (72%)	169 (28%)	–

*Note*: ONT, Oxford Nanopore Technologies; HiFi, Pacific Biosciences high-fidelity.

### Contribution of homology sequence in CSV formation

**Figure 6:**
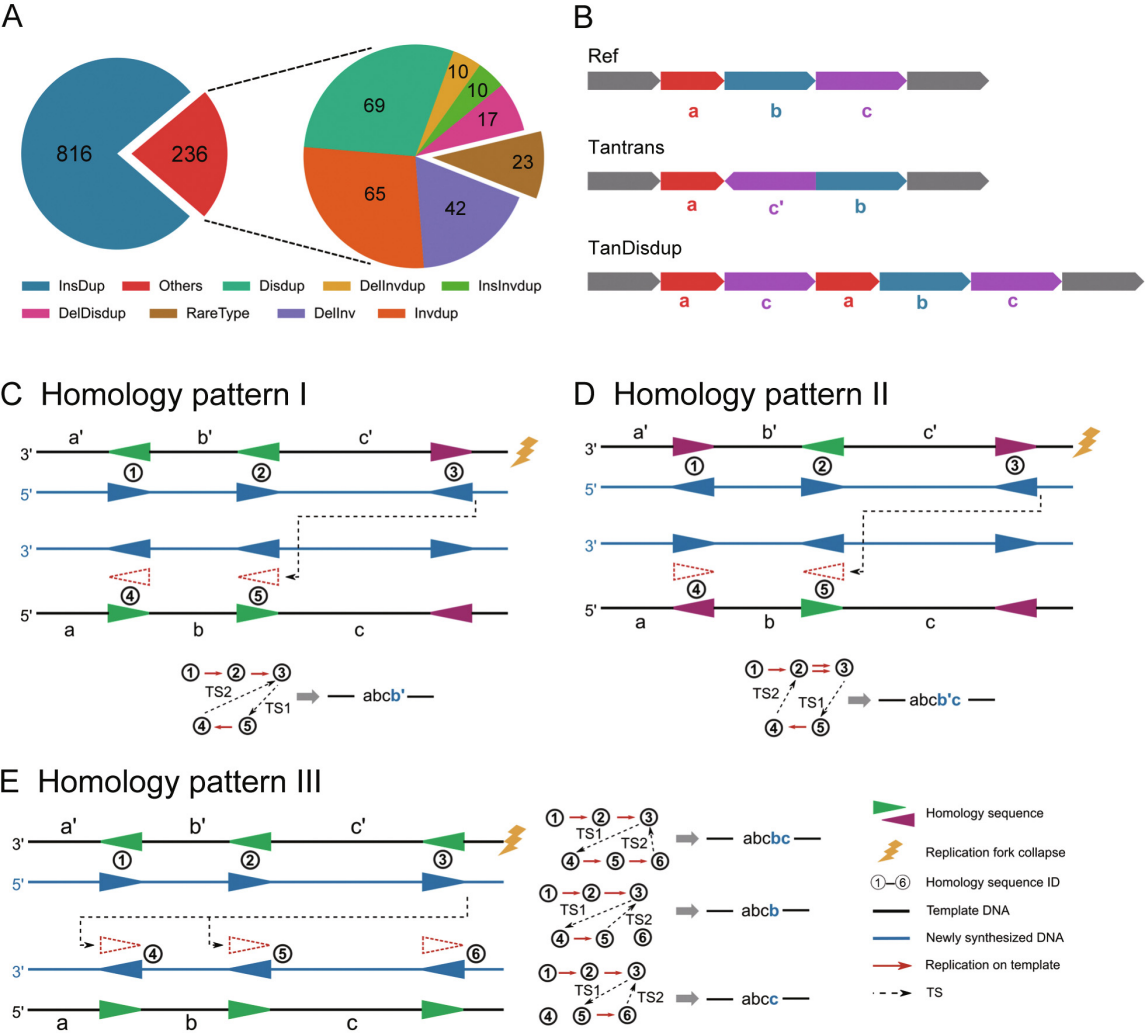
**Overview of Mako’s CSV discoveries from three healthy samples and proposed CSV formation mechanisms** **A.** Summary of discovered CSV types. These types are reconstructed by PacBio HiFi reads, where a type with less than 10 events is summarized as RareType. **B.** Diagrams of two novel and rare CSV types discovered by Mako. In particular, Mako finds three Tantrans events and only one TanDisdup event. **C.–E.** Different replication diagrams explaining the impact of homology pattern for MMBIR-produced CSVs. In these diagrams, sequence abc has been replicated before the replication fork collapse (flash symbol). The single-strand DNA at the DNA DSB starts searching for homology sequence (purple and green triangles) to repair. The a forementioned procedure is explicitly explained as a replication graph, where nodes are homology sequences and edges keep track of TS (dotted arrow lines) as well as the normal replication at different strands (red lines). If there are two red lines between two nodes, the sequence between these two nodes will be replicate twice, as shown in (D). InsDup, insertion associated with duplication; Disdup, dispersed duplication; Invdup, inverted duplication; DelInvdup, deletion associated with inverted duplication; InsInvdup, insertion associated with inverted duplication; DelDisdup, deletion associated with dispersed duplication; DelInv, deletion associated with inversion; Tantrans, adjacent segment swap; TanDisdup, tandem dispersed duplication; MMBIR, microhomology-mediated break-induced replication; DSB, double-strand break; TS, template switch.

Given 1568 detected CSVs from three genomes (HG00514, HG00733, and NA19240), we further investigated the formation mechanisms of these CSVs. Ongoing studies have revealed that inaccurate DNA repair and the 2–33 bp long microhomology sequence at breakpoint junctions play an important role in CSV formation [18], [44], [45], [46], [47]. To further characterize CSVs’ internal structure and examine the impact of homology sequence on CSV formation, we manually reconstructed 1052 high-confident CSV calls given by Mako (252/403 from HG00514, 440/609 from HG00733, and 360/556 from NA19240) via Dotplots created by PacBio HiFi reads (Figure 6A, Figure S19; Table S10; File S3). The percentage of successfully reconstructed events was similar to the orthogonal validation rate, showing that CSVs detected by Mako were accurate, and the validation method was effective. The high-confident CSV callset contains 816 insertion associated with duplication (InsDup) events with both Ins and Dup edge connections. Further investigation revealed that these events contain irregular repeat sequence expansion, making them different from simple Ins or Dup events (Figure S20). Besides, we found two novel types, named adjacent segment swap (Tantrans) and tandem dispersed duplication (TanDisdup) (Figure 6B, Figures S21 and S22). We inferred that homology sequence-mediated inaccuracy replication was the major cause for these two types. Furthermore, we observed that 134 CSVs contain either Invdup or Disdup events (Table S10). These Invdup/Disdup-involved CSVs were mainly caused by microhomology-mediated break-induced replication (MMBIR) according to previous studies [18], [45], [48]. It was known that different homology patterns caused distinct CSV types (Figure 6C and D). Surprisingly, one particular homology pattern yielded multiple CSV types (Figure 6E). In particular situations of the three different homology patterns, DNA double-strand break (DSB) occurred after replication of fragment c. According to the MMBIR mechanism and template switch (TS) [22], [45], [46], [47], the pattern I (Figure 6C) and pattern II (Figure 6D) each yield one output, but pattern III (Figure 6E) produced three different outcomes. These results provide additional evidence for understanding the impact of sequence contents on DNA DSB repair, leading to a better understanding of diversity variants produced by CRISPR [49], [50].

## Discussion

Currently, short-read sequencing is significantly reduced in cost and has been applied to clinical diagnostics and large cohort studies [16], [51], [52]. However, CSVs from short-read data are not fully explored due to the methodology limitations. Although long-read sequencing technologies bring us promising opportunities to characterize CSVs [13], [14], [38], their application is currently limited to small-scale projects, and the methods for CSV discovery are also underdeveloped. As far as we know, NGMLR combined with Sniffles is the only pipeline that utilizes the model-match strategy to discover two specific forms of CSVs, namely DelInv and Invdup. Therefore, there is a strong demand in the genomic community to develop effective and efficient algorithms to detect CSVs using short-read data. It should be noted that CSV breakpoints might come from either single haplotype or different haplotypes, where two simple SVs from different haplotypes lead to false positives (Figure S23). This may increase the false discovery rate due to a lack of haplotype information. Therefore, the combination of short-read and long-read sequencing might improve CSV discovery and characterization.

To sum up, we develop Mako, utilizing the graph-based pattern growth approach, for CSV discovery with 70% accuracy and 20 bp median breakpoint shift. To the best of our knowledge, Mako is the first algorithm that utilizes the bottom-up guided model-free strategy for SV discovery, avoiding the complicated model and match procedures. Given the fact that CSVs are largely unexplored, Mako presents opportunities to broaden our knowledge of genome evolution and disease progression.

## Code availability

Mako is implemented in Java 1.8, and it is available at https://github.com/xjtu-omics/Mako. It is free for non-commercial use by academic, government, and non-profit/not-for-profit institutions. A commercial version of the software is available and licensed through Xi’an Jiaotong University. All scripts used in this study are also included in the Github repository, and a detailed description of using these scripts and other tools is provided.

## CRediT author statement

**Jiadong Lin:** Methodology, Software, Formal analysis, Data curation, Visualization, Writing - original draft, Writing - review & editing. **Xiaofei Yang:** Methodology, Writing - original draft, Writing - review & editing. **Walter Kosters:** Methodology, Writing - original draft. **Tun Xu:** Data curation. **Yanyan Jia:** Validation. **Songbo Wang:** Validation, Formal analysis. **Qihui Zhu:** Validation. **Mallory Ryan:** Validation. **Li Guo:** Writing - original draft. **Chengsheng Zhang:** Validation, Writing - original draft. **HGSVC:** Resources. **Charlse Lee:** Resources, Writing - original draft. **Scott E. Devine:** Resources. **Evan E. Eichler:** Resources. **Kai Ye:** Conceptualization, Resources, Supervision, Project administration, Funding acquisition. All authors have read and approved the final manuscript.

## Competing Interests

The authors have declared no competing interests.

## Supplementary material

Supplementary File S1 **Supplementary note for Mako**

Supplementary File S2 **IGV view and PacBio reads Dotplot of each benchmark CSVs**

Supplementary File S3 **Dotplot used for manually inspection of CSVs from HG00733**

Supplementary File S4 **PCR results and visualization of CSV breakpoint validated through Sanger sequencing**

Supplementary Figure S1 **A toy example to explain the pattern growth process**

Supplementary Figure S2 **Workflow of CSV simulation**

Supplementary Figure S3 **Hierarchical clustering tree view of SVs from NA19240 chromosome 1**

Supplementary Figure S4 **Hierarchical clustering tree view of SVs from SKBR3 chromosome 1**

Supplementary Figure S5 **The curve plot between cluster distance cutoff and number of clusters for SVs from NA19240 autosomes**

Supplementary Figure S6 **The curve plot between cluster distance cutoff and number of clusters for SVs from SKBR3 autosomes**

Supplementary Figure S7 **Diagram of selecting primers for each CSV**

Supplementary Figure S8 **Examples of PCR electrophoretic bands visualized under the UV light**

Supplementary Figure S9 **Workflow of HiFi assembly K-mer validation**

Supplementary Figure S10 **A screenshot using Gepard to investigate a deletion associated with inversion event**

Supplementary Figure S11 **Dotplot patterns used to identify CSVs at highly repetitive regions**

Supplementary Figure S12 **Dotplot patterns used to identify SVs at highly repetitive regions**

Supplementary Figure S13 **Example call at high repetitive regions that labeled as NA by VaPoR at chr6:165,749,273-165,749,500**

Supplementary Figure S14 **Size distribution of SV in the range [50bp, 10Kbp] from NA19240**

Supplementary Figure S15 **Size distribution of SV in the range [50bp, 10Kbp] from SKBR3 breast cancer cell line**

Supplementary Figure S16 **Running time comparison between different methods A.** Runtime comparison on simulated data at 30× coverage. **B.** Runtime of Mako on real data at different coverage. The time baseline is decided by copying the original BAM to another location. **C.** Memory usage of Mako on real data at different coverage.

Supplementary Figure S17 **Repeat annotation and connection types of Mako****-****detected CSVs from three samples A.** Repeat annotation of CSVs detected from three genomes. **B.** Mako predicted CSV types of three genomes.

Supplementary Figure S18 **Mako****-**detected CSV breakpoint resolution compared to HiFi contig (*K*-mer) and experiment

Supplementary Figure S19 **Mako-detected CSV and PacBio HiFi read refined CSV size****distribution**

Supplementary Figure S20 **Example of an insertion associated with duplication event (InsDup) at chr6:165,749,273-165,749,500**

Supplementary Figure S21 **The IGV view and sequence dot-plot of the adjacent segment swap from NA19240 at Chr7:83,316,809-83,317,466**

Supplementary Figure S22 **The IGV view and sequence dot-plot of the tandem dispersed duplication from NA19240 at Chr17:43,359,104-43,365,253**

Supplementary Figure S23 **Examples to show the difference of CSV breakpoints from single haplotype or two haplotypes A.** Diagram of two simple SVs at different haplotypes. **B.** Diagram of complex SV at the same haplotype

Supplementary Table S1 **Parameters used for creating the CSV benchmarks for NA19240 and SKBR3**

Supplementary Table S2 **CSV benchmarks for NA19240 and SKBR3**

Supplementary Table S3 **Mako detected CSVs for HG00733, HG00514, and NA19240**

Supplementary Table S4 **CSVs of successfully designed primers**

Supplementary Table S5 **Summary of experimental and computational validation as well as manual inspections of HG00733**

Supplementary Table S6 **Comparing Mako detected breakpoints with PCR validated breakpoints**

Supplementary Table S7 **Comparing Mako breakpoints with K-mer realigned breakpoints**

Supplementary Table S8 **Details of VaPoR validation results**

Supplementary Table S9 **Details of breakpoints for the two examples in Figure 5**

Supplementary Table S10 **Summary of PacBio HiFi reads refined CSV types**
