# Supplementary material for: Mako: A Graph-based Pattern Growth Approach to Detect Complex Structural Variants
Source: Genomics Proteomics Bioinformatics. 2021 Jul 3;20(1):205–18. doi: 10.1016/j.gpb.2021.03.007 (PMC9510932; doi:10.1016/j.gpb.2021.03.007)
Supplement: Supplementary File S2 — IGV view and PacBio reads Dotplot of each benchmark CSVs [file mmc2.pdf]

IGV view and PacBio reads Dotplot of each benchmark CSVs

NA19240 benchmark CSVs

chr1:16081190-16082430

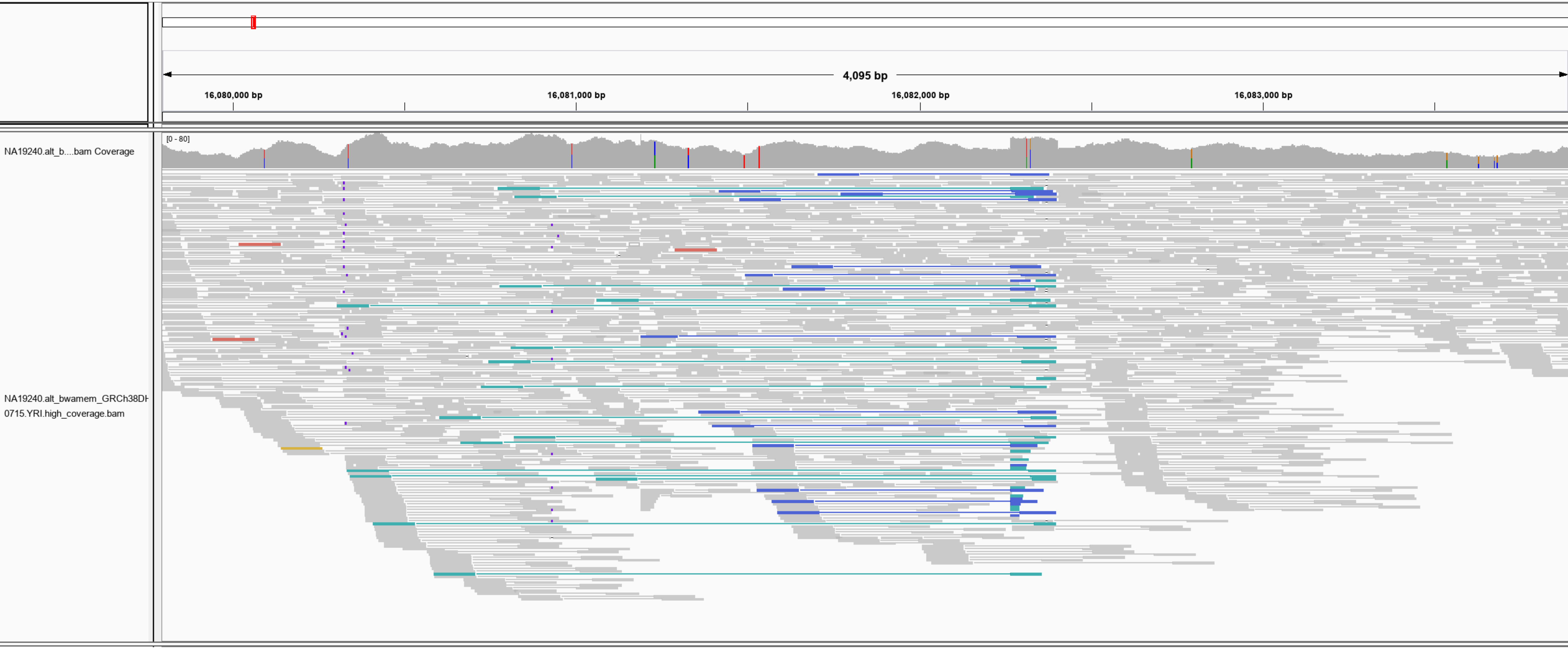

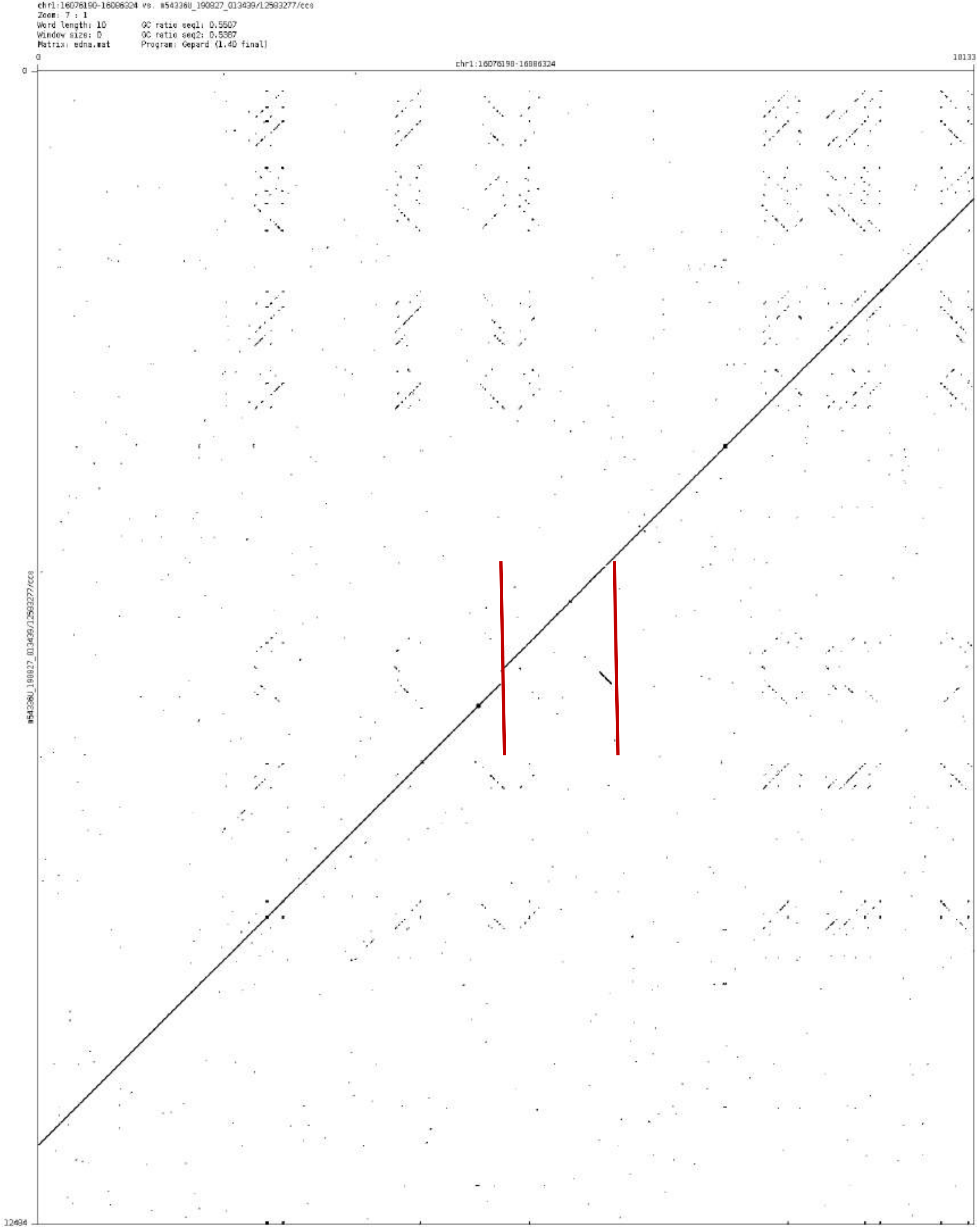
chr1:16076190-16086324 vs. m54336U_190827_013439/12583277/ccs
Zoom: 7 : 1
Word length: 10
Window size: 0
Matrix: edna.mat
GC ratio seq1: 0.5507
GC ratio seq2: 0.5367
Program: Gepard (1.40 final)
0
chr1:16076190-16086324
18133
0
m54336U_190827_013439/12583277/ccs
12494

chr1:41278200-41279034

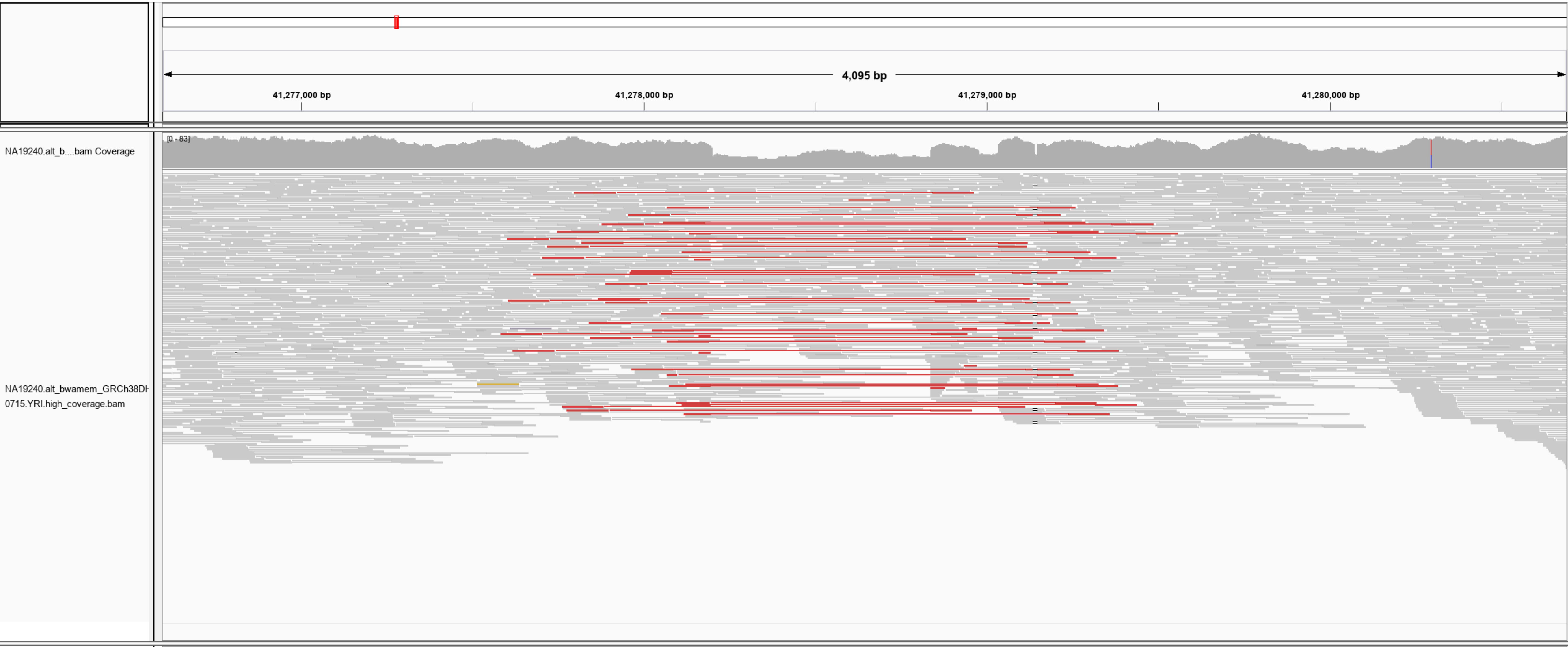

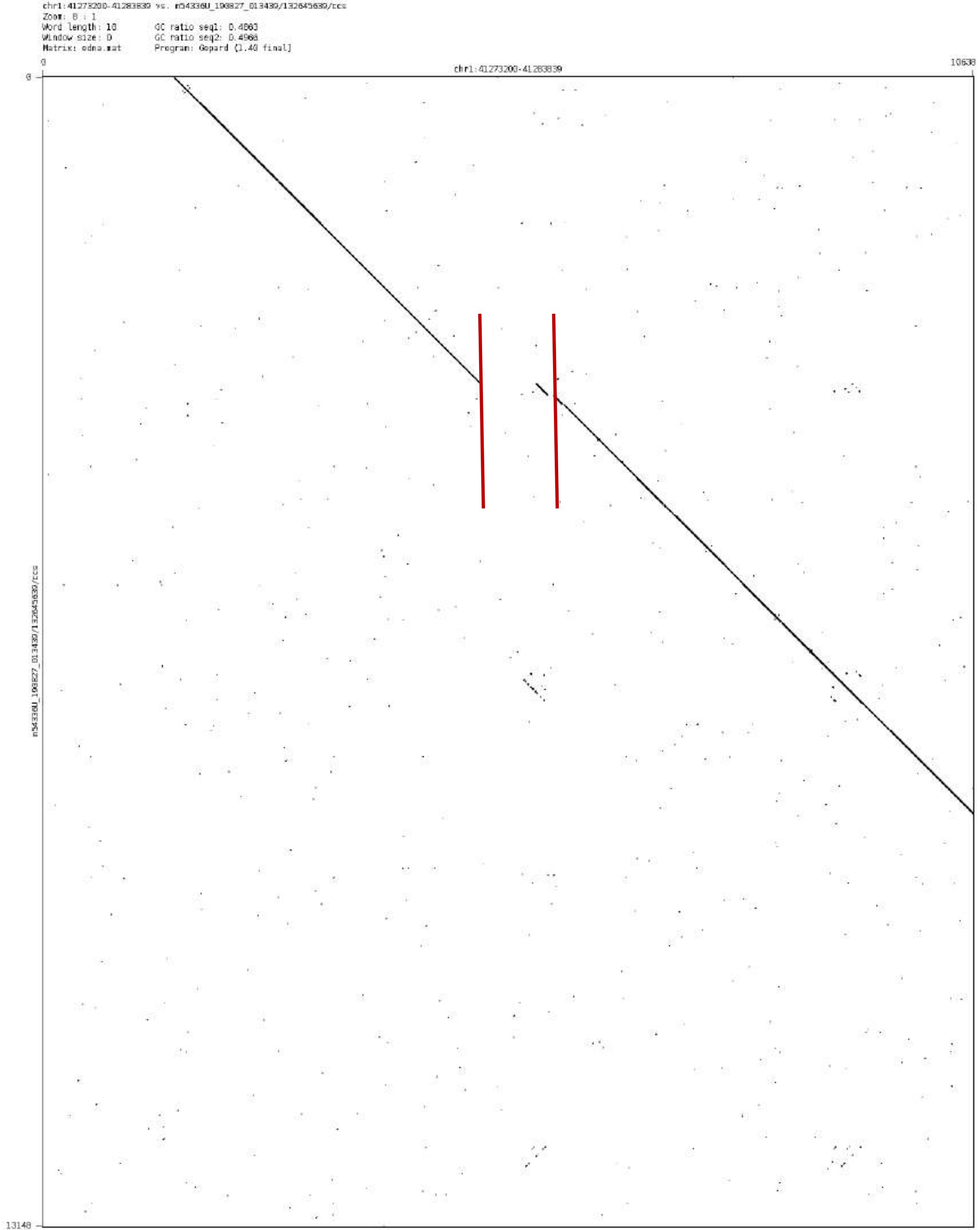
chr1:41273200-41283839 vs. m54336U_190827_013439/132645639/ccs
Zoom: 8 : 1
Word length: 10
Window size: 0
Matrix: edna.mat
GC ratio seq1: 0.4863
GC ratio seq2: 0.4968
Program: Gepard (1.40 final)
0
chr1:41273200-41283839
10638
0
m54336U_190827_013439/132645639/ccs
13148

chr1:43593621-43594291

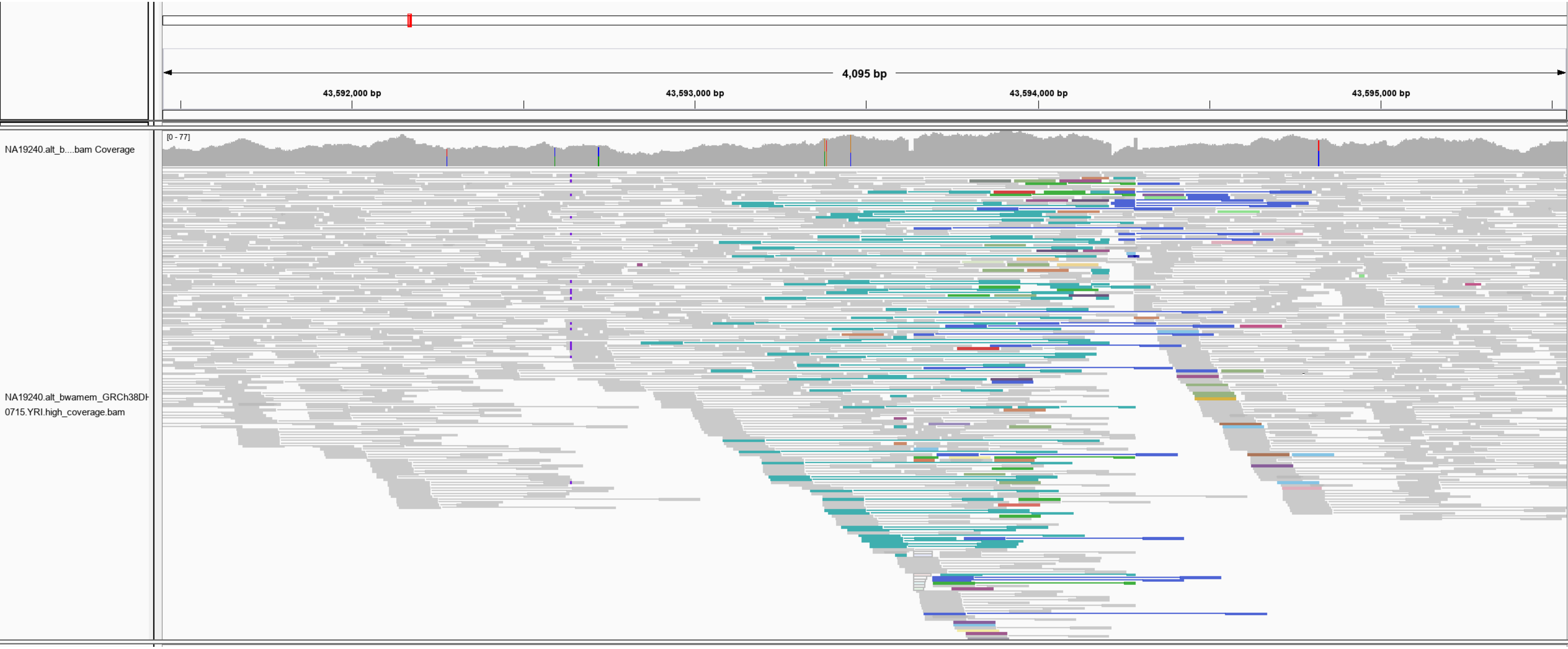

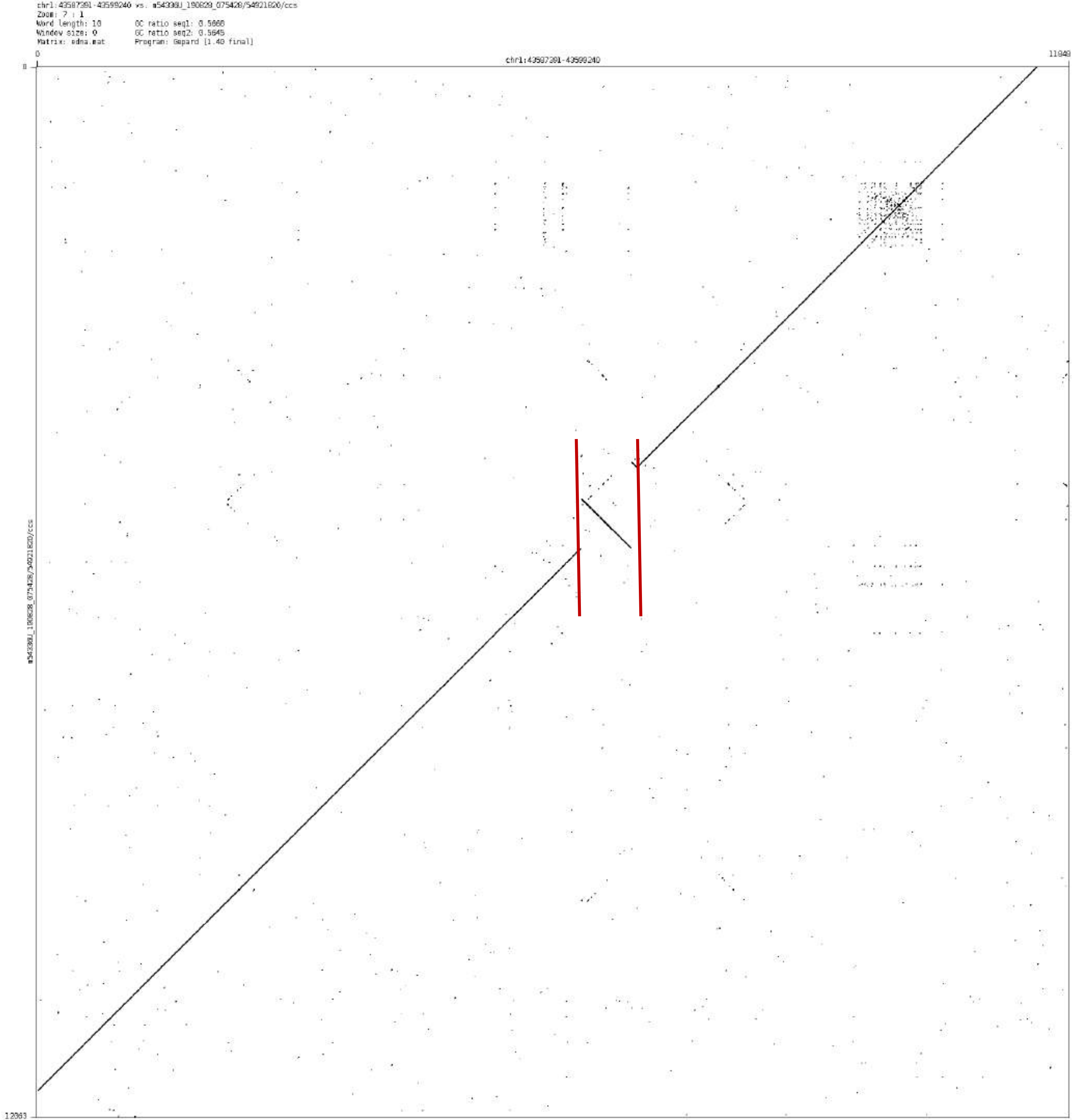

chr1:43587391-43599240 vs. m54338U_190828_075428/54921820/ccs
Zoom: 7 : 1
Word length: 10
GC ratio seq1: 0.5668
Window size: 0
GC ratio seq2: 0.5645
Matrix: edna.mat
Program: Gepard [1.40 final]
0
chr1:43587391-43599240
11849
0
12063
m54338U_190828_075428/54921820/ccs

chr1:175231315-175232795

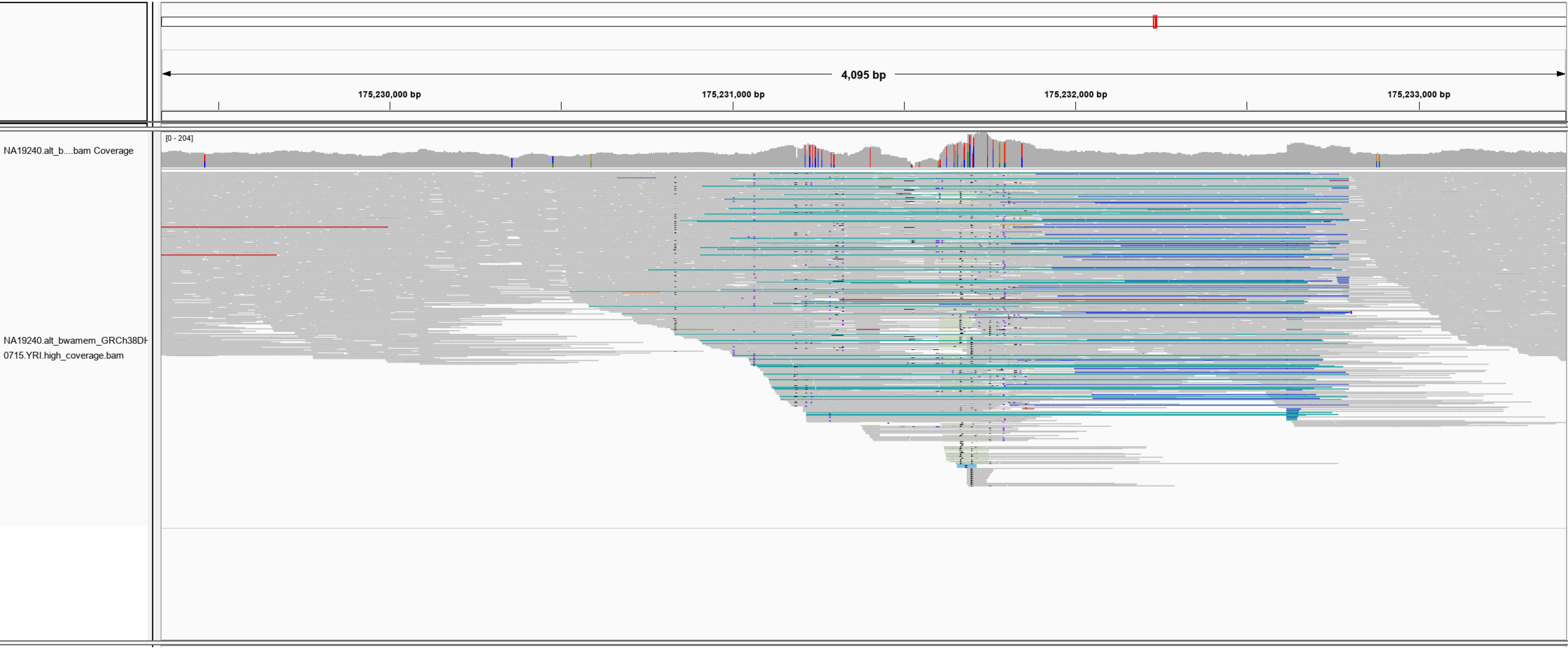

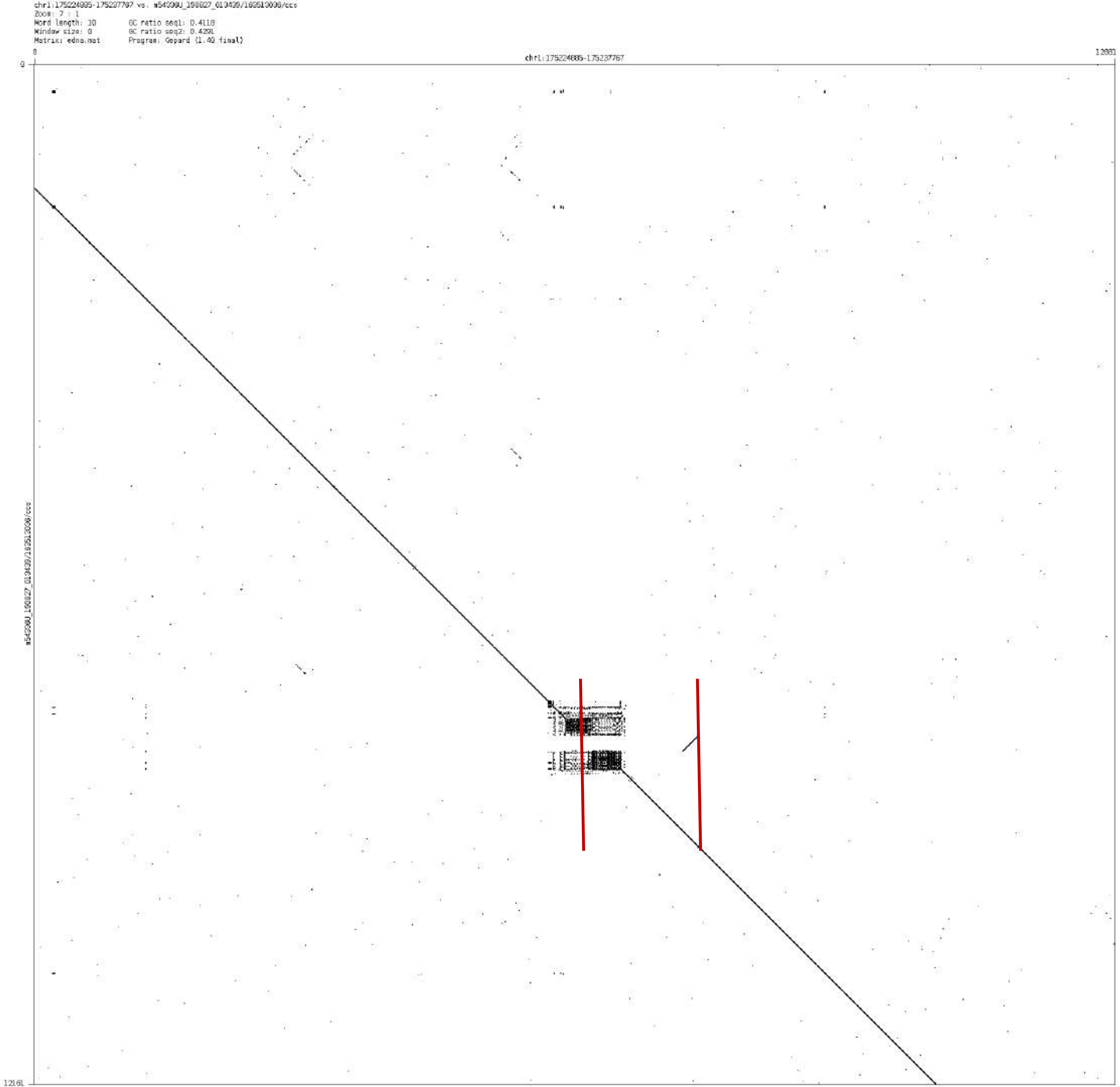
chr1:175224885-175237767 vs. m54330U_190827_013439/163513098/ccs
Zoom: 7 : 1
Word length: 10
Window size: 0
Matrix: edna.mat
GC ratio seq1: 0.4118
GC ratio seq2: 0.4291
Program: Gepard (1.40 final)
0
chr1:175224885-175237767
12881
0
m54330U_190827_013439/163513098/ccs
12161

chr1:187495696-187497571

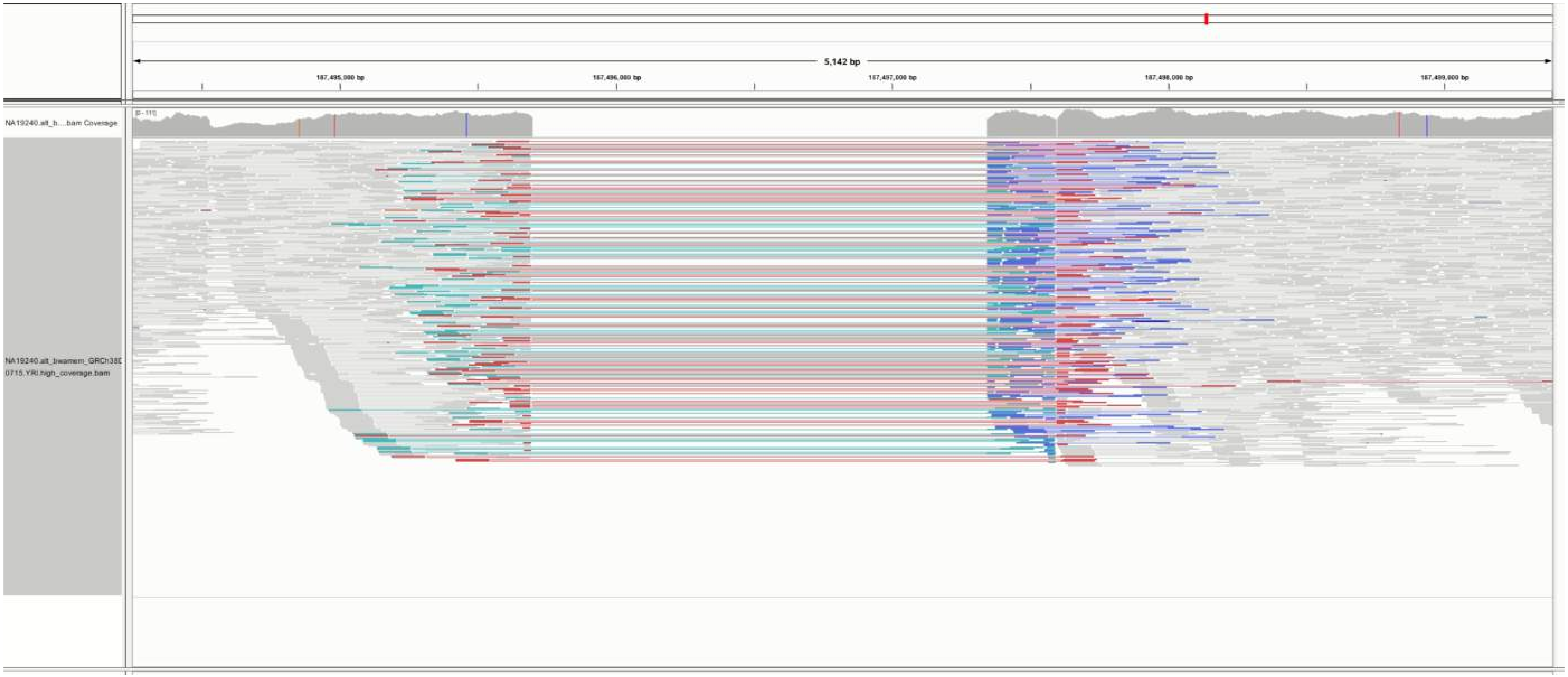

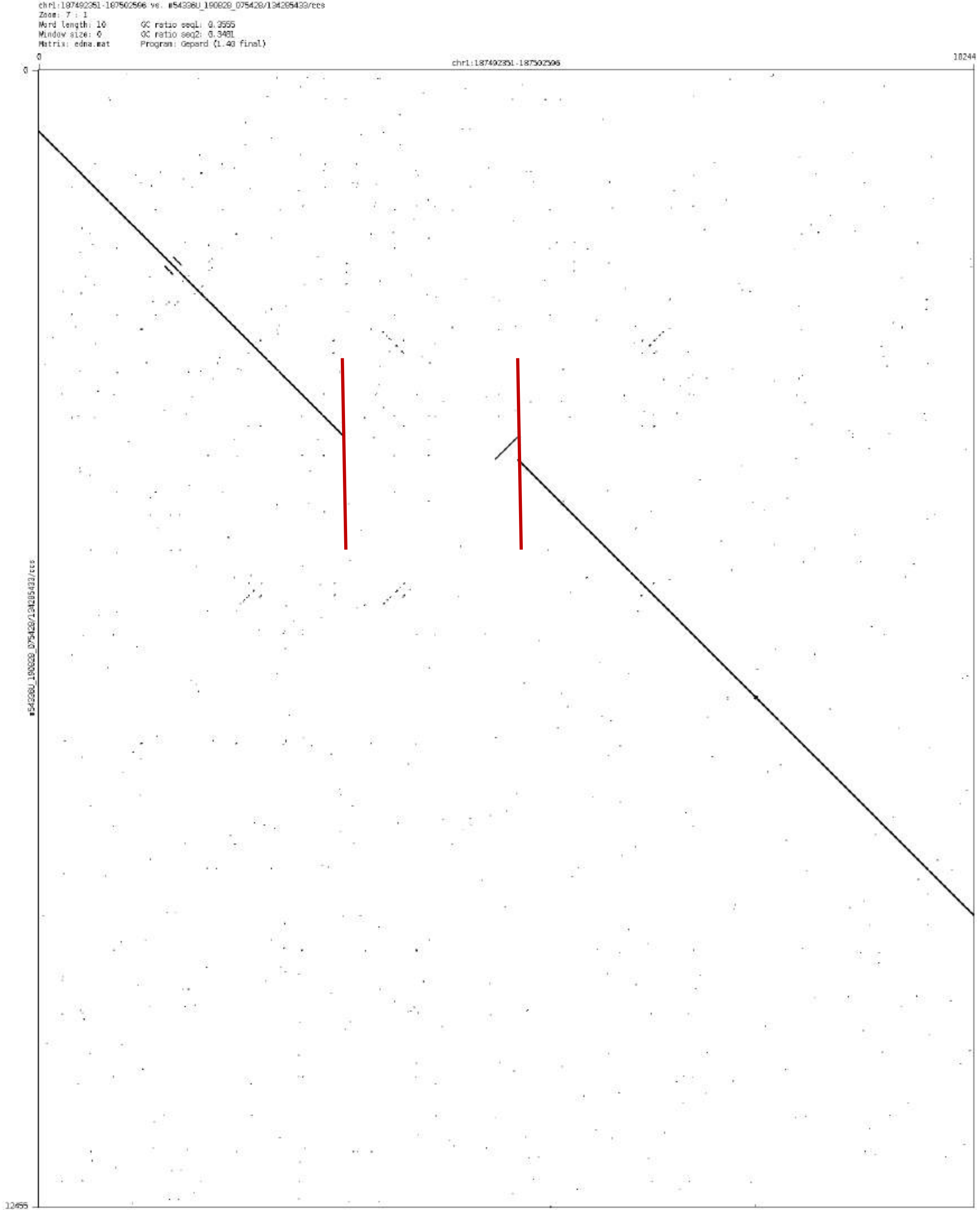
chr1:187492351-187502596 vs. m54336U_190828_075428/134285433/ccs
Zoom: 7 : 1
Word length: 10
Window size: 0
Matrix: edna.mat
GC ratio seq1: 0.3555
GC ratio seq2: 0.3481
Program: Gepard (1.40 final)
0
chr1:187492351-187502596
10244
0
12455

chr1:209761615-209762730

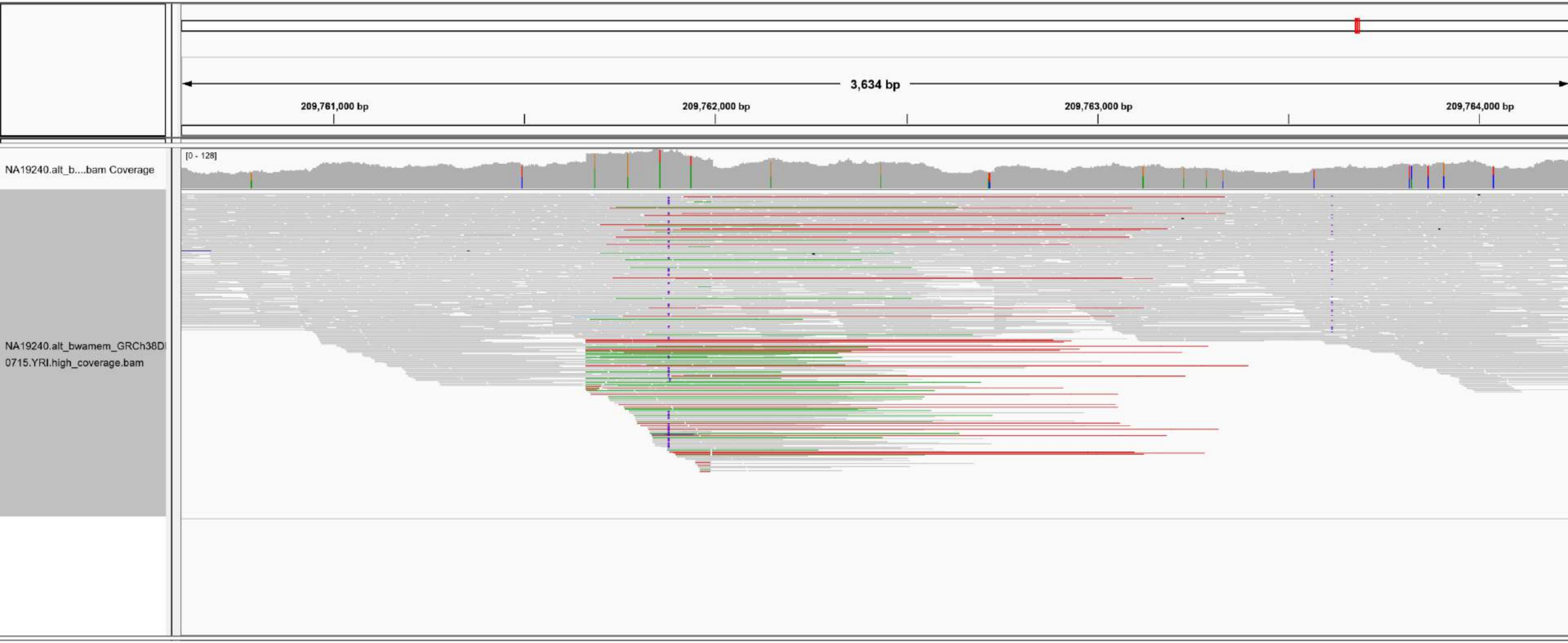

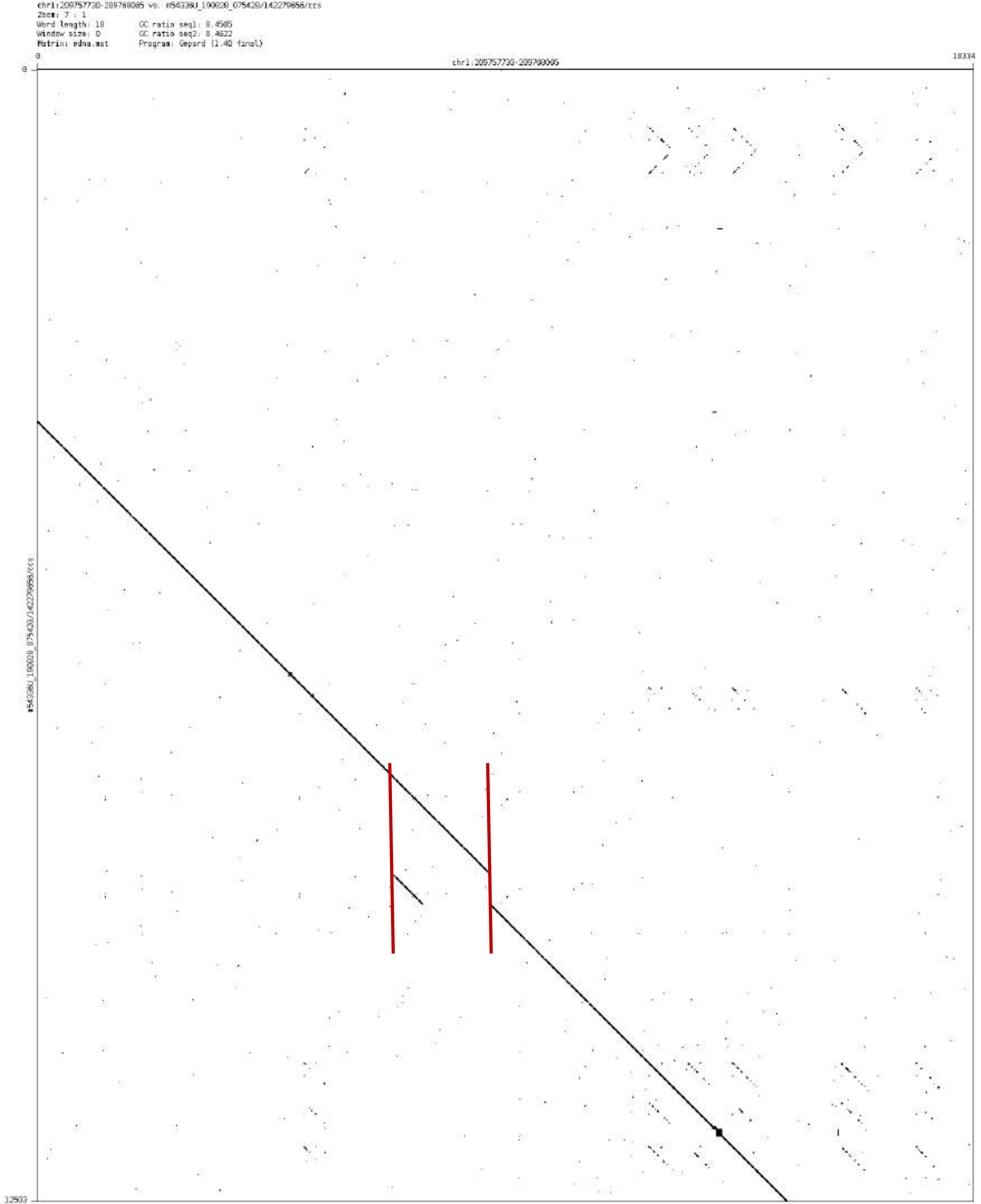

chr1:209757730-209768085 vs. m54336U_190828_075428/142279656/ccs
Zoom: 7 : 1
Word length: 10
Window size: 0
Matrix: edna.mat
GC ratio seq1: 0.4585
GC ratio seq2: 0.4622
Program: Gepard (1.40 final)
0
18334
chr1:209757730-209768085
0
12503
m54336U_190828_075428/142279656/ccs

chr1:237390170-237392642

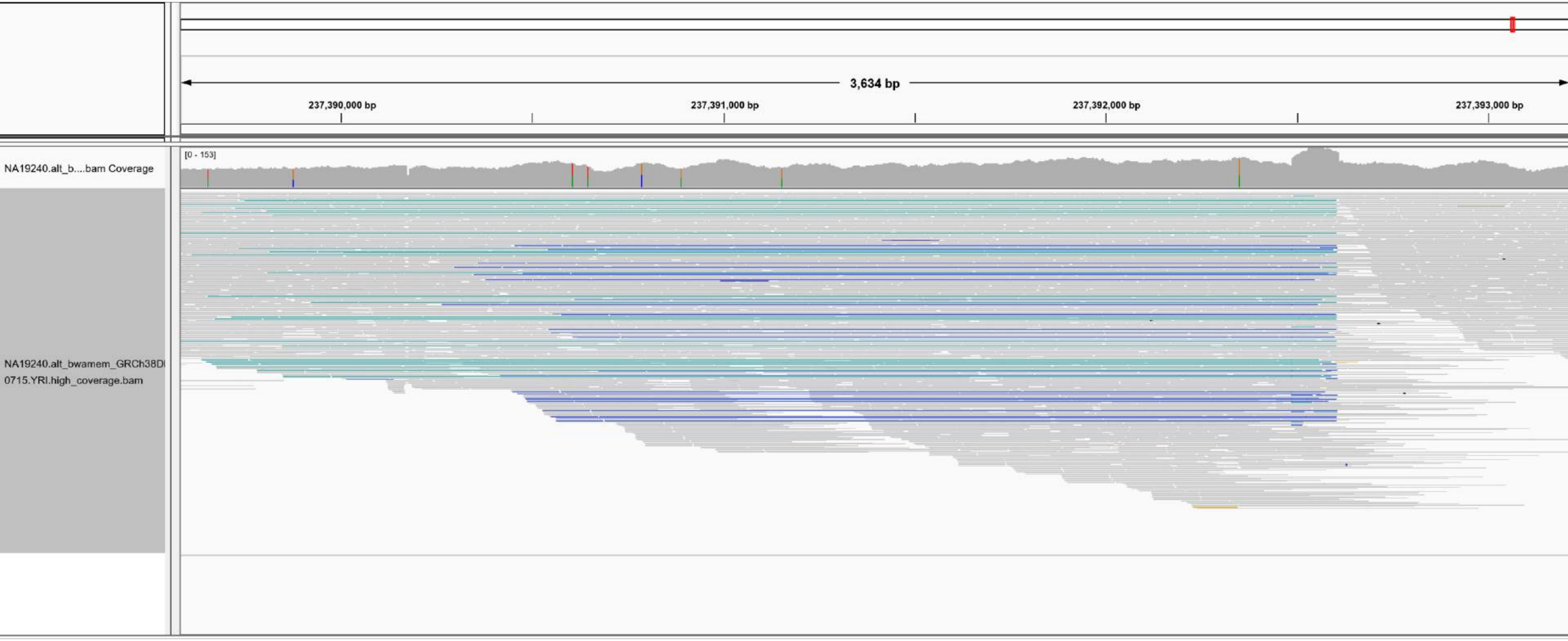

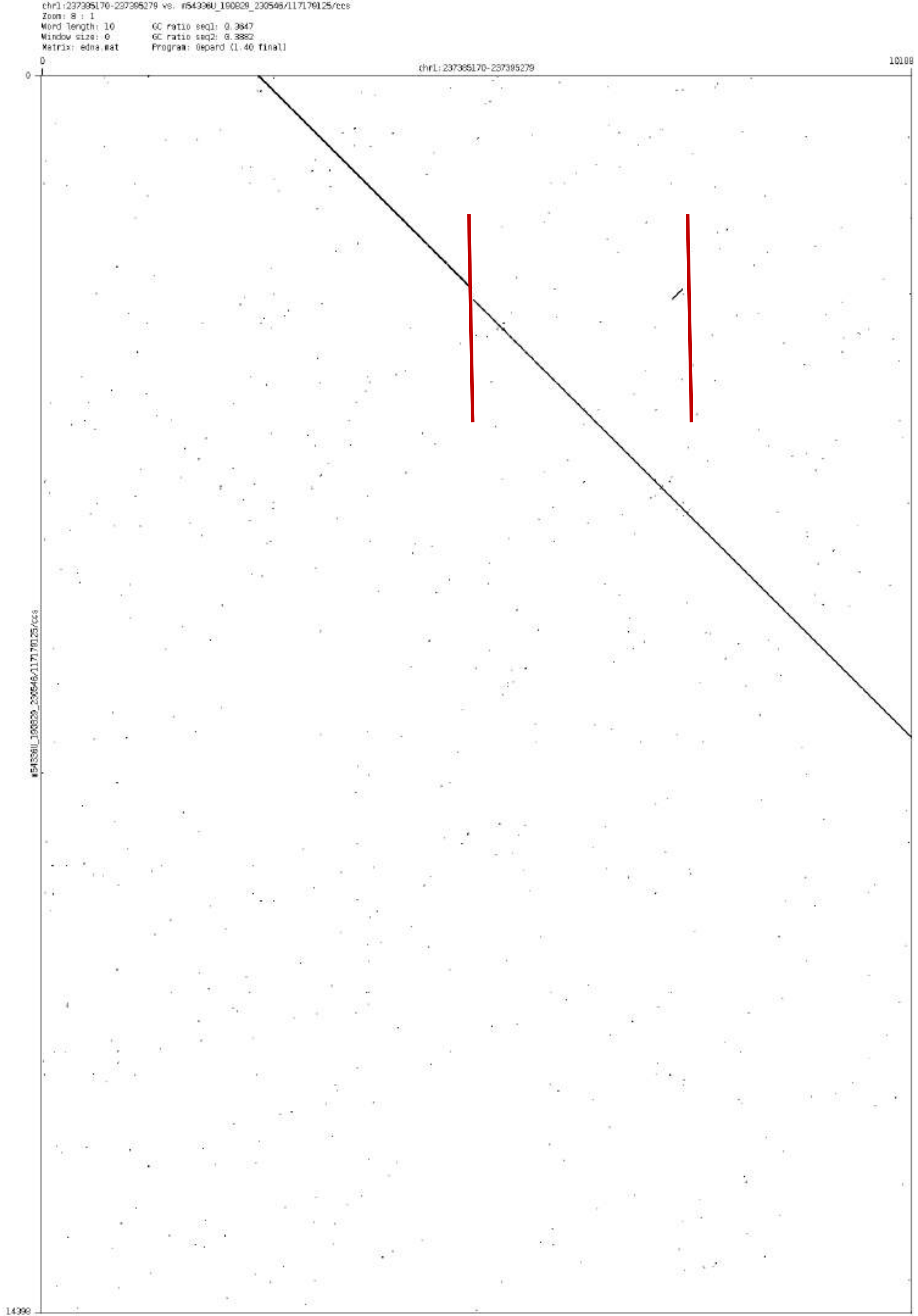
chr1:237395170-237395279 vs. m54336U_190829_230546/117179125/ccs
Zoom: 8 : 1
Word length: 10
Window size: 0
Matrix: edna.mat
GC ratio seq1: 0.3647
GC ratio seq2: 0.3882
Program: Gepard (1.40 final)
0
chr1:237395170-237395279
10188
0
14390

chr1:239952898-239953380

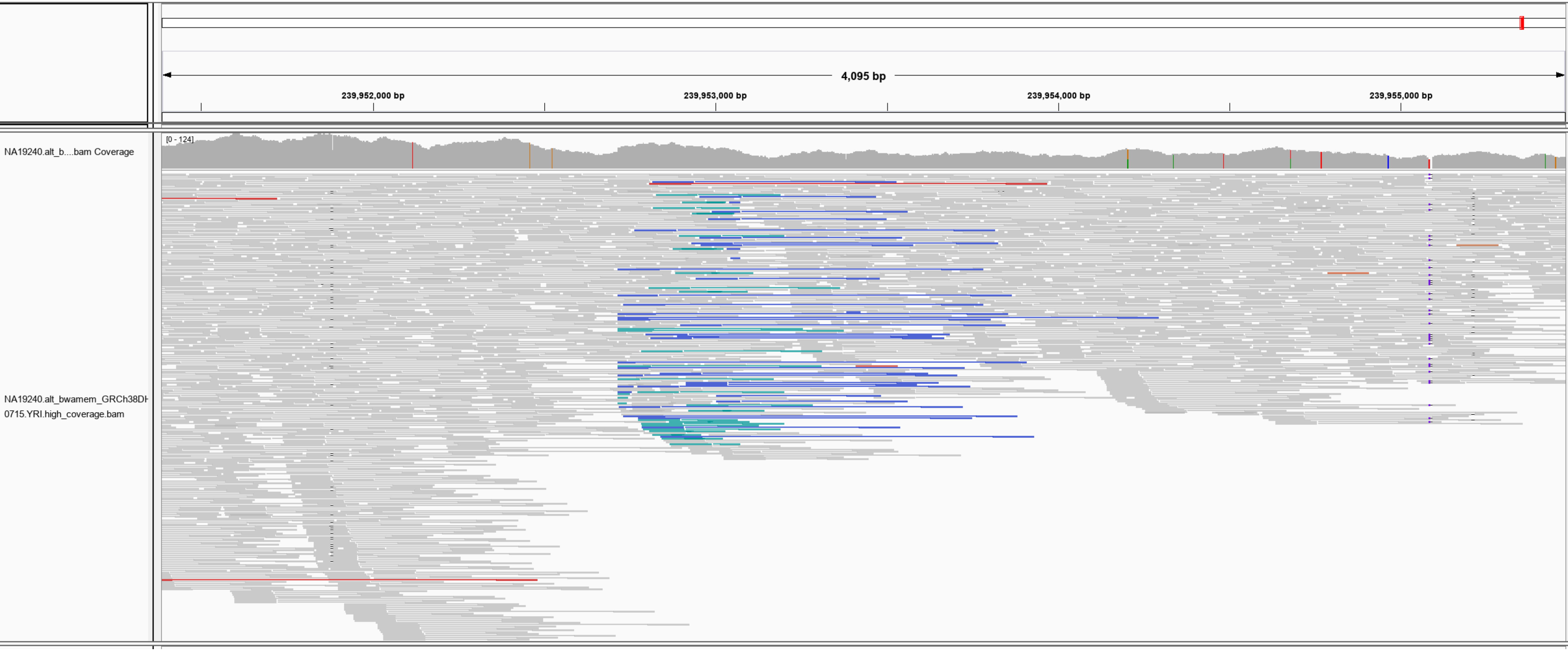

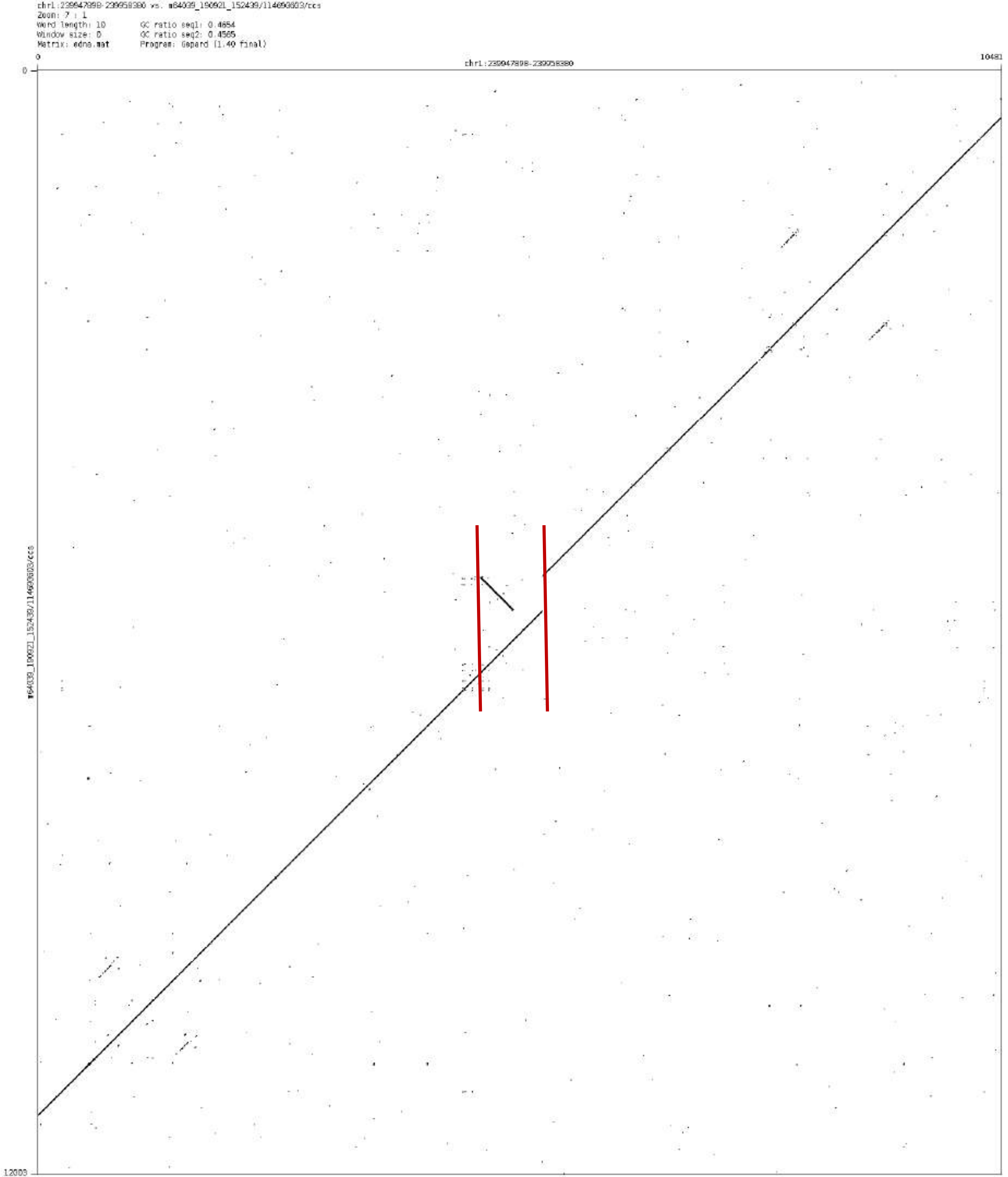
chr1:239947898-239958380 vs. m64039_190921_152439/114690803/ccs
Zoom: 7 : 1
Word length: 10
Window size: 0
Matrix: edna.mat
GC ratio seq1: 0.4654
GC ratio seq2: 0.4565
Program: Gepard (1.40 final)
0
chr1:239947898-239958380
10481
0
m64039_190921_152439/114690803/ccs
12003

chr1:248795414-248799304

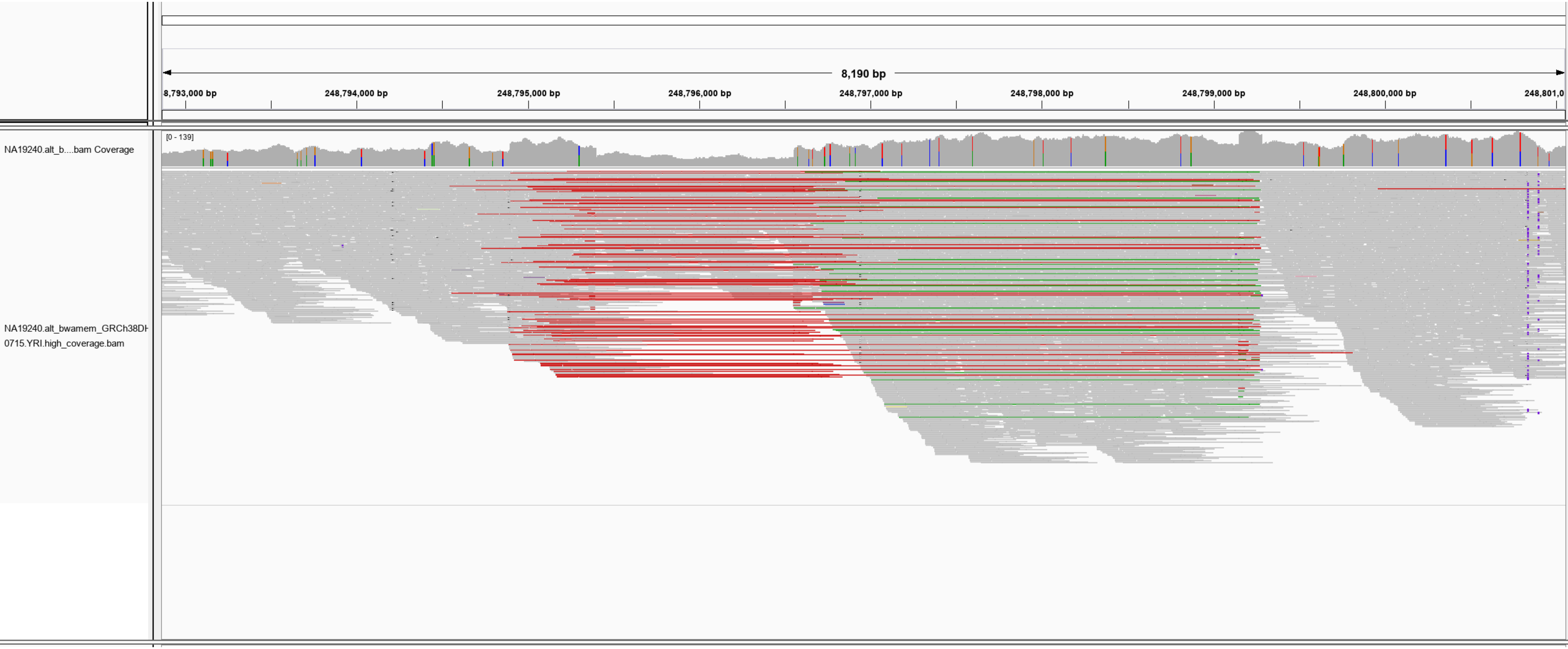

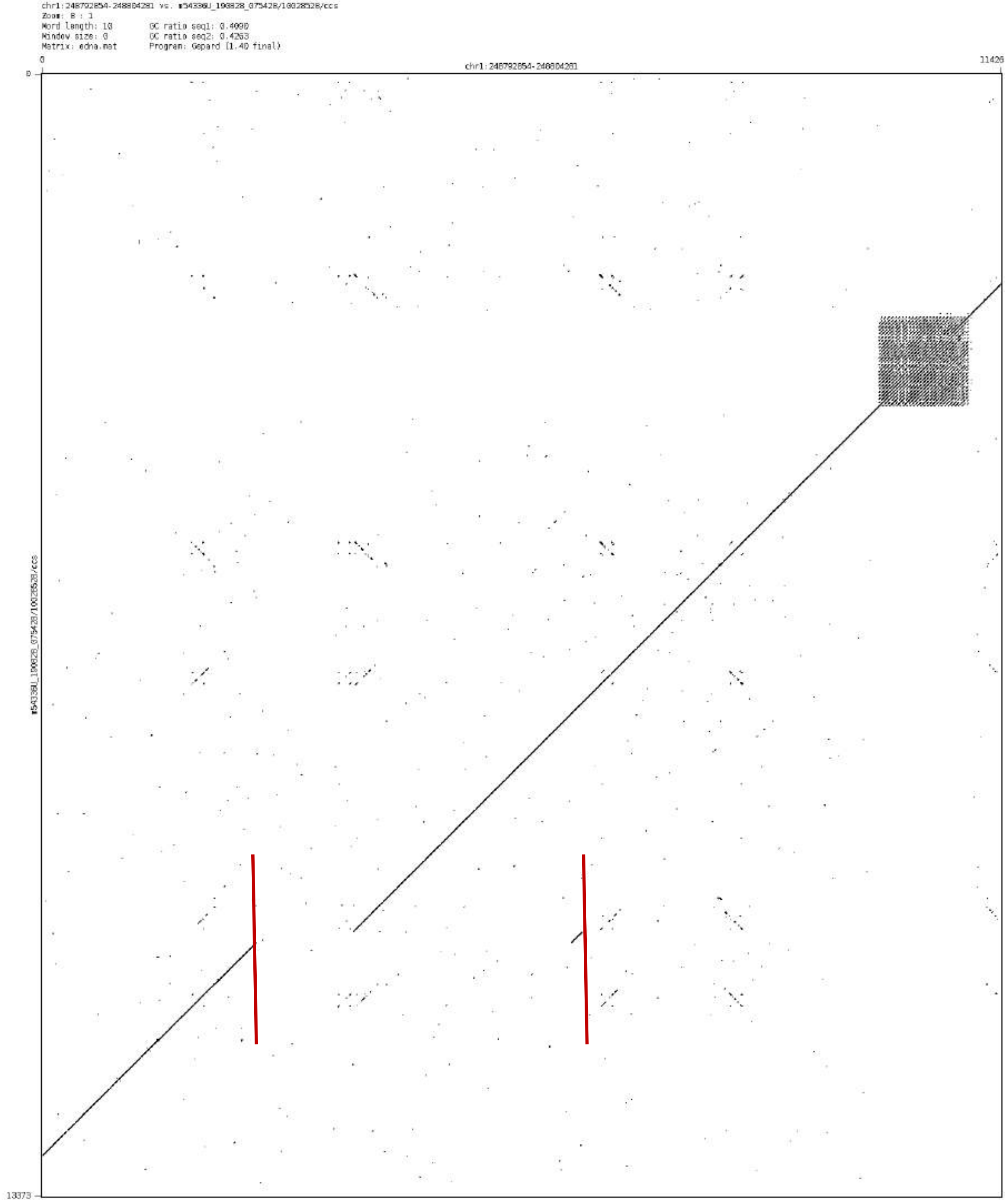
chr1:248792854-248804281 vs. m54336U_190828_075428/10028528/ccs
Zoom: 8 : 1
Word length: 10
Window size: 0
Matrix: edna.mat
GC ratio seq1: 0.4090
GC ratio seq2: 0.4263
Program: Gepard (1.40 final)
0
chr1:248792854-248804281
11426
0
13373
m54336U_190828_075428/10028528/ccs

chr2:16225125-16226710

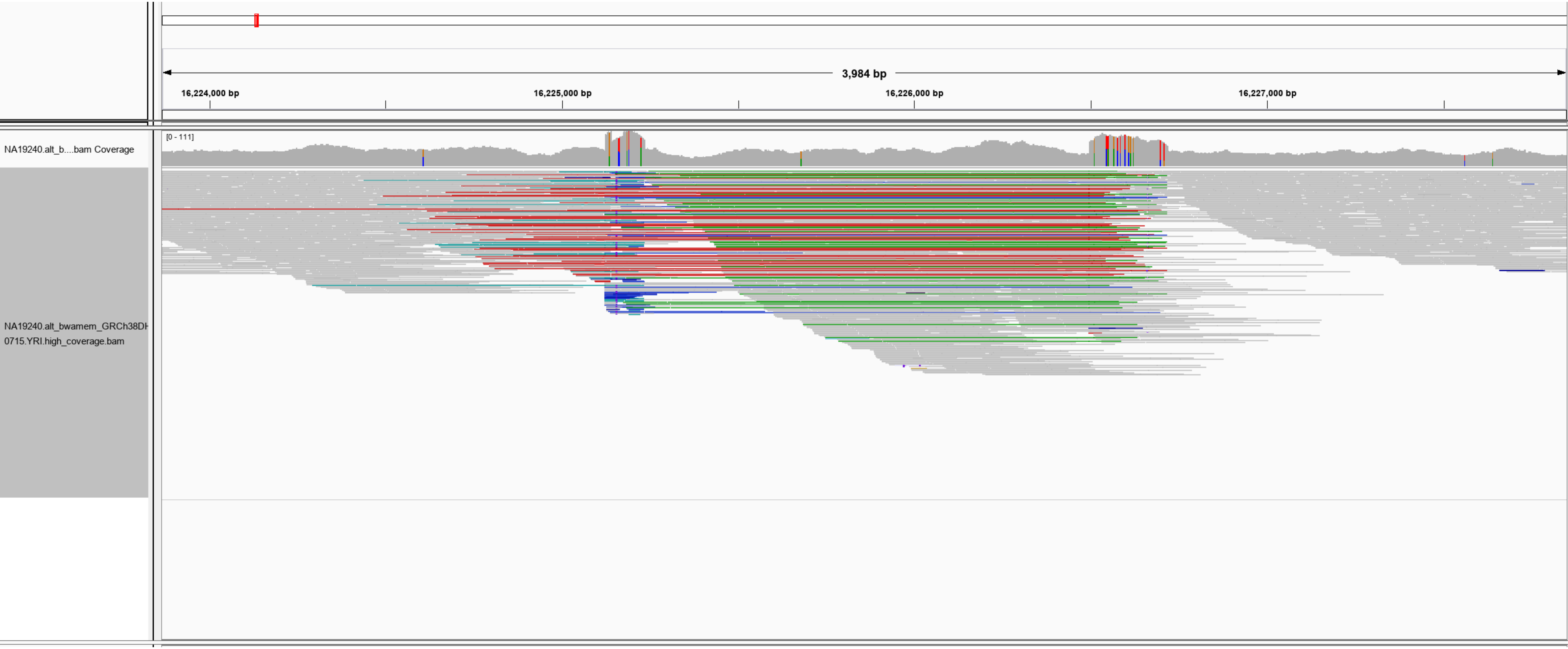

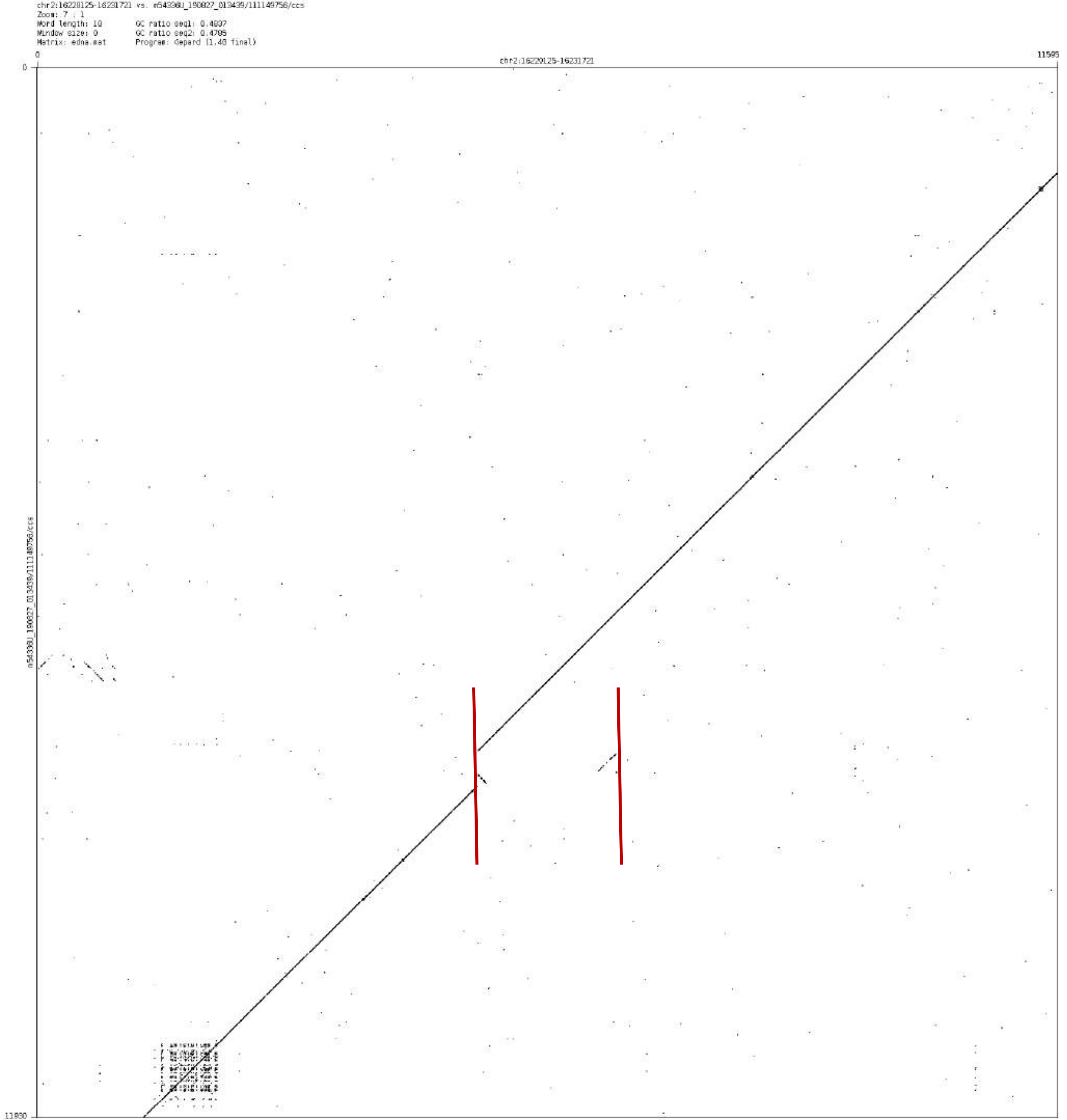
chr2:16228125-16231721 vs. m54330U_190827_013439/111149756/ccs
Zoom: 7 : 1
Word length: 10
Window size: 0
Matrix: edna.mat
GC ratio seq1: 0.4837
GC ratio seq2: 0.4785
Program: depard (1.40 final)
0
11595
chr2:16228125-16231721
0
11930

chr2:50538785-50539113

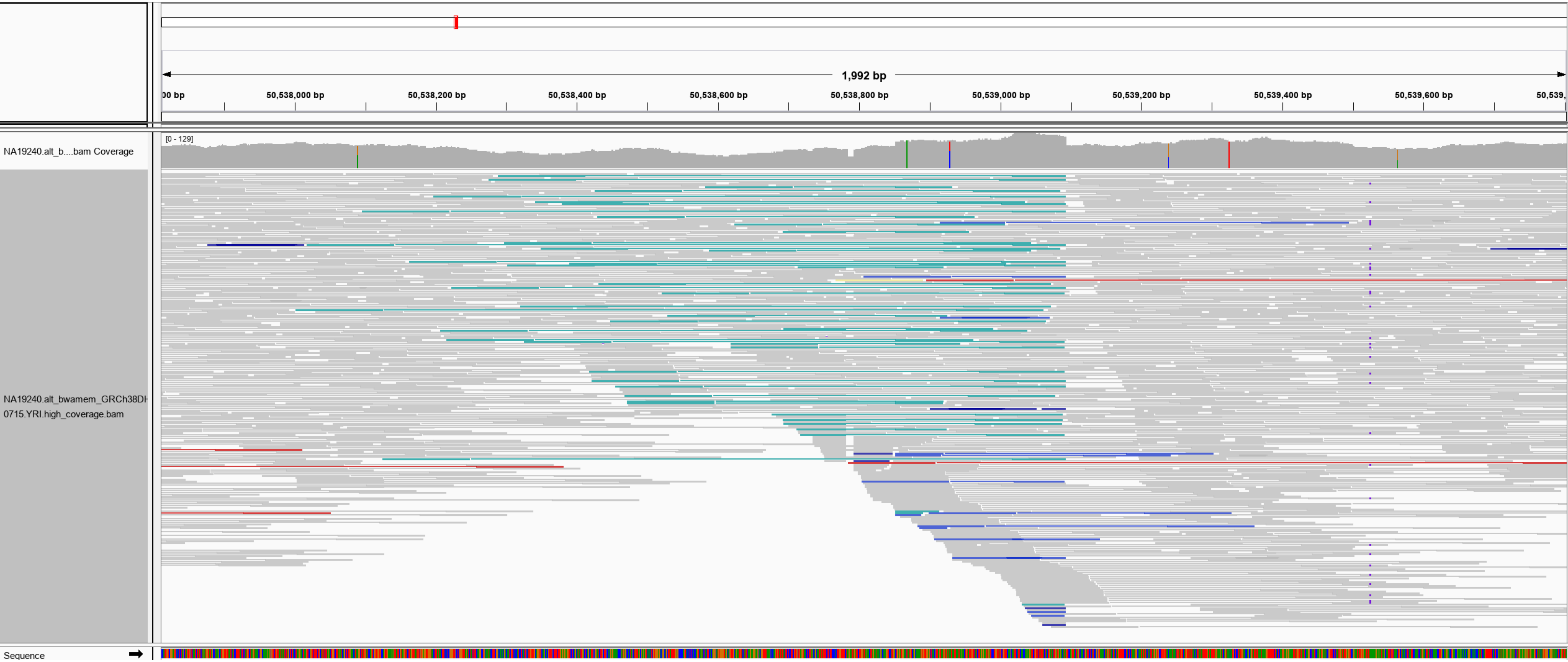

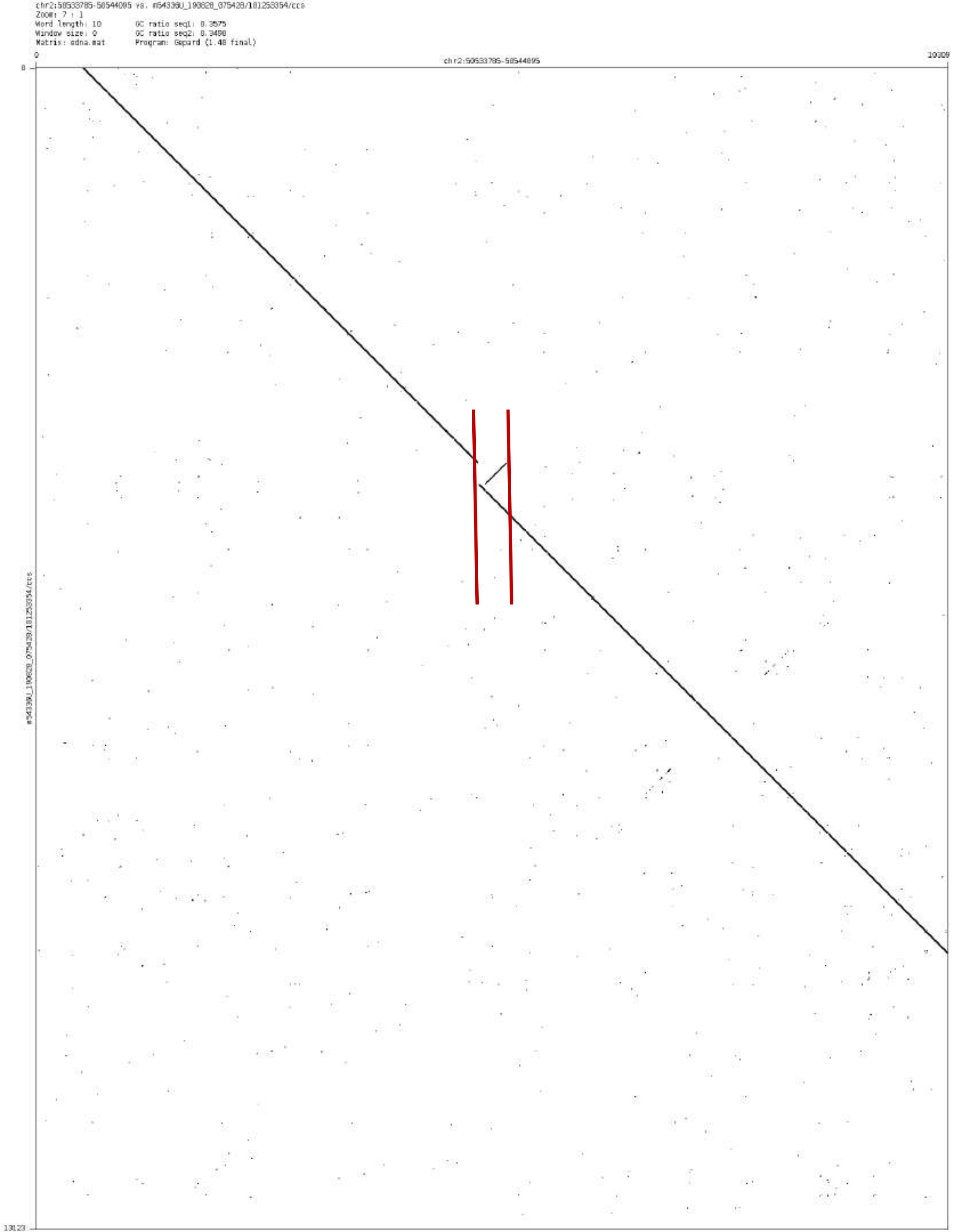
chr2:58533785-58544085 vs. m54336U_190828_075428/181253354/ccs
Zoom: 7 : 1
Word length: 10
Window size: 0
Matrix: edna.mat
GC ratio seq1: 0.3575
GC ratio seq2: 0.3490
Program: Gepard (1.40 final)
chr2:58533785-58544085
13123

chr2:124294182-124295678

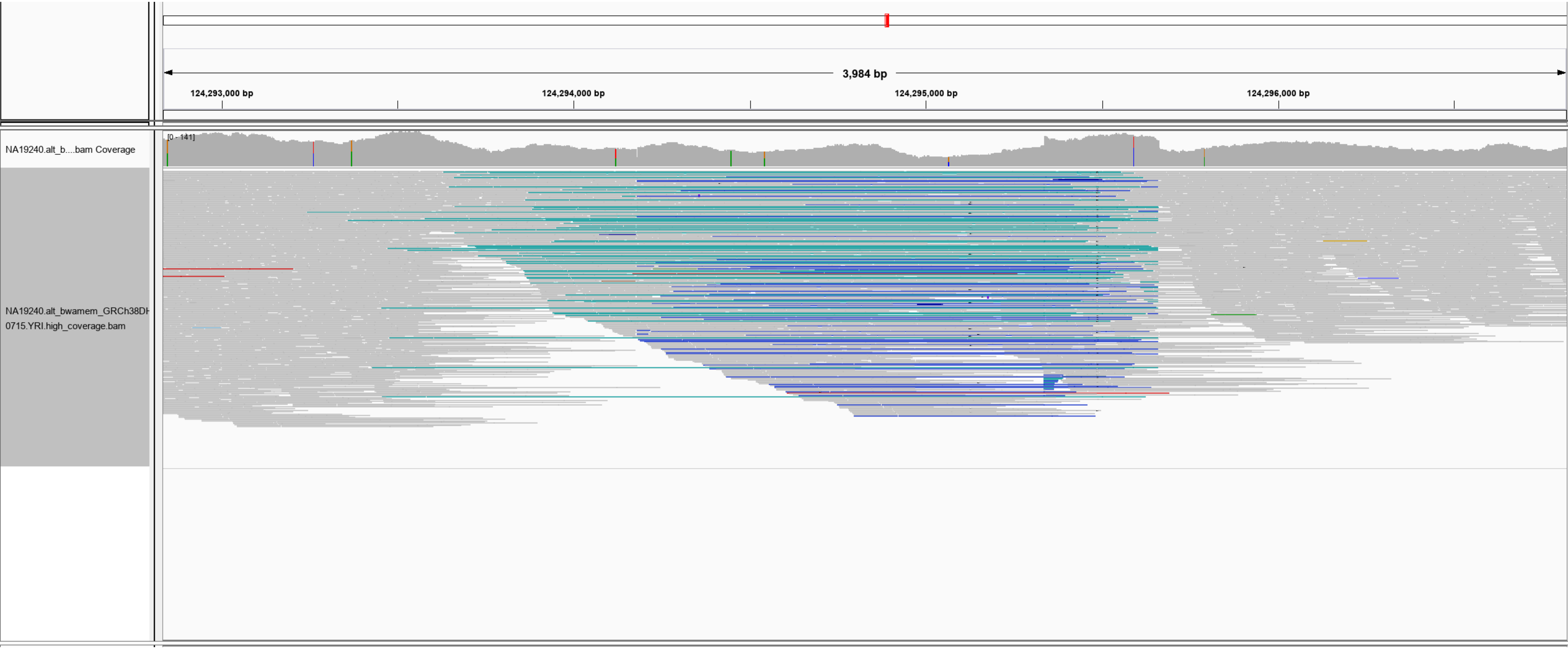

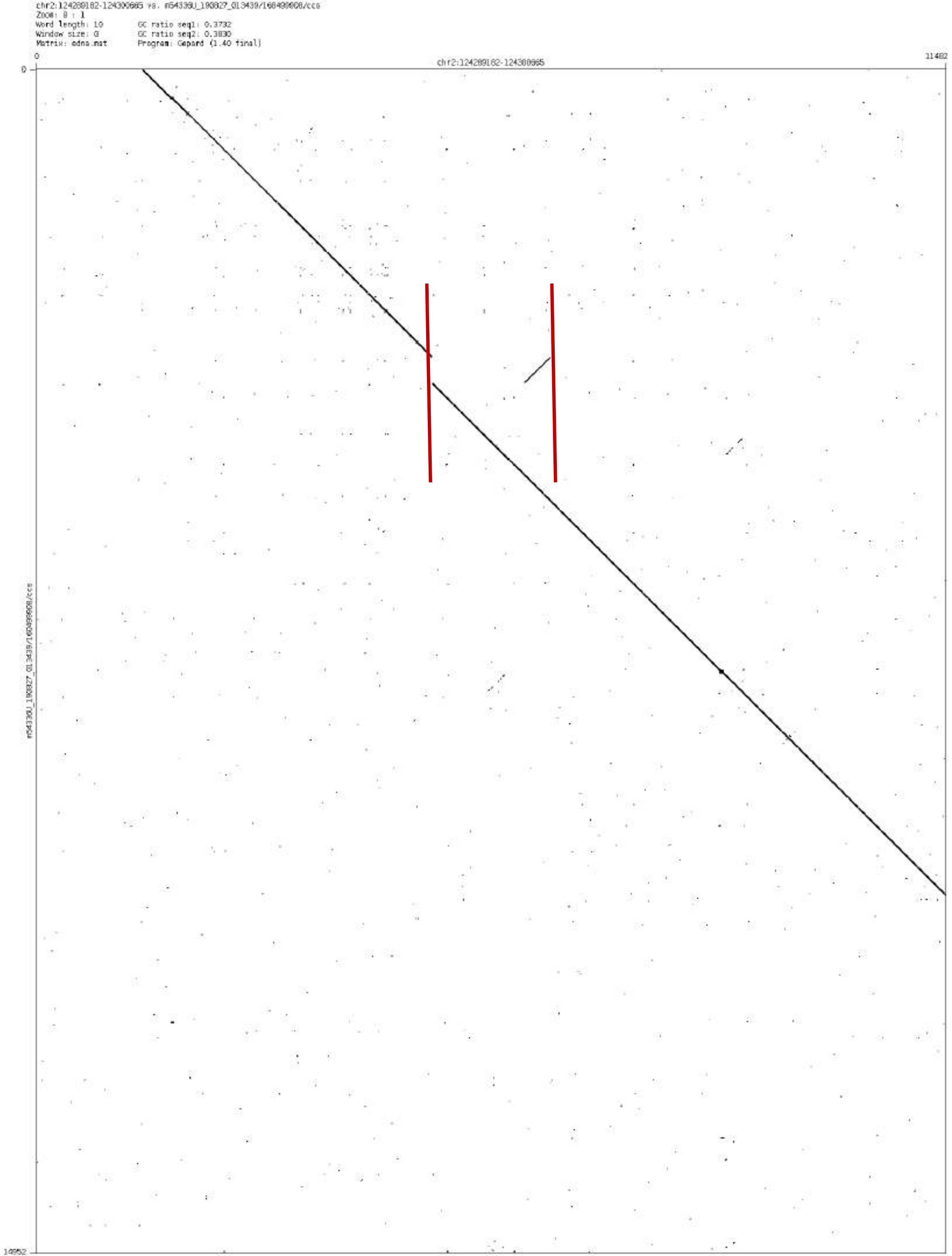
chr2:124289182-124300665 vs. m54336U_190827_013439/160499808/ccs
Zoom: 8 : 1
Word length: 10
Window size: 0
Matrix: edna.mat
GC ratio seq1: 0.3732
GC ratio seq2: 0.3830
Program: Gepard (1.40 final)
0
11482
chr2:124289182-124300665
14952

chr2: 129791792-129801213

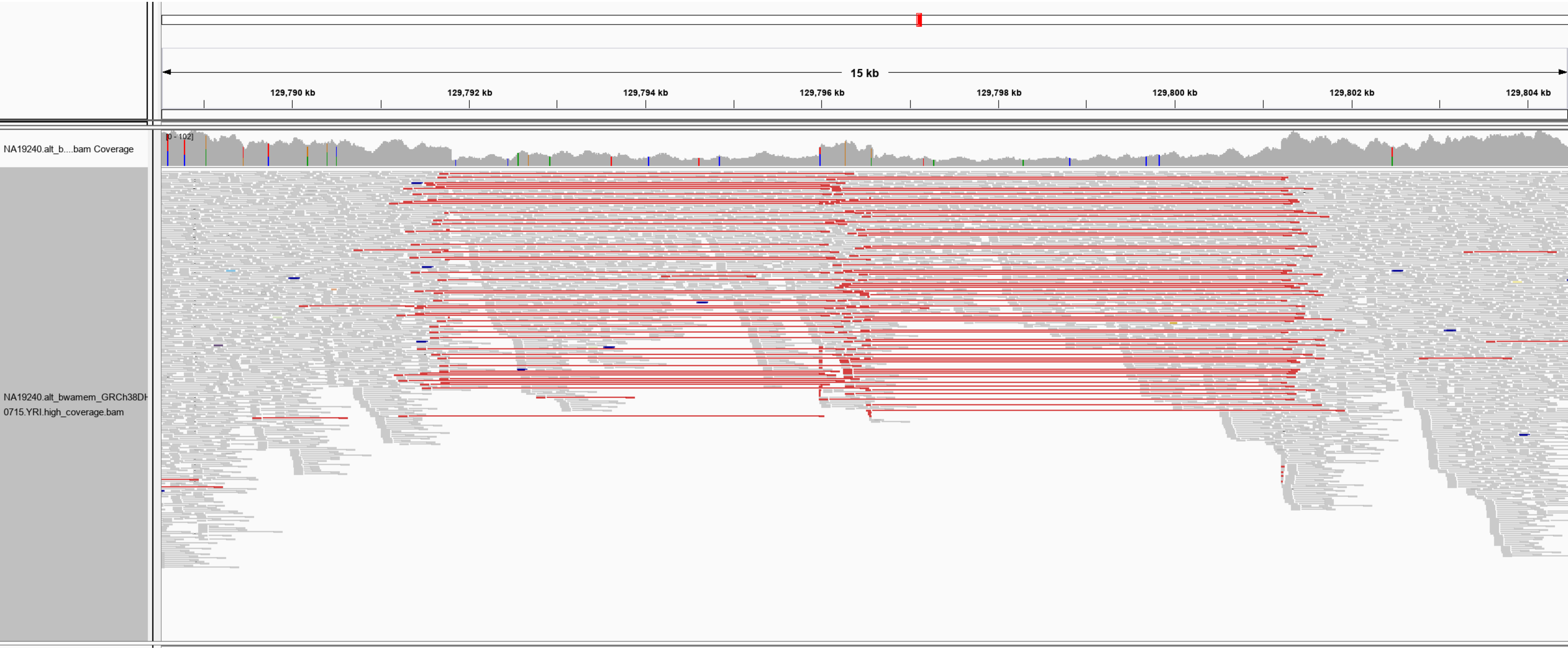

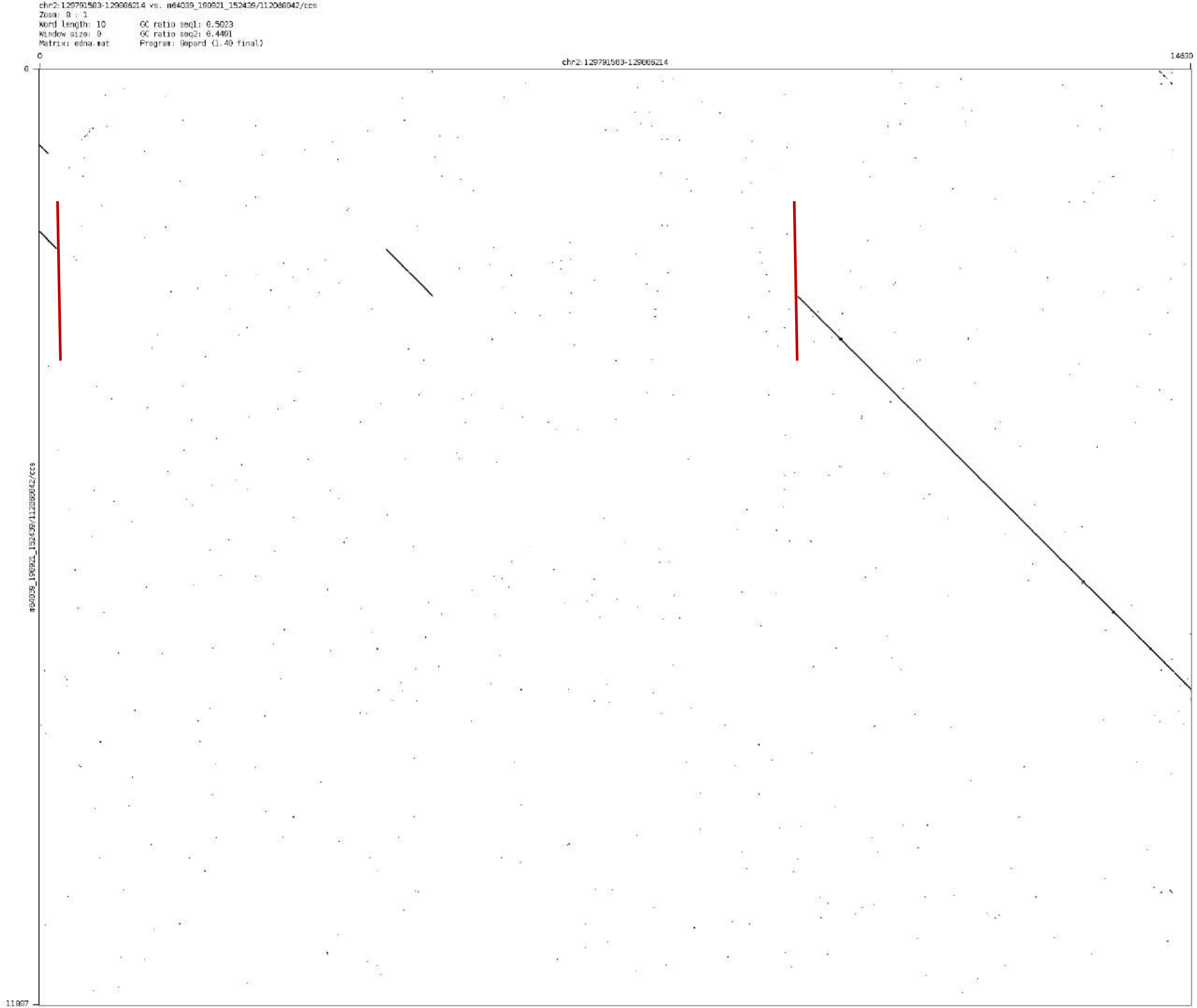

chr2:129791583-129806214 vs. m64039_190921_152439/112068042/ccs
Zoom: 8 : 1
Word length: 10
GC ratio seq1: 0.5023
Window size: 0
GC ratio seq2: 0.4491
Matrix: edna.mat
Program: Gepard (1.40 final)
0
14630
chr2:129791583-129806214
0
m64039_190921_152439/112068042/ccs
11887

chr2:143314745-143316563

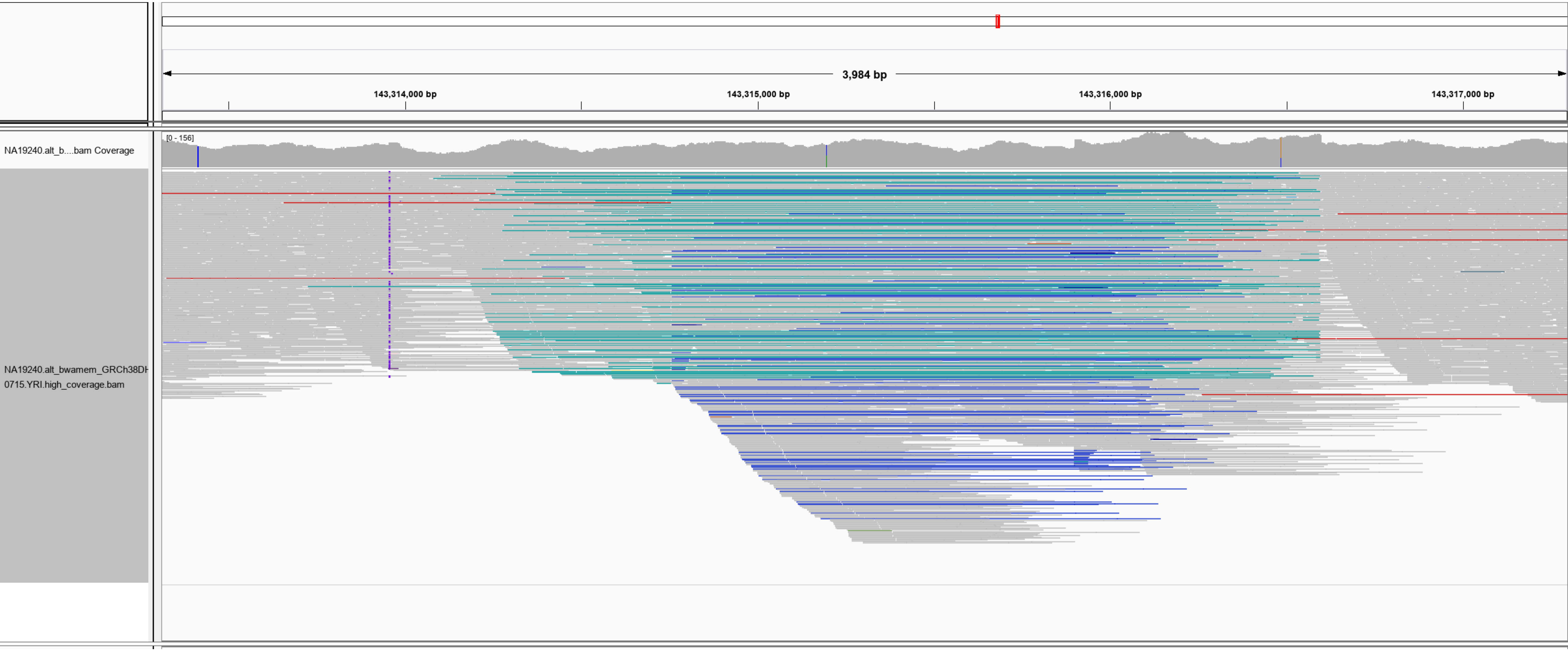

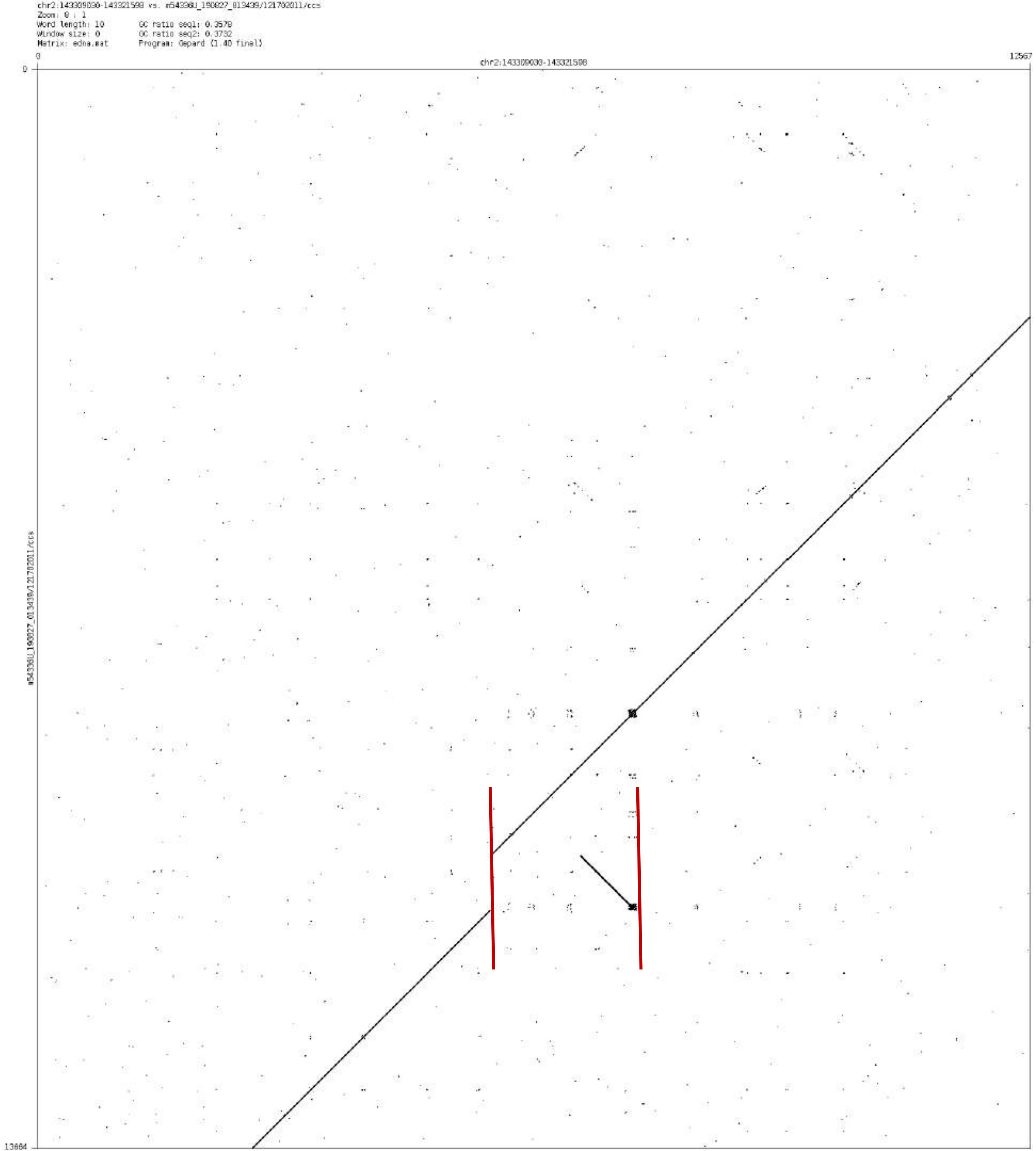
chr2:143309030-143321598 vs. m54398U_190827_013439/121702011/ccs
Zoom: 0 : 1
Word length: 10
Window size: 0
Matrix: edna.mat
GC ratio seq1: 0.3578
GC ratio seq2: 0.3732
Program: Gepard (1.40 final)
0
chr2:143309030-143321598
12567
0
m54398U_190827_013439/121702011/ccs
13664

chr2:156813519-156815219

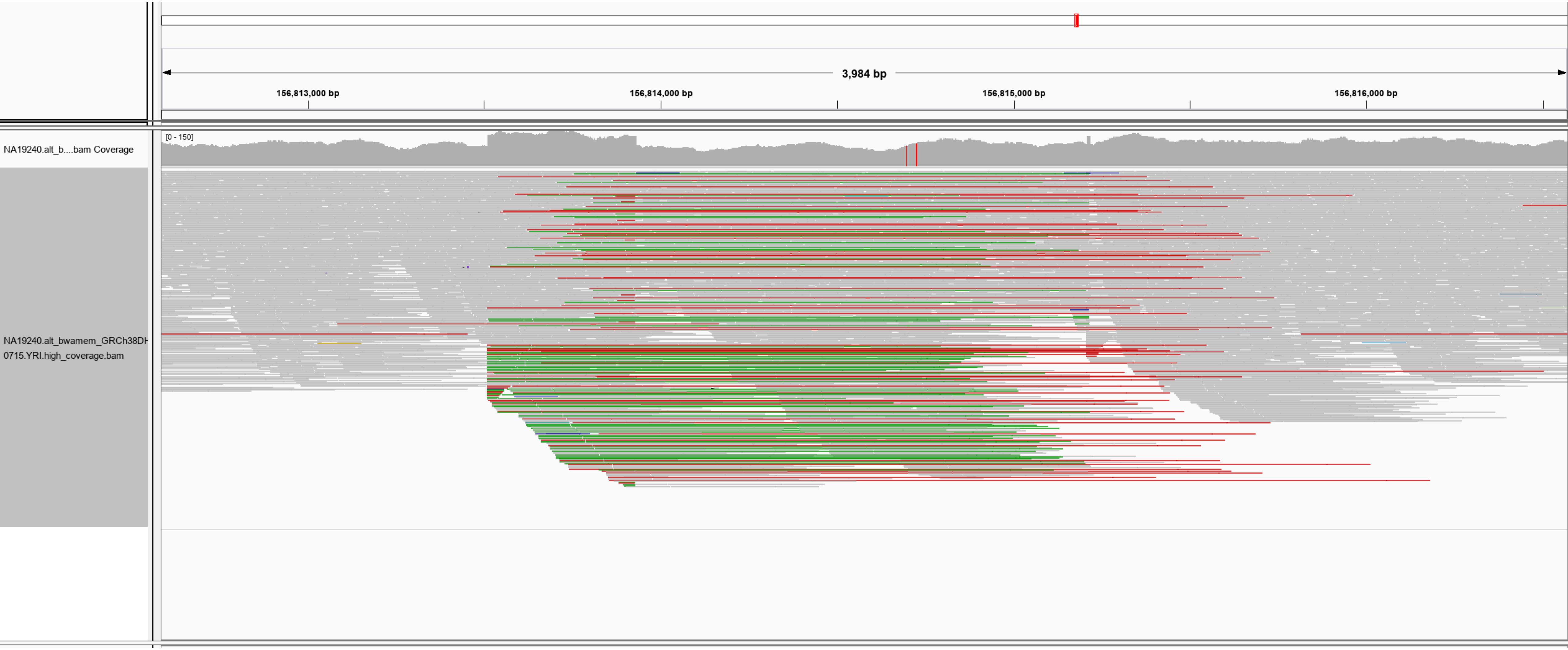

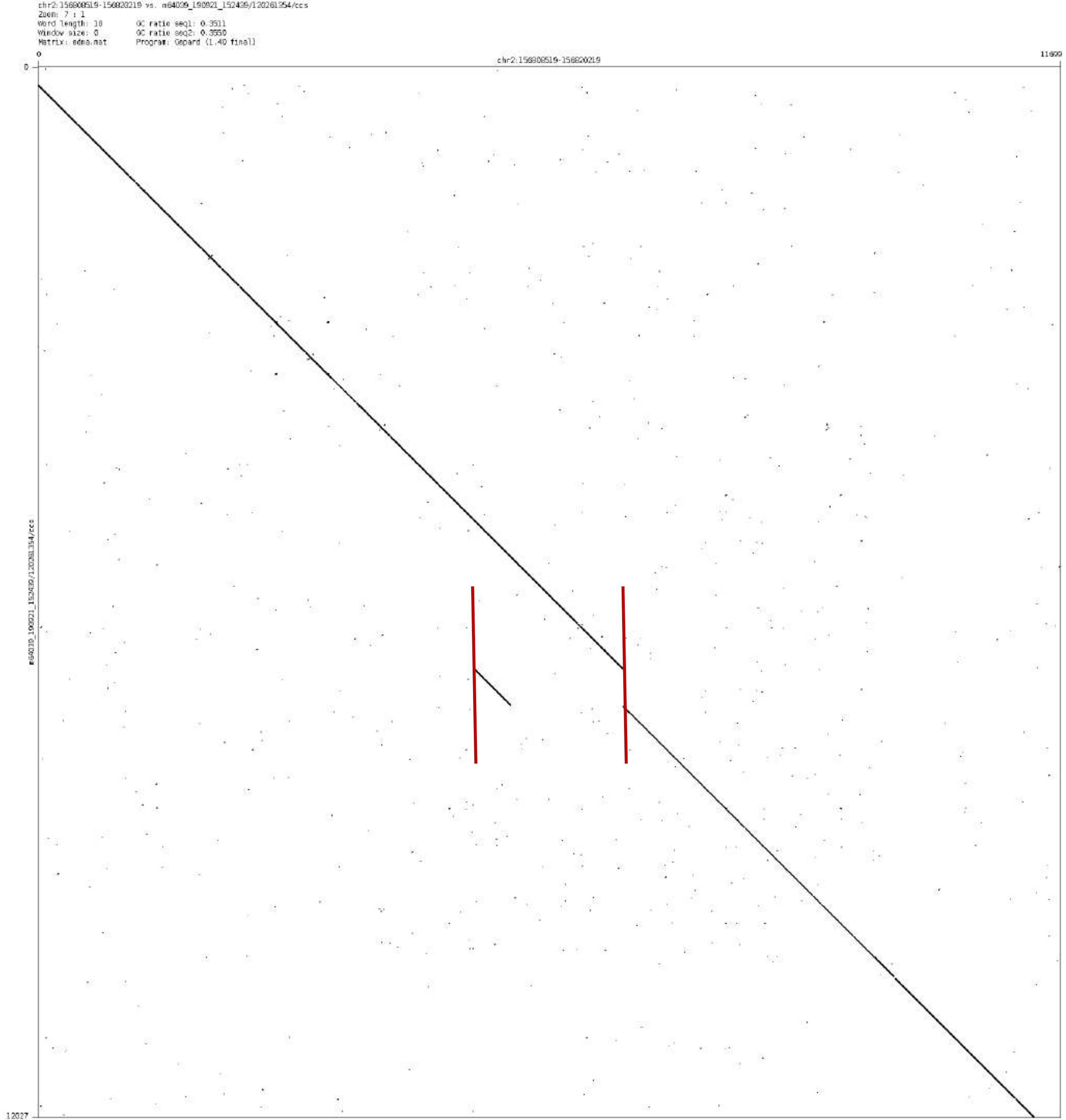
chr2:156808519-156820219 vs. m64039_190921_152439/120261354/ccs
Zoom: 7 : 1
Word length: 10
GC ratio seq1: 0.3511
Window size: 0
GC ratio seq2: 0.3550
Matrix: edna.mat
Program: Gepard (1.40 final)
0
chr2:156808519-156820219
11699
0
m64039_190921_152439/120261354/ccs
12027

chr2:216225220-216228380

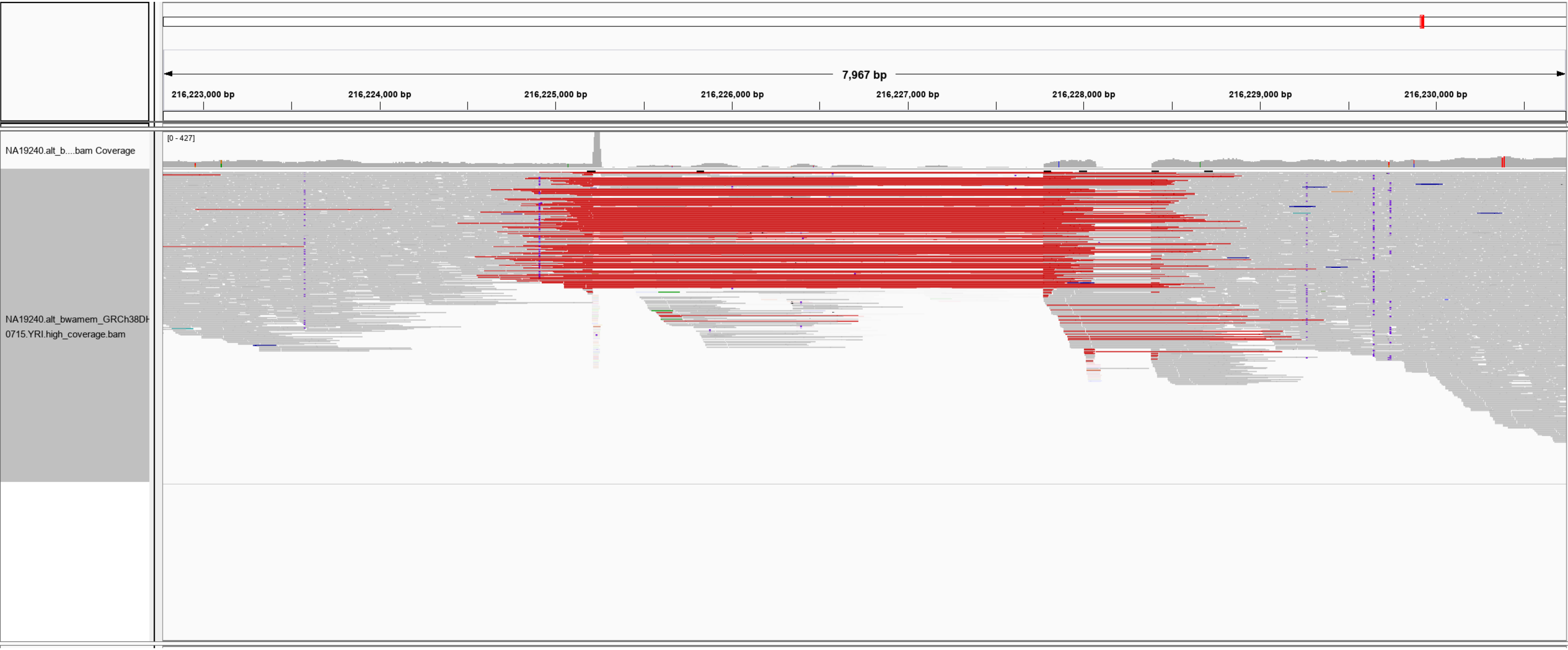

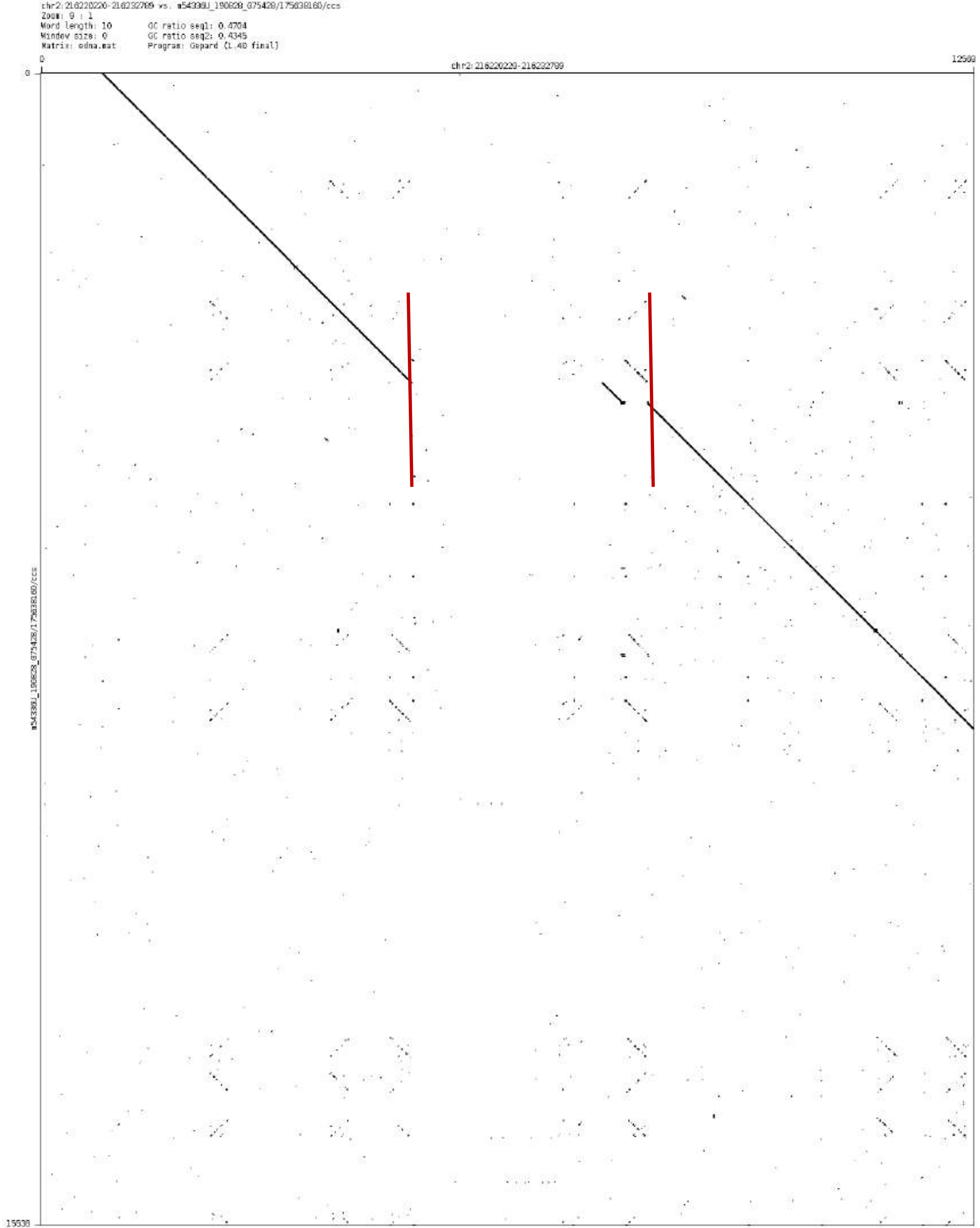
chr2:218220226-218232789 vs. m54338U_190828_075428/175838160/ccs
Zoom: 9 : 1
Word length: 10
Window size: 0
Matrix: edna.mat
GC ratio seq1: 0.4704
GC ratio seq2: 0.4345
Program: Gepard (1.40 final)
0
chr2:218220226-218232789
12569
0
m54338U_190828_075428/175838160/ccs
15538

chr3:95746925-95752145

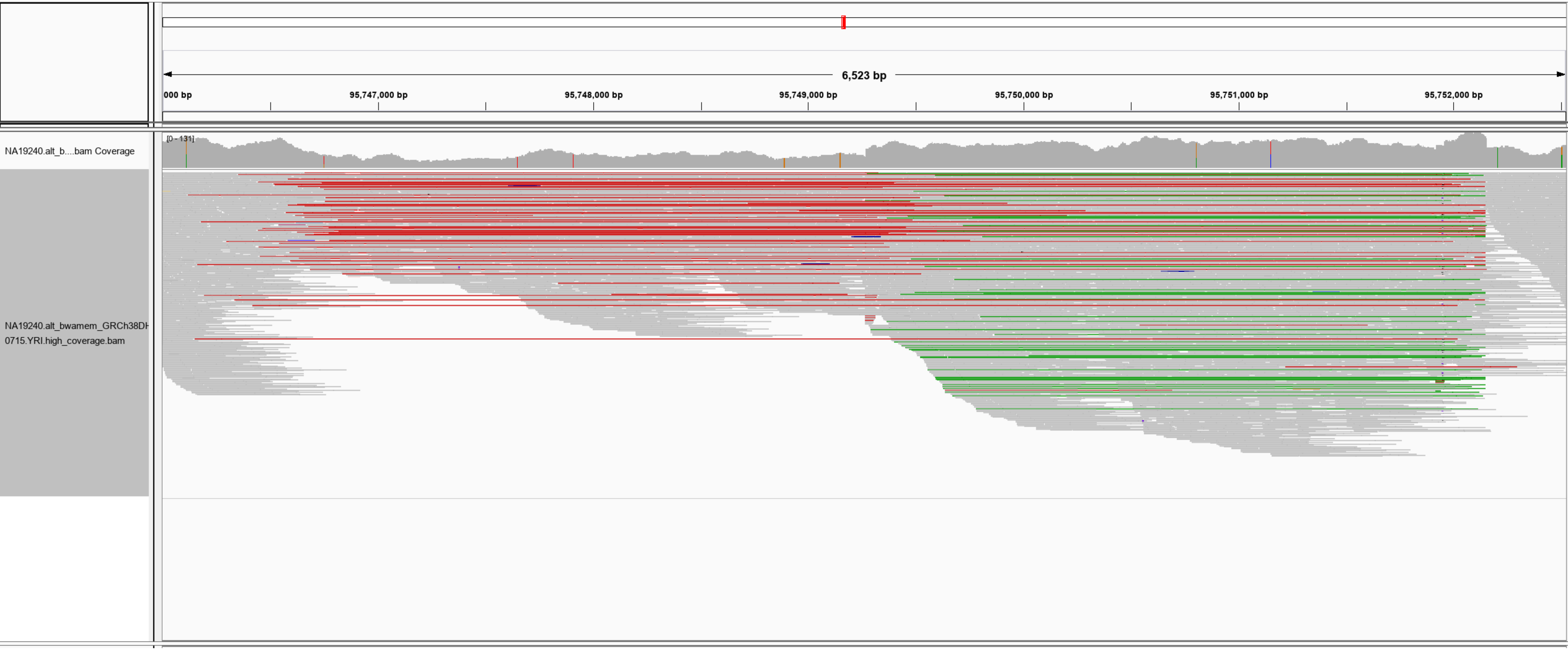

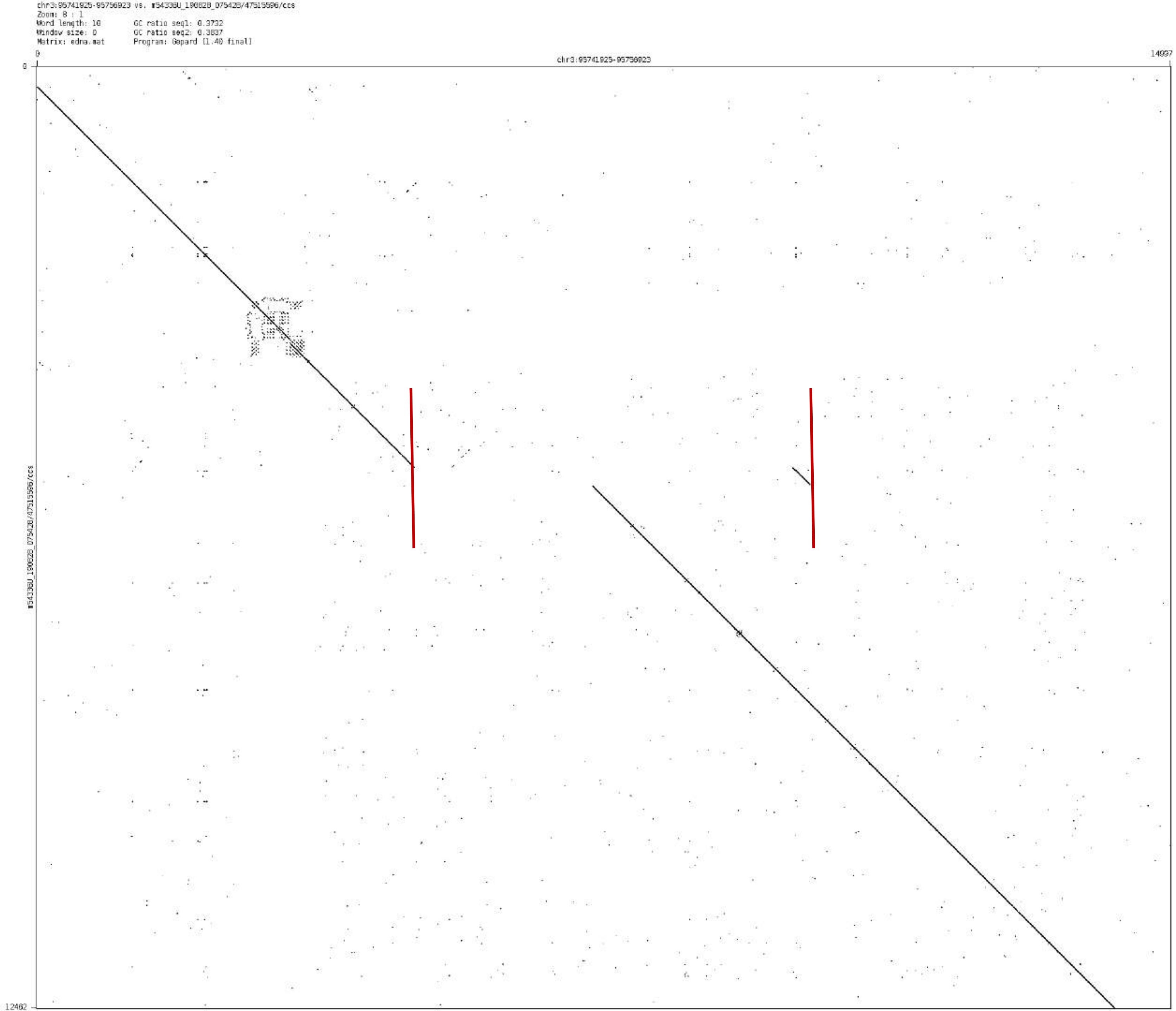
chr3:95741925-95756923 vs. m54336U_190828_075428/47515596/ccs
Zoom: 8 : 1
Word length: 10
Window size: 0
Matrix: edna.mat
GC ratio seq1: 0.3732
GC ratio seq2: 0.3837
Program: Gepard [1.40 final]
0
14997
chr3:95741925-95756923
0
12462
m54336U_190828_075428/47515596/ccs

chr3:146667406-146677351

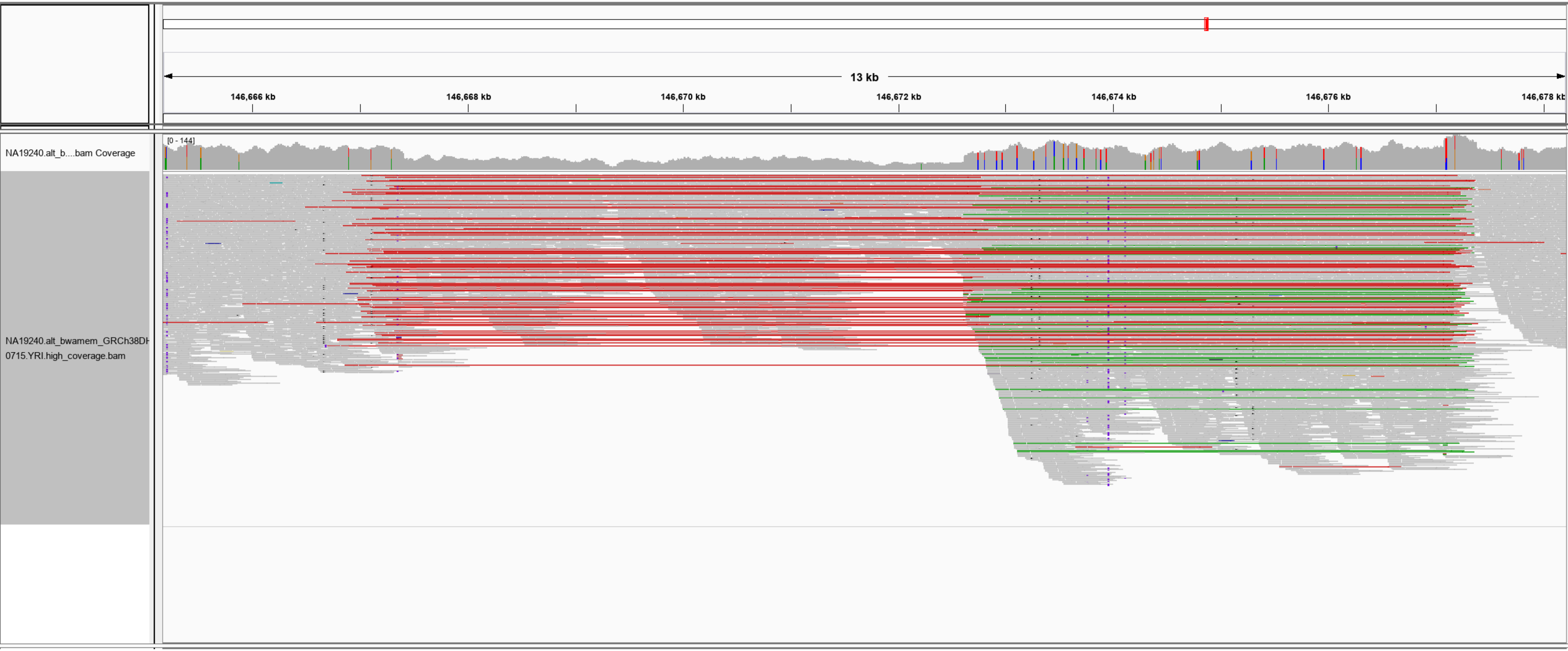

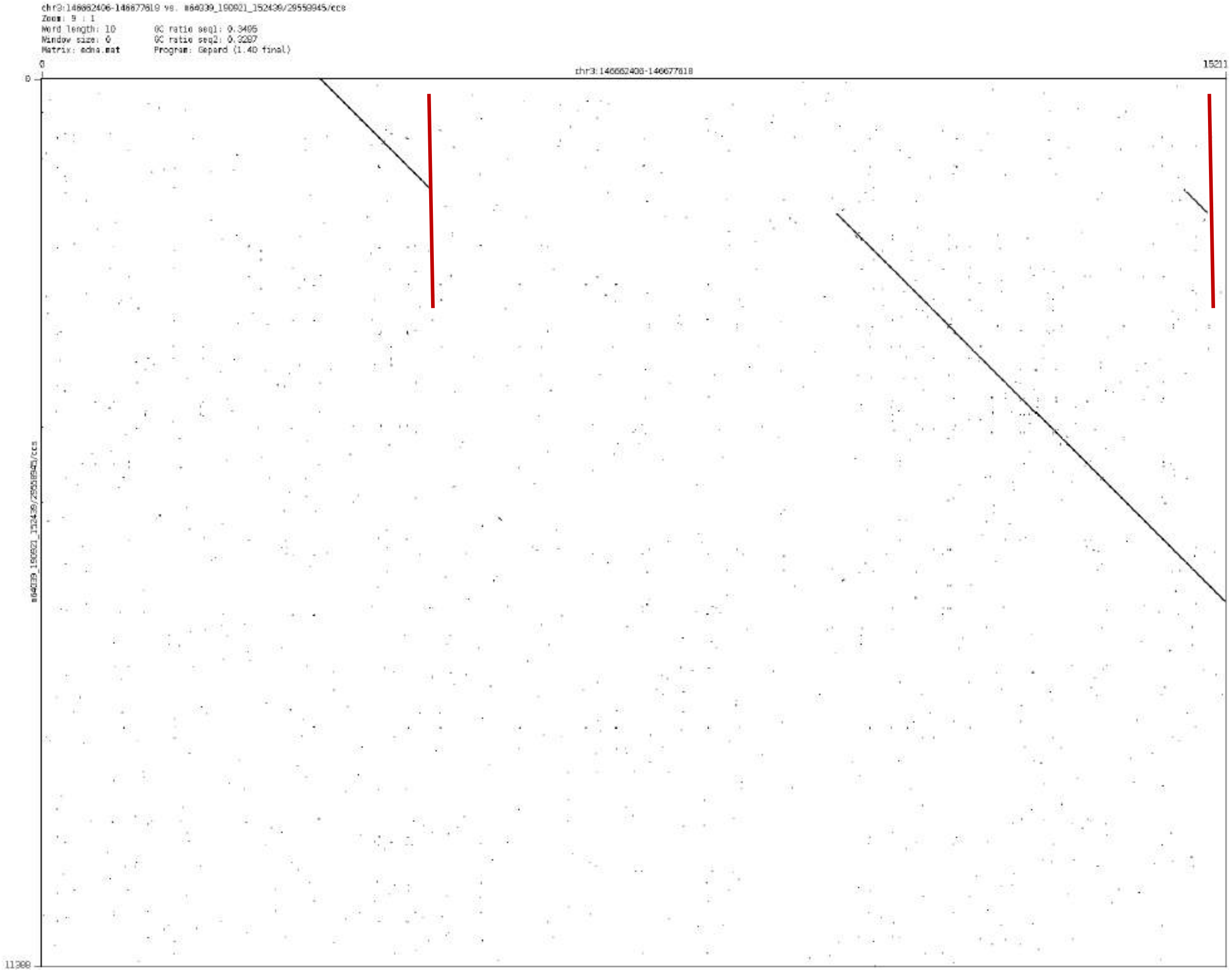

chr3:146662406-146677619 vs. m64039_190921_152439/29559945/ccs
Zoom: 9 : 1
Word length: 10
Window size: 0
Matrix: edna.mat
GC ratio seq1: 0.3495
GC ratio seq2: 0.3287
Program: Gepard (1.40 final)
0
15211
chr3:146662406-146677619
0
11388
m64039_190921_152439/29559945/ccs

chr4: 92646201-92649031

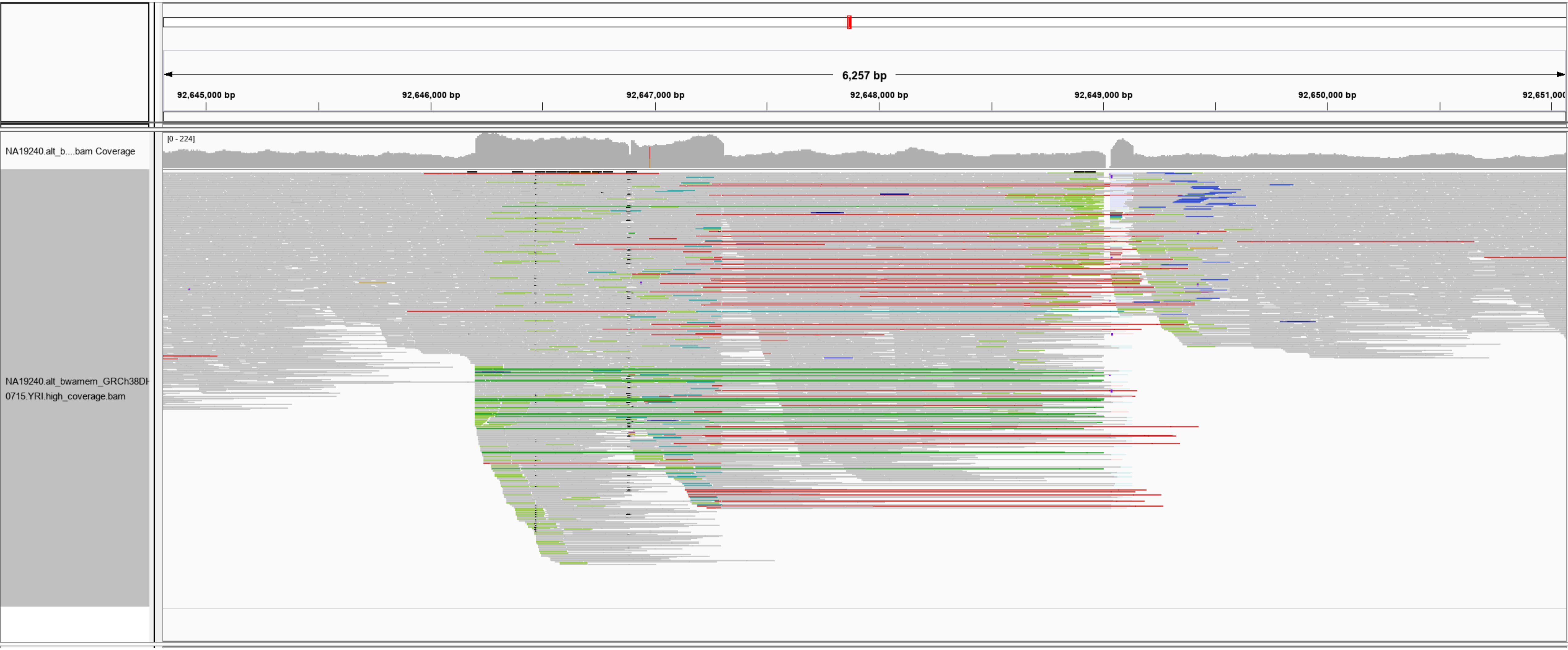

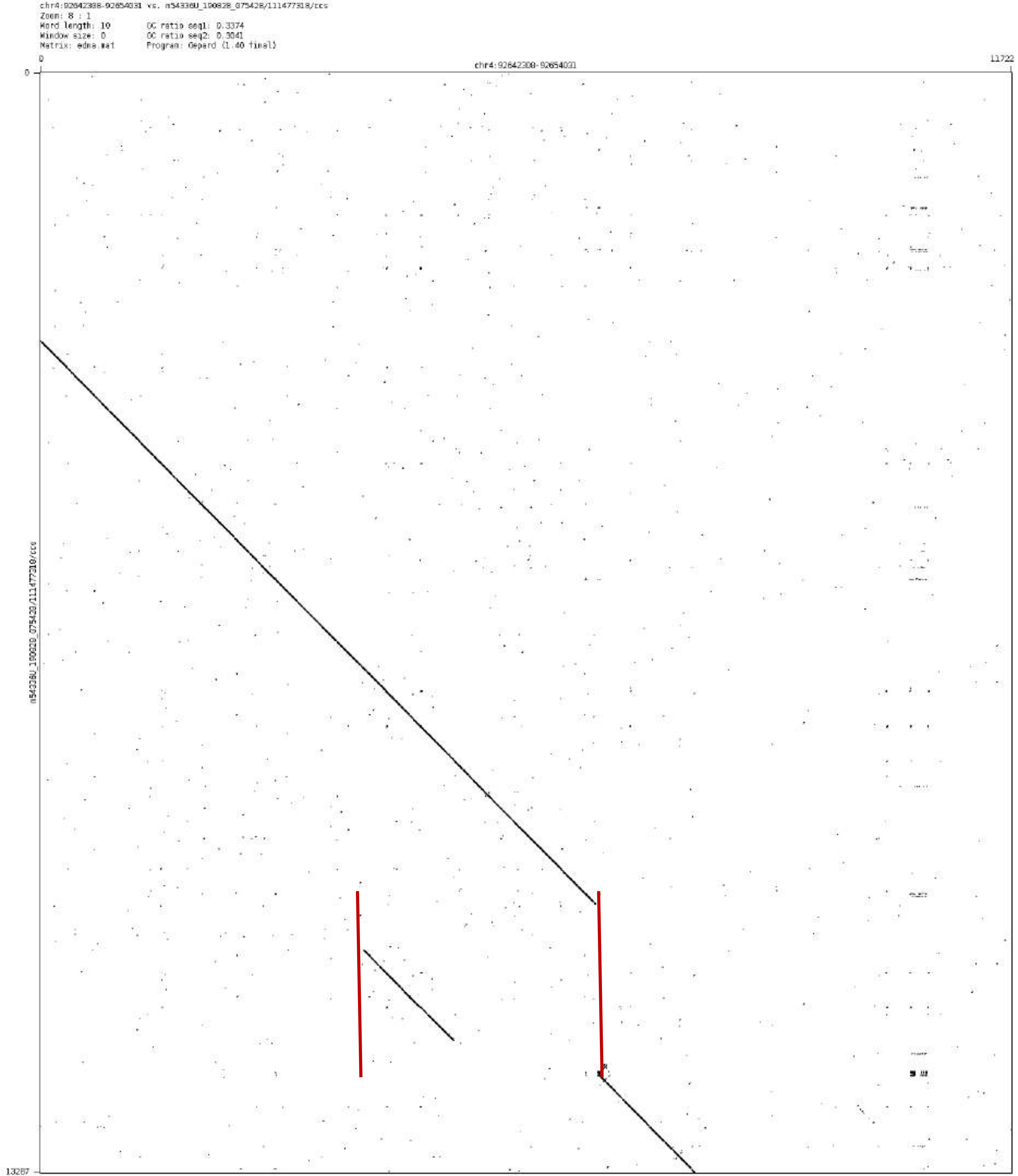

chr4:92642388-92654031 vs. m54336U_190828_075428/111477318/ccs
Zoom: 8 : 1
Word length: 10
Window size: 0
Matrix: edna.mat
GC ratio seq1: 0.3374
GC ratio seq2: 0.3041
Program: Gepard (1.40 final)
0
chr4:92642388-92654031
11722
0
13287

chr5:25821671-25826900

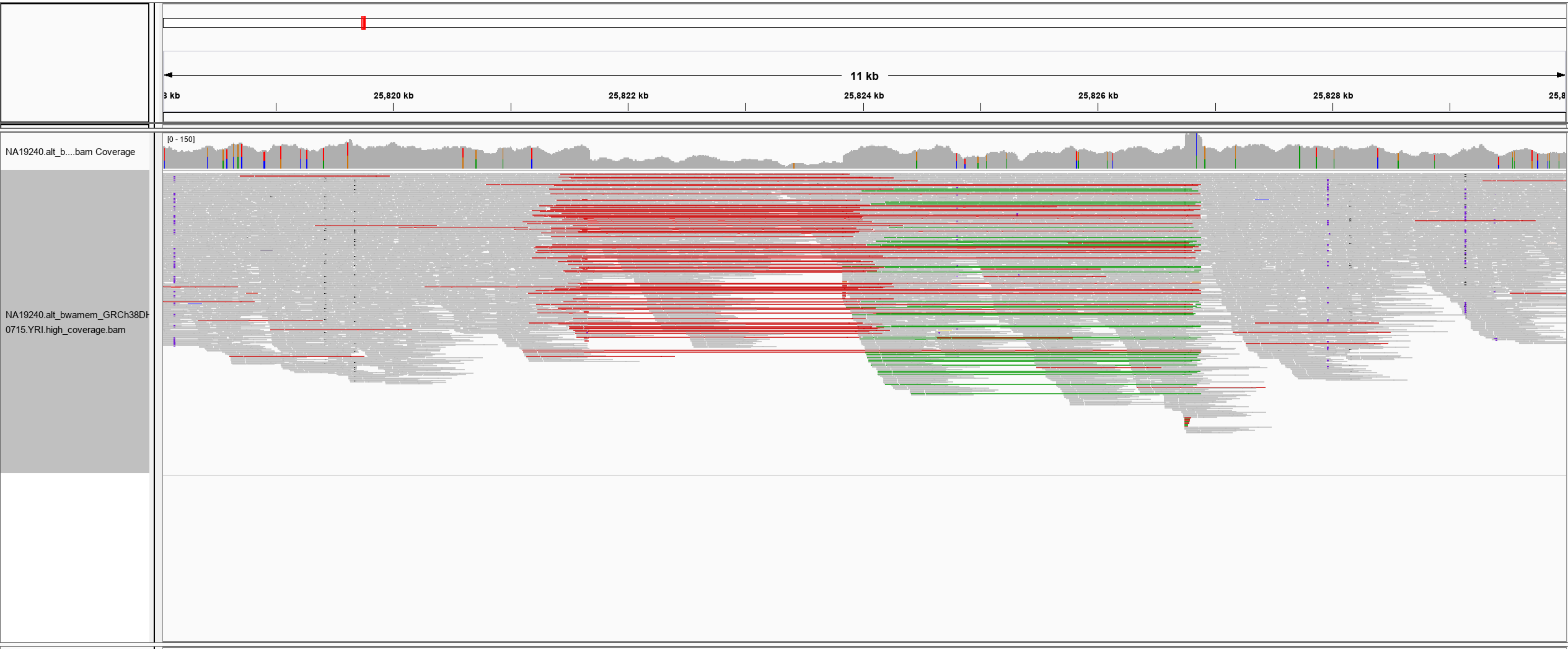

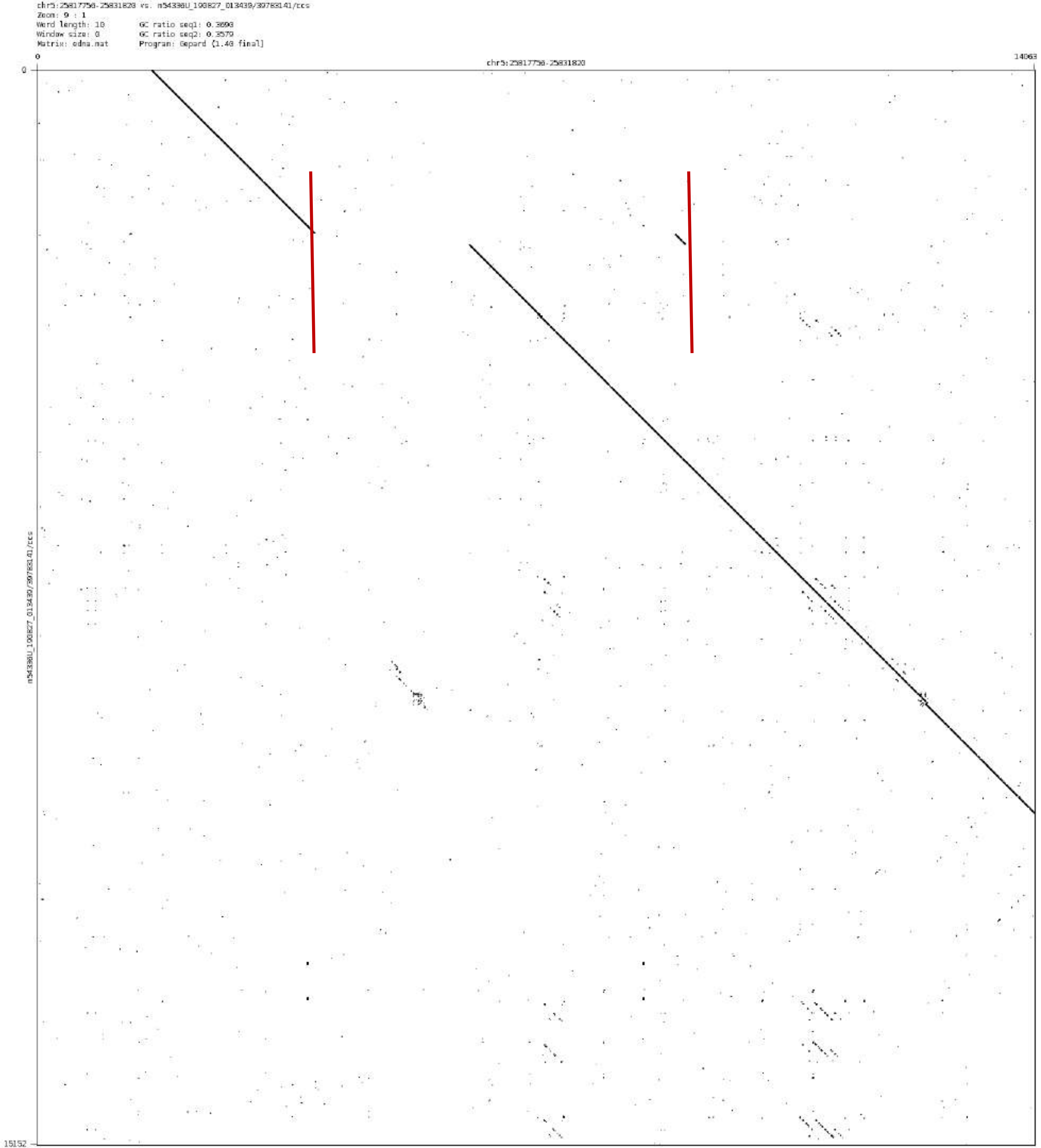
chr5:25817756-25831820 vs. m54336U_190827_013439/39783141/ccs
Zoom: 9 : 1
Word length: 10
Window size: 0
Matrix: edna.mat
GC ratio seq1: 0.3693
GC ratio seq2: 0.3579
Program: Gepard (1.40 final)
0
chr5:25817756-25831820
14063
0
15152
m54336U_190827_013439/39783141/ccs

chr5:114919235-114925727

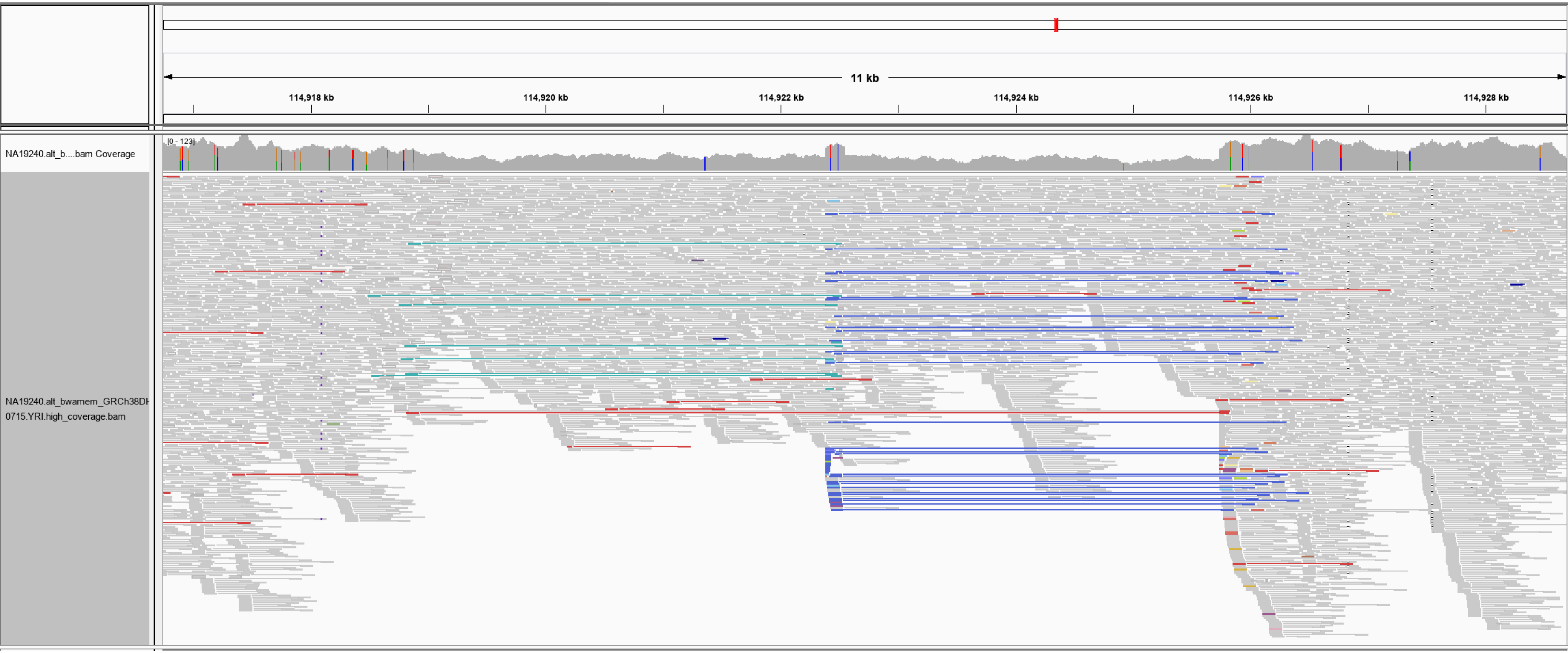

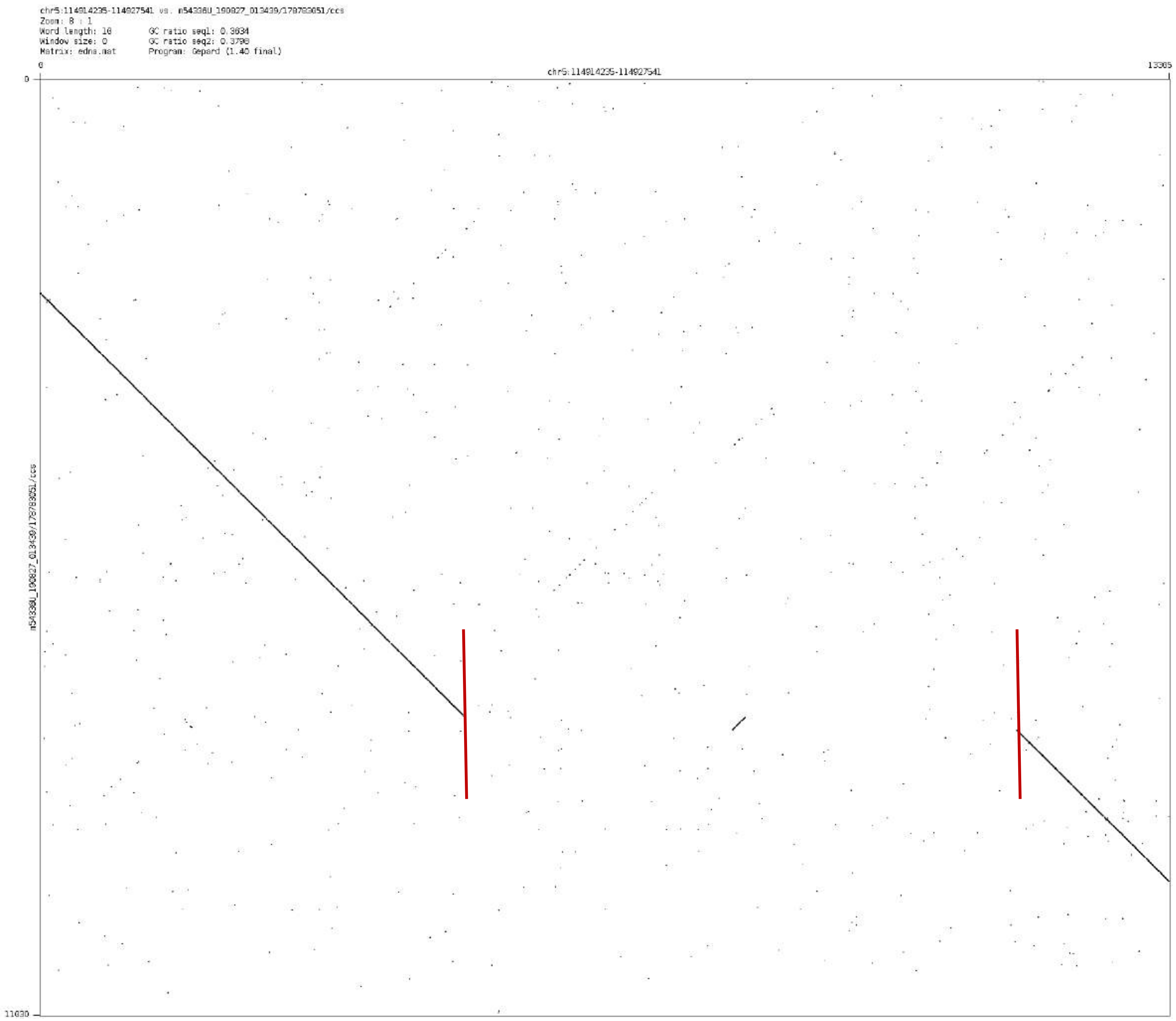
chr5:114914235-114927541 vs. m54336U_190827_013439/178783051/ccs
Zoom: 8 : 1
Word length: 10
GC ratio seq1: 0.3634
Window size: 0
GC ratio seq2: 0.3798
Matrix: edna.mat
Program: Gepard (1.40 final)
0
chr5:114914235-114927541
13305
0
11030
m54336U_190827_013439/178783051/ccs

chr5:116011020-116015362

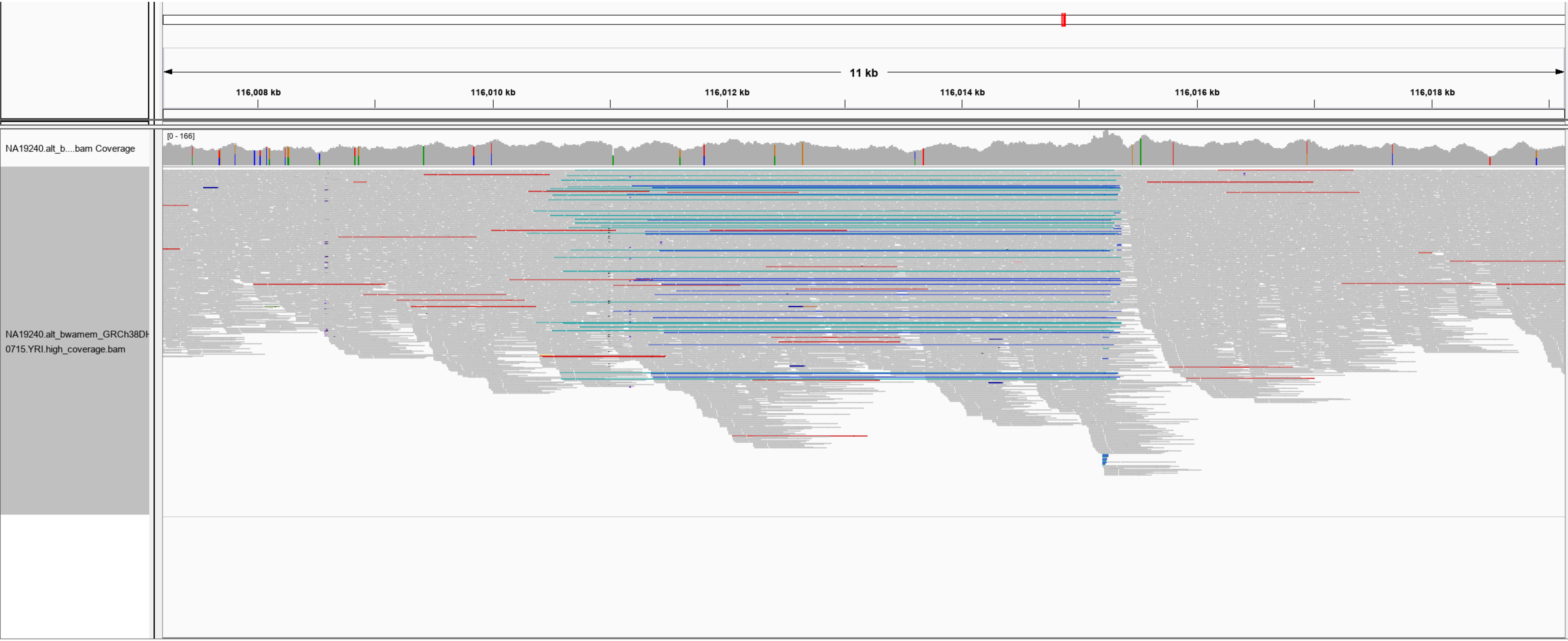

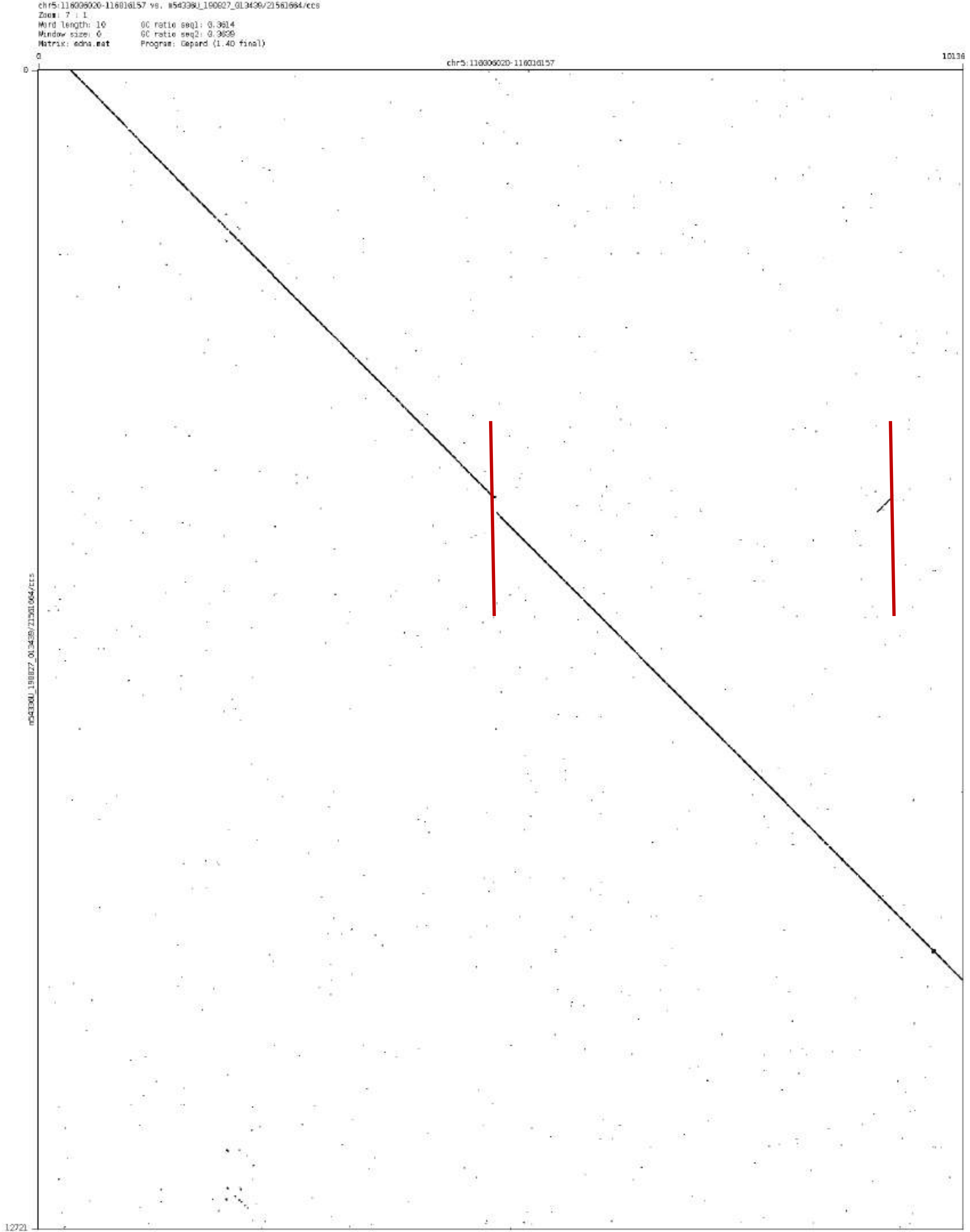
chr5:116006020-116016157 vs. m54336U_190827_013439/21561664/ccs
Zoom: 7 : 1
Word length: 10
Window size: 0
Matrix: edna.mat
GC ratio seq1: 0.3614
GC ratio seq2: 0.3839
Program: Gepard (1.40 final)
0
10138
chr5:116006020-116016157
0
12721
m54336U_190827_013439/21561664/ccs

chr5:144132930-144135491

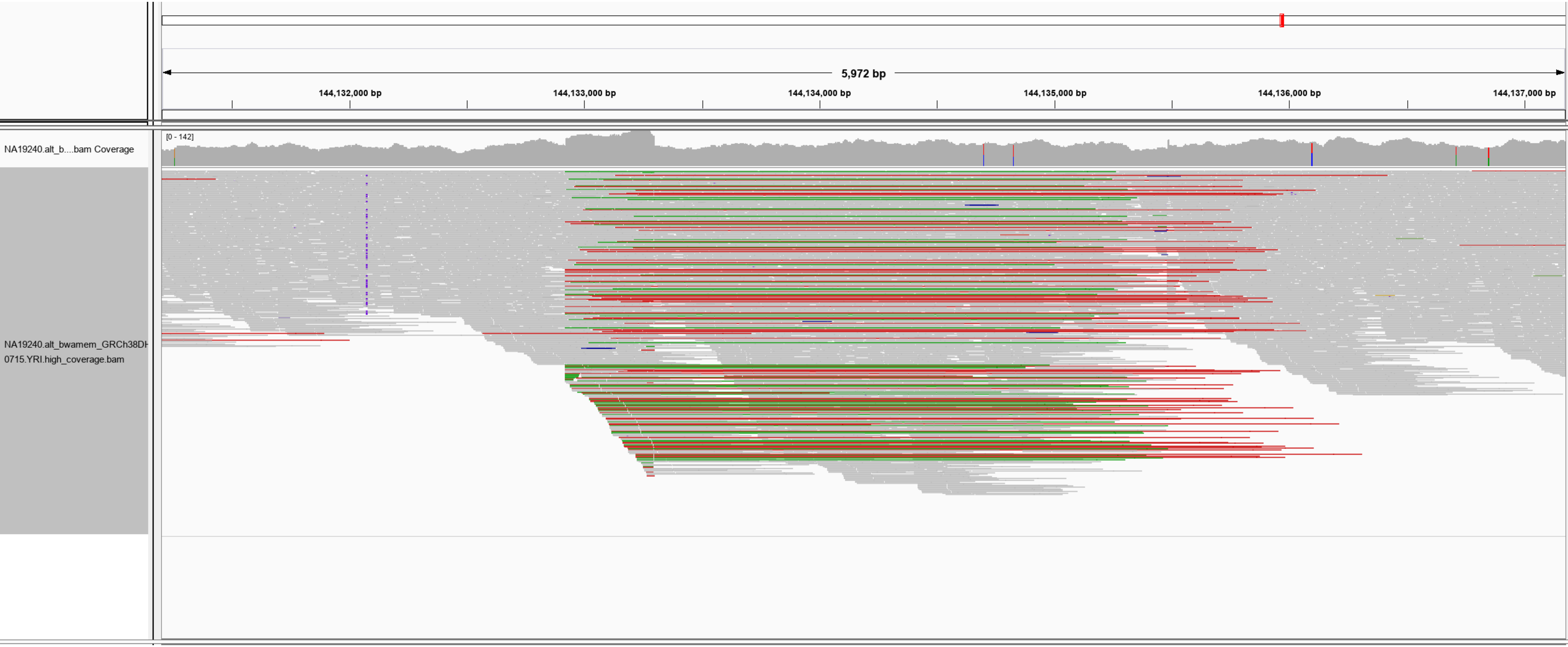

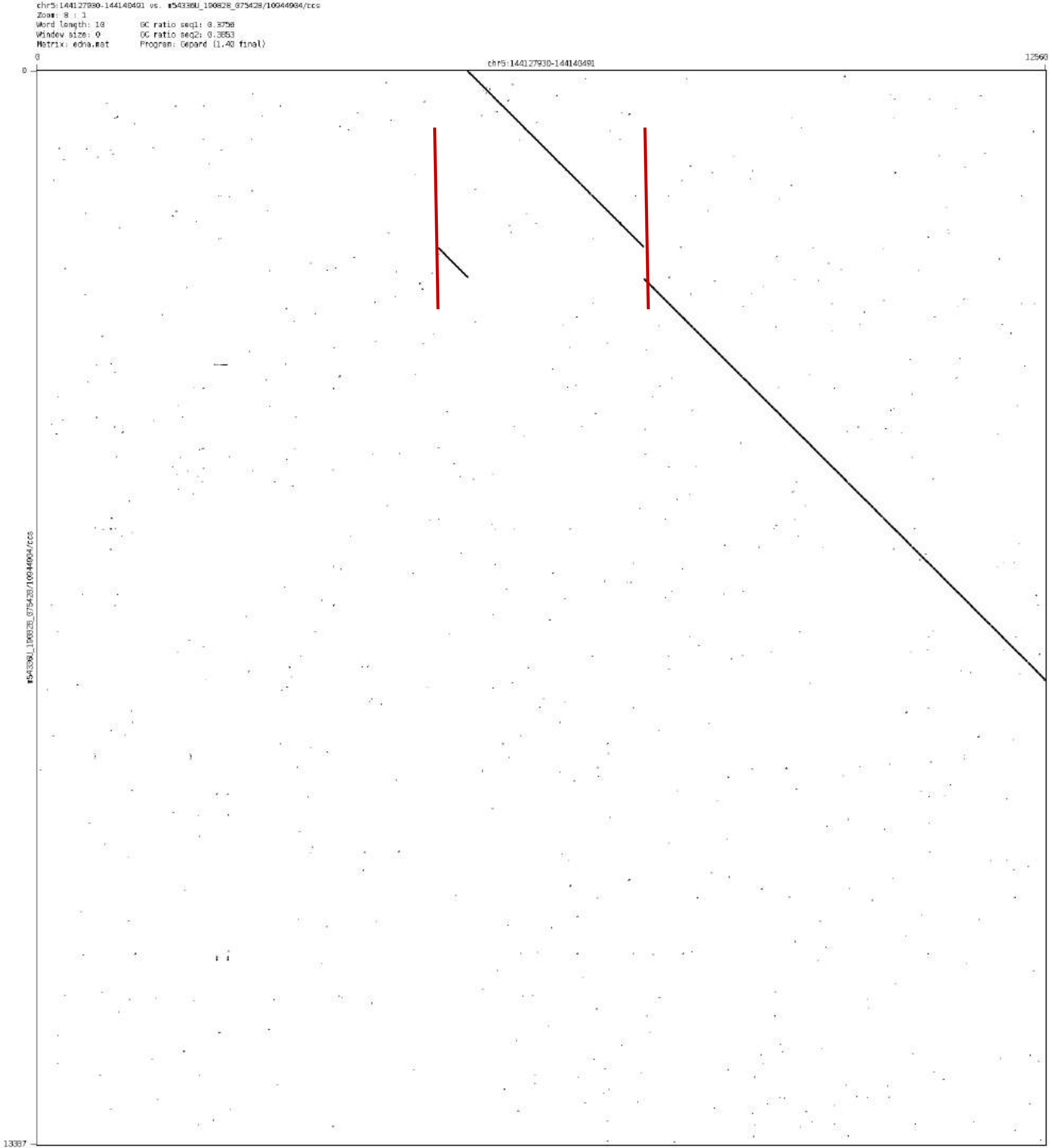

chr5:144127930-144140491 vs. m54336U_190828_075428/10944934/ccs
Zoom: 8 : 1
Word length: 10
Window size: 0
Matrix: edna.mat
GC ratio seq1: 0.3758
GC ratio seq2: 0.3853
Program: Gepard (1.40 final)
0
chr5:144127930-144140491
12560
0
13387
m54336U_190828_075428/10944934/ccs

chr6:102801951-102802684

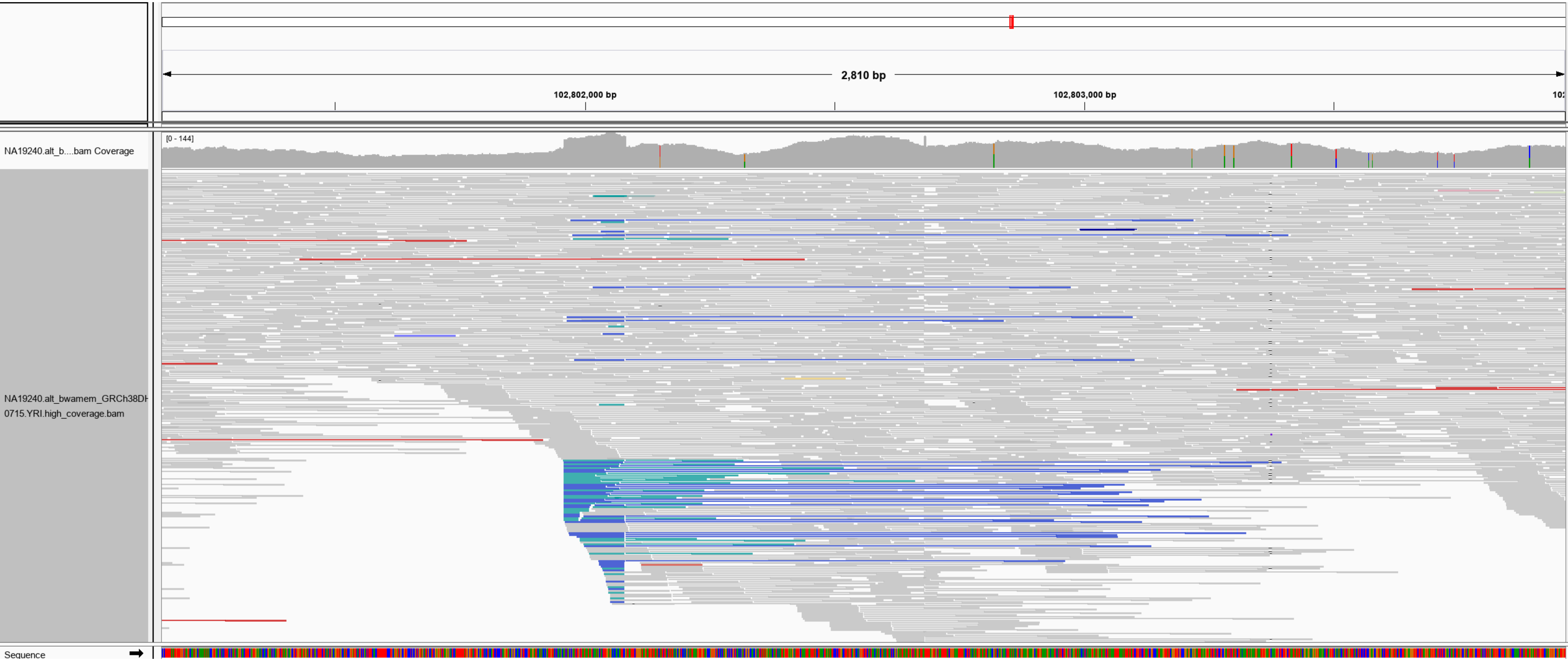

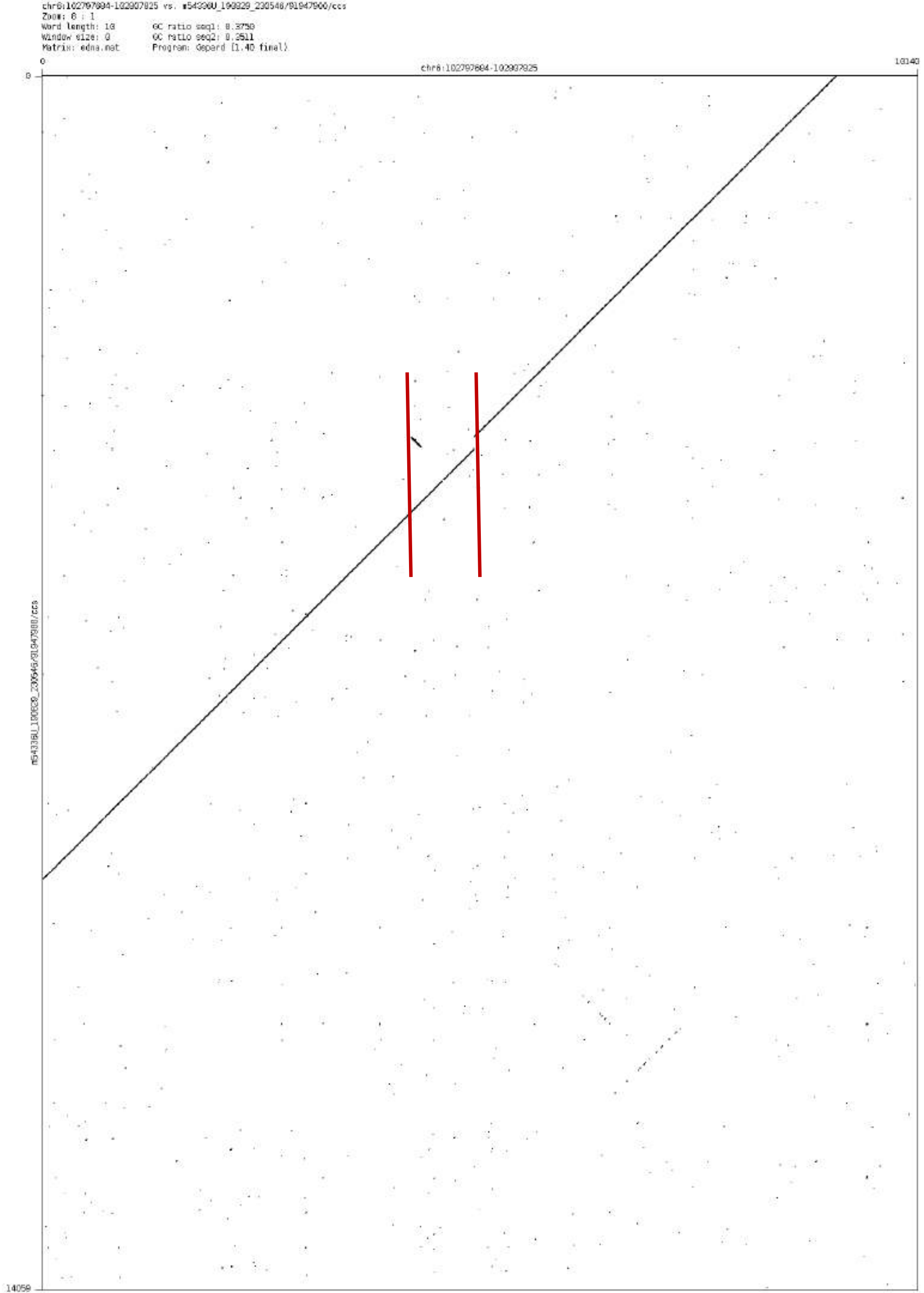
chr8:102797884-102907825 vs. m54336U_190829_230546/81947900/ccs
Zoom: 6 : 1
Word length: 10
Window size: 0
Matrix: edna.mat
GC ratio seq1: 0.3750
GC ratio seq2: 0.3511
Program: Gepard [1.40 final]
0
chr8:102797884-102907825
10140
0
14059

chr6:128961308-128962212

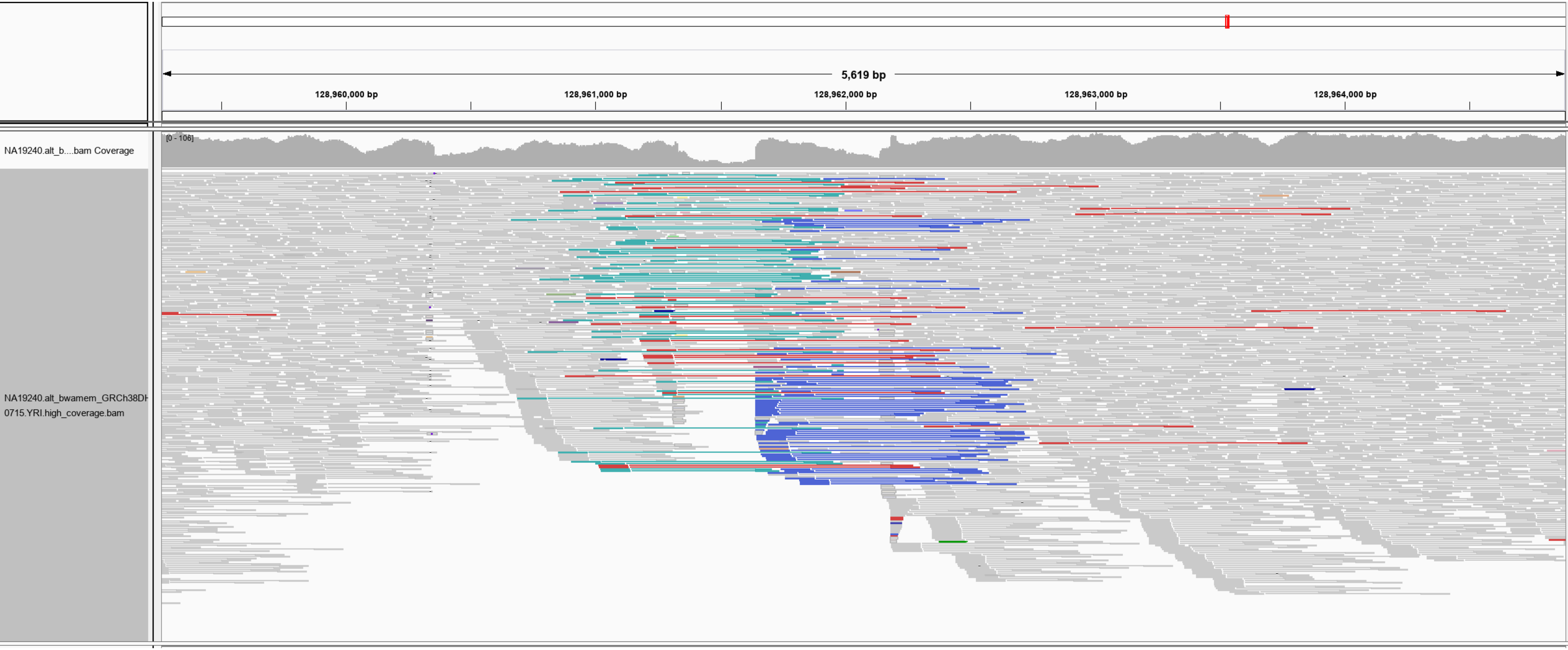

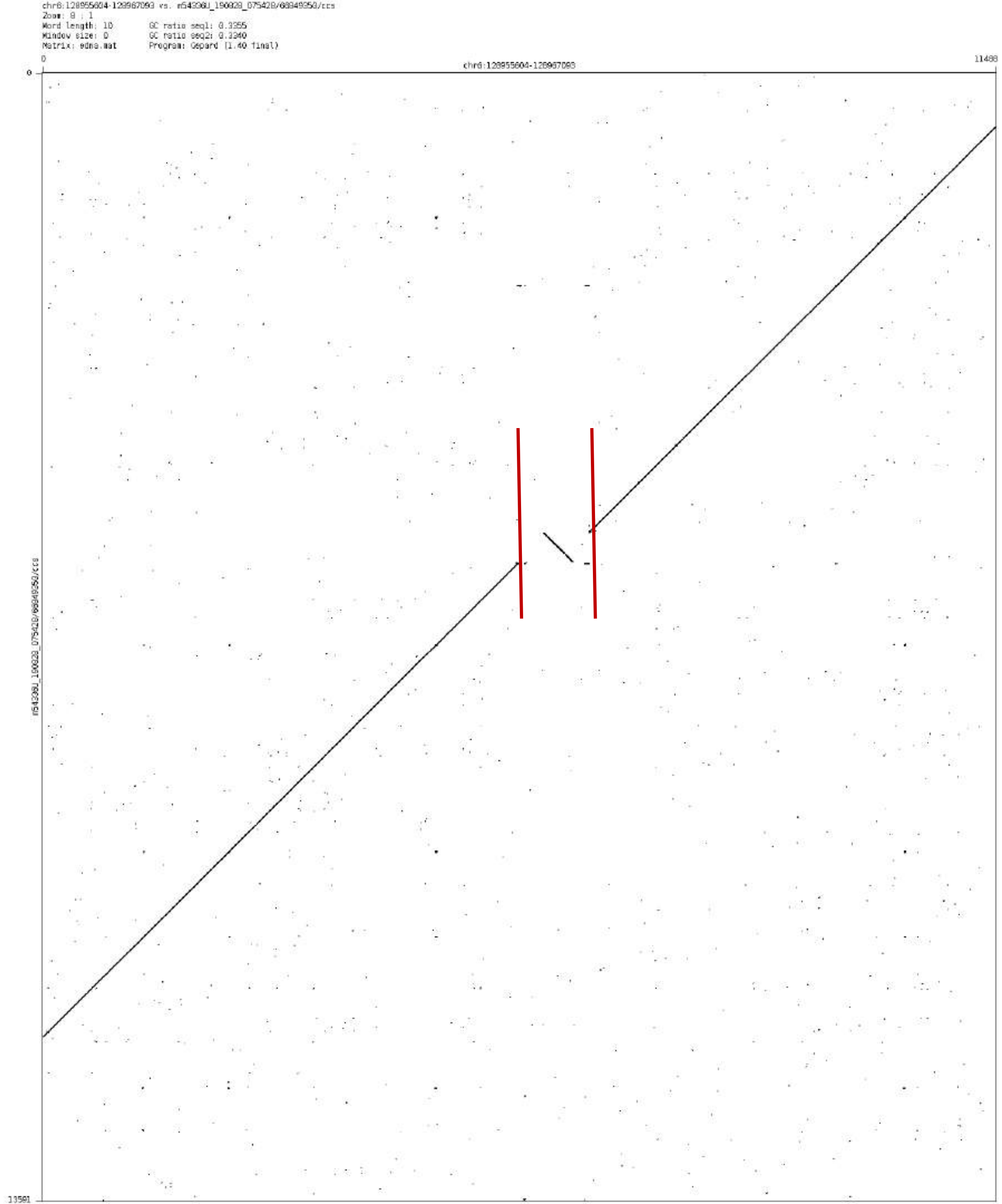
chr8:128955604-128967093 vs. m54336U_190828_075428/68849350/ccs
Zoom: 8 : 1
Word length: 10
Window size: 0
Matrix: edna.mat
GC ratio seq1: 0.3355
GC ratio seq2: 0.3340
Program: Gepard (1.40 final)
0
chr8:128955604-128967093
11488
0
13591

chr6:139281530-139285938

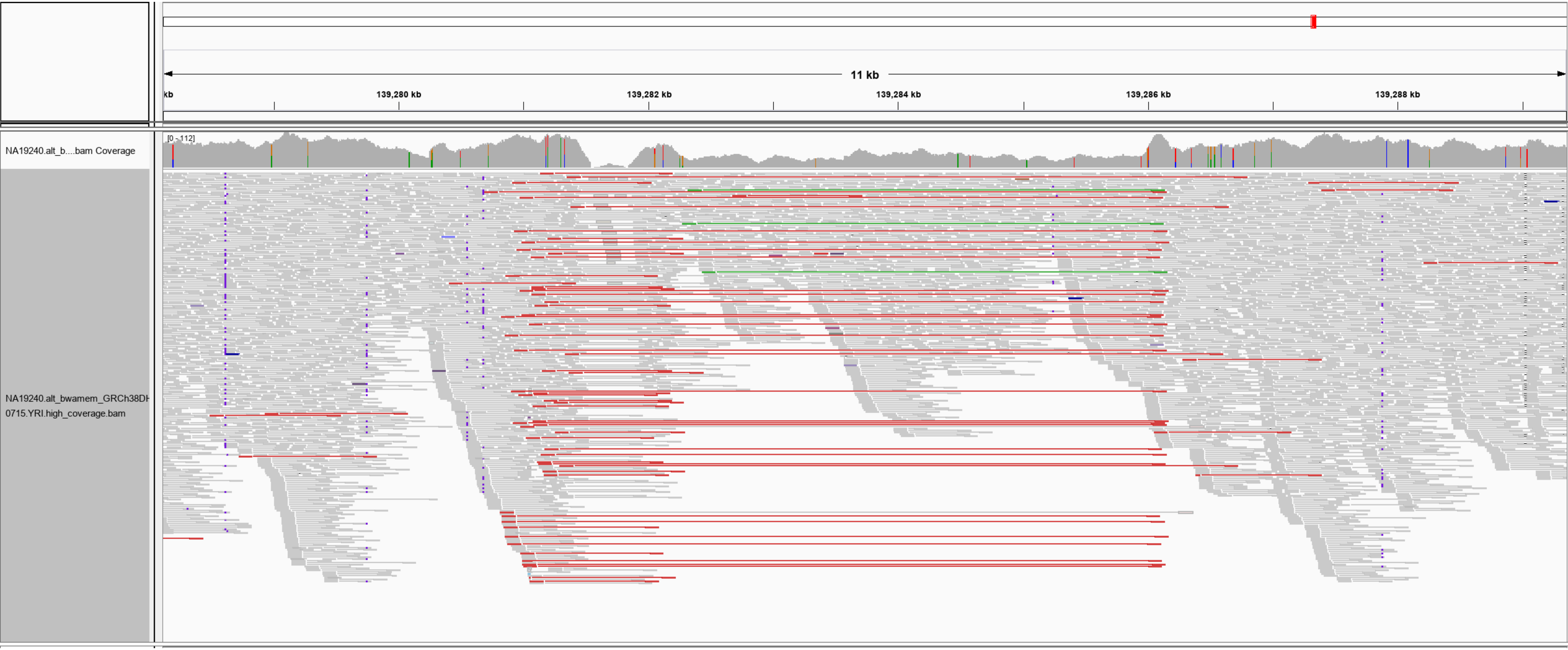

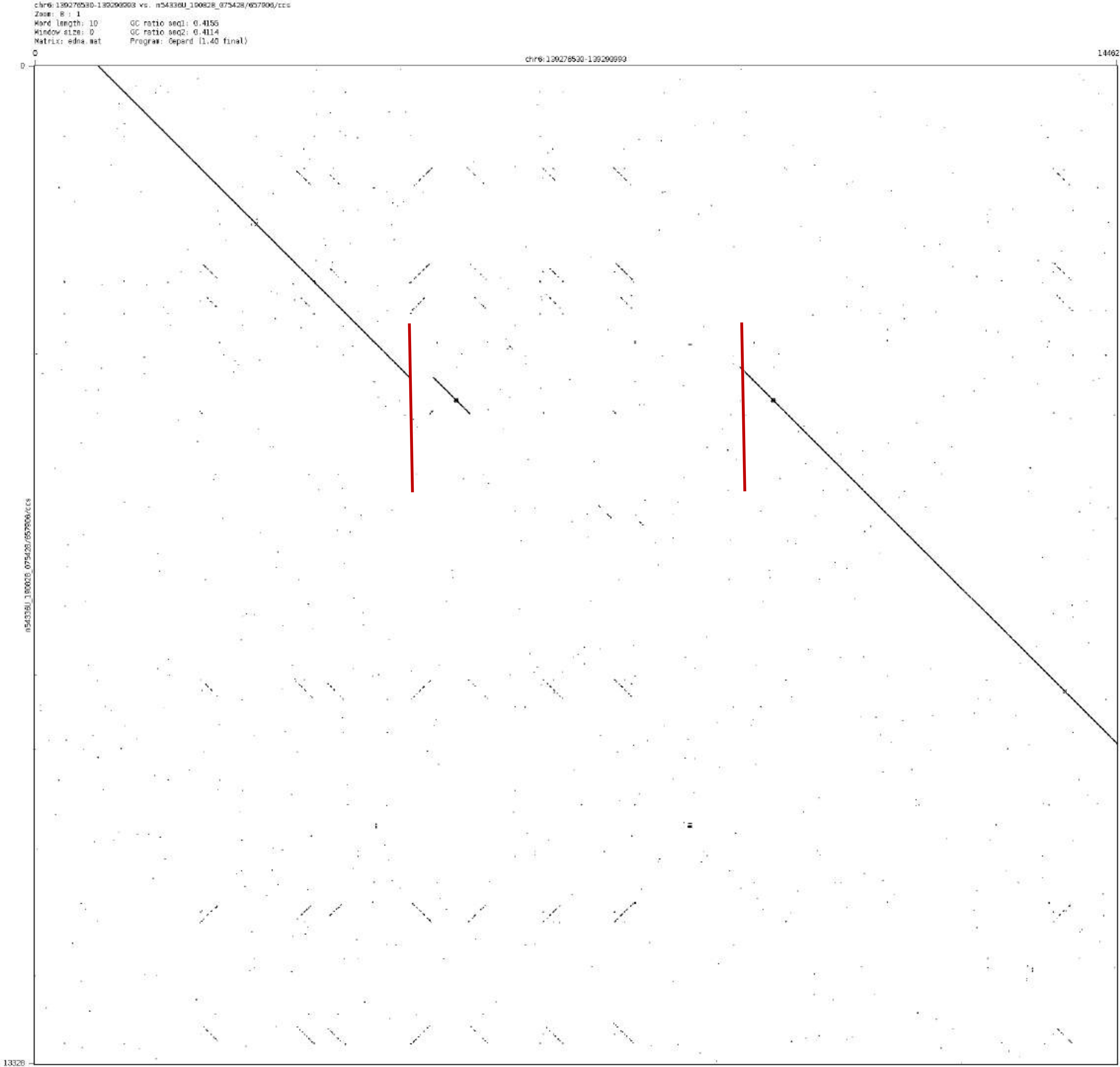
chr6:139276530-139290993 vs. m54336U_190828_075428/657906/ccs
Zoom: 8 : 1
Word length: 10
Window size: 0
Matrix: edna.mat
GC ratio seq1: 0.4155
GC ratio seq2: 0.4114
Program: Gepard (1.40 final)
0
chr6:139276530-139290993
14462
0
m54336U_190828_075428/657906/ccs
13328

chr7:83317161-83318171

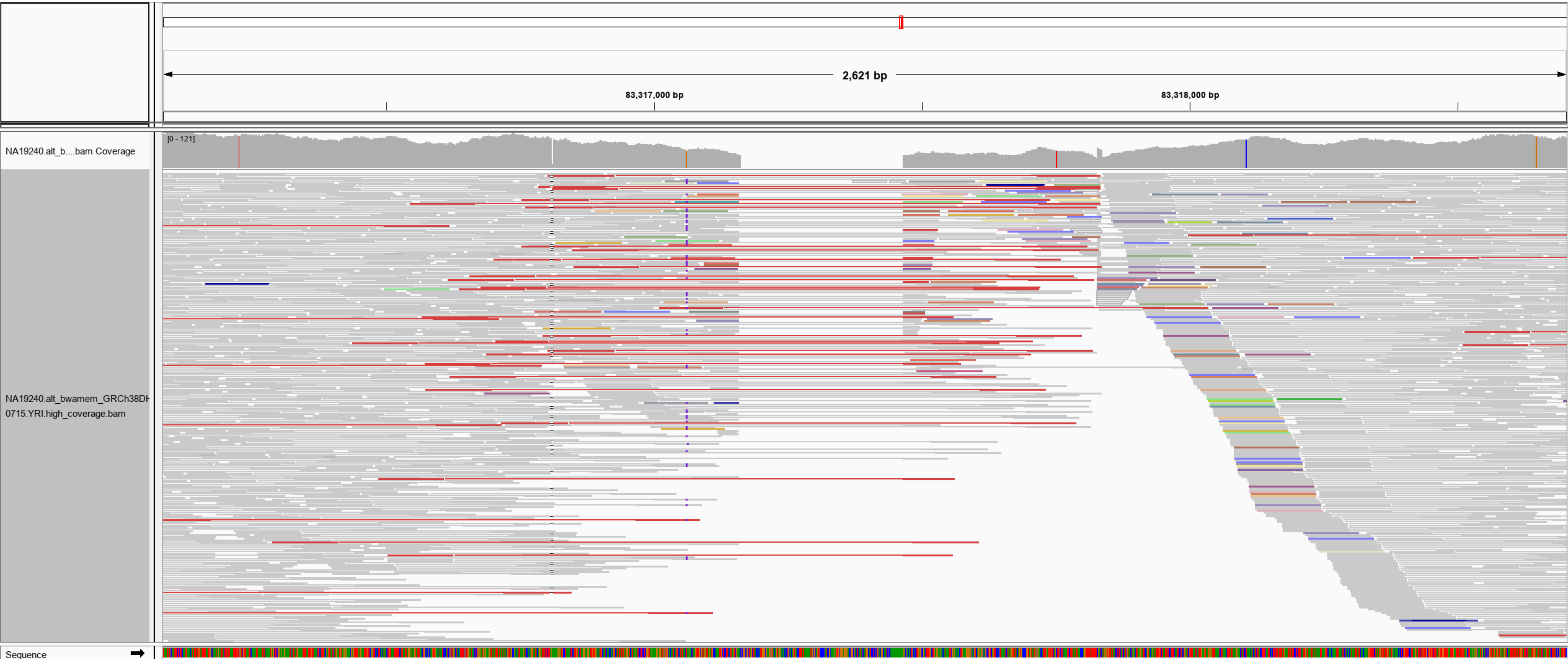

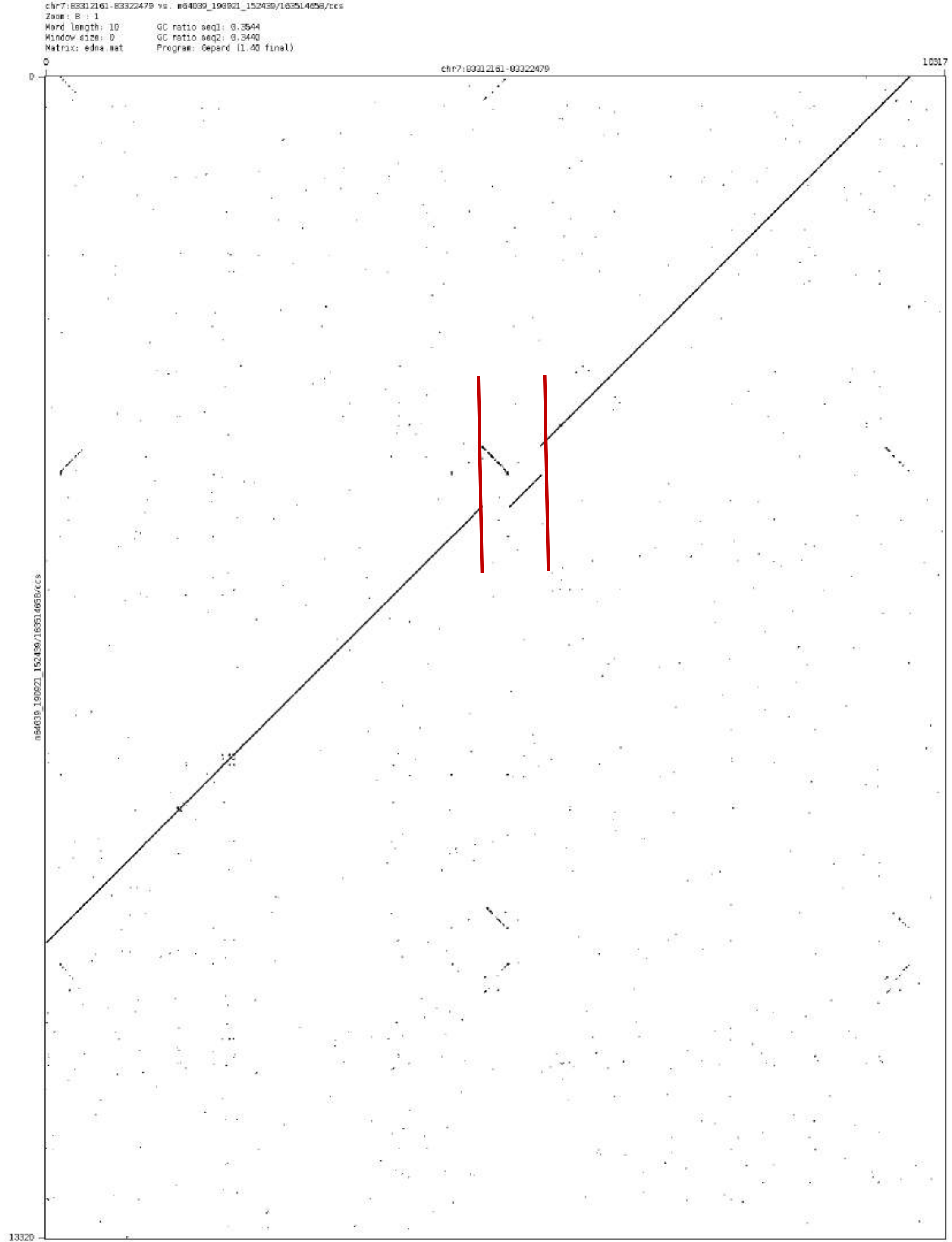
chr7:83312161-83322479 vs. m64039_190921_152439/163514658/ccs
Zoom: 8 : 1
Word length: 10
GC ratio seq1: 0.3544
Window size: 0
GC ratio seq2: 0.3440
Matrix: edna.mat
Program: Gepard (1.40 final)
0
10317
chr7:83312161-83322479
0
13320
m64039_190921_152439/163514658/ccs

chr7: 89324124-89324962

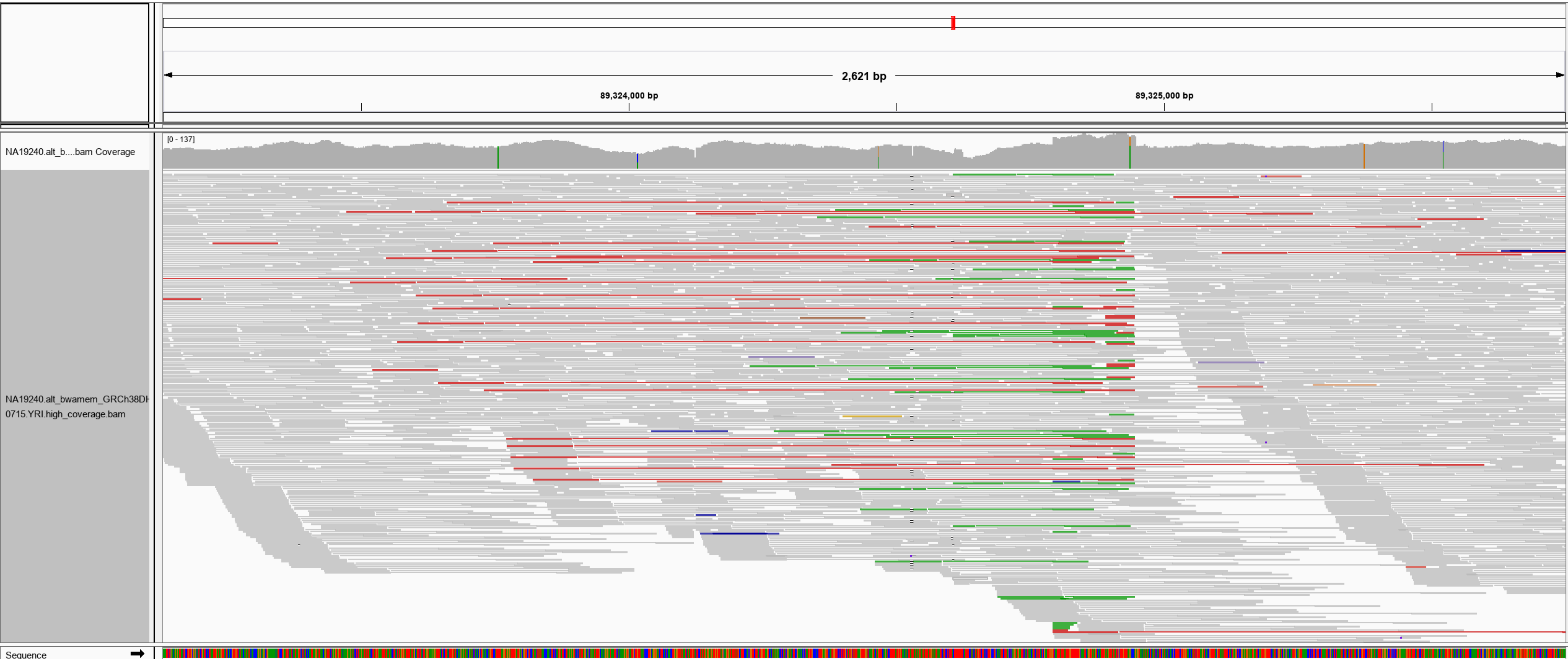

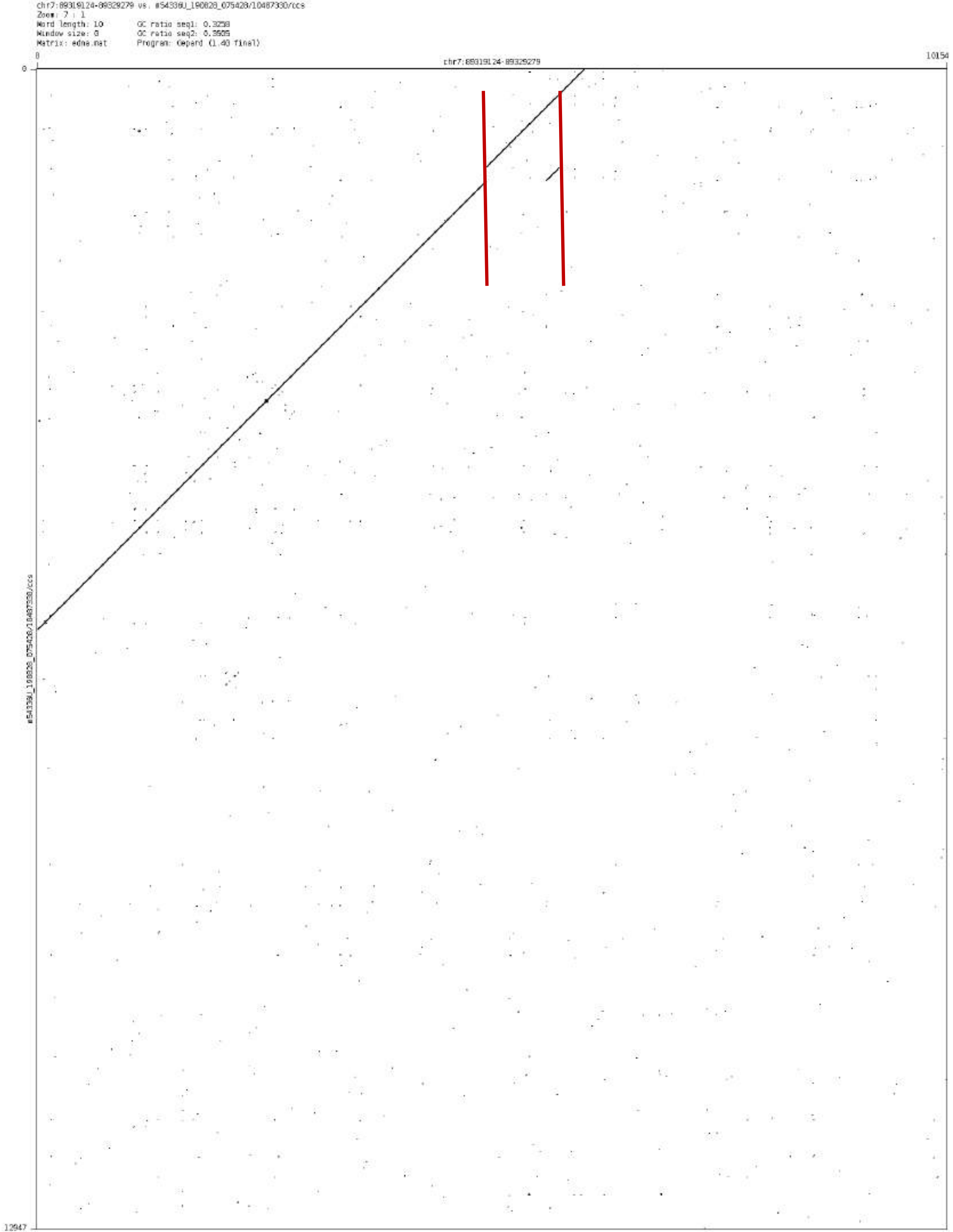

chr7:89319124-89329279 vs. #54336U_190828_075428/10487330/ccs
Zoom: 7 : 1
Word length: 10
Window size: 0
Matrix: edna.mat
GC ratio seq1: 0.3258
GC ratio seq2: 0.3505
Program: Gepard (1.40 final)
0
chr7:89319124-89329279
10154
0
#54336U_190828_075428/10487330/ccs
12947

chr7:151312949-151314989

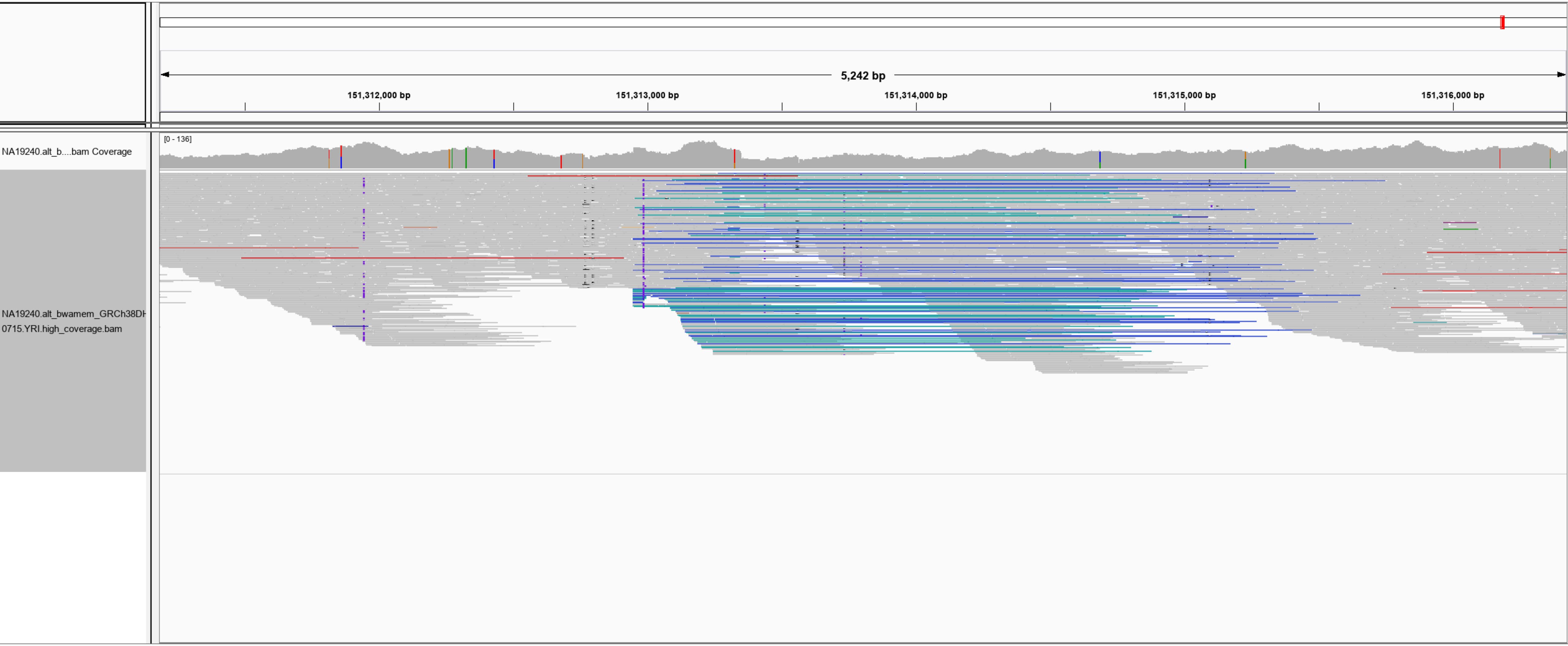

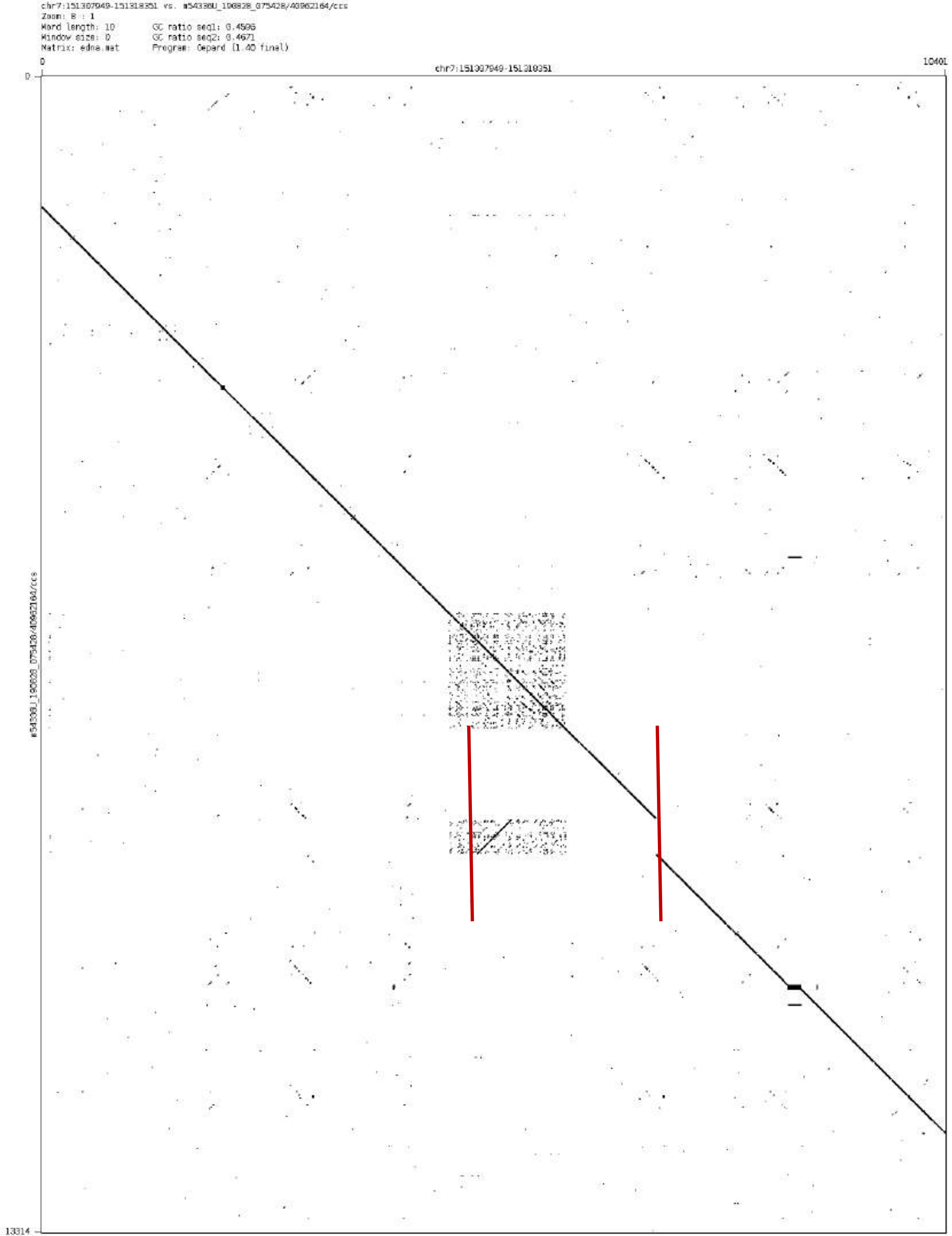
chr7:151307949-151318351 vs. m54330U_190828_075428/40962164/ccs
Zoom: 8 : 1
Word length: 10
GC ratio seq1: 0.4596
Window size: 0
GC ratio seq2: 0.4671
Matrix: edna.mat
Program: Gepard (1.40 final)
0
10401
chr7:151307949-151318351
0
13314

chr9:69280304-69281825

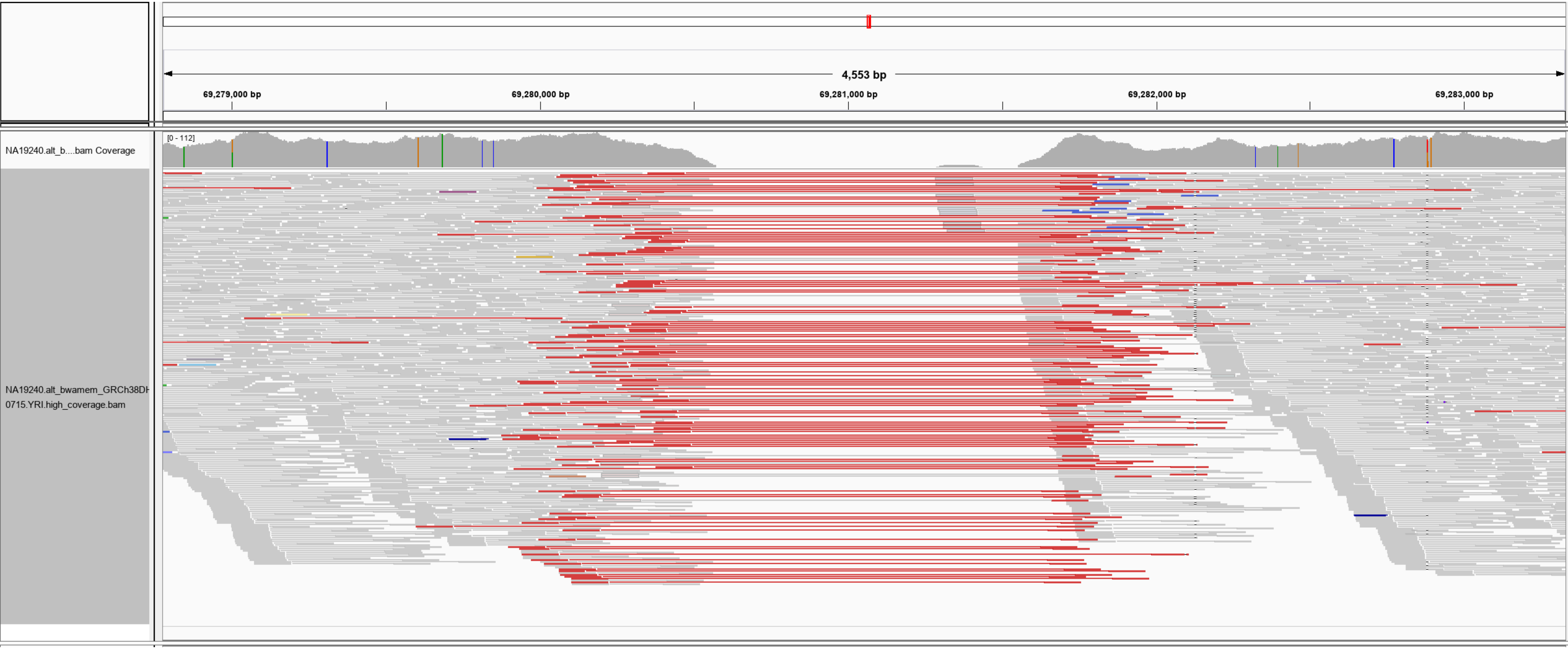

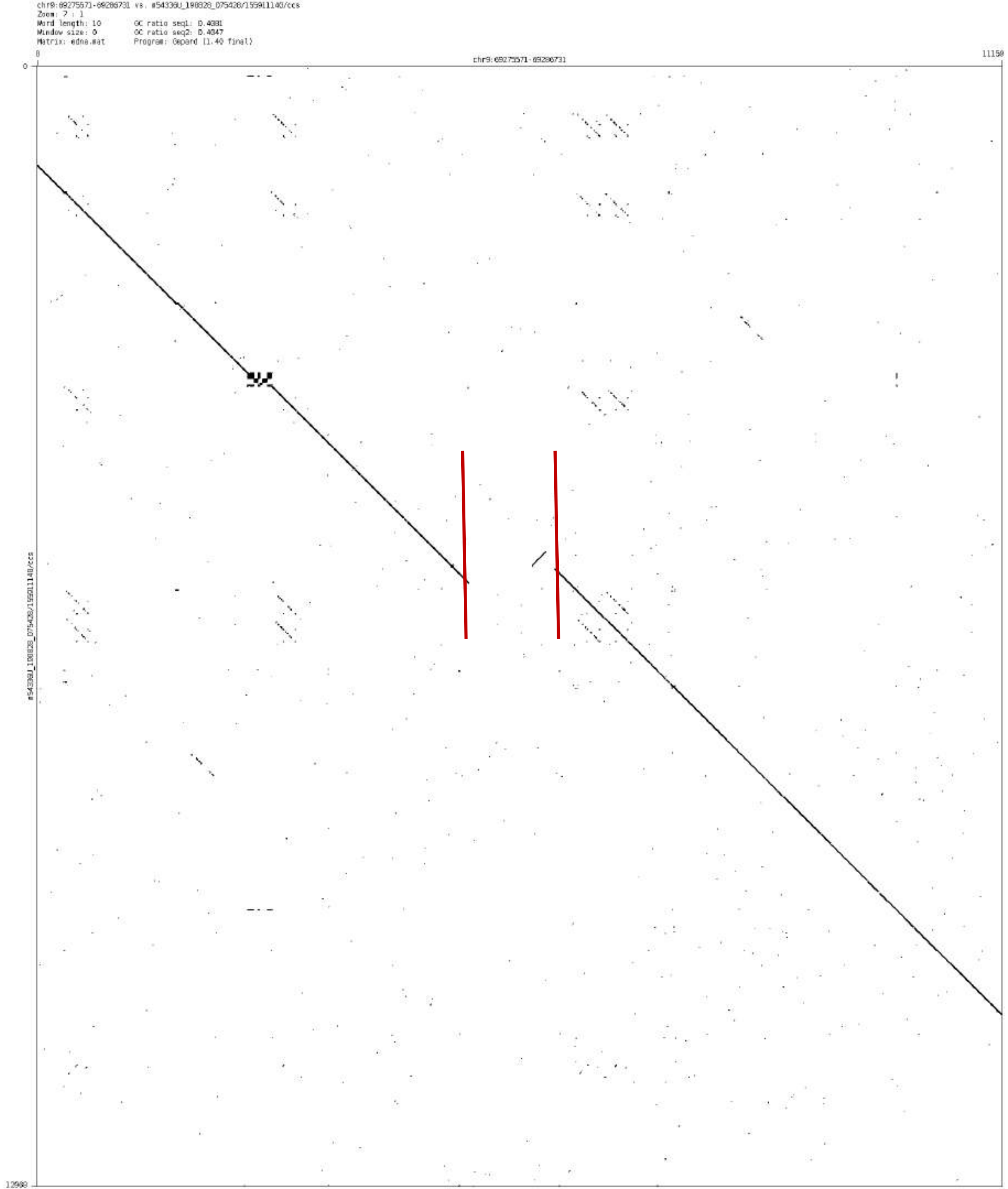
Zoom: 7 : 1
Word length: 10
Window size: 0
Matrix: edna.mat
GC ratio seq1: 0.4381
GC ratio seq2: 0.4347
Program: Gepard (1.40 final)
0
11150
12988

chr9:86539584-86541086

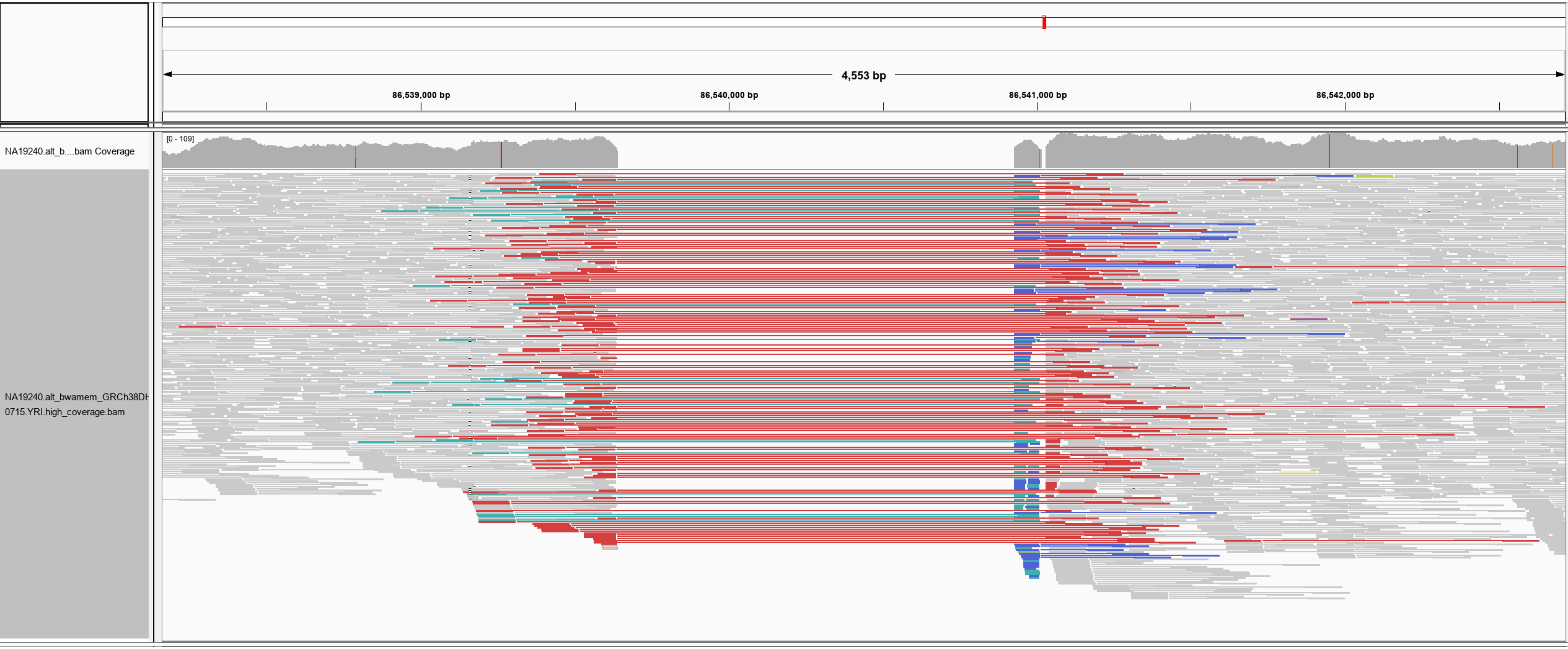

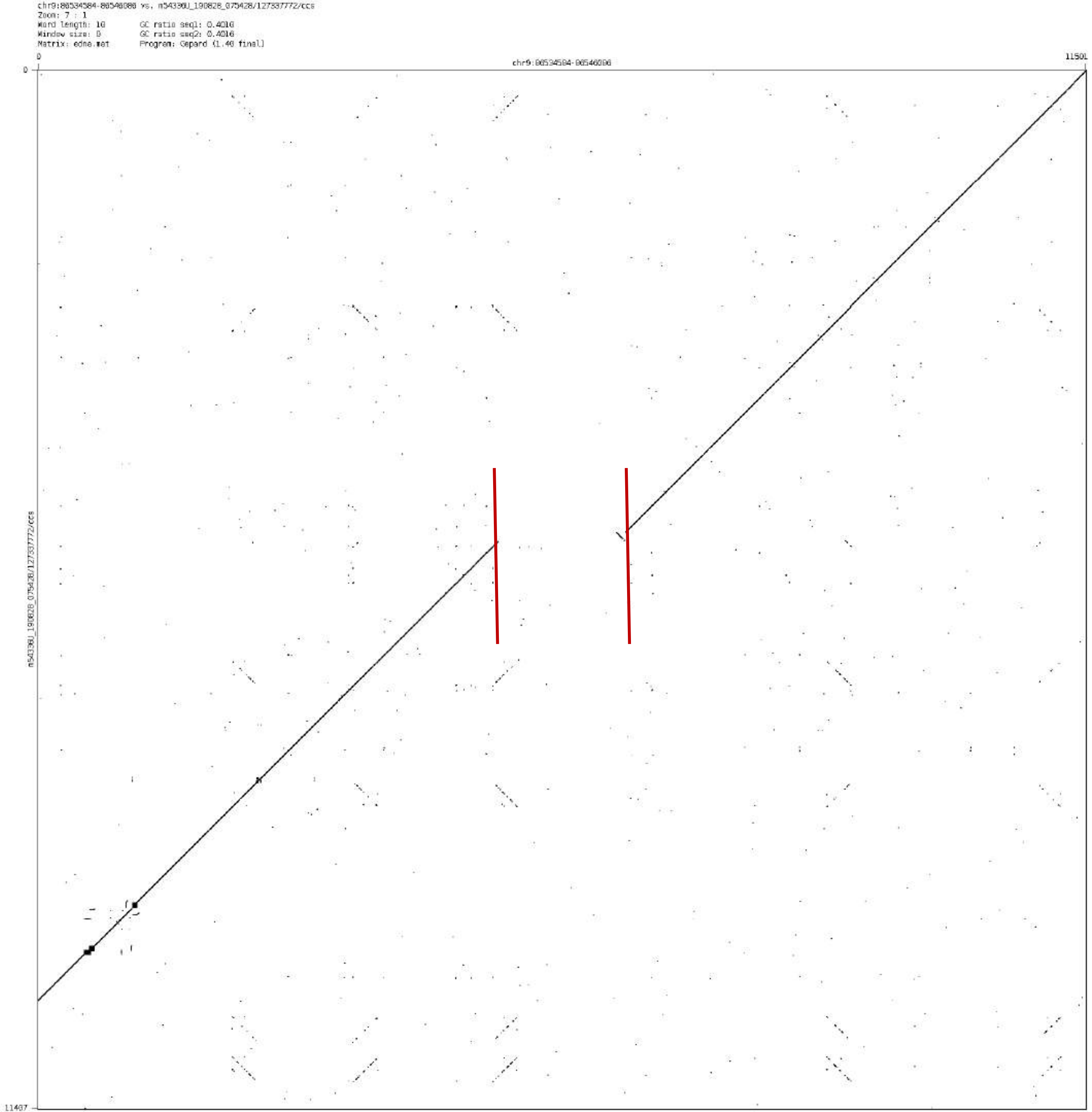
Zoom: 7 : 1
Word length: 10
Window size: 0
Matrix: edna.mat
GC ratio seq1: 0.4016
GC ratio seq2: 0.4016
Program: Gepard (1.40 final)
0
11501
11407

chr9:105054355-105055067

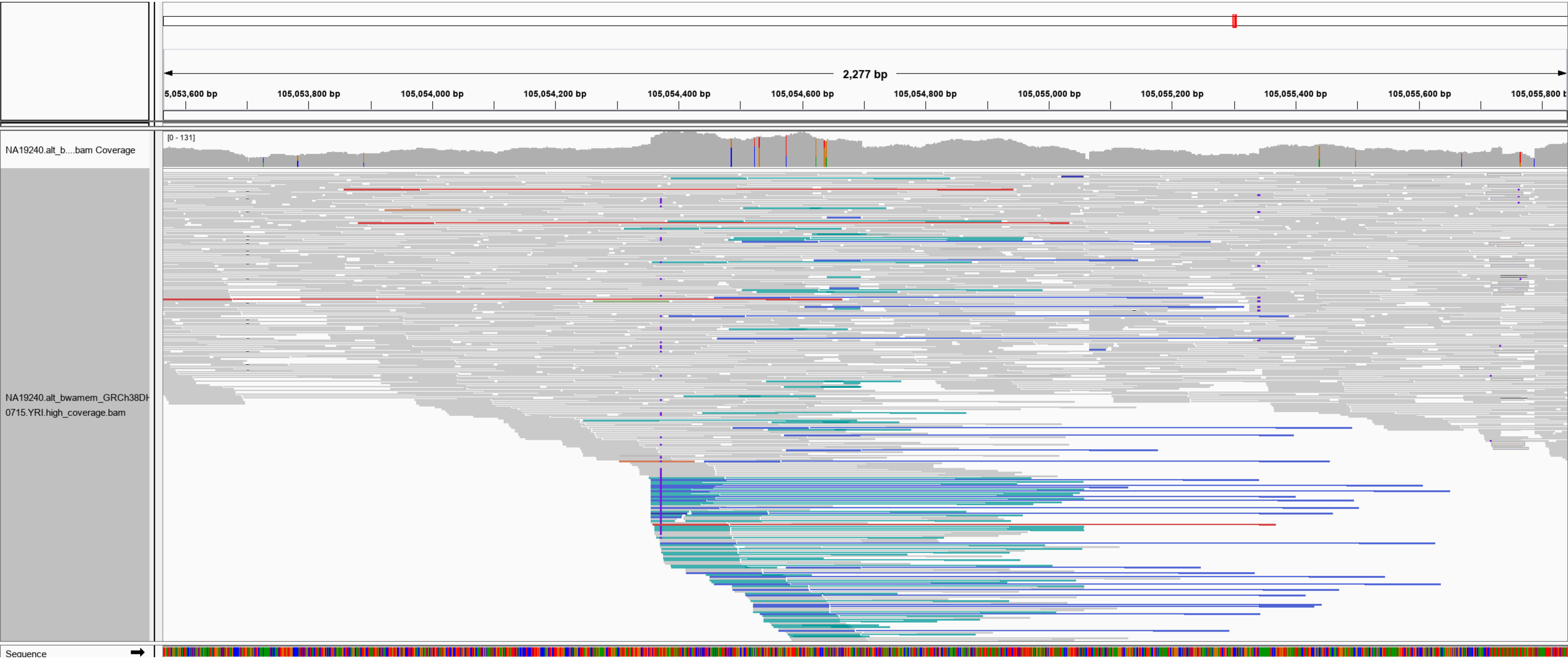

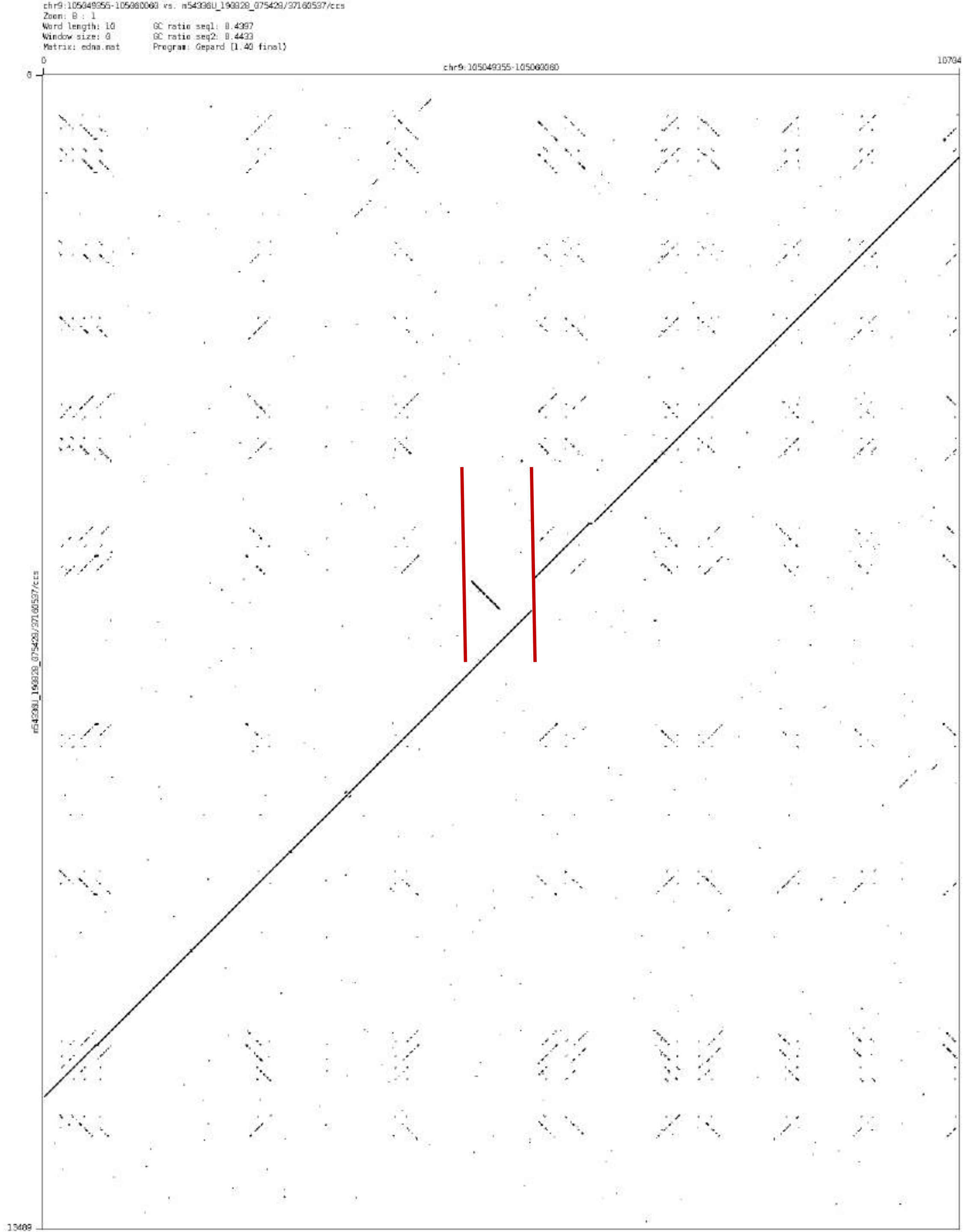
chr9:105049355-105060060 vs. m54336U_190828_075428/37160537/ccs
Zoom: 8 : 1
Word length: 10
Window size: 0
Matrix: edna.mat
GC ratio seq1: 0.4397
GC ratio seq2: 0.4433
Program: Gepard [1.40 final]
0
chr9:105049355-105060060
10704
0
13409
m54336U_190828_075428/37160537/ccs

chr9:109523550-109524392

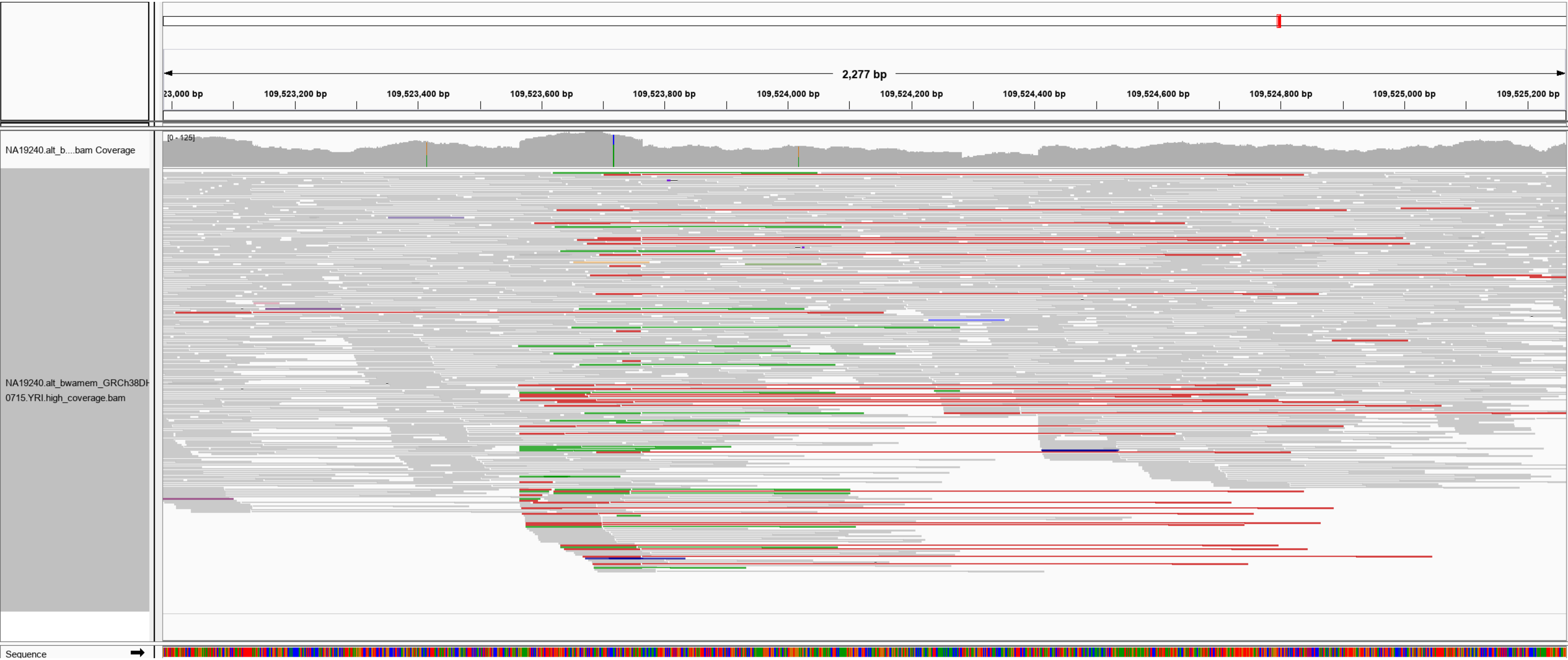

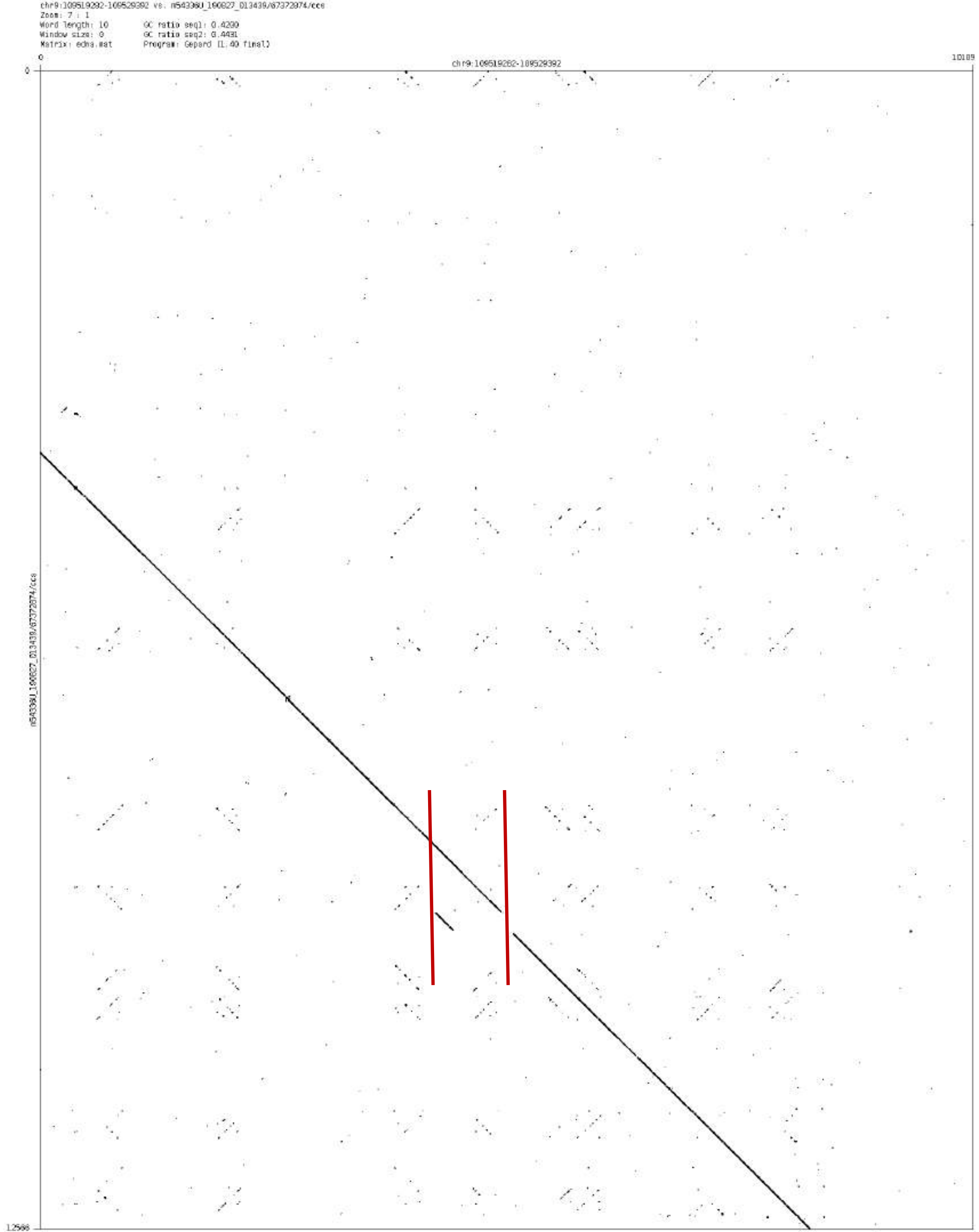

chr9:109519292-109529392 vs. m54336U_190827_013439/67372974/ccs
Zoom: 7 : 1
Word length: 10
Window size: 0
Matrix: edna.mat
GC ratio seq1: 0.4290
GC ratio seq2: 0.4431
Program: Gepard (1.40 final)
0
chr9:109519262-109529392
10189
0

chr9:132883700-132884812

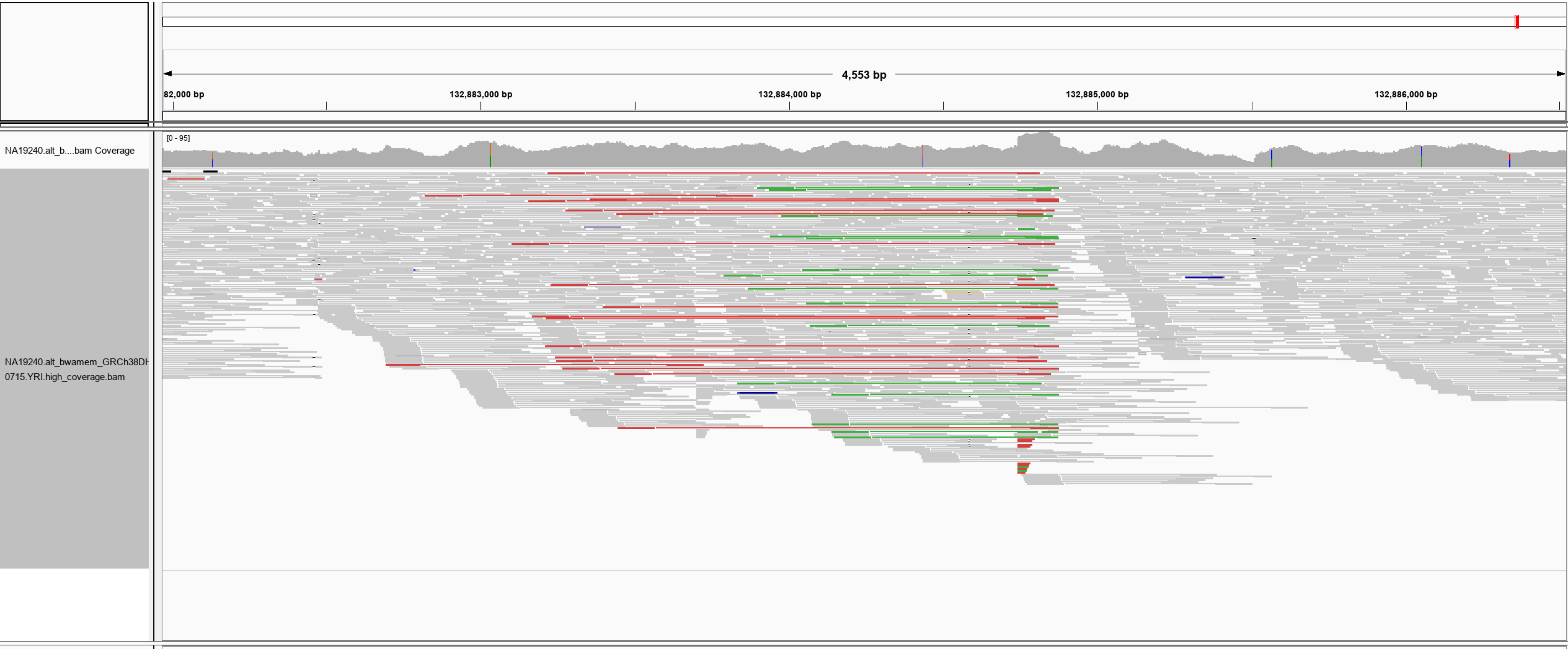

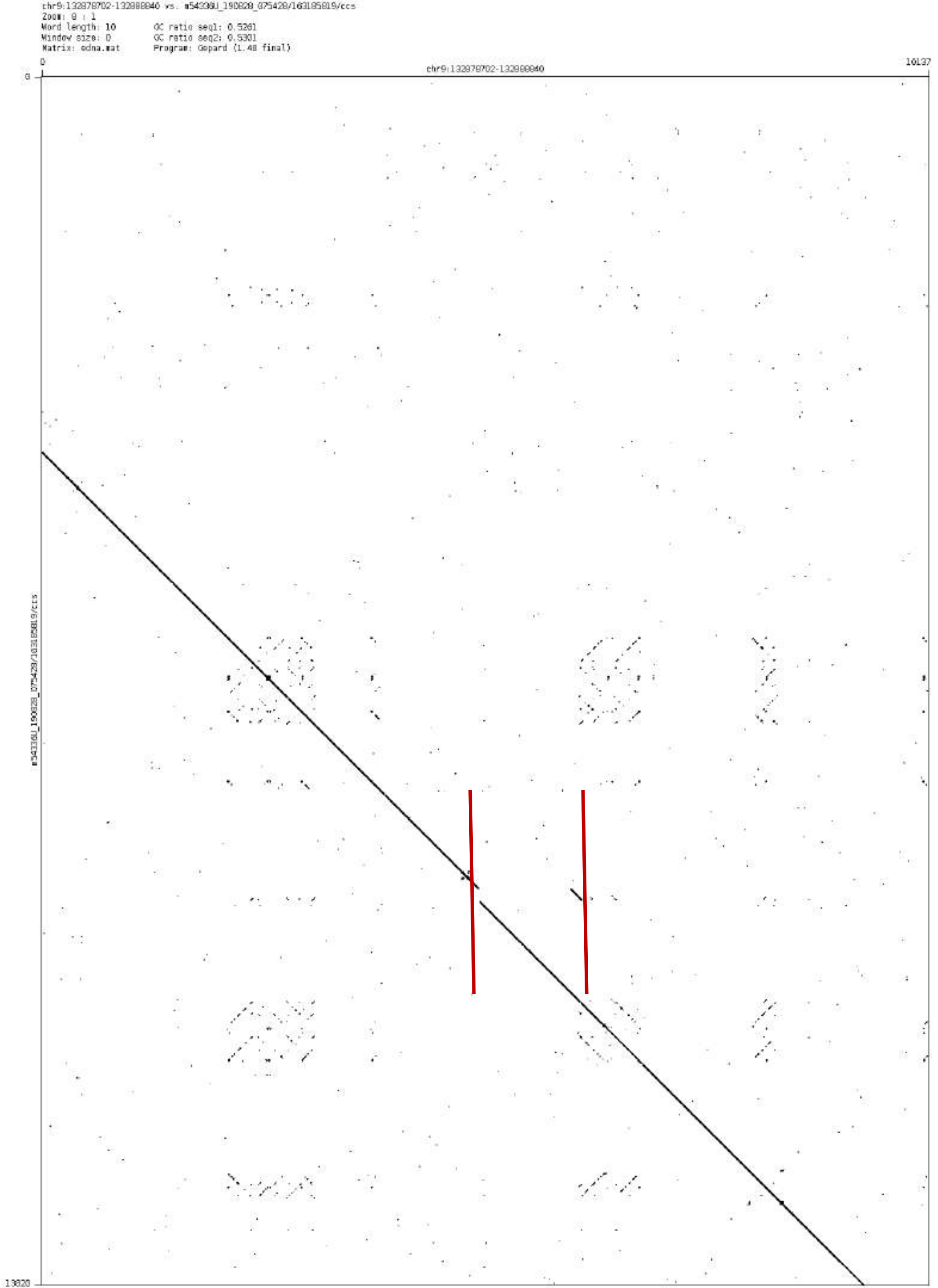
chr9:132878702-132888840 vs. m54336U_190828_075428/163185819/ccs
Zoom: 8 : 1
Word length: 10
GC ratio seq1: 0.5261
Window size: 0
GC ratio seq2: 0.5301
Matrix: ednа.mat
Program: Gepard (1.40 final)
0
10137
chr9:132878702-132888840
0
13820
m54336U_190828_075428/163185819/ccs

chr10:44927495-44928688

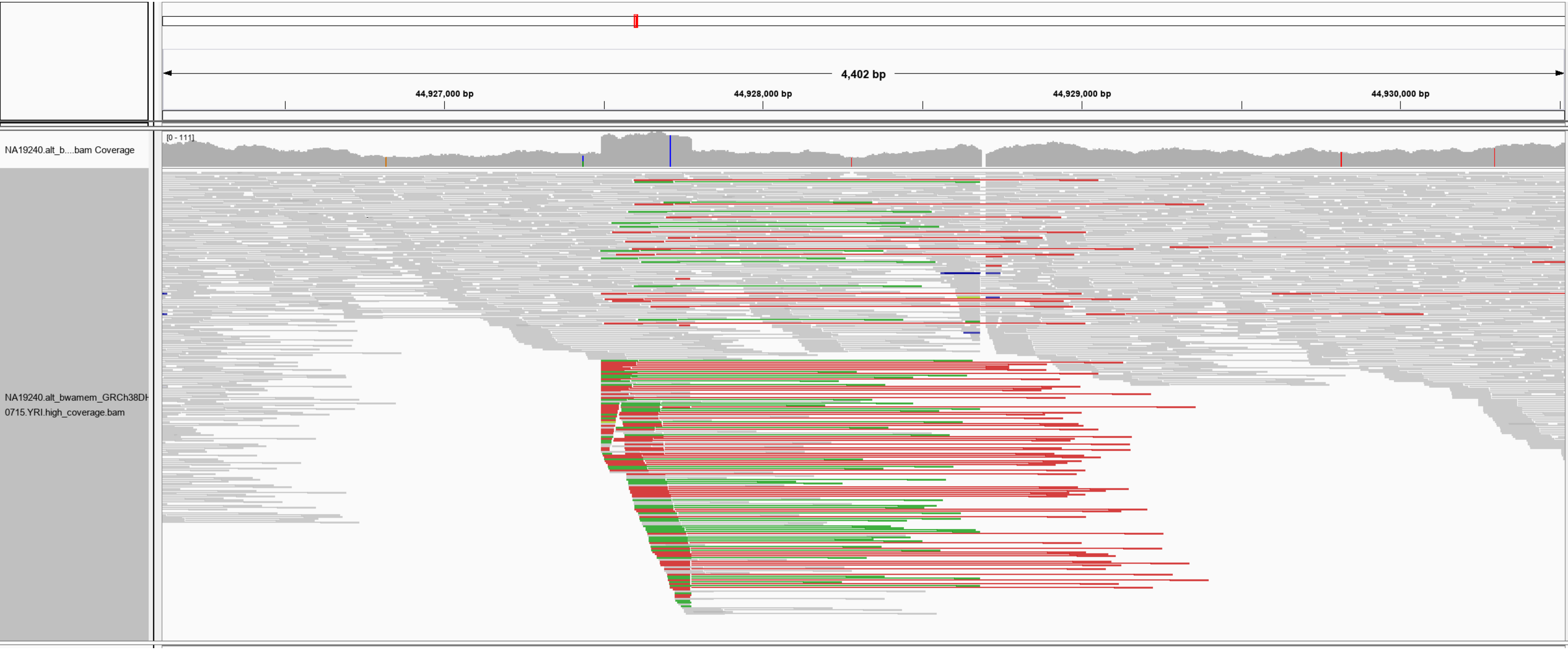

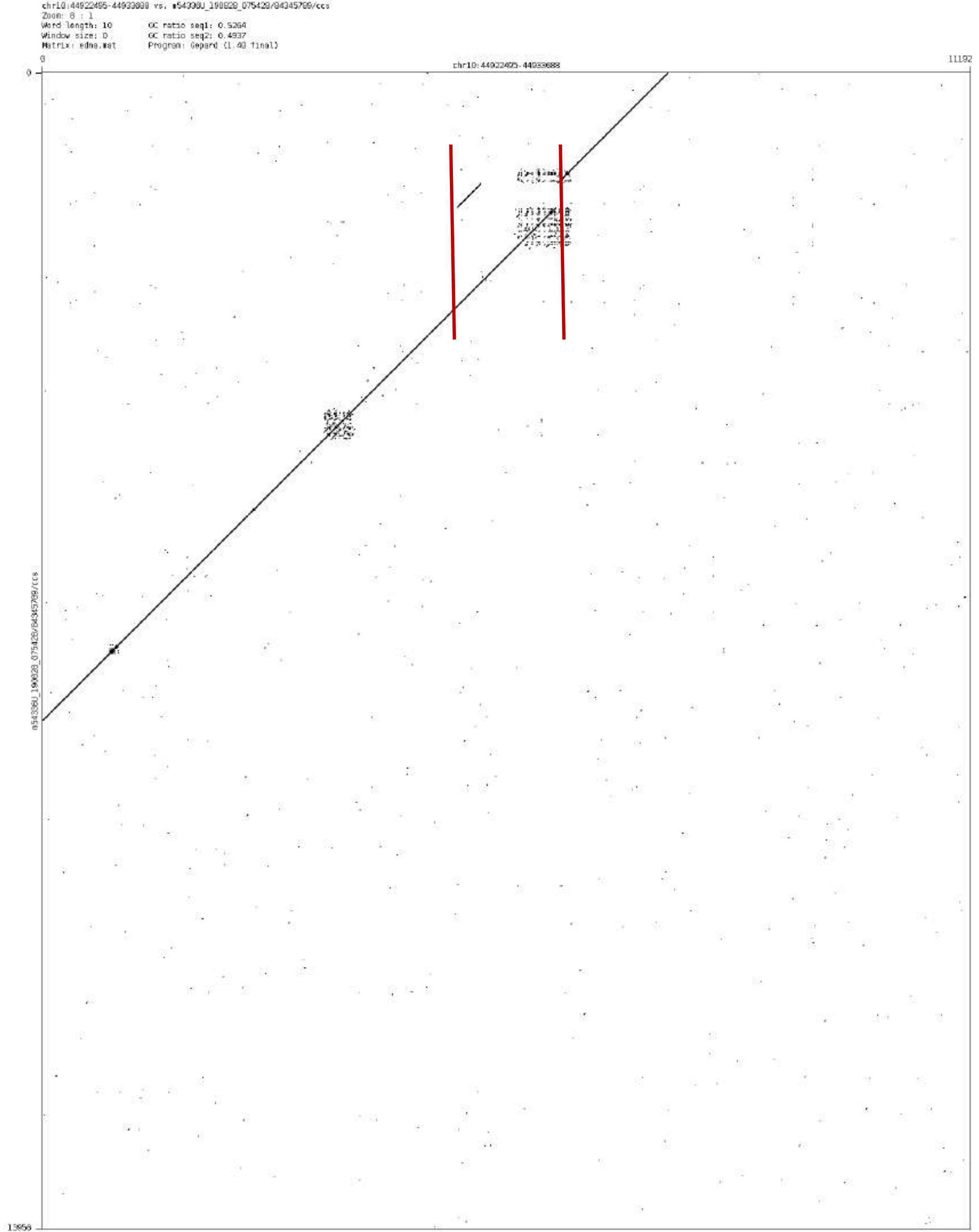
chr10:44922495-44933888 vs. m54330U_190828_075429/84345799/ccs
Zoom: 8 : 1
Word length: 10
Window size: 0
Matrix: edna.mat
GC ratio seq1: 0.5264
GC ratio seq2: 0.4937
Program: Gepard (1.40 final)
chr10:44922495-44933888
11192
13956

chr11:6042862-6044585

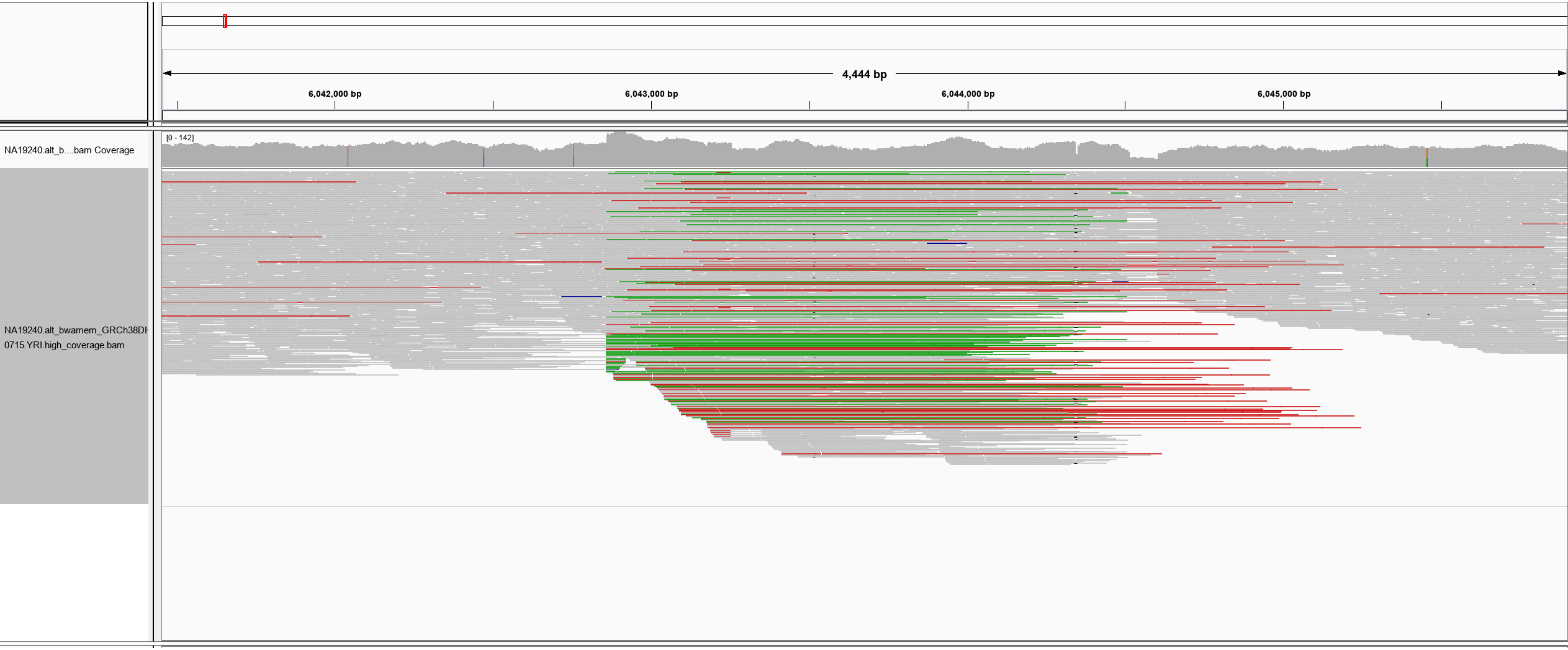

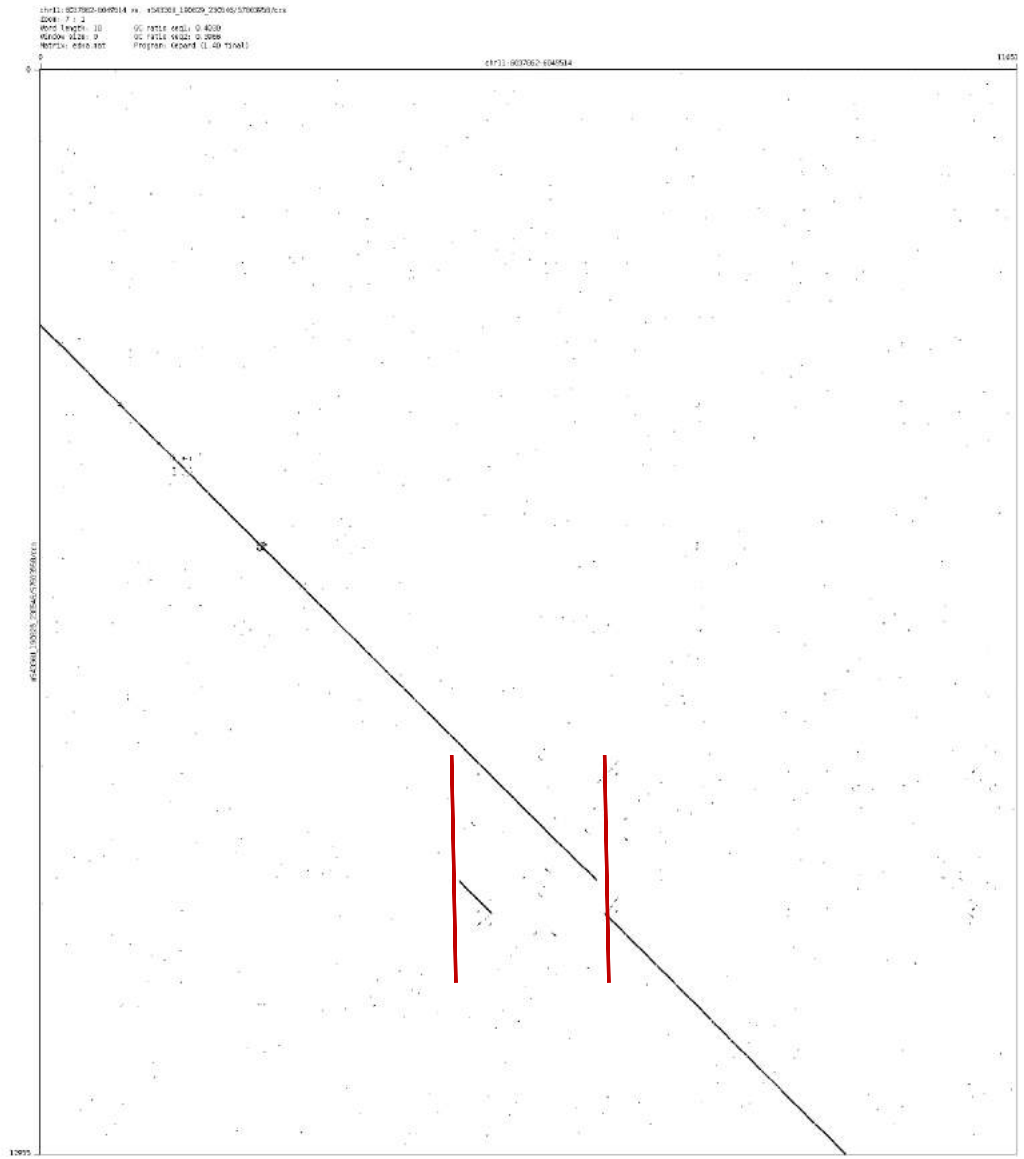
Word length: 10
Matrix: edna.mat
Program: Gepard (1.40 final)

chr11:24882412-24883333

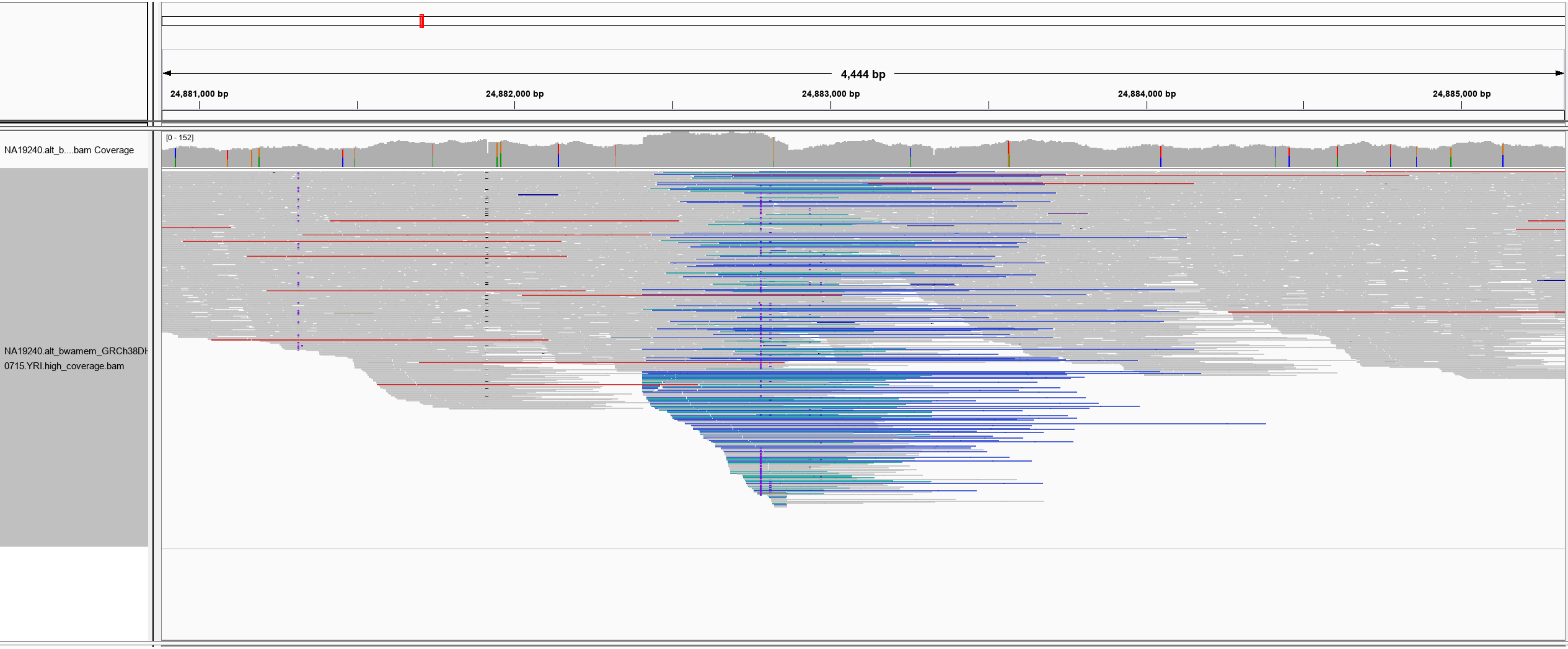

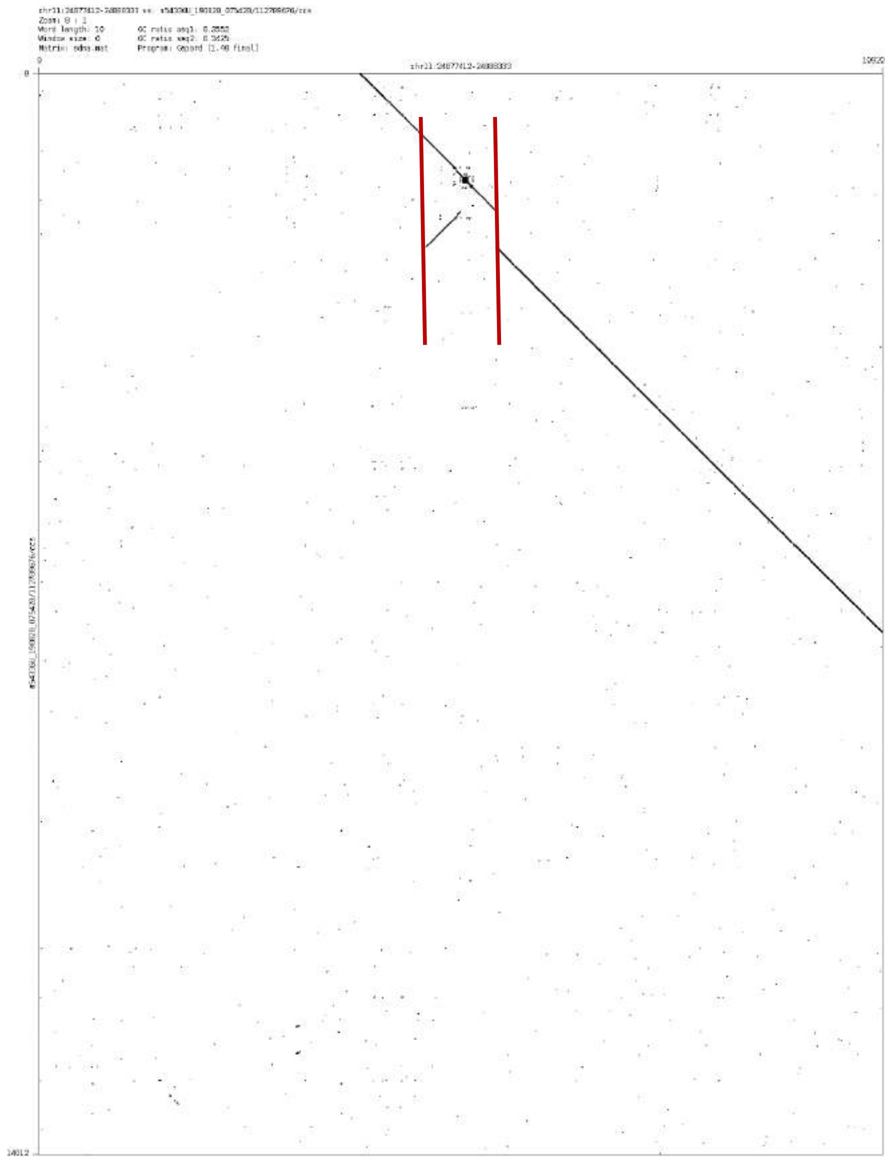

Zoom: 8 : 1
Word length: 10
Window size: 0
Matrix: edna.mat
Program: Gepard [1.40 final]
0
10920
14012

chr11:99819304-99820578

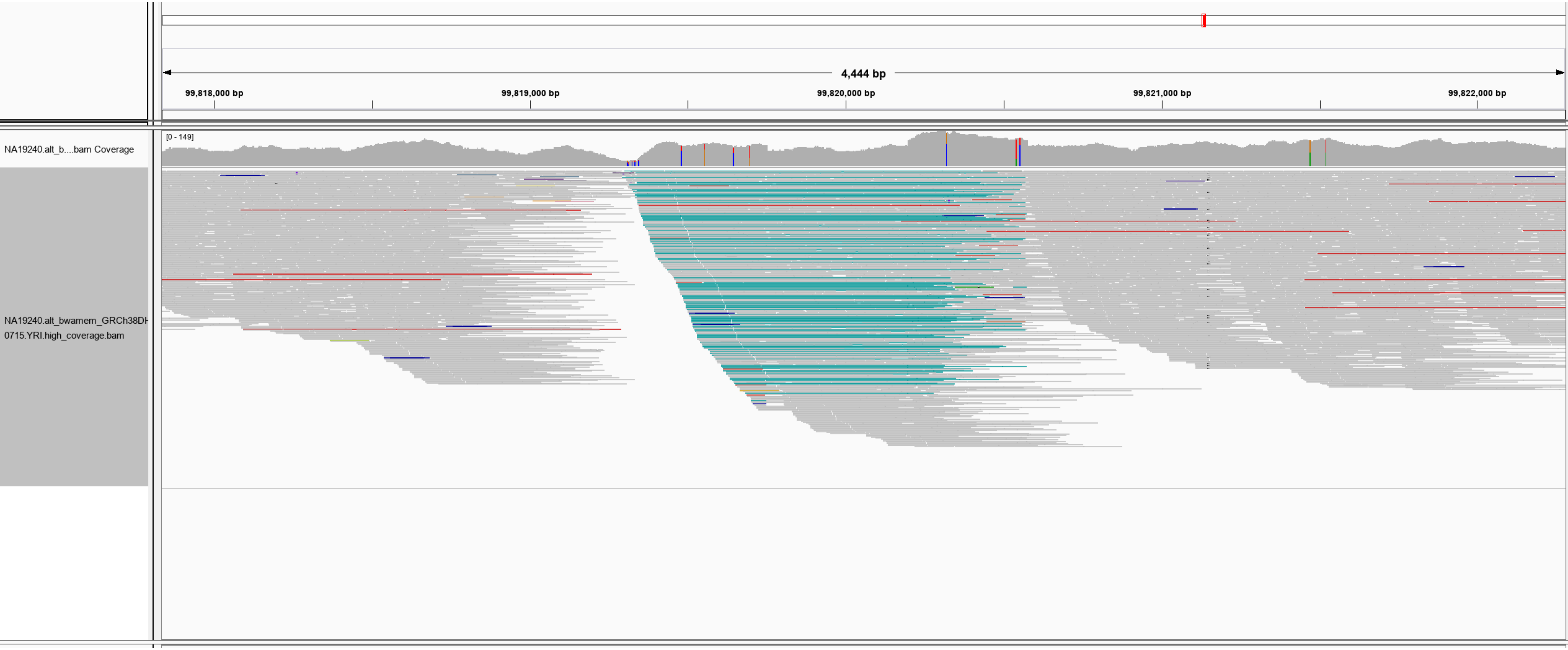

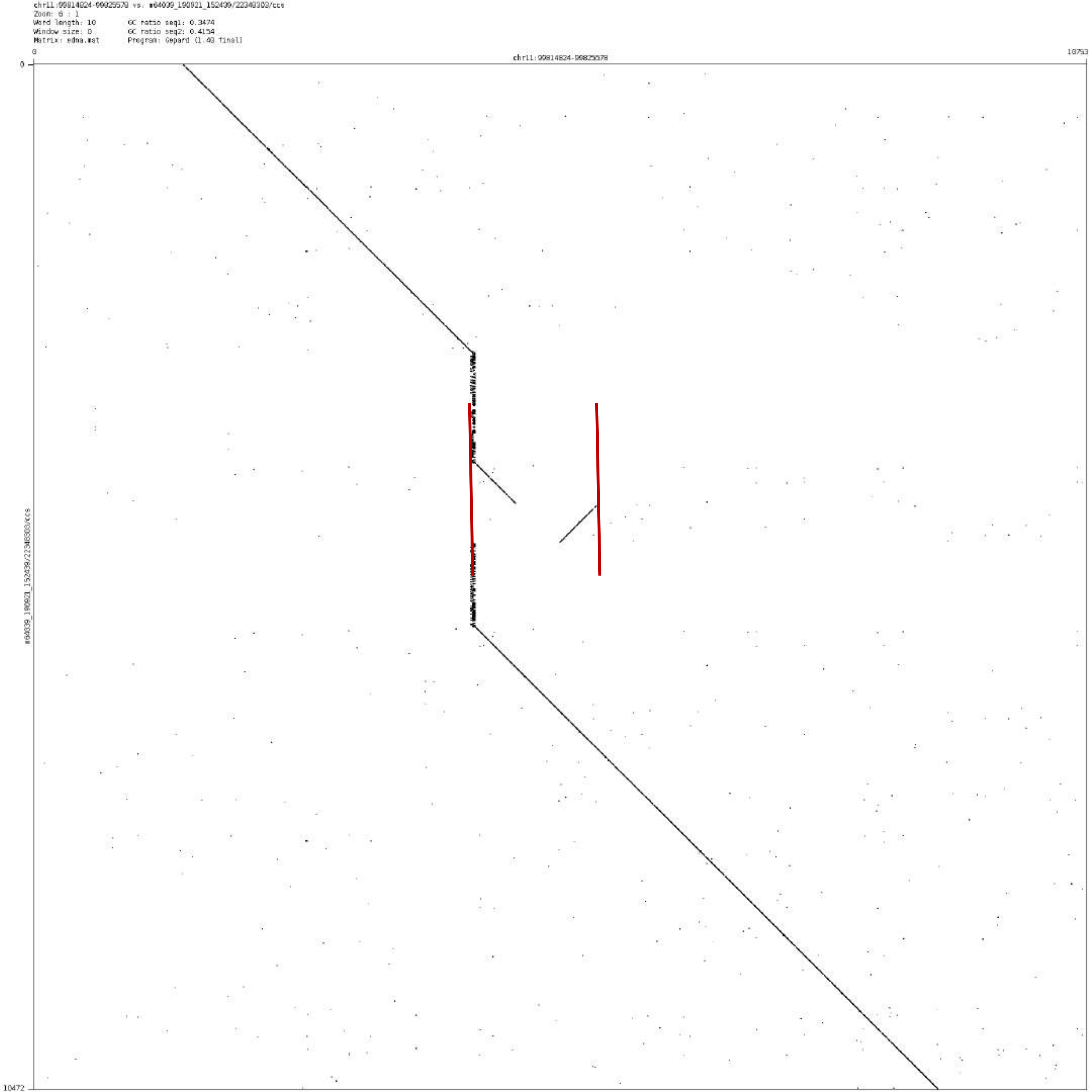

chr11:99814824-99825578 vs. m64039_190921_152439/22348303/ccs
Zoom: 6 : 1
Word length: 10
Window size: 0
Matrix: edna.mat
GC ratio seq1: 0.3474
GC ratio seq2: 0.4154
Program: Gepard (1.40 final)
0
chr11:99814824-99825578
10753
0
m64039_190921_152439/22348303/ccs
10472

chr11:120061454-120062063

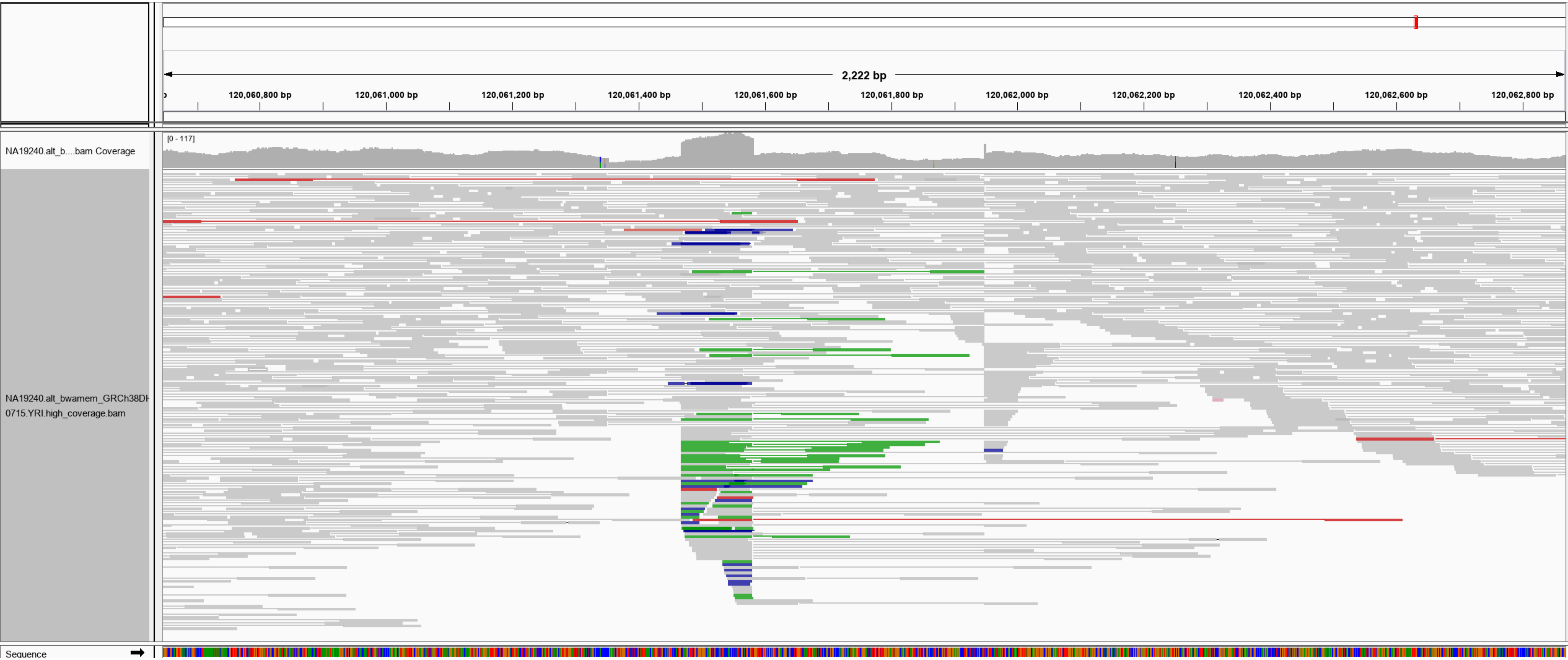

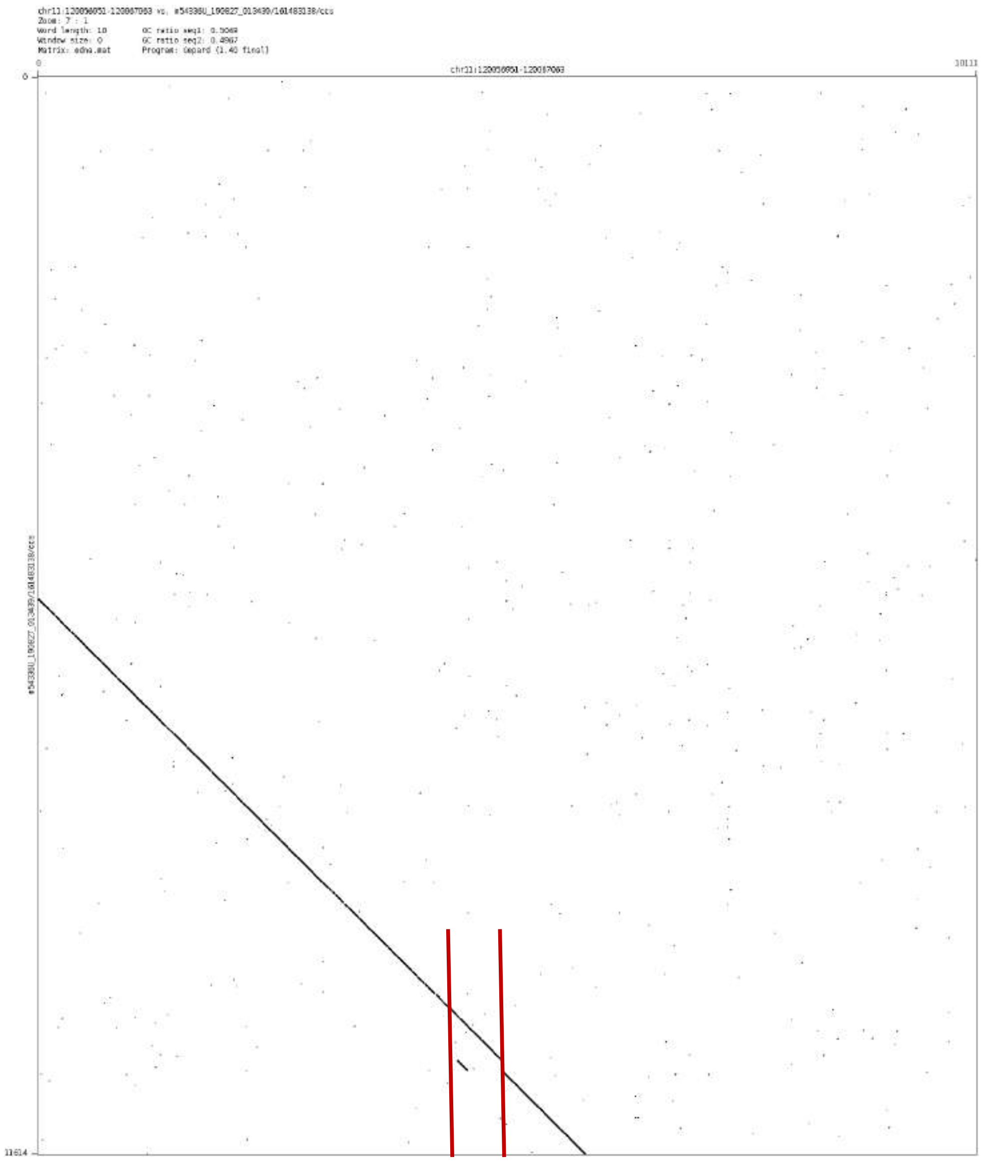

Zoom: 7 : 1
Word length: 10
Window size: 0
Matrix: edna.mat
Program: Gepard (1.40 final)
0
11614

chr11:129143073-129145383

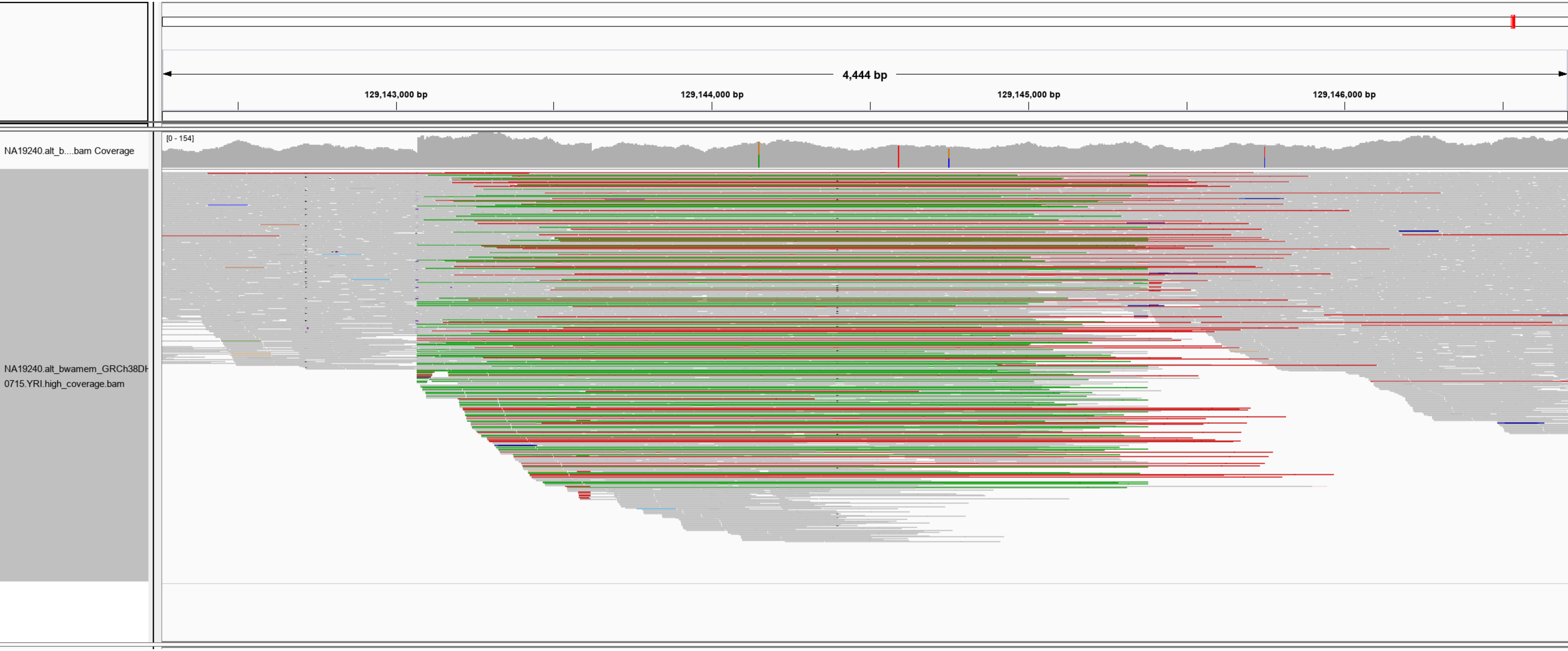

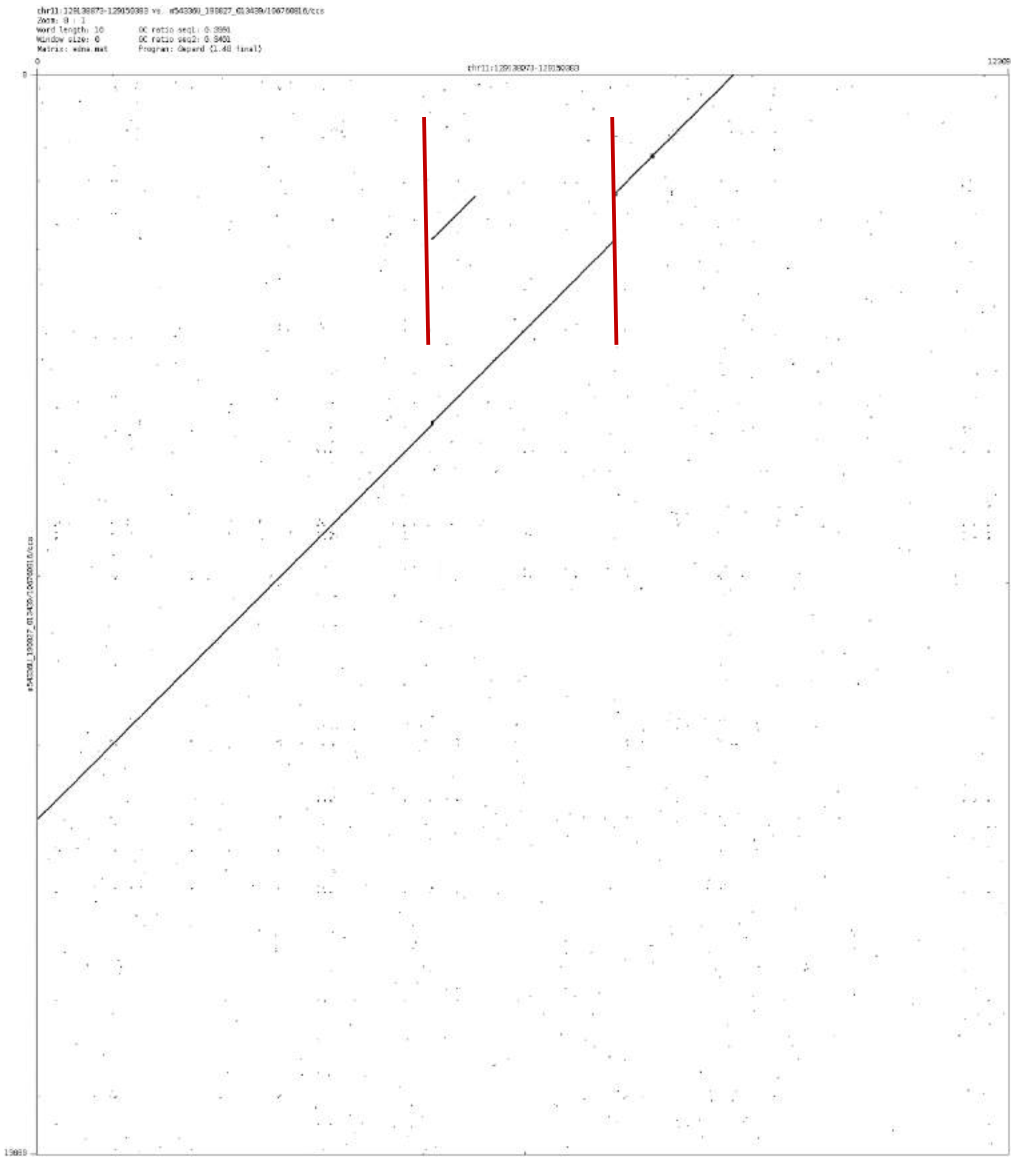

Word length: 10
Window size: 0
Matrix: edna.mat
GC ratio seq1:
GC ratio seq2:

chr12:46634787-46639521

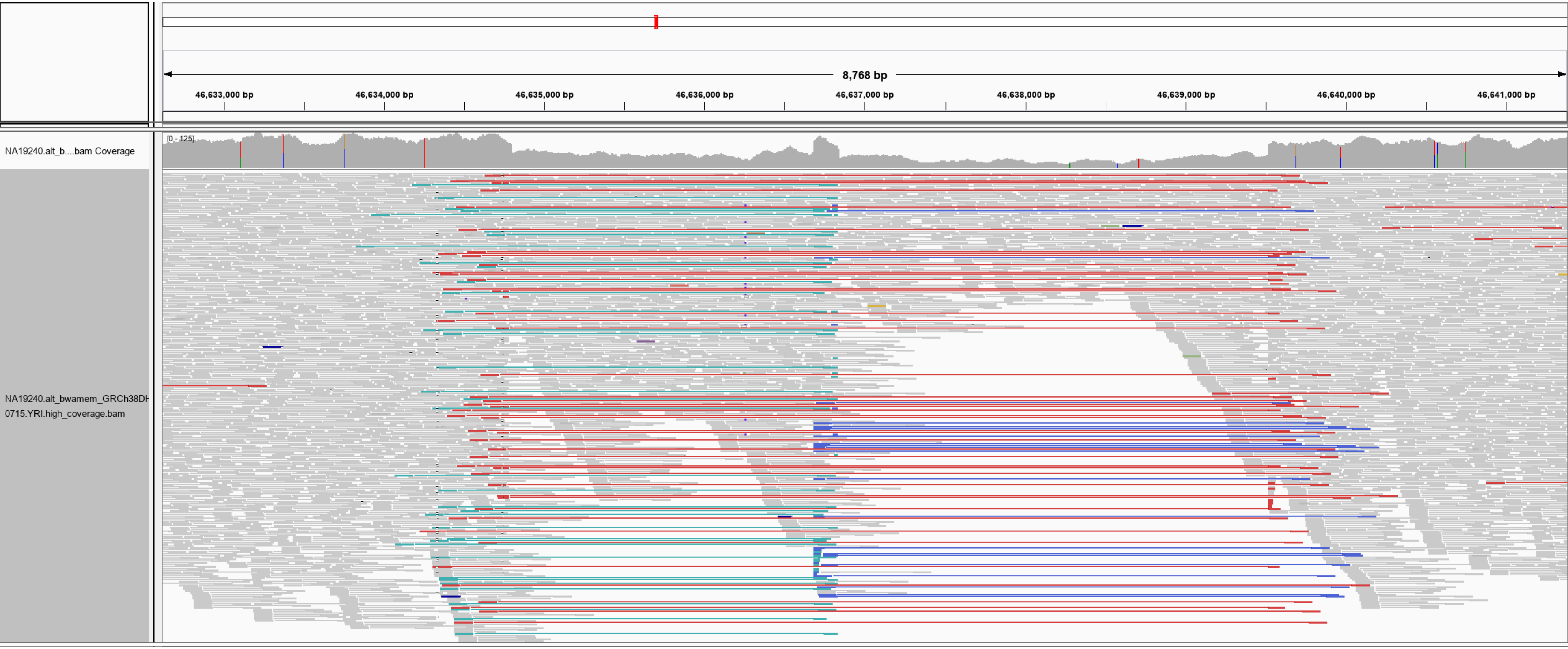

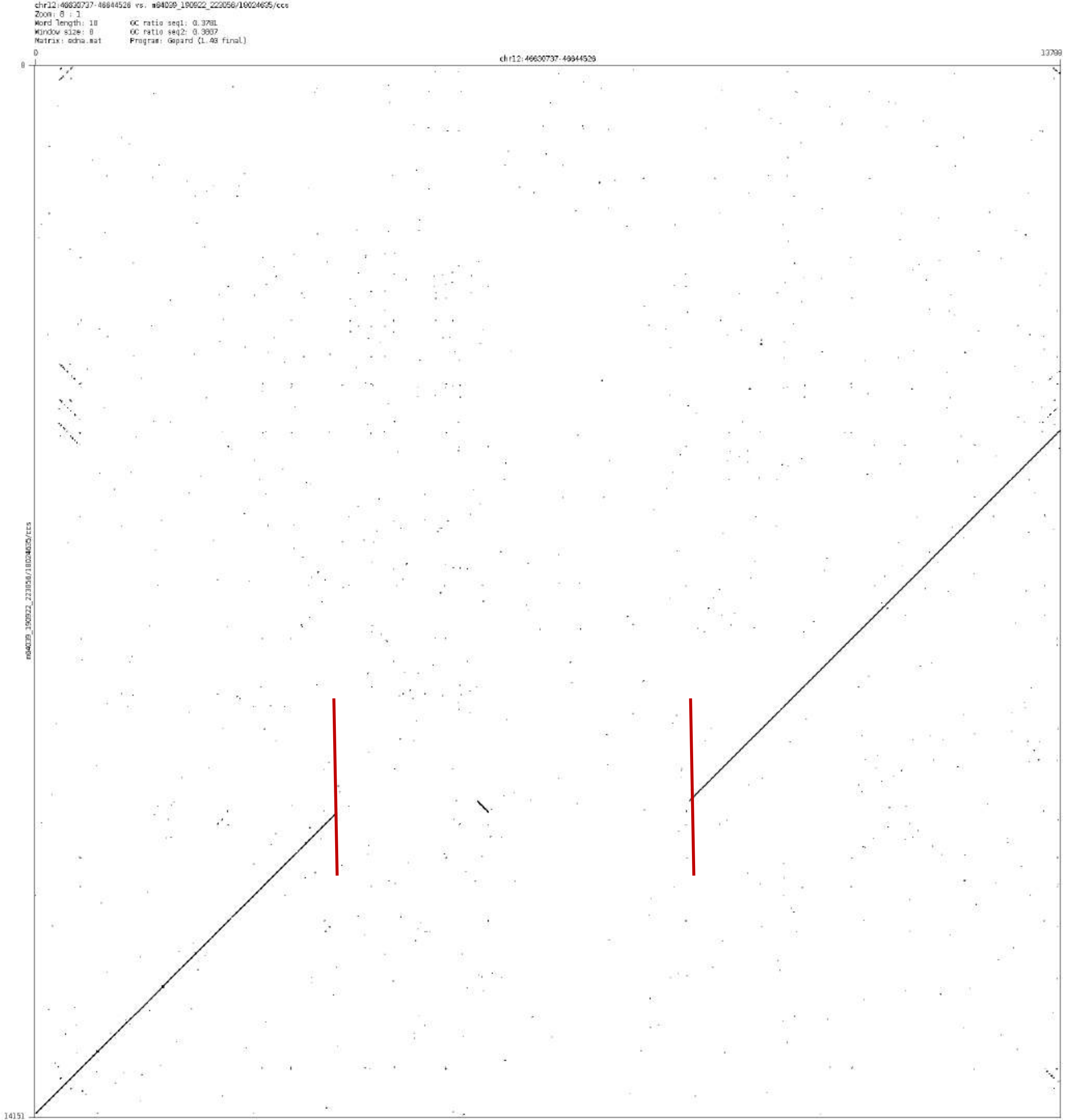
chr12:46830737-46844526 vs. m64039_190922_223056/10024635/ccs
Zoom: 8 : 1
Word length: 10
Window size: 0
Matrix: edna.mat
GC ratio seq1: 0.3781
GC ratio seq2: 0.3807
Program: Gepard (1.40 final)
0
chr12:46830737-46844526
13789
0
m64039_190922_223056/10024635/ccs
14151

chr12:71139013-71139972

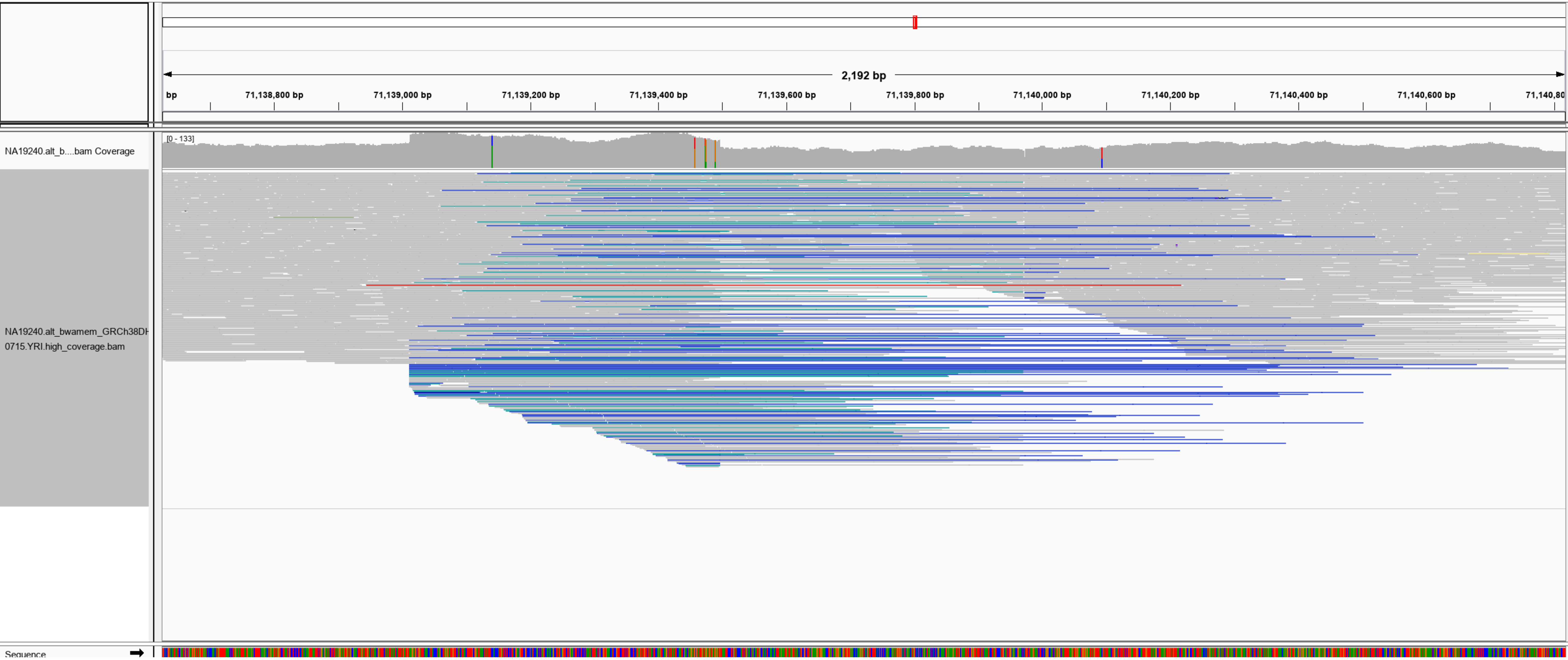

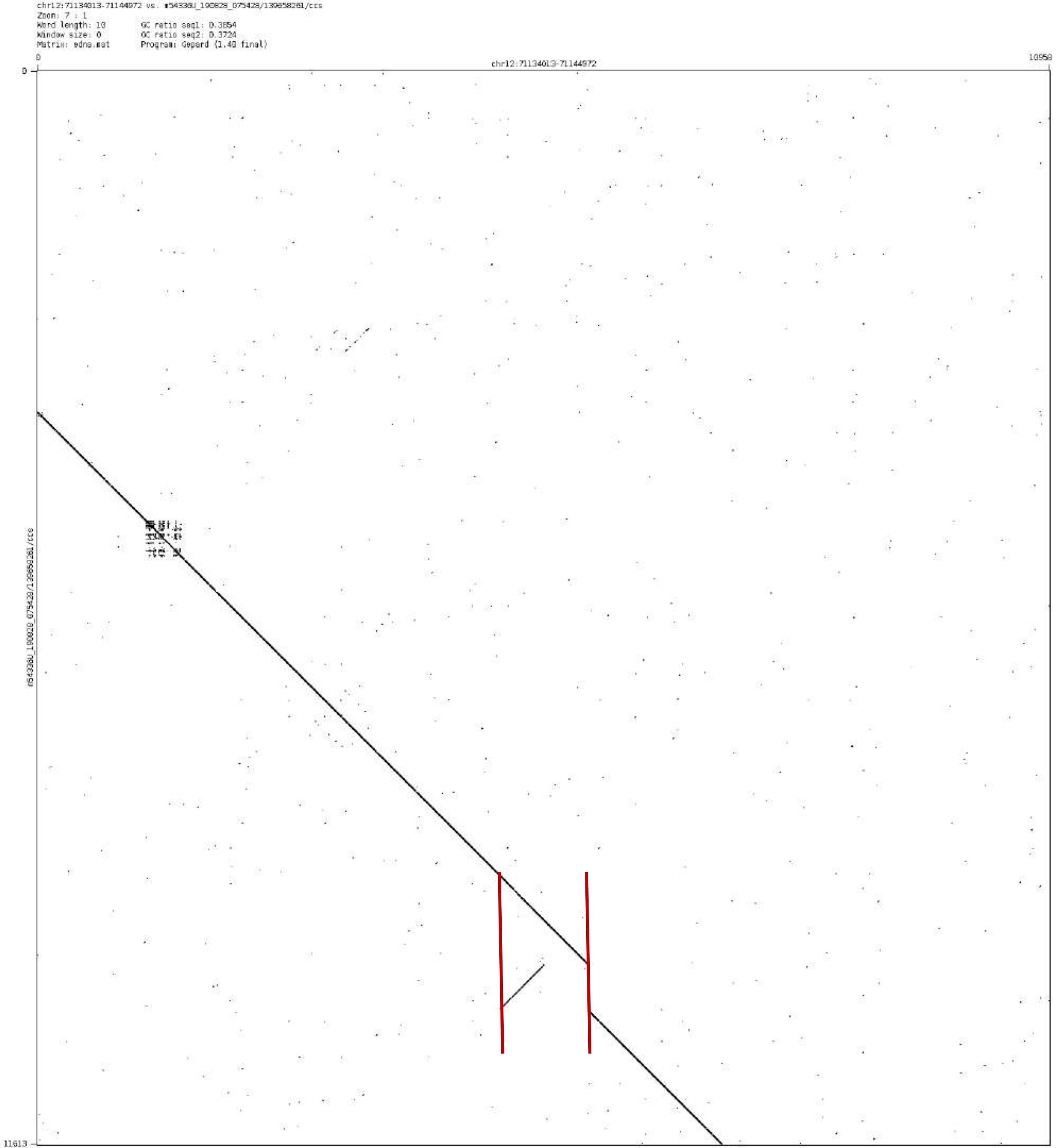

chr12:71134013-71144972 vs. m54338U_190828_075428/139658261/ccs
Zoom: 7 : 1
Word length: 10
Window size: 0
Matrix: edna.mat
GC ratio seq1: 0.3854
GC ratio seq2: 0.3724
Program: Gepard (1.40 final)
chr12:71134013-71144972
0
10958
11613

chr12:77990108-77994493

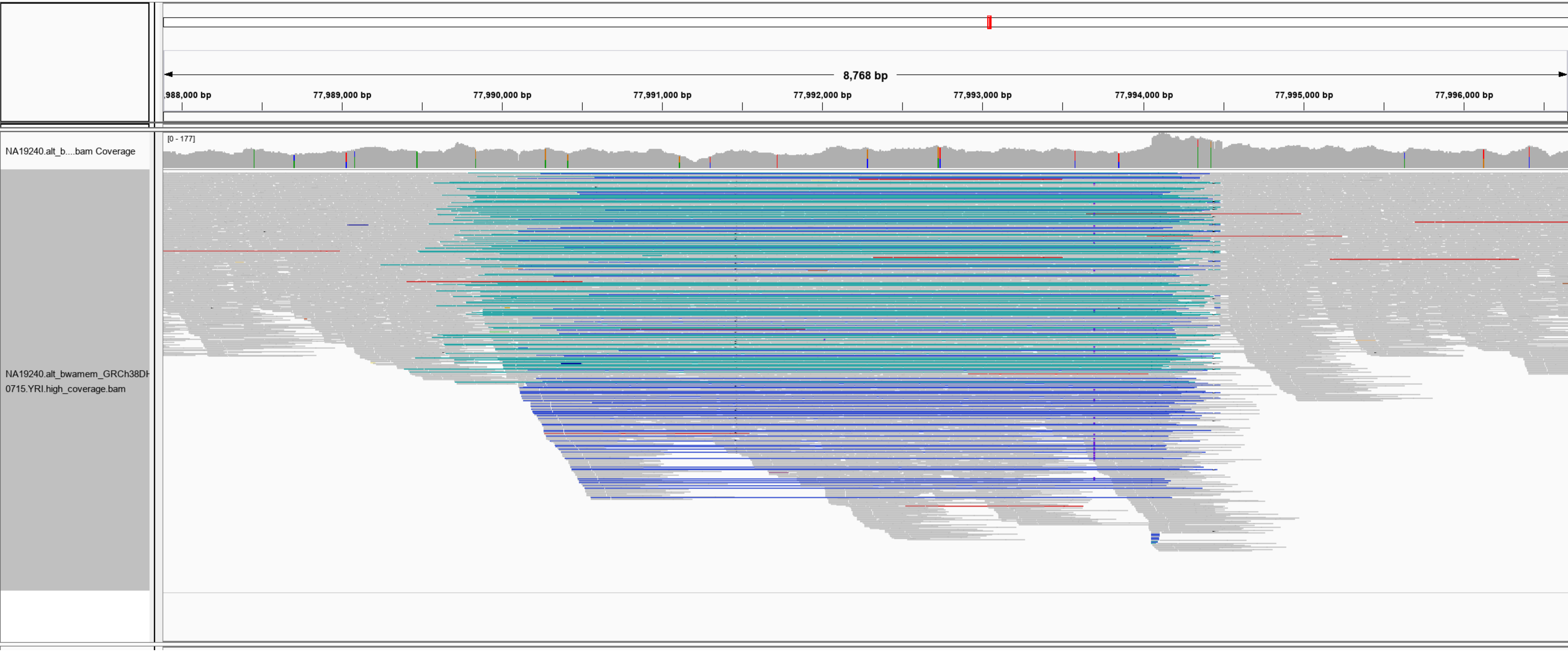

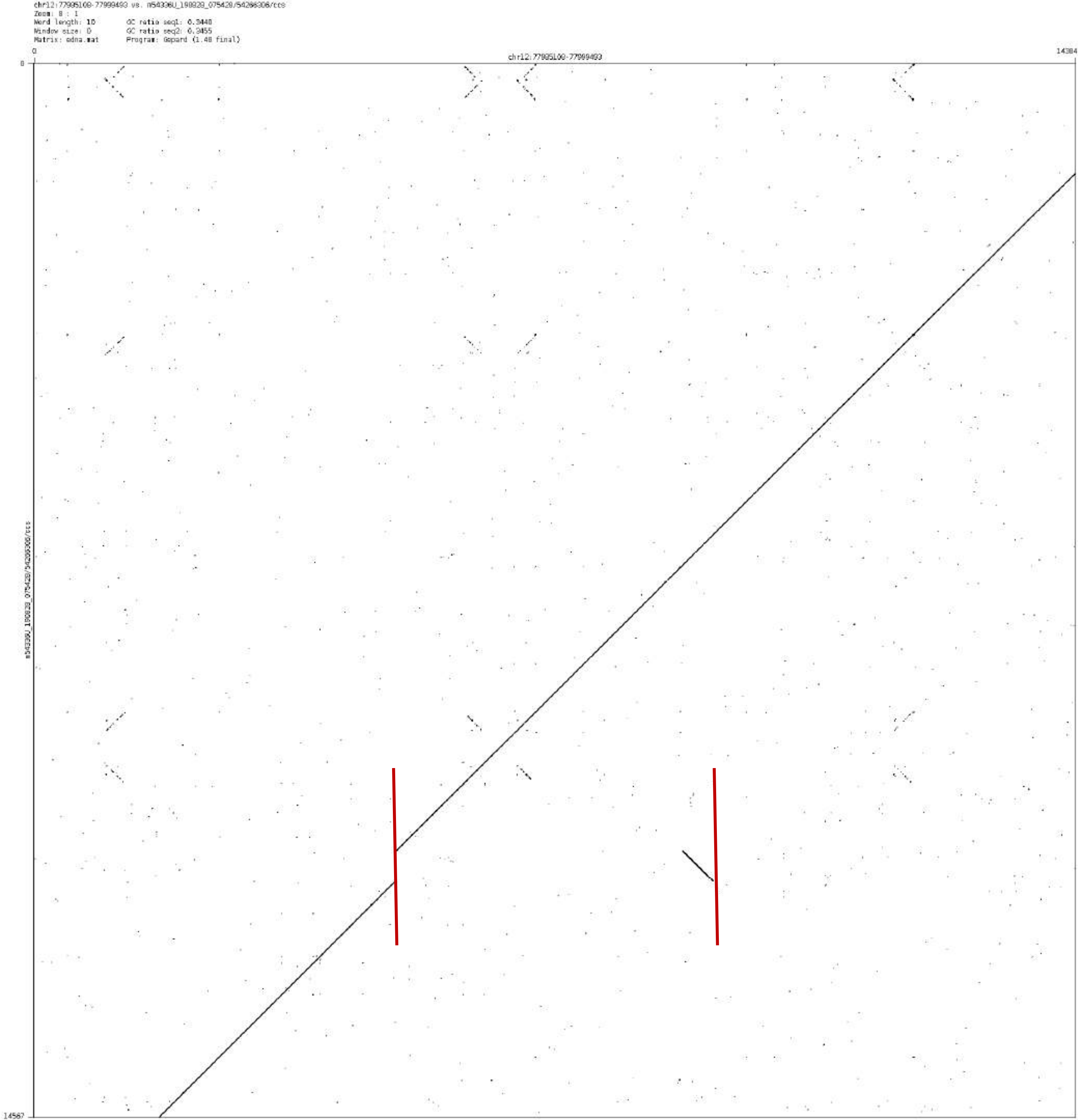
chr12:77985100-77999493 vs. m54336U_190829_075420/54206306/ccs
Zoom: 8 : 1
Word length: 10
Window size: 0
Matrix: edna.mat
GC ratio seq1: 0.3448
GC ratio seq2: 0.3455
Program: Gepard (1.40 final)
0
chr12:77985100-77999493
14384
0
m54336U_190829_075420/54206306/ccs
14567

chr12:92745382-92748451

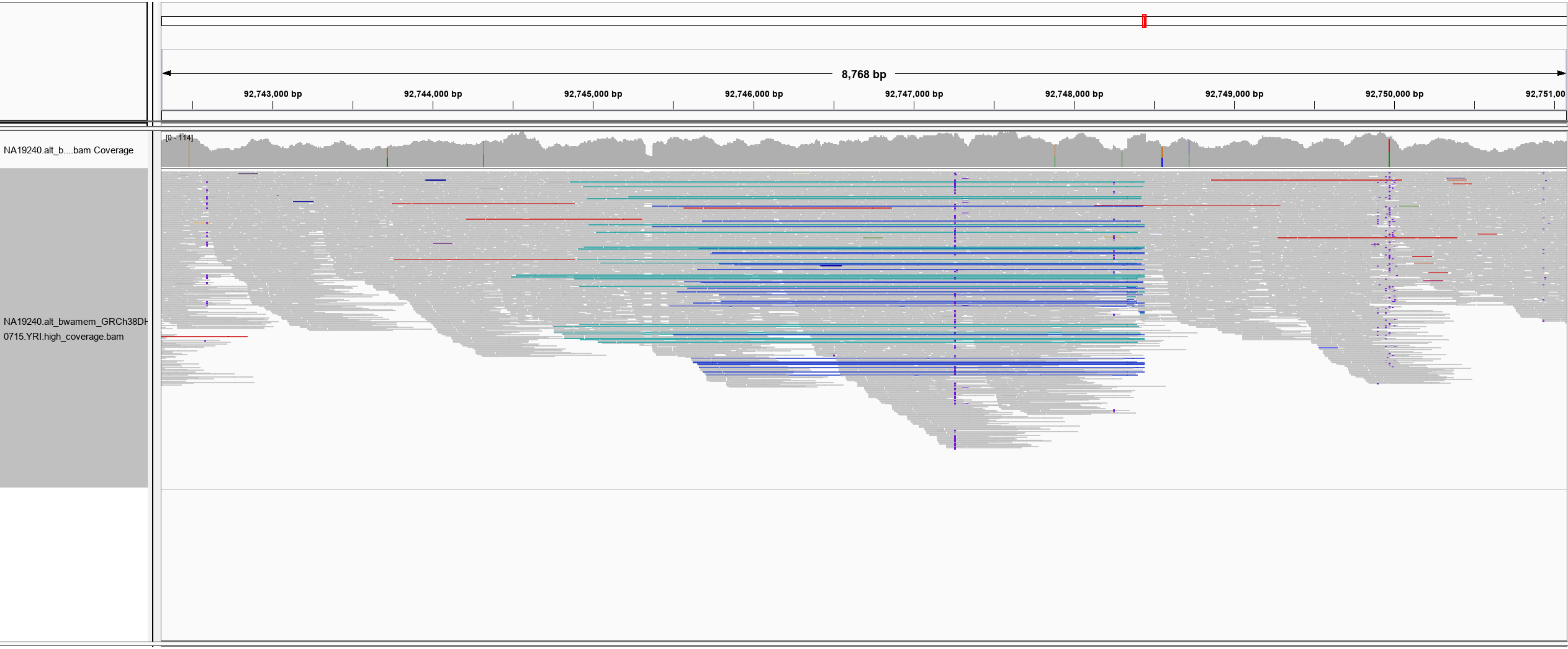

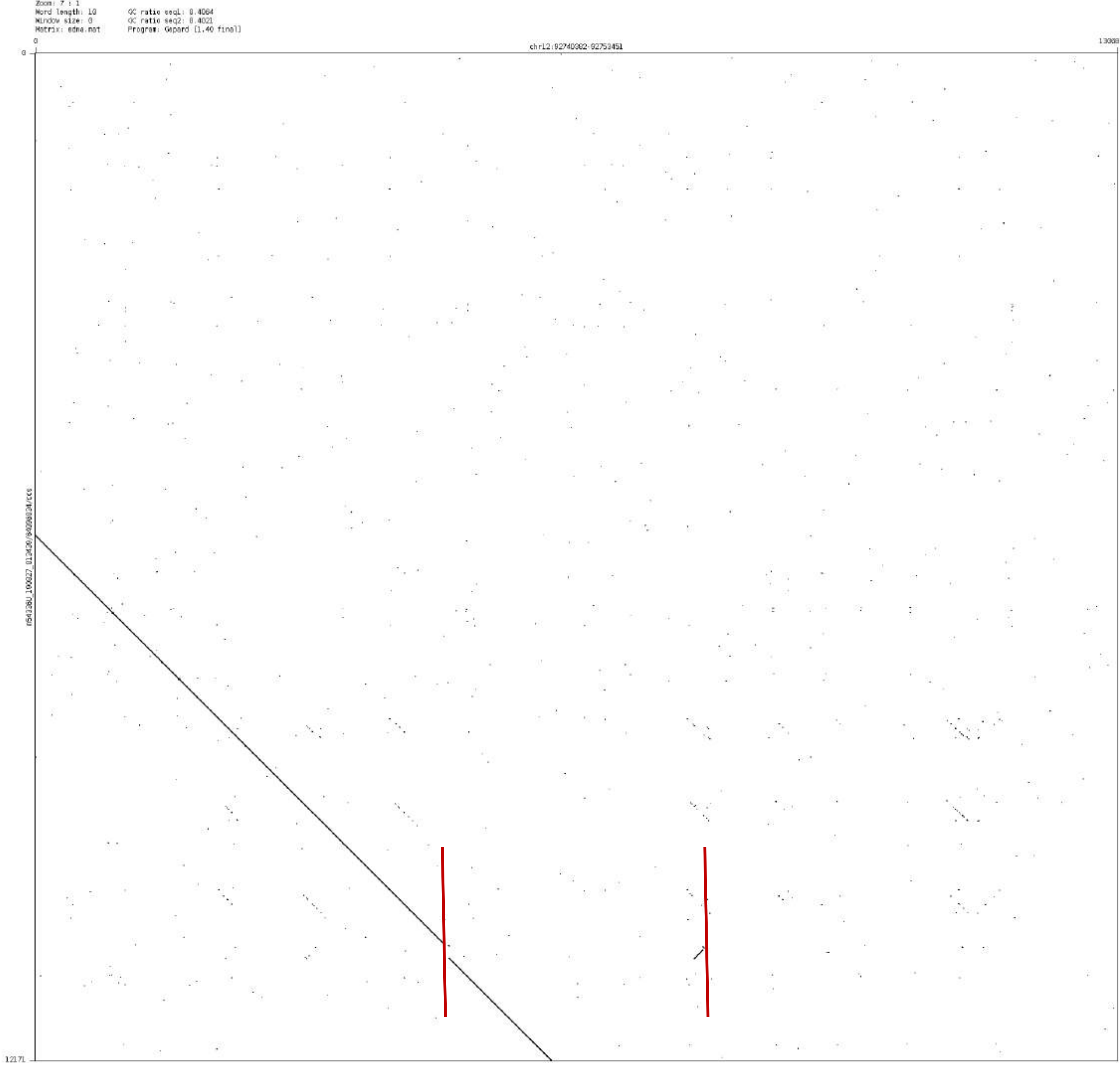
Zoom: 7 : 1
Word length: 10
Window size: 0
Matrix: edna.mat
GC ratio seq1: 0.4064
GC ratio seq2: 0.4021
Program: Gepard [1.40 final]
0
chr12:92740382-92753451
13088
12171

chr12:95558627-95559959

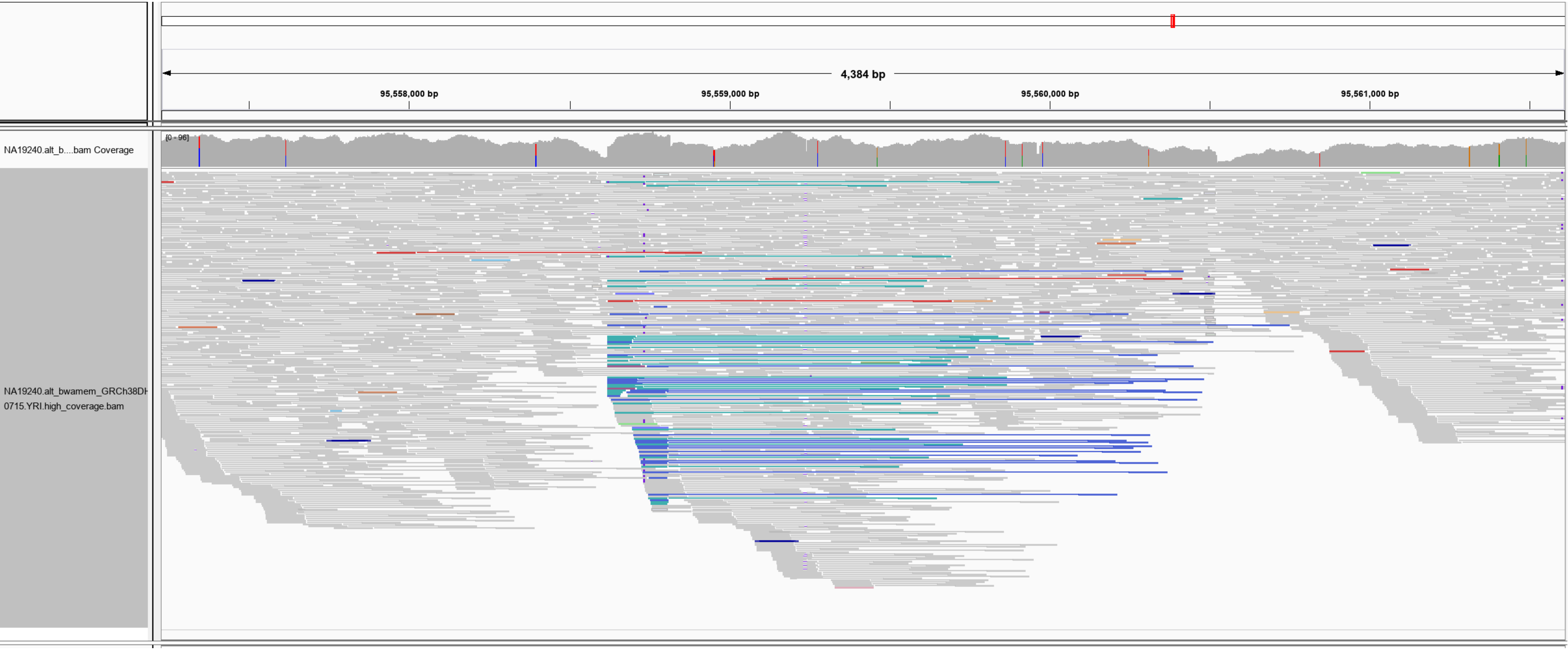

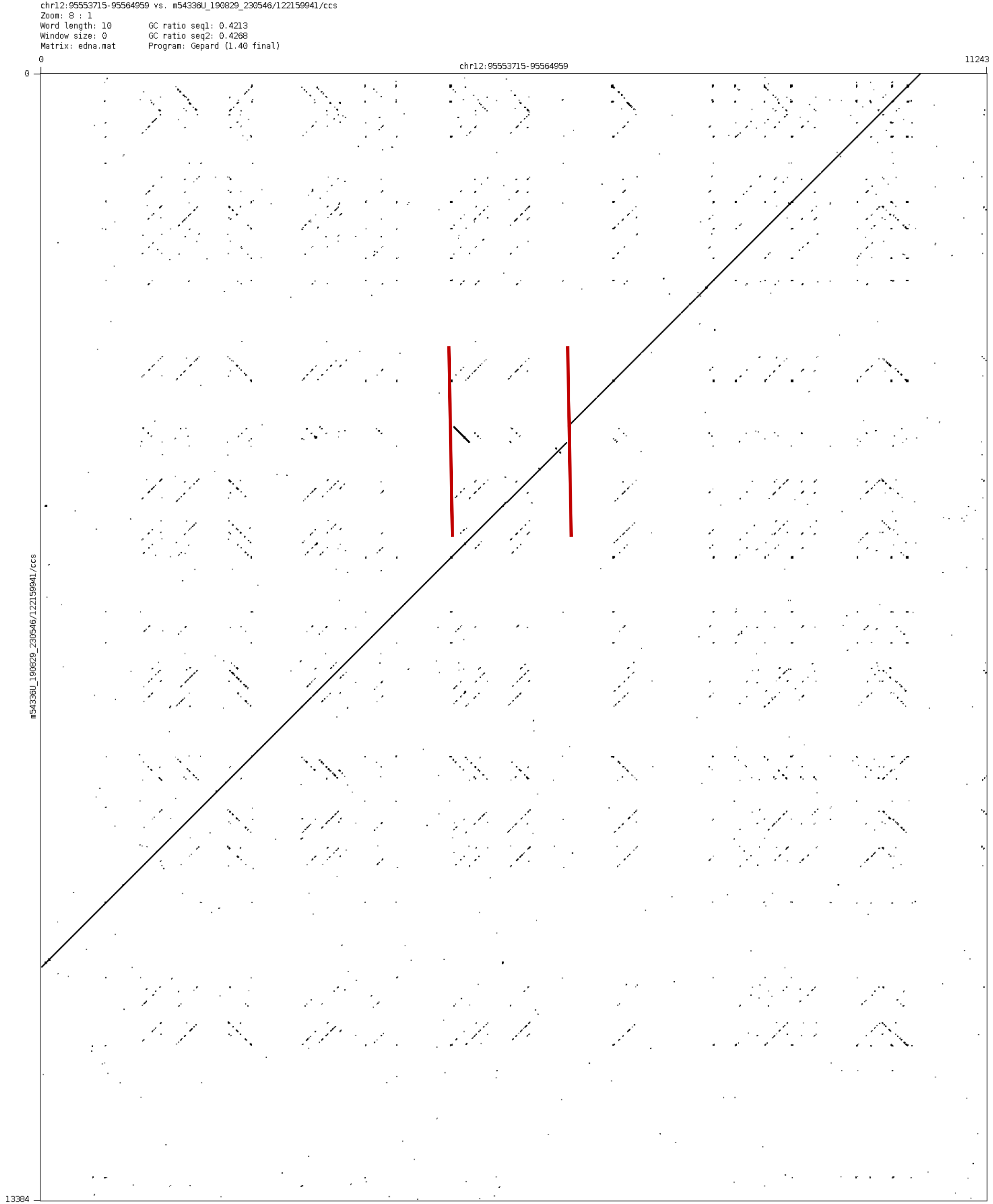
chr12:95553715-95564959 vs. m54336U_190829_230546/122159941/ccs
Zoom: 8 : 1
Word length: 10
GC ratio seq1: 0.4213
Window size: 0
GC ratio seq2: 0.4268
Matrix: edna.mat
Program: Gepard (1.40 final)
0
chr12:95553715-95564959
11243
0
m54336U_190829_230546/122159941/ccs
13384

chr14:20082944-20087060

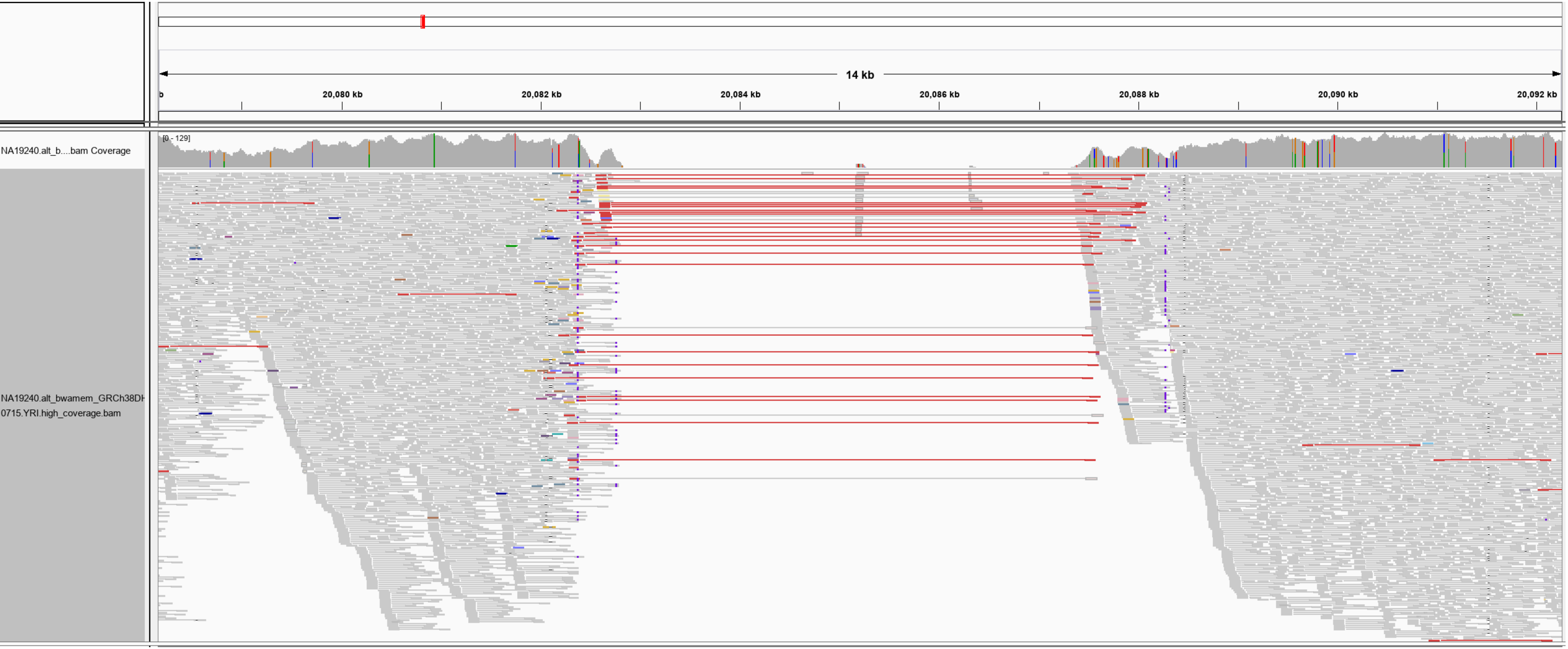

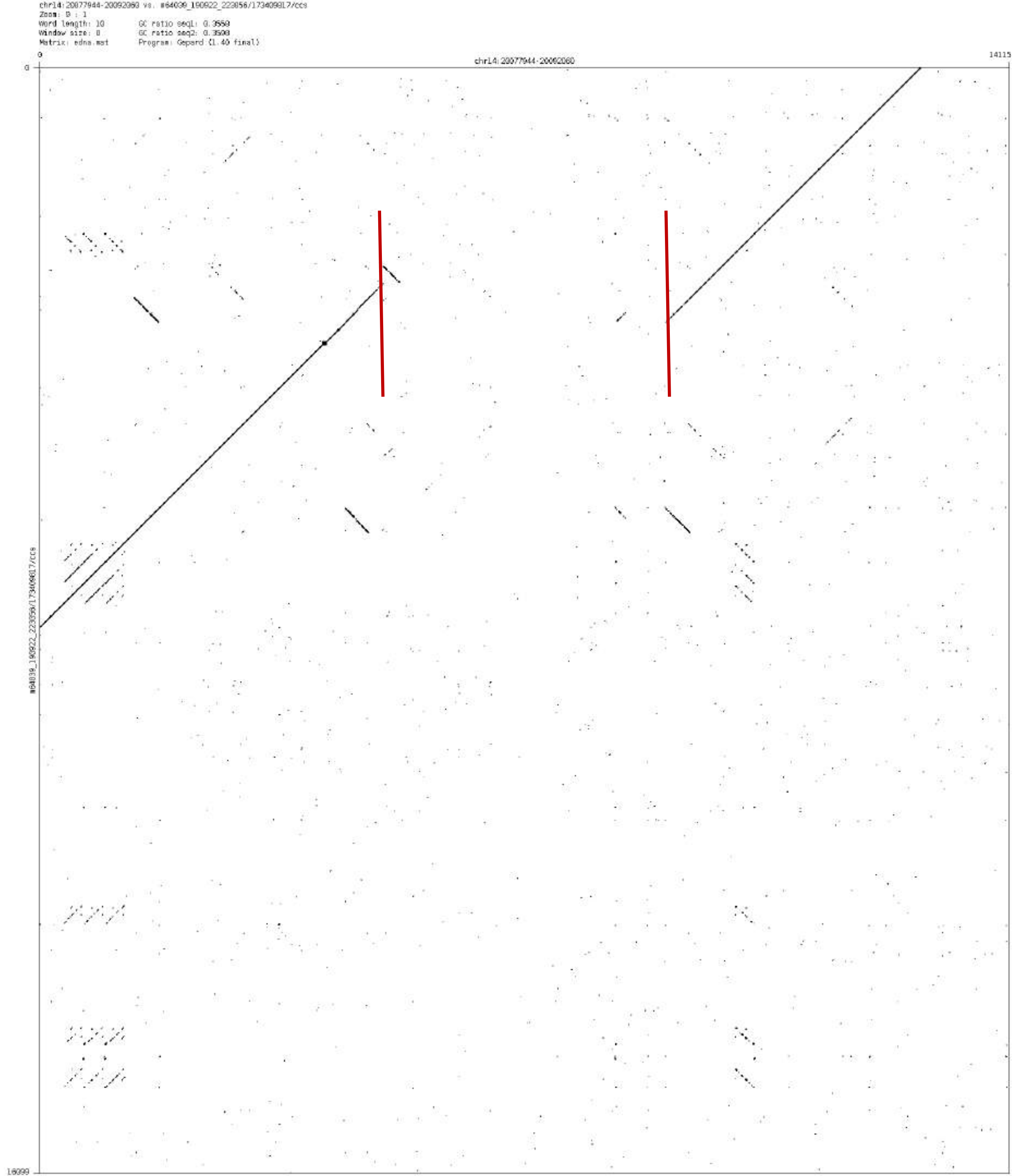
chr14:20077944-20092060 vs. m64039_190922_223056/173409817/ccs
Zoom: 0 : 1
Word length: 10
Window size: 0
Matrix: edna.mat
GC ratio seq1: 0.3550
GC ratio seq2: 0.3500
Program: Gepard (1.40 final)
0
14115
chr14:20077944-20092060
m64039_190922_223056/173409817/ccs
16099

chr14:22636224-22640024

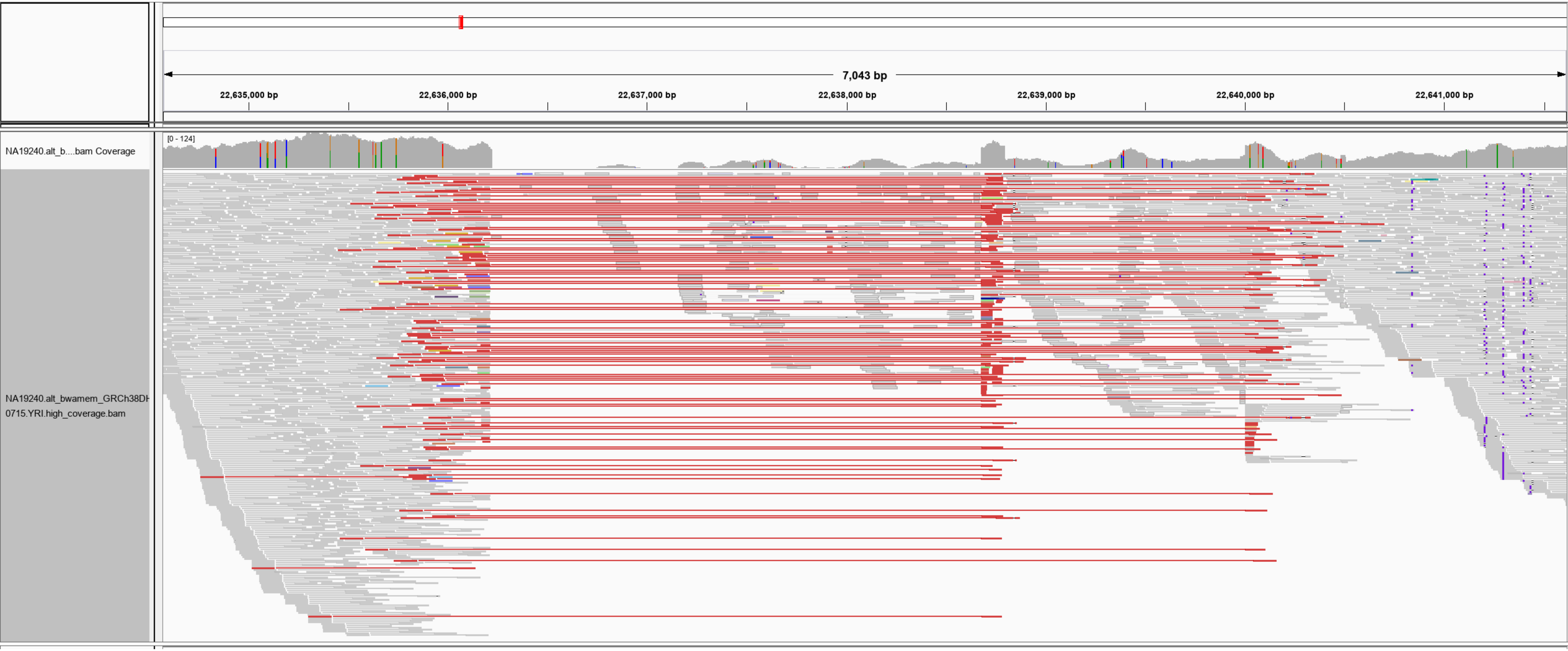

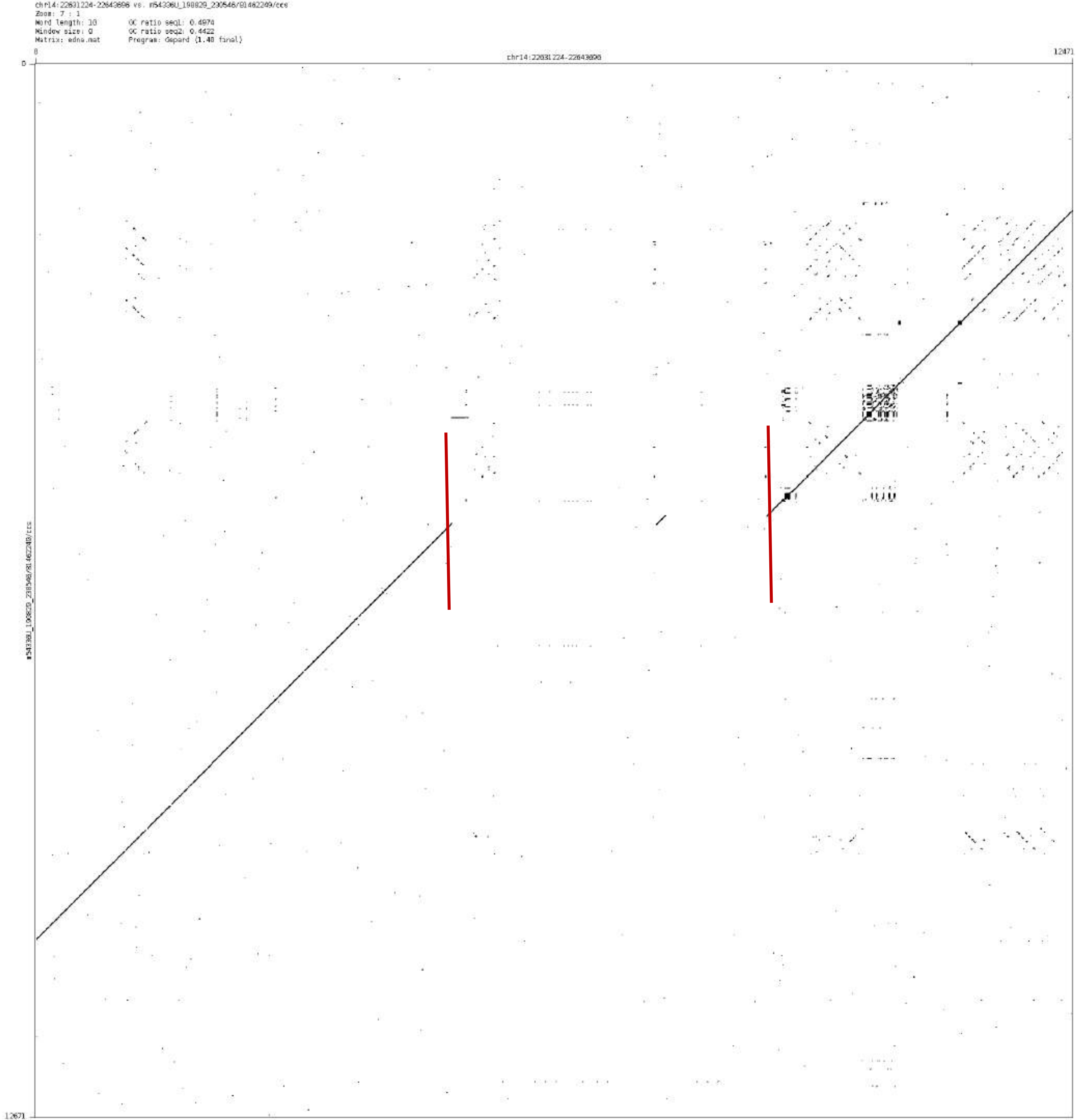
chr14:22631224-22643696 vs. m54336U_190829_230546/81462249/ccs
Zoom: 7 : 1
Word length: 10
Window size: 0
Matrix: edna.mat
GC ratio seq1: 0.4974
GC ratio seq2: 0.4422
Program: Gepard (1.40 final)
0
12471
chr14:22631224-22643696
0
12671

chr14:65375854-65376595

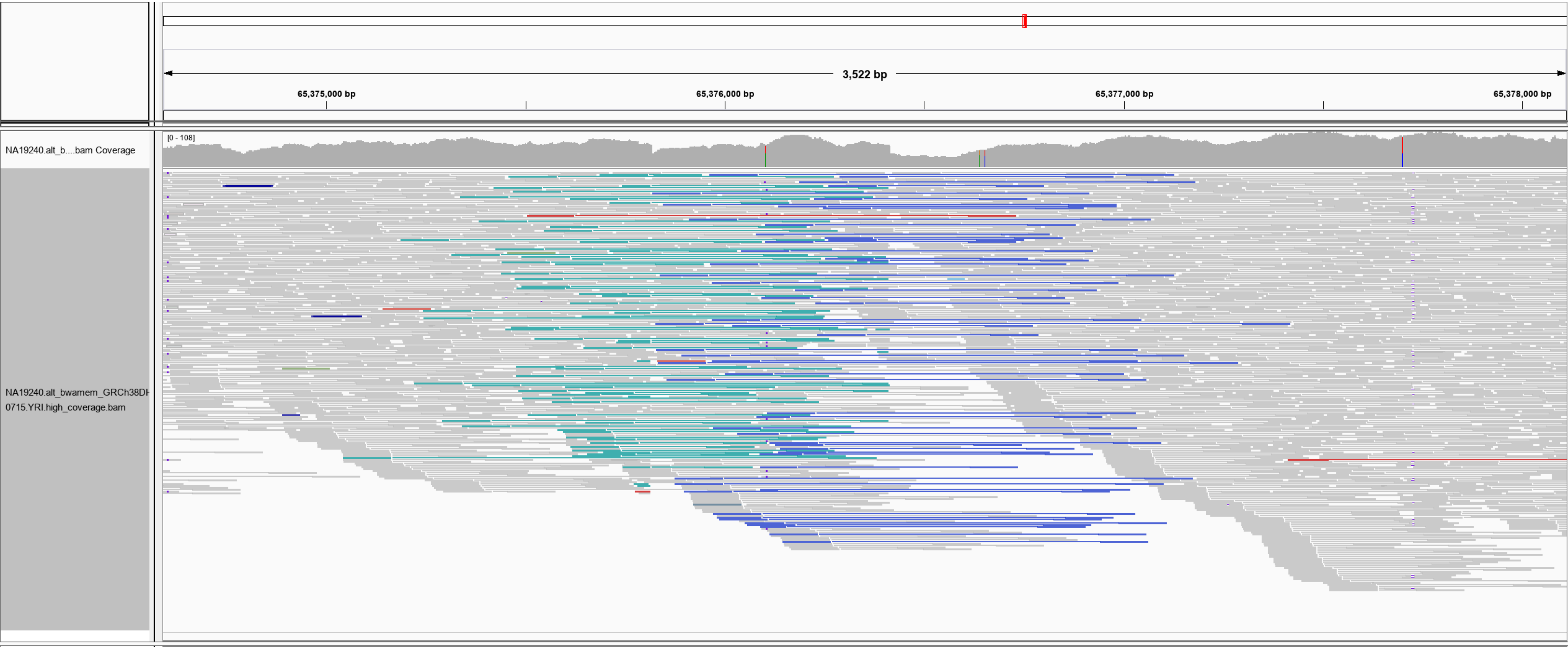

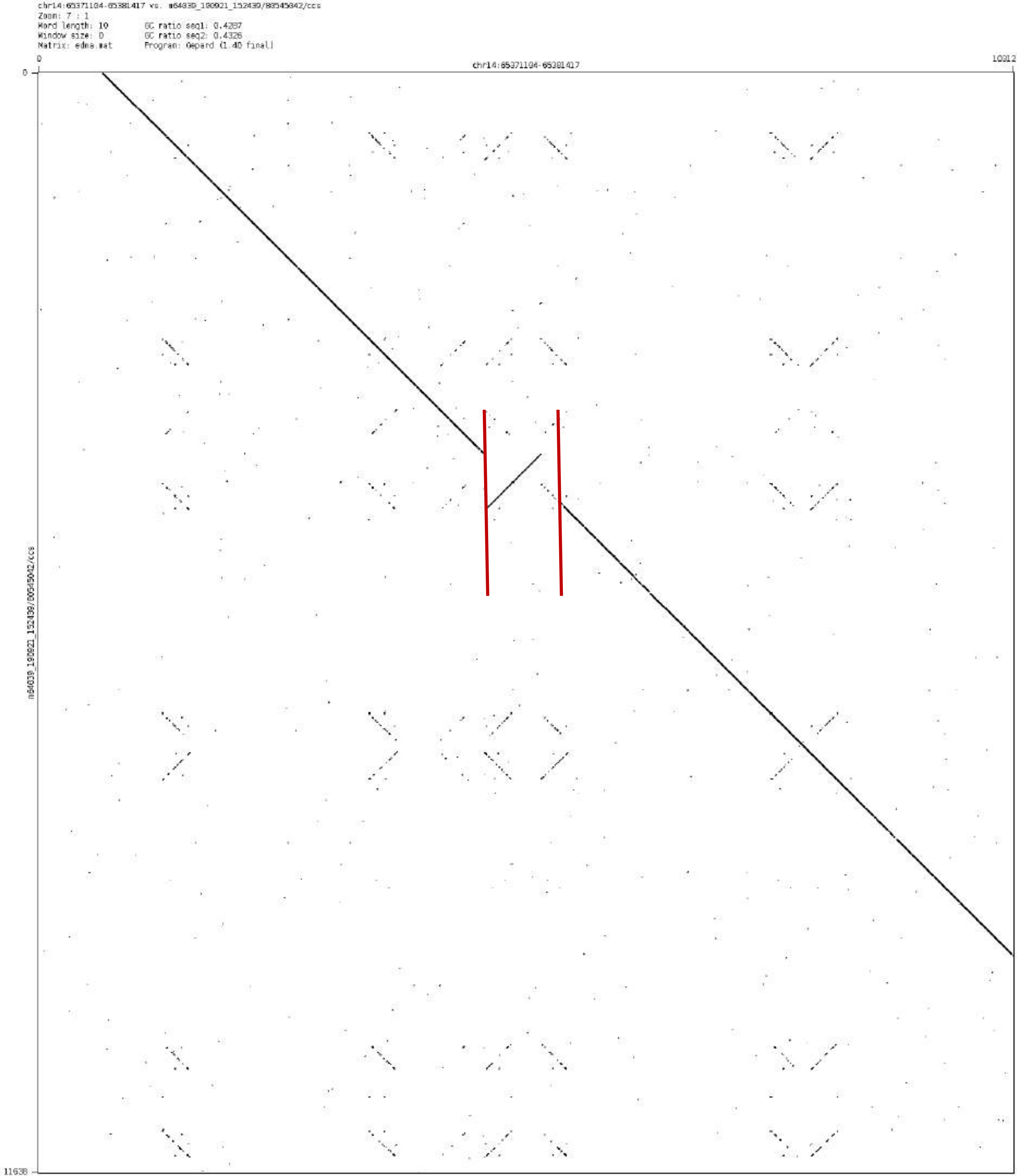

chr14:65371104-65381417 vs. m64039_180921_152439/80545342/ccs
Zoom: 7 : 1
Word length: 10
Window size: 0
Matrix: edna.mat
GC ratio seq1: 0.4287
GC ratio seq2: 0.4326
Program: Gepard (1.40 final)
0
chr14:65371104-65381417
10312
0
11638

chr14:66703613-66705301

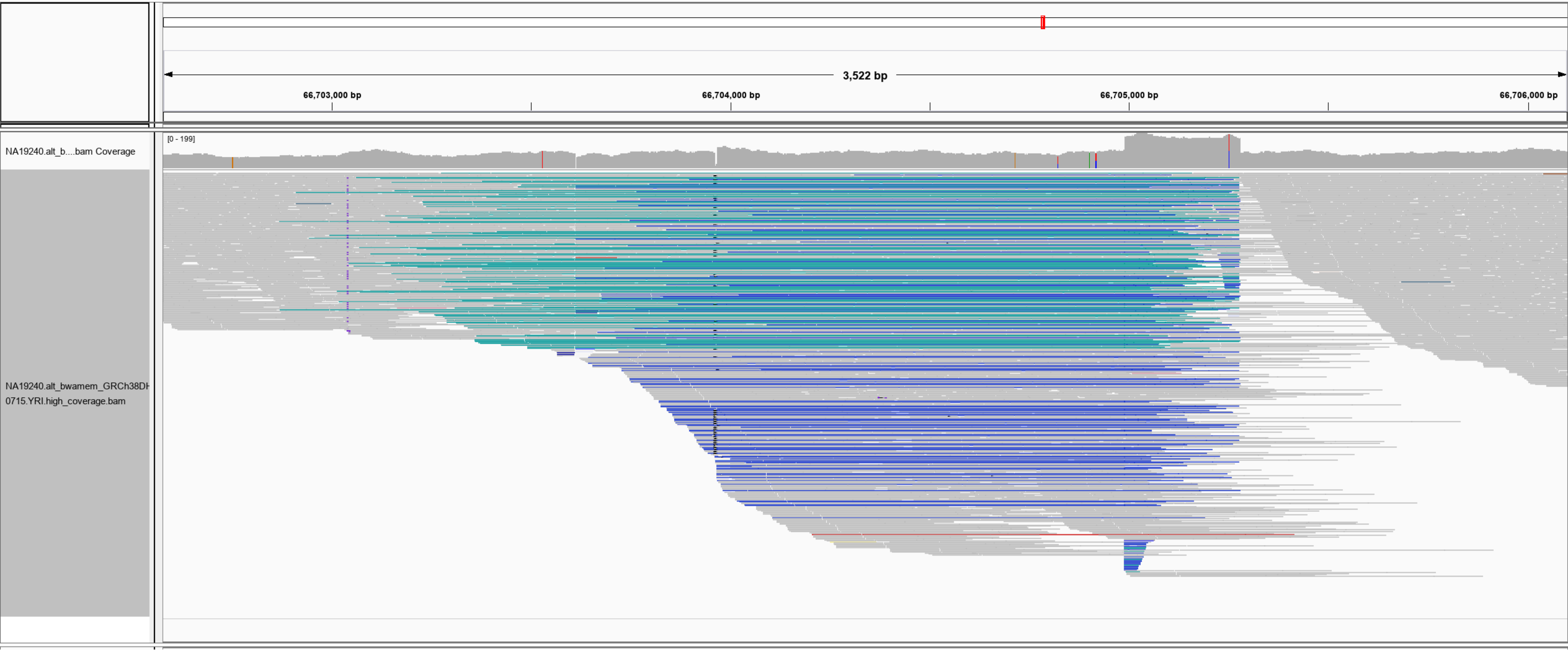

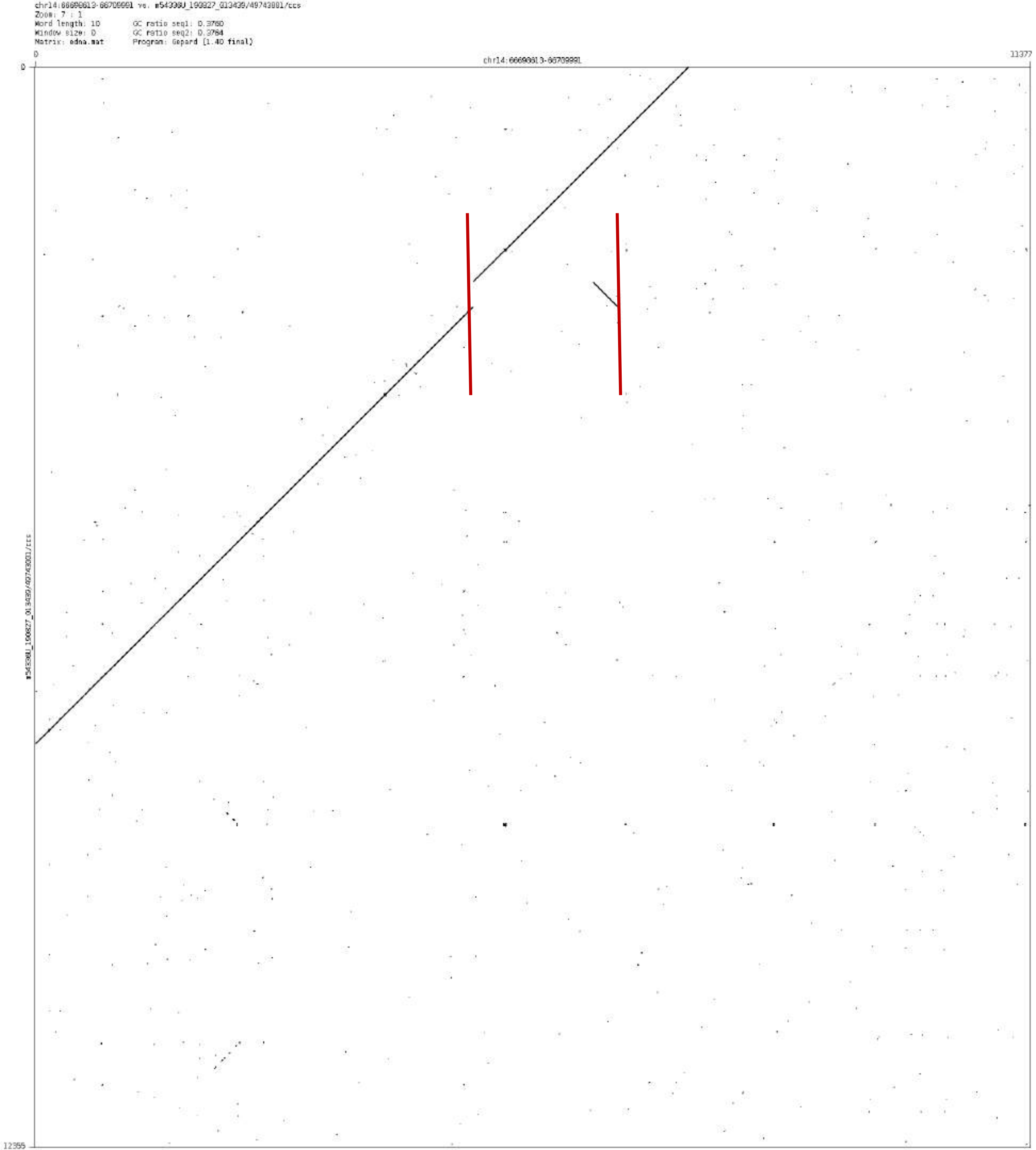

chr14:66698613-66709991 vs. m54330U_190827_013439/49743081/ccs
Zoom: 7 : 1
Word length: 10
Window size: 0
Matrix: edna.mat
GC ratio seq1: 0.3760
GC ratio seq2: 0.3764
Program: Gepard (1.40 final)
0
chr14:66698613-66709991
11377
0
m54330U_190827_013439/49743081/ccs
12355

chr15:95148441-95149540

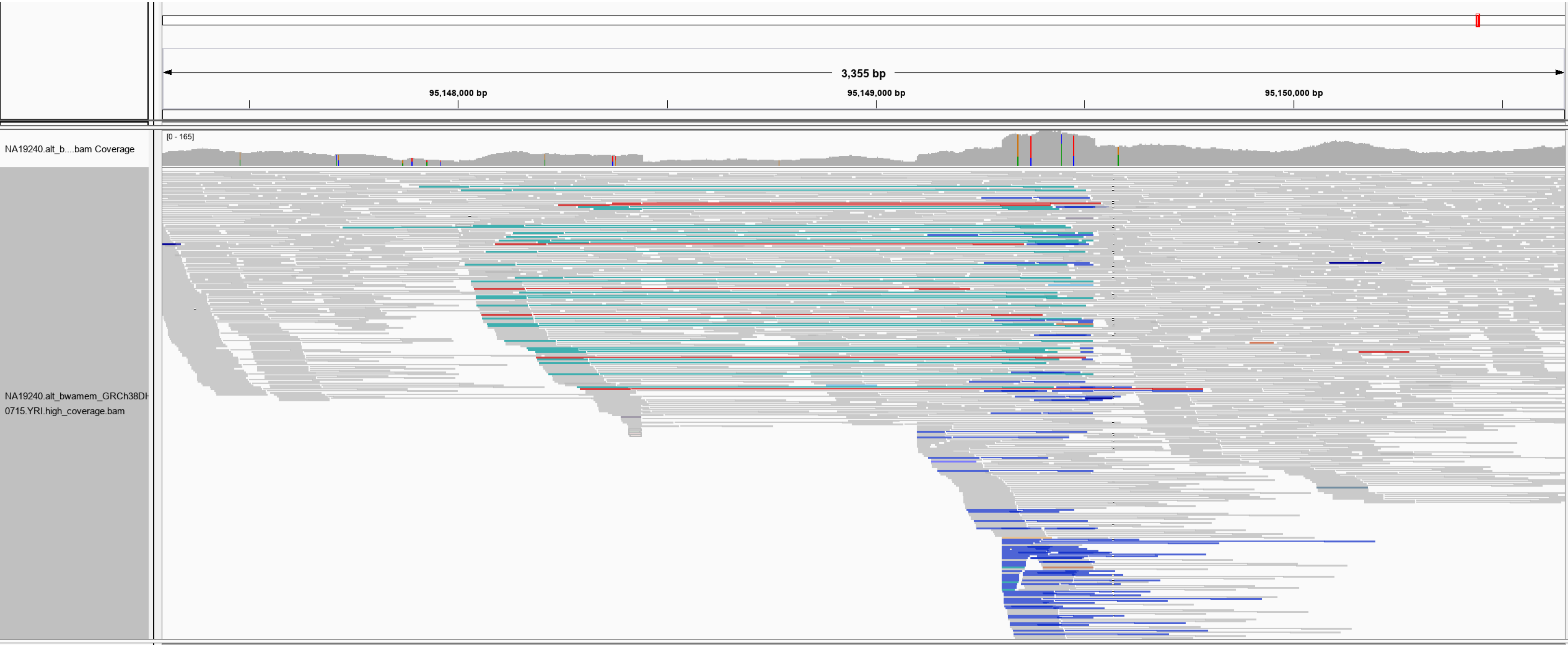

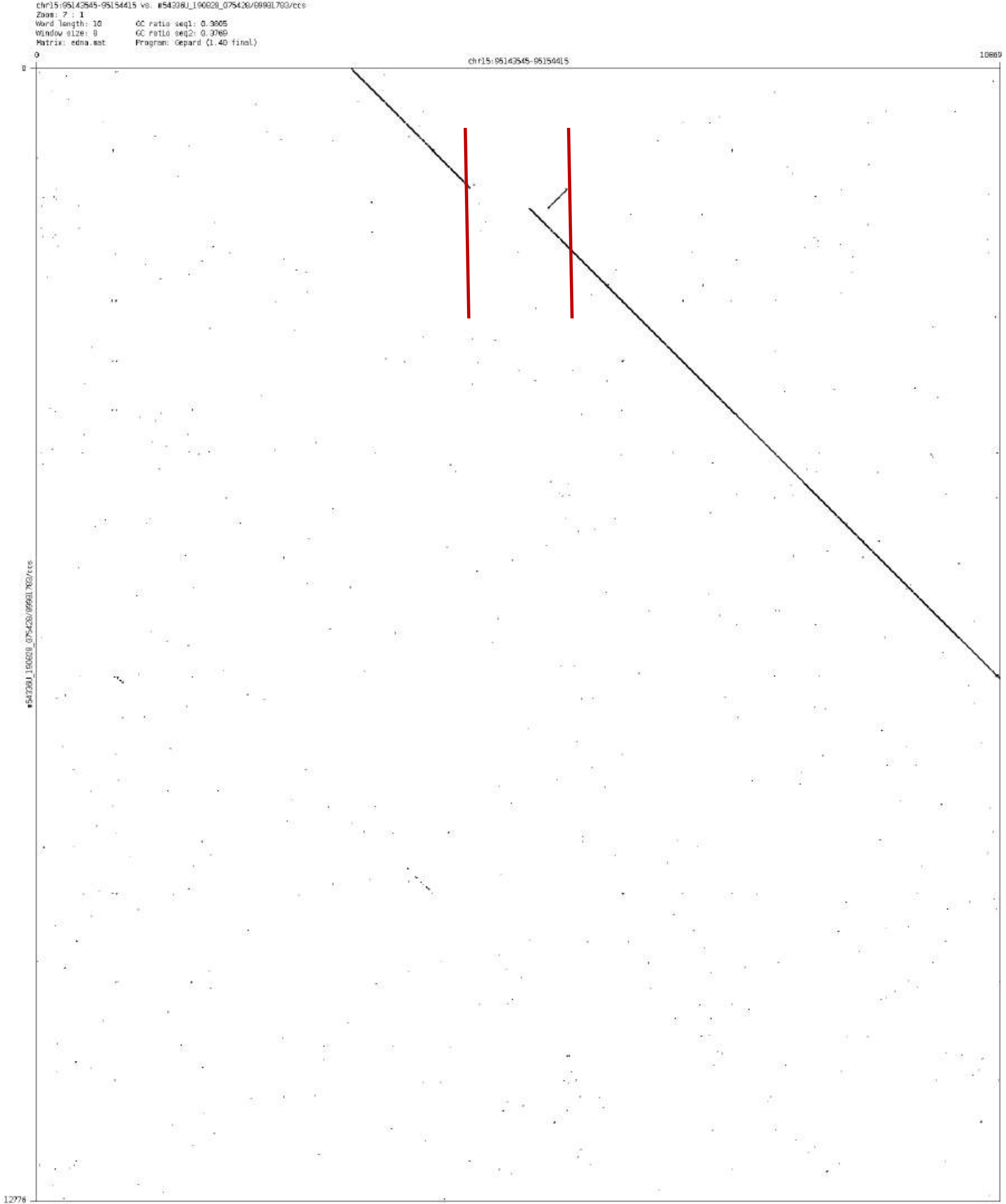
chr15:95143545-95154415 vs. m54338U_190828_075428/80881783/ccs
Zoom: 7 : 1
Word length: 10
Window size: 0
Matrix: edna.mat
GC ratio seq1: 0.3805
GC ratio seq2: 0.3769
Program: Gepard (1.40 final)
0
10869
chr15:95143545-95154415
0
12776

chr16:10676974-10677779

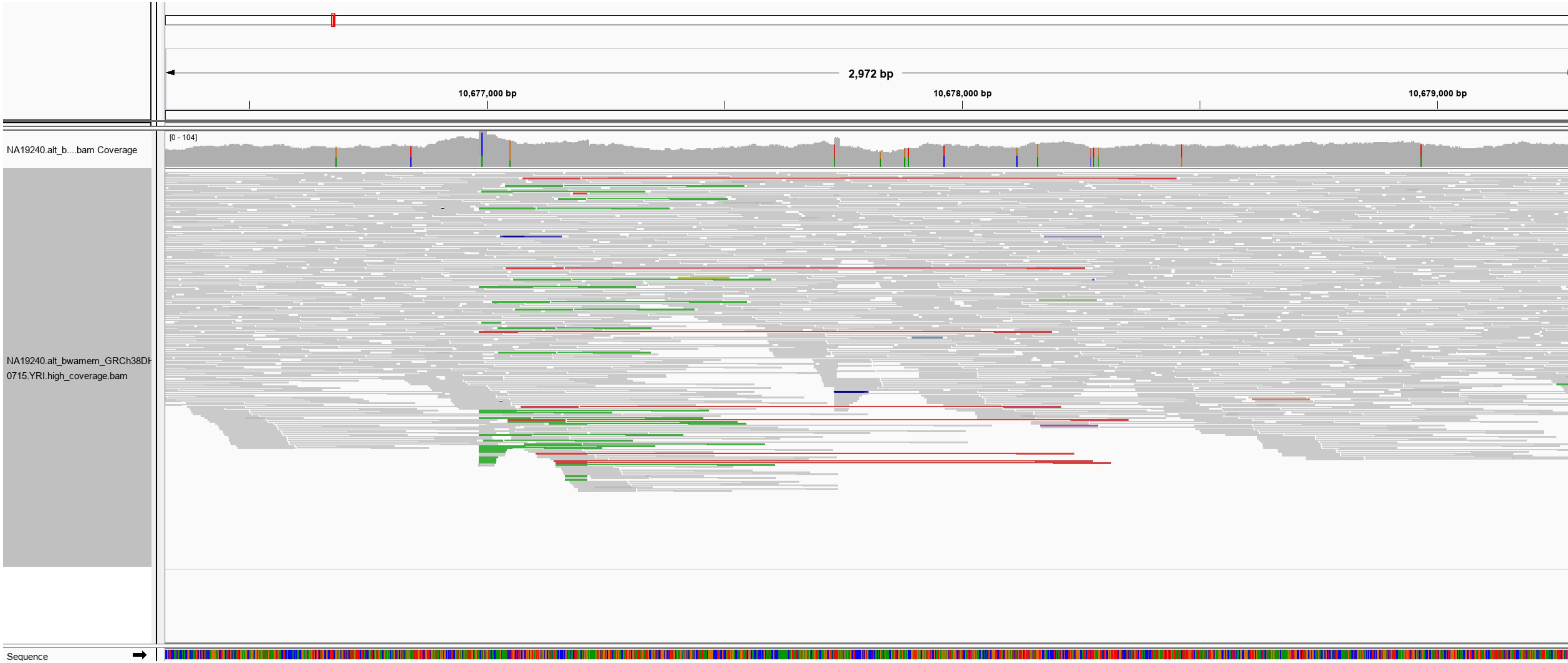

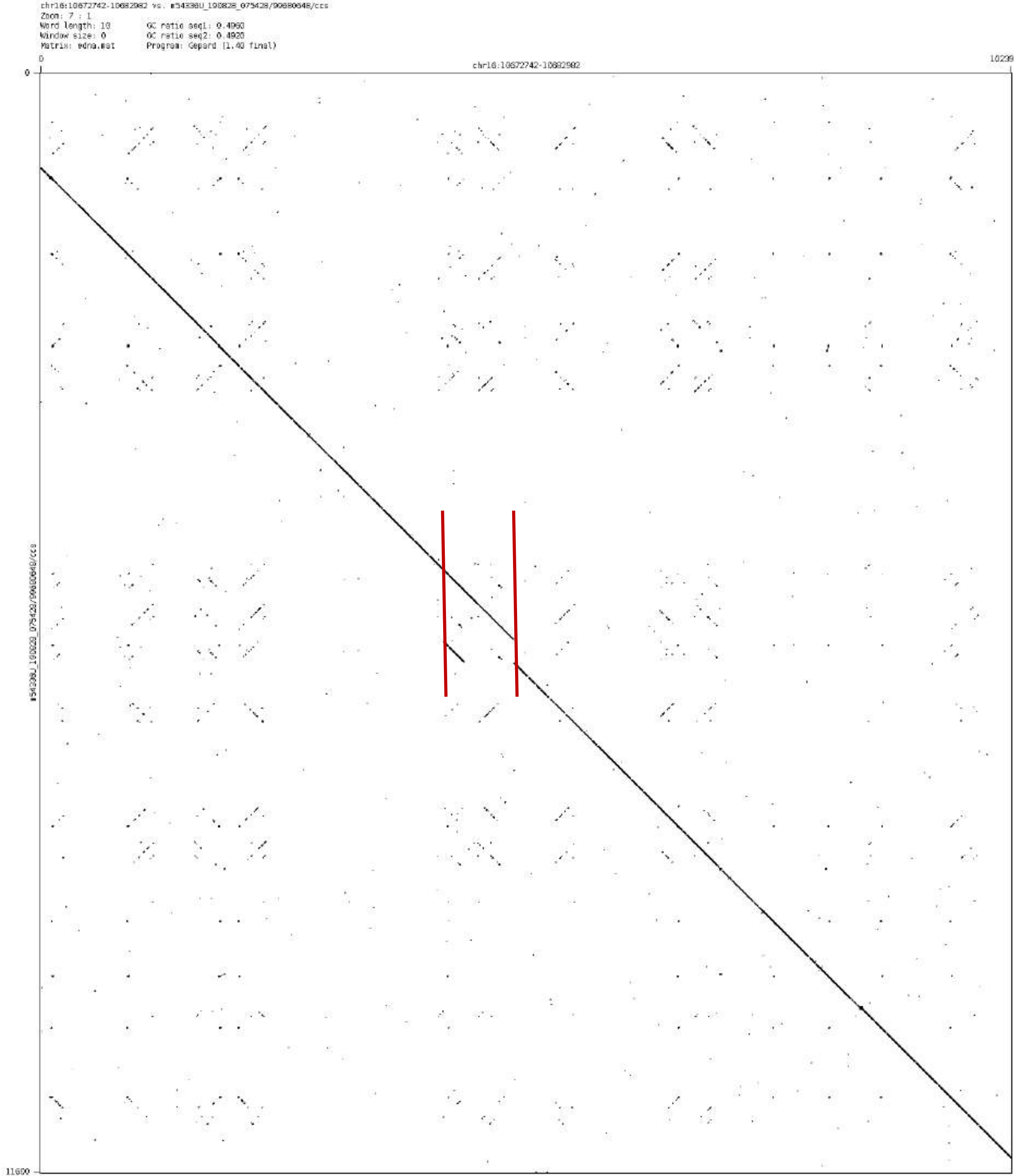
chr16:10672742-10682982 vs. m54338U_190828_075428/99680648/ccs
Zoom: 7 : 1
Word length: 10
Window size: 0
Matrix: edna.mat
GC ratio seq1: 0.4960
GC ratio seq2: 0.4920
Program: Gepard (1.40 final)
0
10239
chr16:10672742-10682982
0
11600

chr16:69727929-69728986

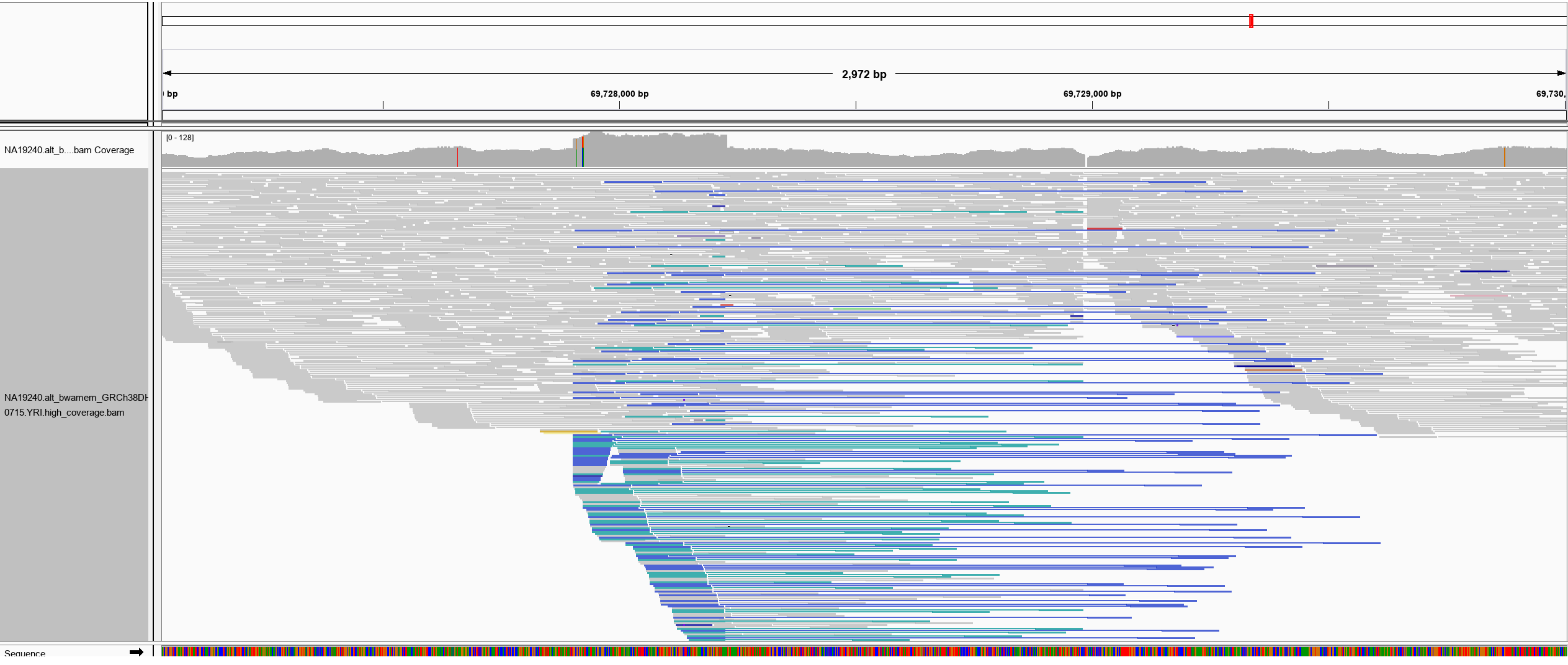

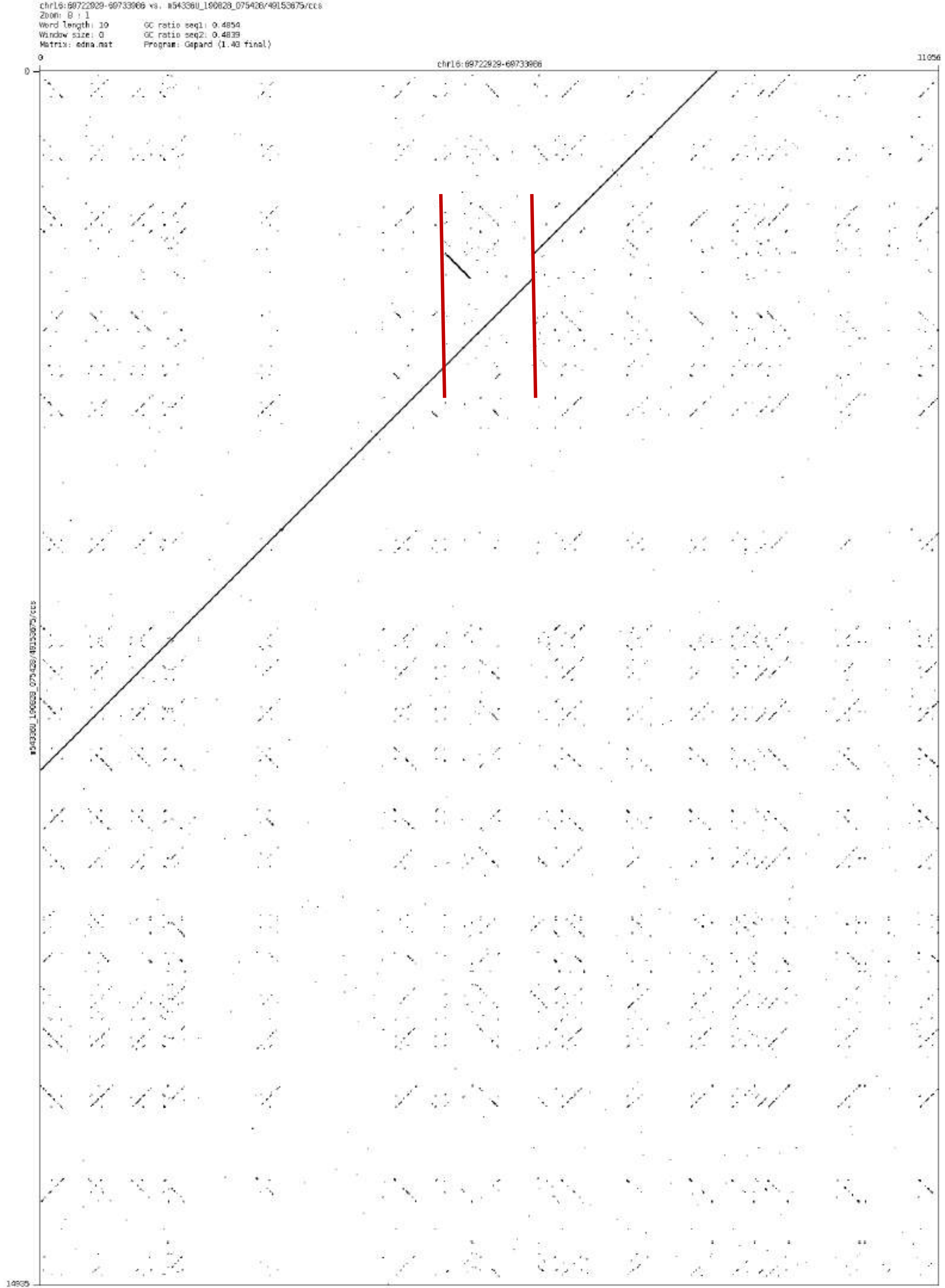

chr16:69722929-69733986 vs. m54336U_190828_075426/49153675/ccs
Zoom: 8 : 1
Word length: 10
Window size: 0
Matrix: edna.mat
GC ratio seq1: 0.4854
GC ratio seq2: 0.4839
Program: Gepard (1.40 final)
0
chr16:69722929-69733986
11056
0
m54336U_190828_075426/49153675/ccs
14905

chr17:10983538-10992414

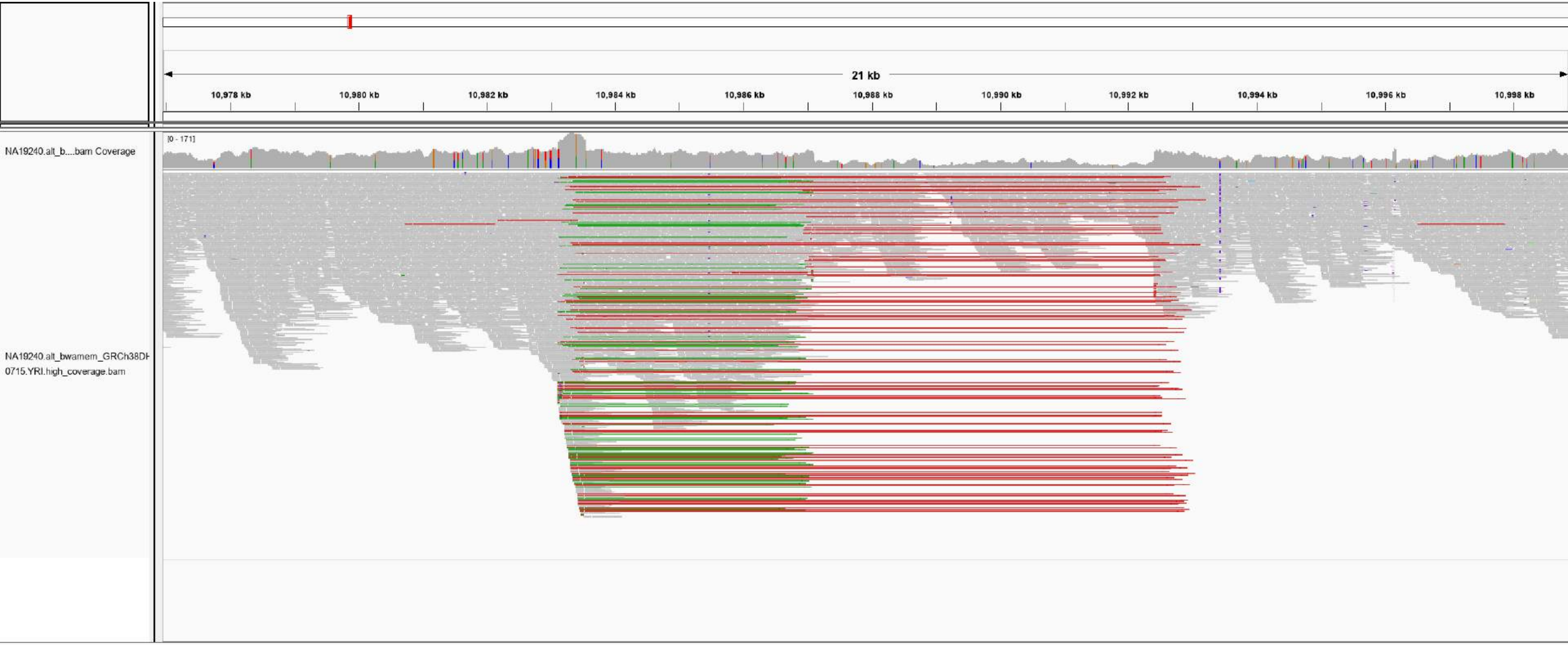

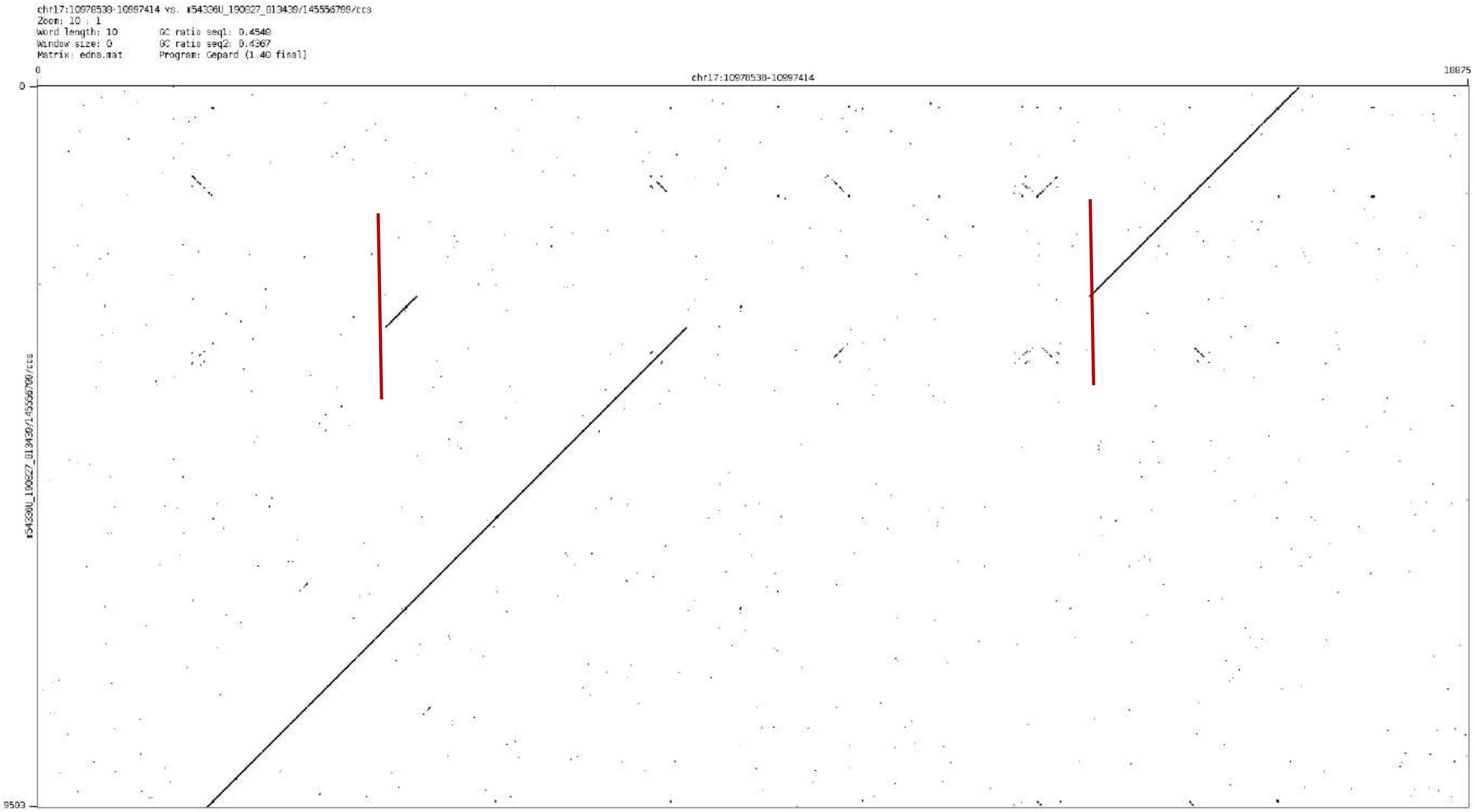
chr17:10978538-10997414 vs. #54336U_190827_013439/145556799/ccs
Zoom: 10 : 1
Word length: 10
Window size: 0
Matrix: edna.mat
GC ratio seq1: 0.4548
GC ratio seq2: 0.4367
Program: Gepard (1.40 final)
0
chr17:10978538-10997414
18875
0
9503

chr17:43358999-43365252

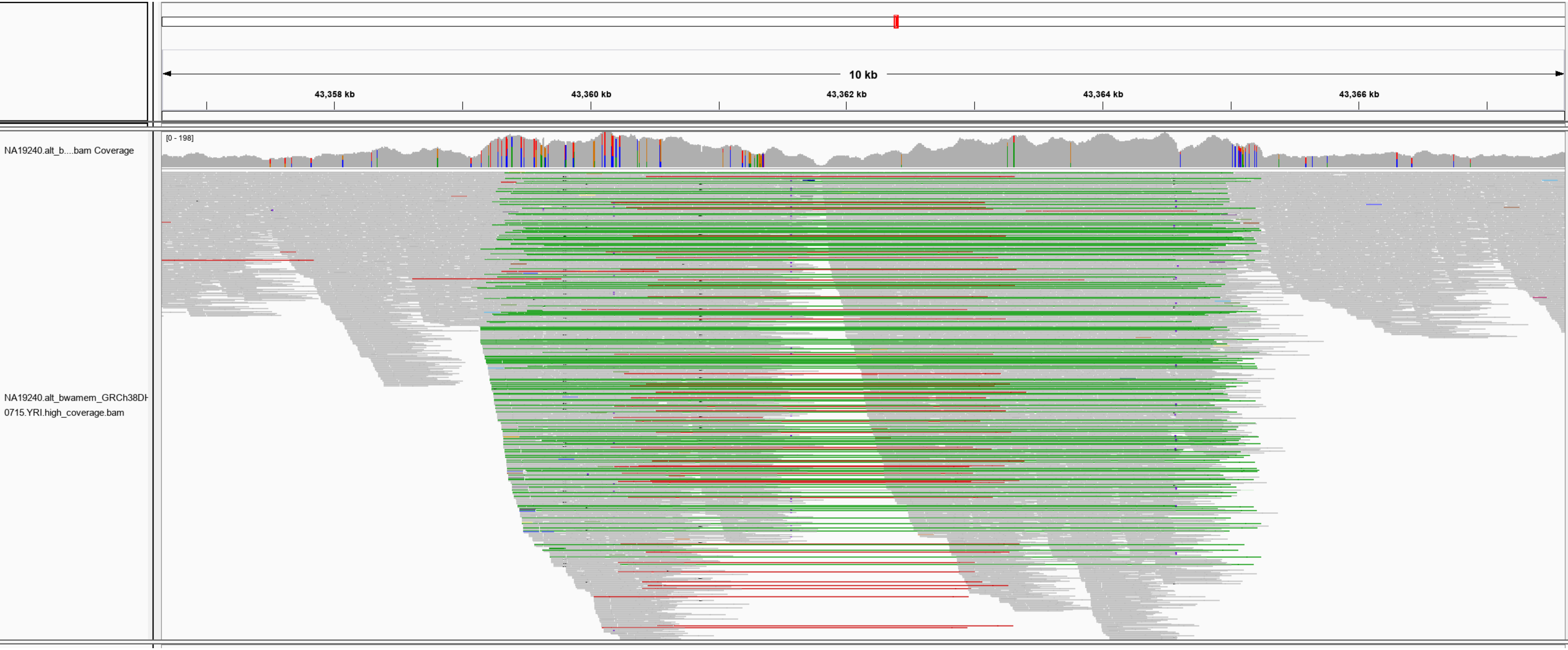

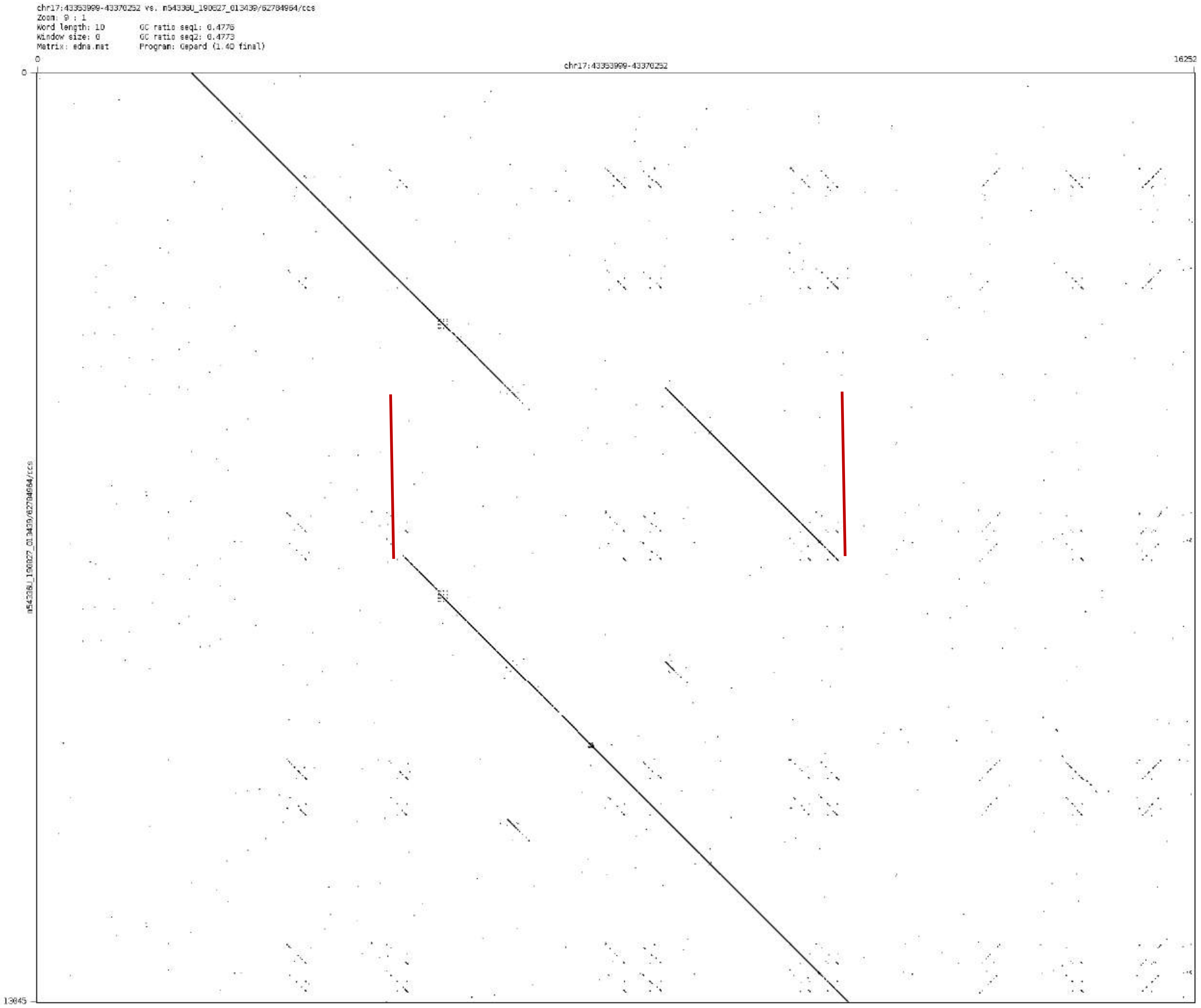
chr17:43353999-43370252 vs. m54336U_190627_013439/62784964/ccs
Zoom: 9 : 1
Word length: 10
Window size: 0
Matrix: edna.mat
GC ratio seq1: 0.4776
GC ratio seq2: 0.4773
Program: Gepard (1.40 final)
0
16252
chr17:43353999-43370252
0
13845
m54336U_190627_013439/62784964/ccs

chr18:11509284-11513469

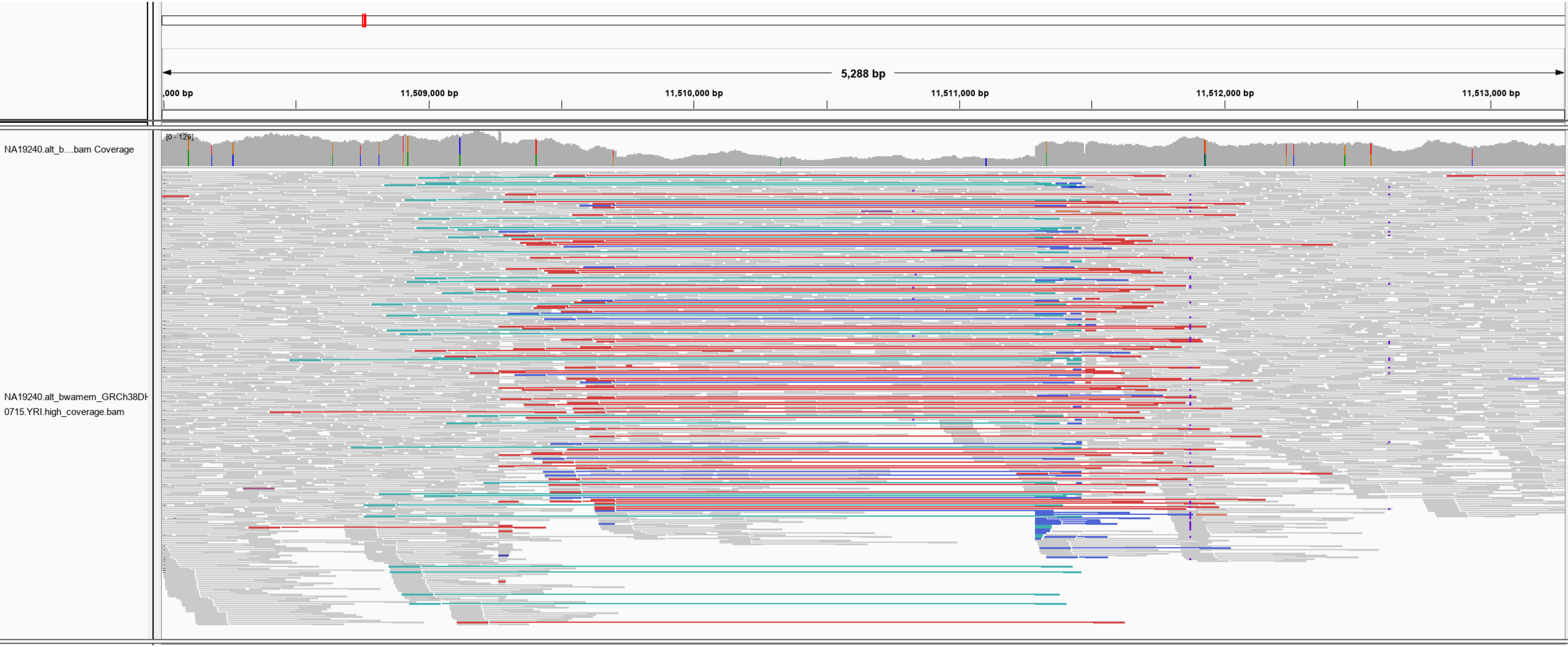

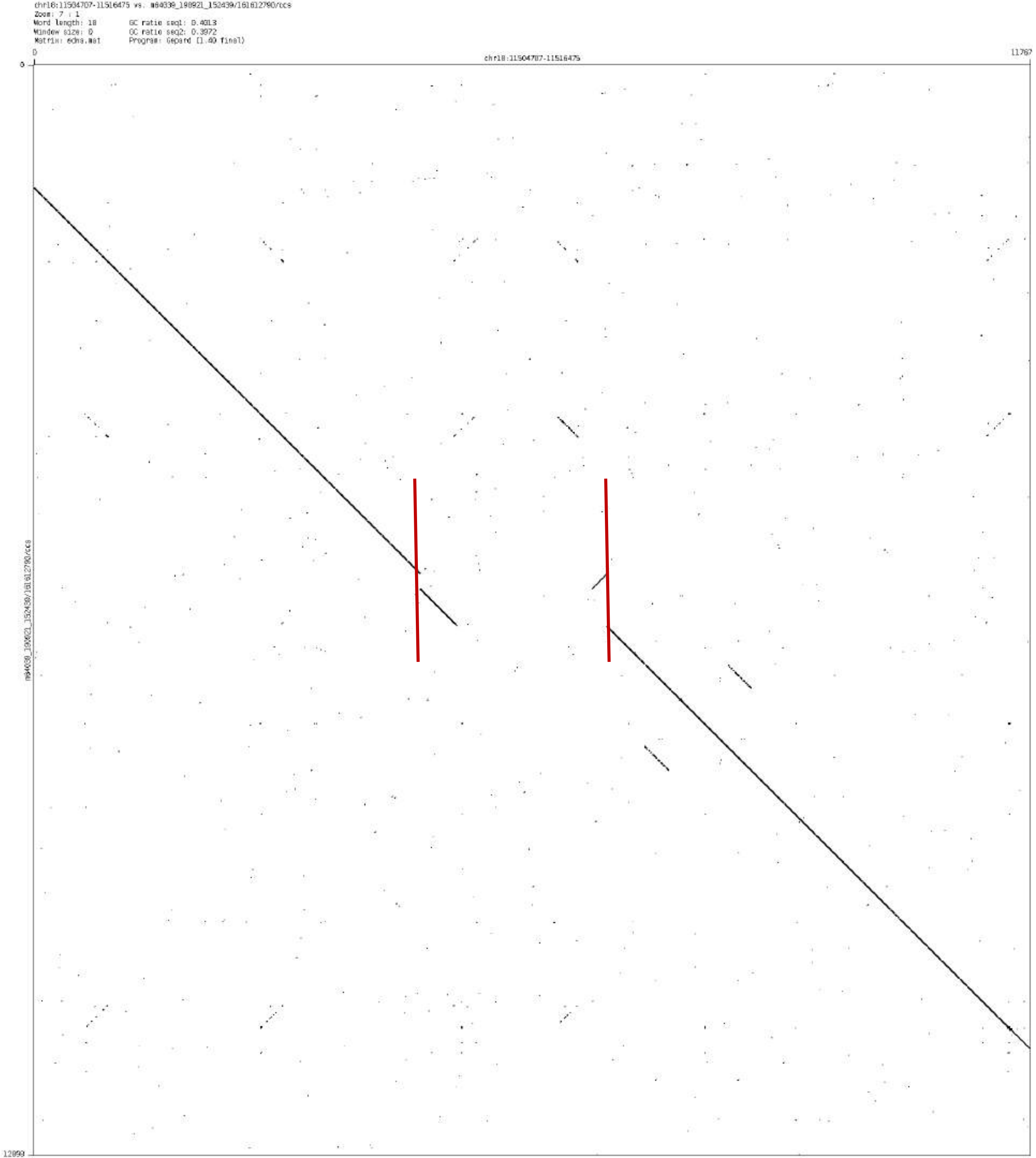
chr18:11504707-11516475 vs. m64039_190921_152439/161612790/ccs
Zoom: 7 : 1
Word length: 18
Window size: 0
Matrix: edna.mat
GC ratio seq1: 0.4013
GC ratio seq2: 0.3972
Program: Gepard (1.40 final)
0
11767
chr18:11504707-11516475
0
12899
m64039_190921_152439/161612790/ccs

chr19:56640093-56640958

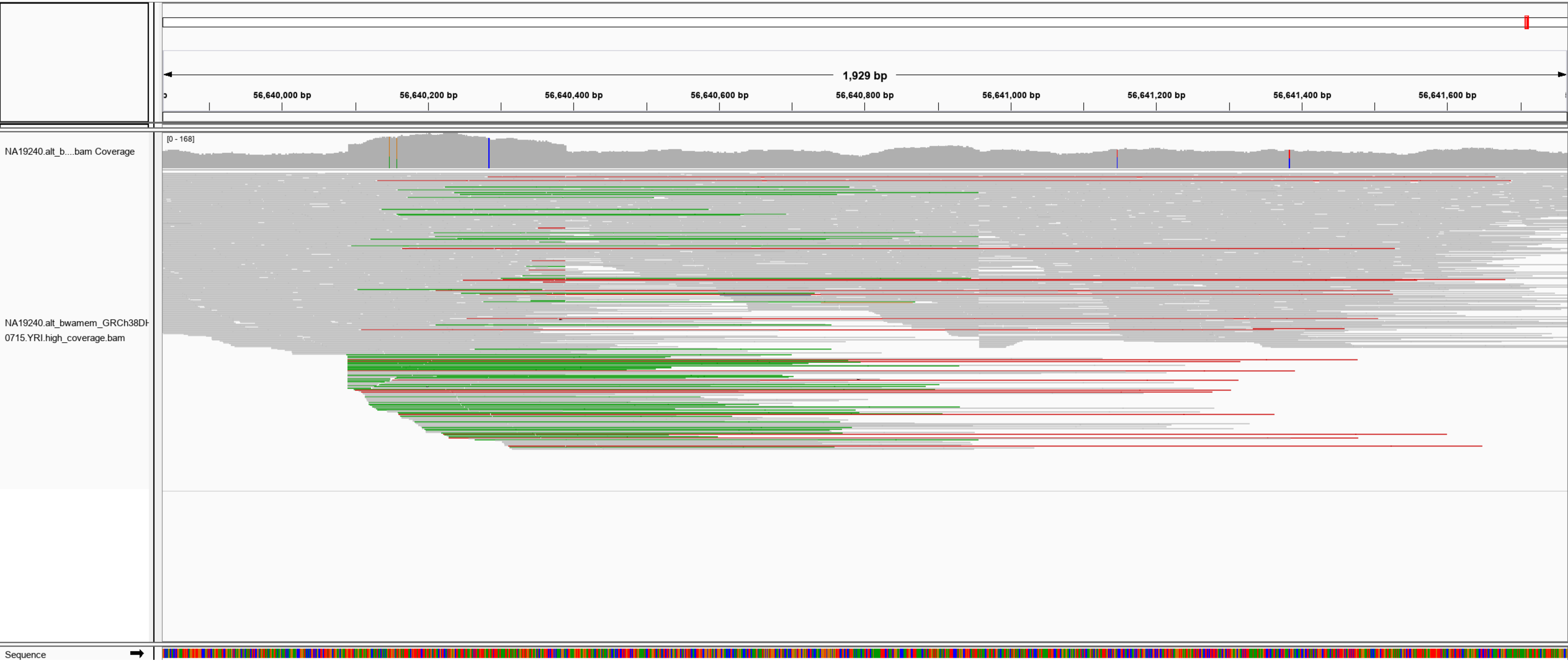

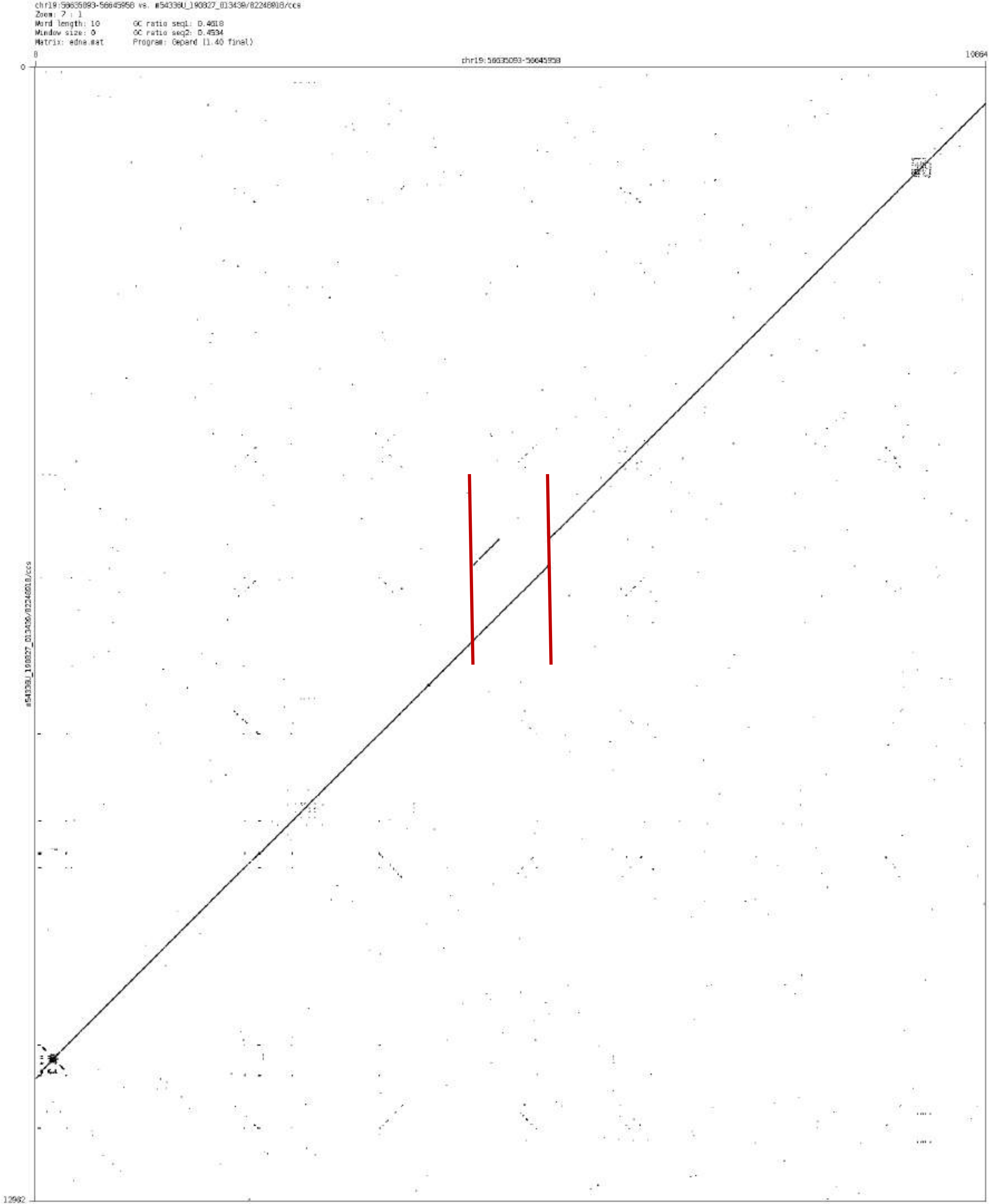
Zoom: 7 : 1
Word length: 10
Window size: 0
Matrix: edna.mat
Program: Gepard (1.40 final)

chr20:2379153-2379968

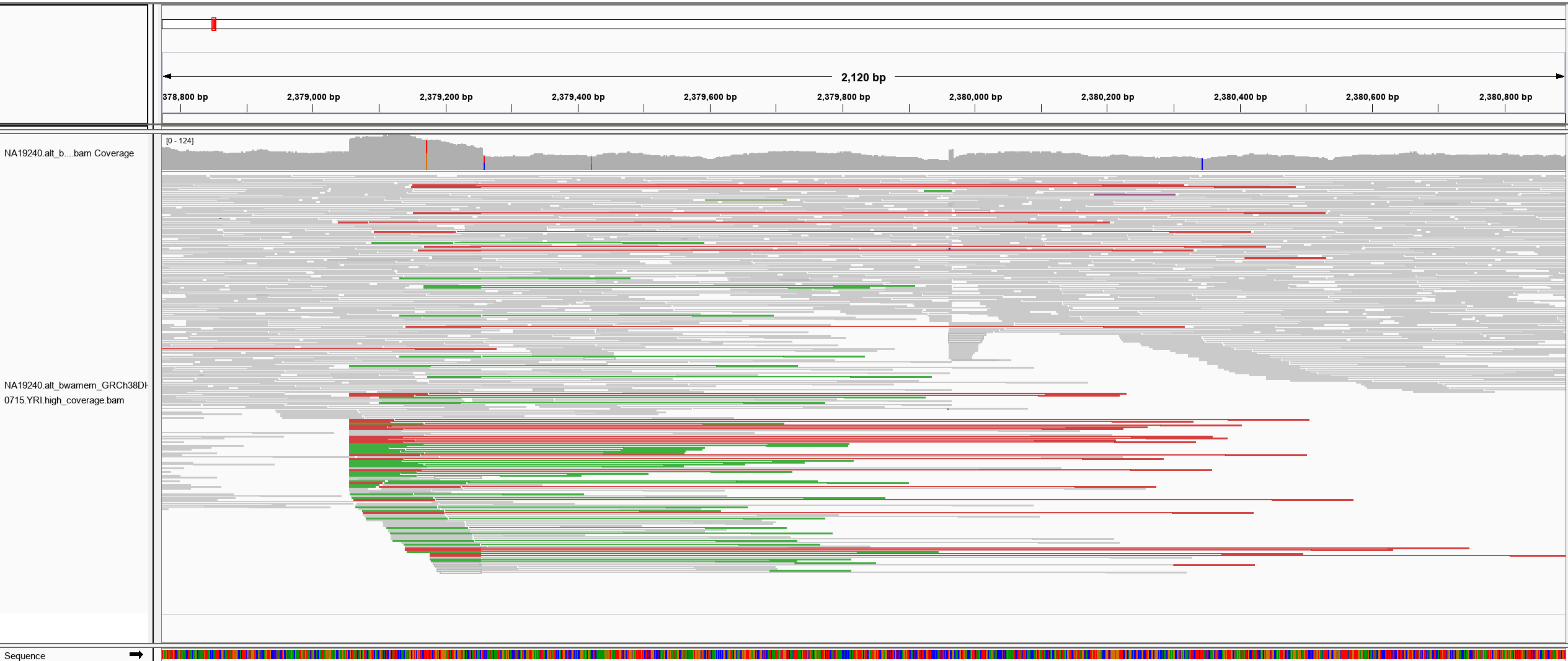

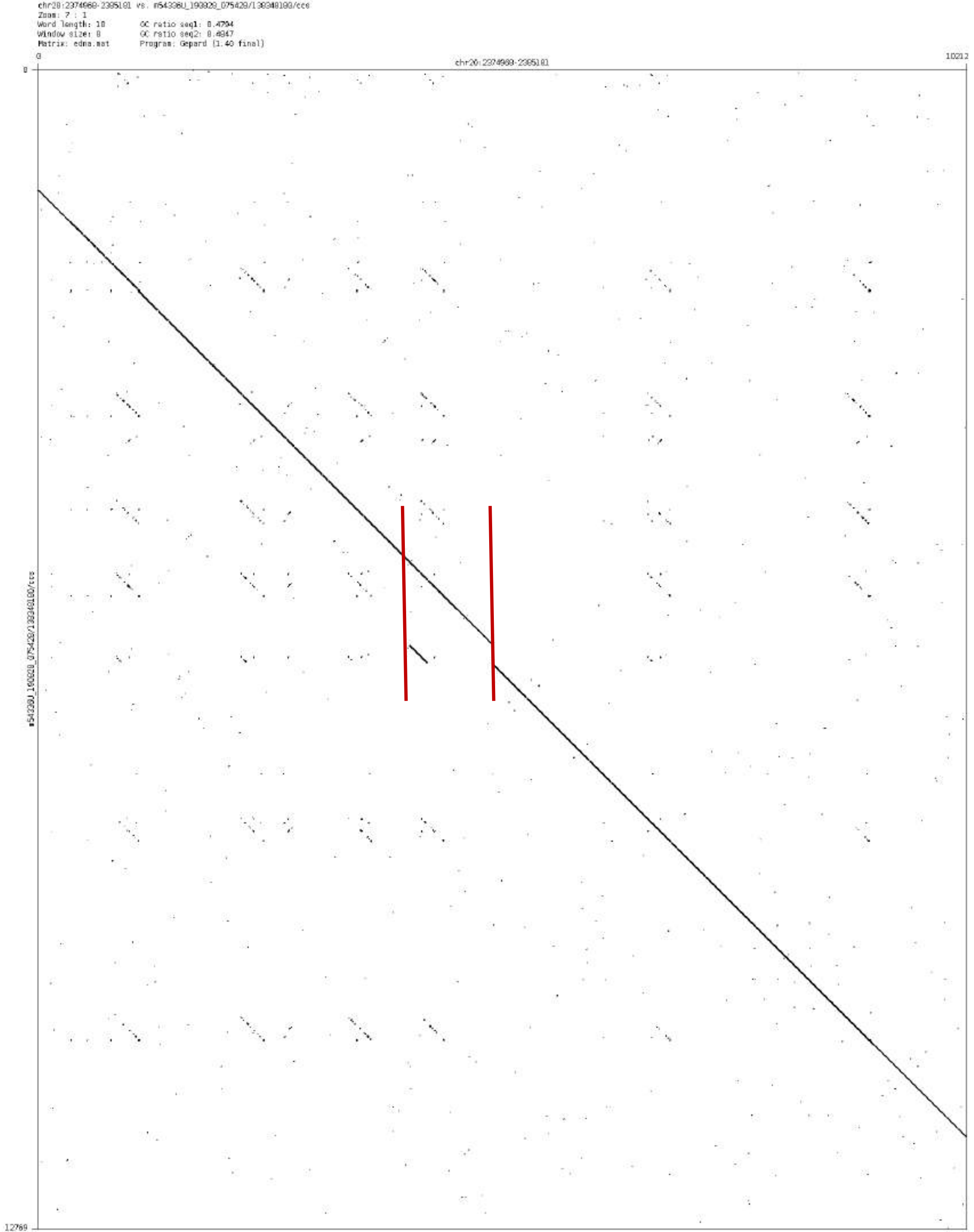
chr20:2374968-2385181 vs. m54336U_190829_075429/138348188/ccs
Zoom: 7 : 1
Word length: 10
Window size: 0
Matrix: edna.mat
GC ratio seq1: 0.4794
GC ratio seq2: 0.4847
Program: Gepard (1.40 final)
0
chr20:2374968-2385181
10212
0
m54336U_190829_075429/138348188/ccs
12769

chr20:23122542-23122996

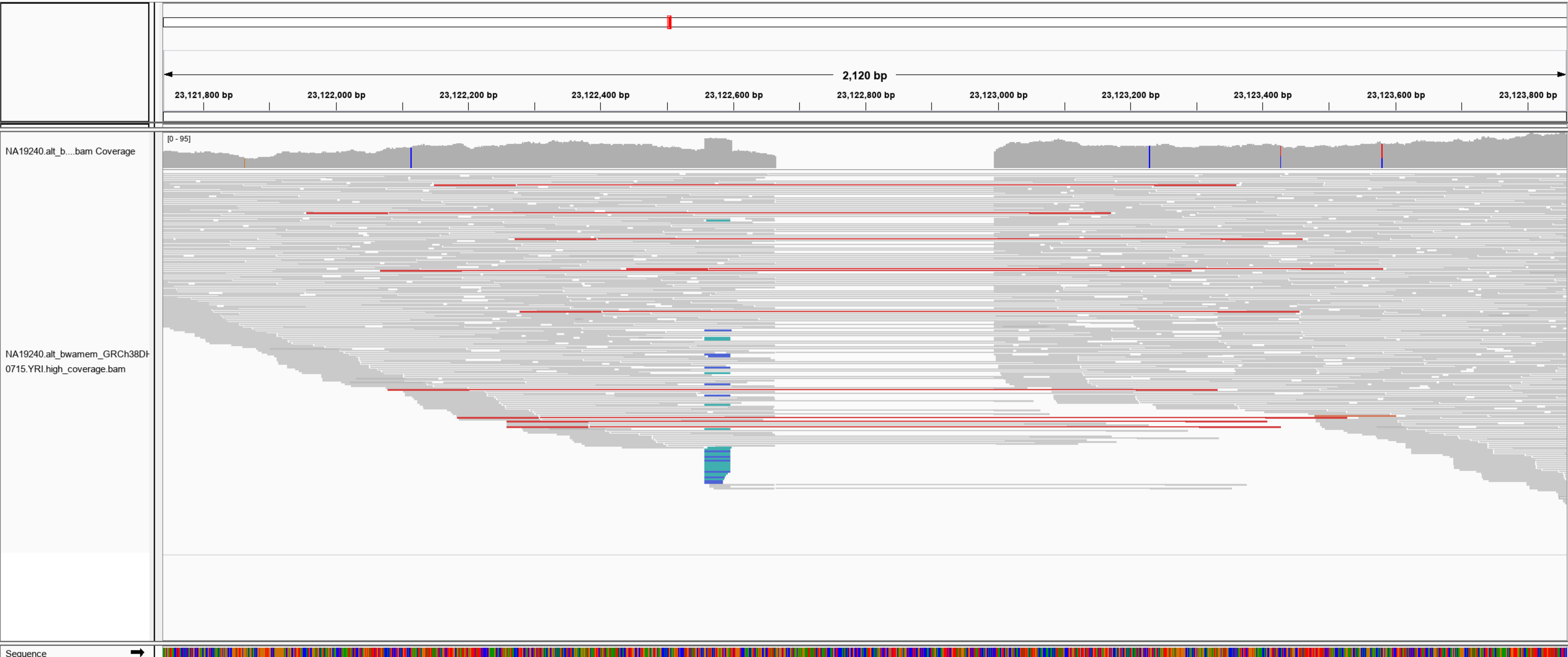

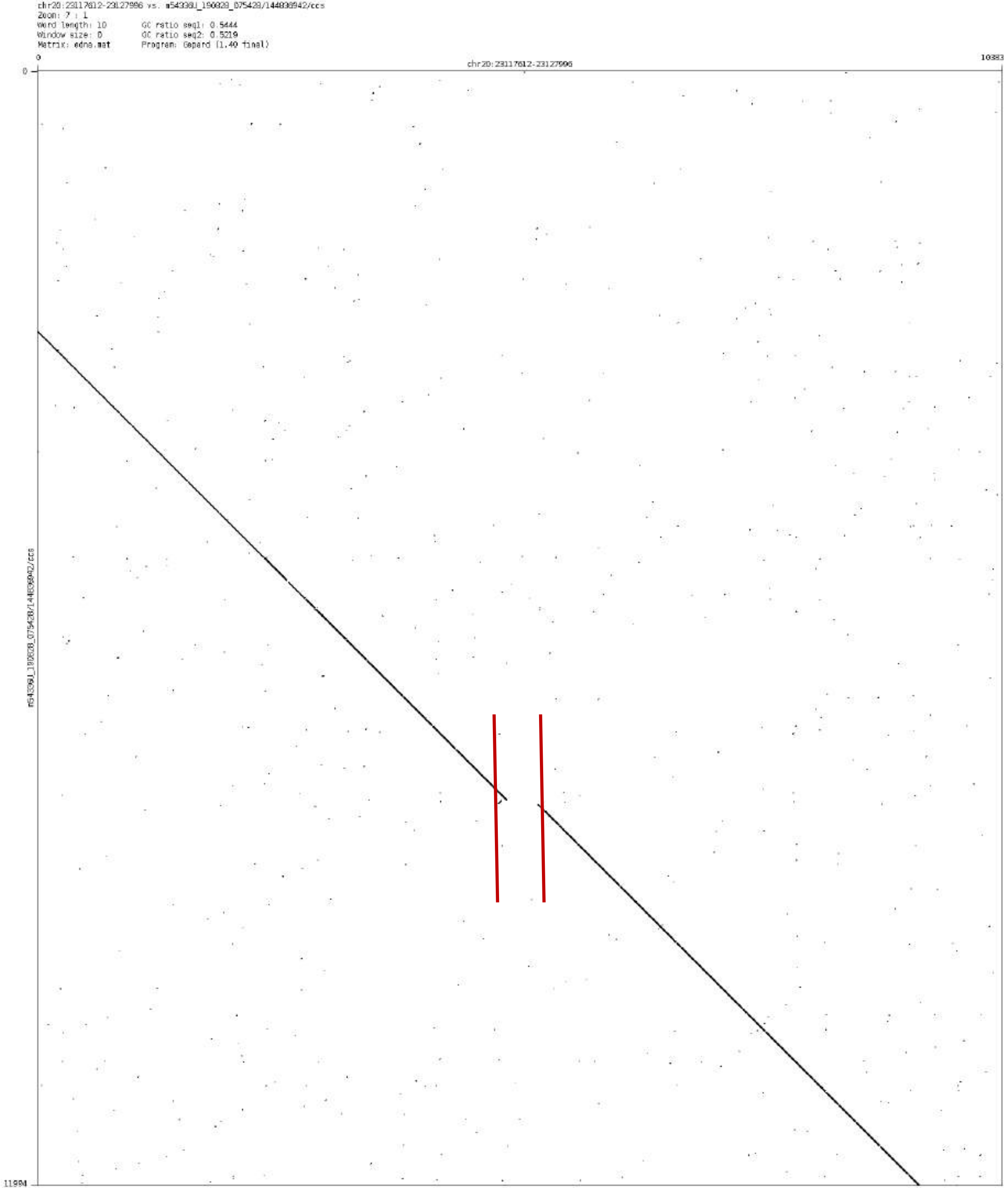
chr20:23117612-23127996 vs. m54338U_190828_075428/144838942/ccs
Zoom: 7 : 1
Word length: 10
GC ratio seq1: 0.5444
Window size: 0
GC ratio seq2: 0.5219
Matrix: edna.mat
Program: Gepard (1.40 final)
0
chr20:23117612-23127996
10383
0
11994

chr22:49368449-49373094

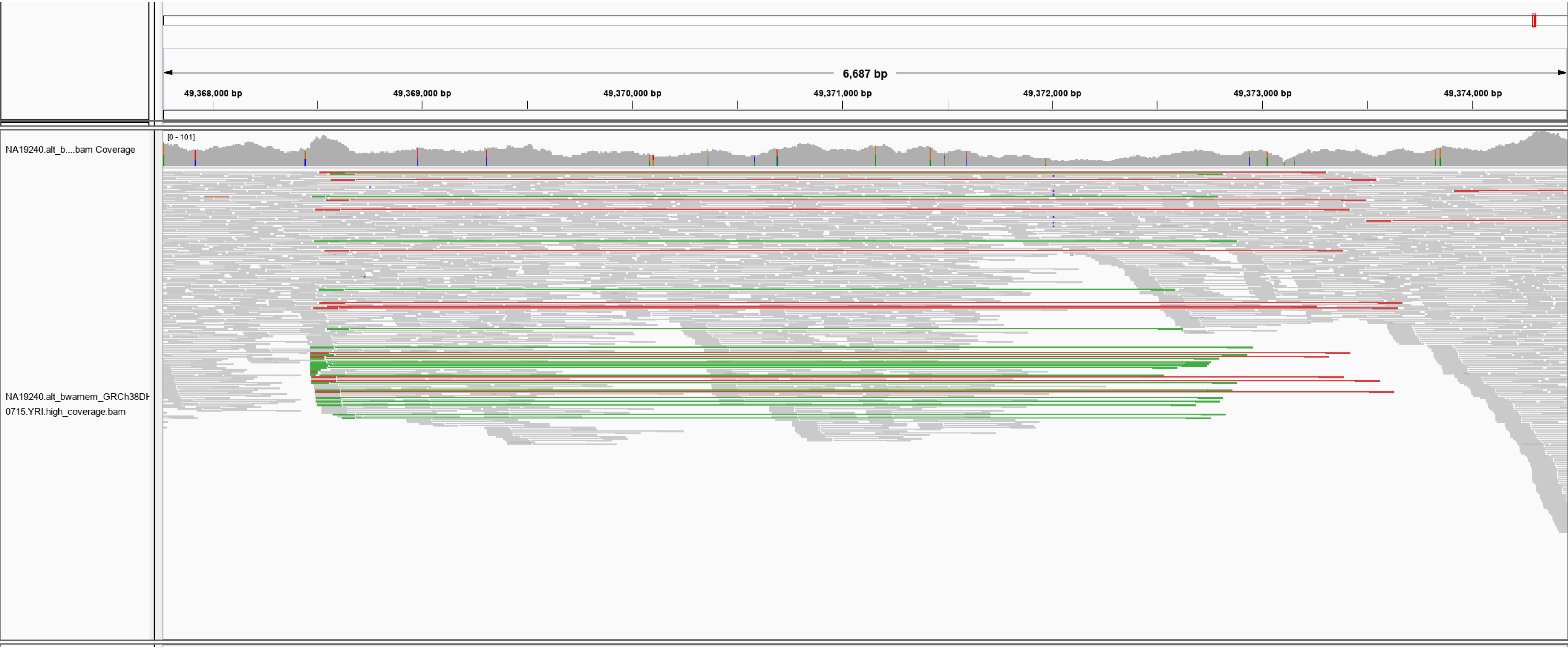

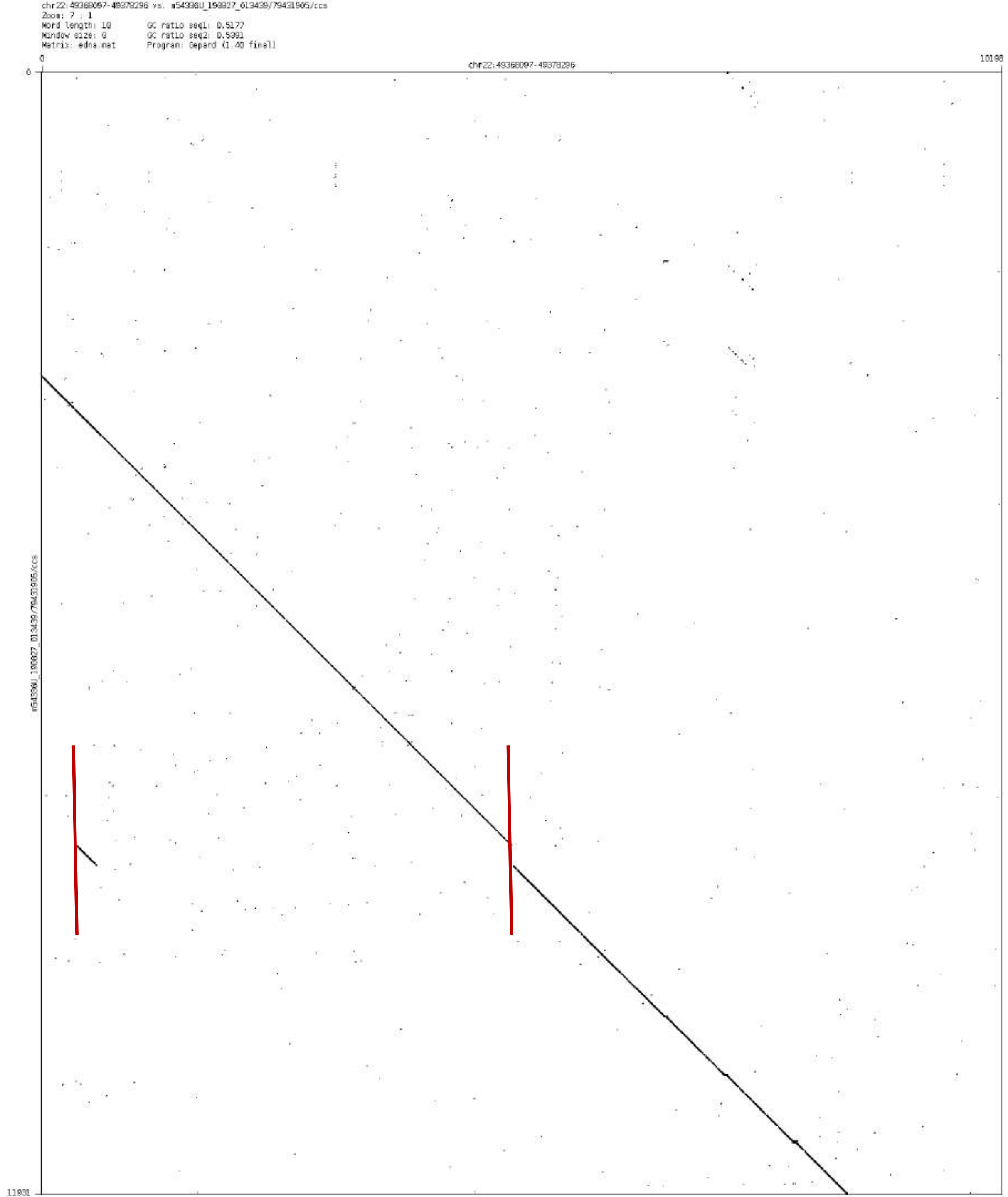

chr22:49368097-49378296 vs. m54338U_190827_013439/79431905/ccs
Zoom: 7 : 1
Word length: 10
Window size: 0
Matrix: edna.mat
GC ratio seq1: 0.5177
GC ratio seq2: 0.5391
Program: Gepard (1.40 final)
chr22:49368097-49378296
m54338U_190827_013439/79431905/ccs
0
10198
11981

SK-BR-3 benchmark CSVs

chr1:61060495-61062387

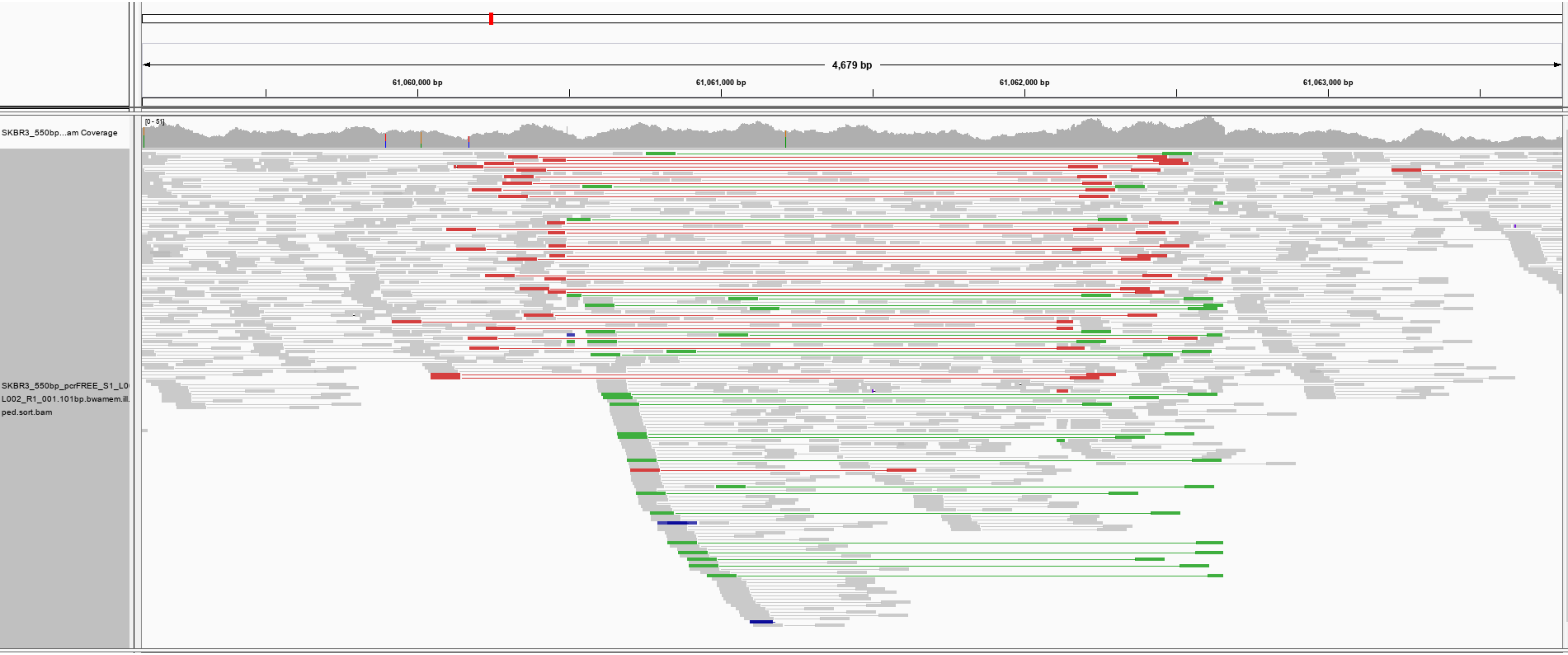

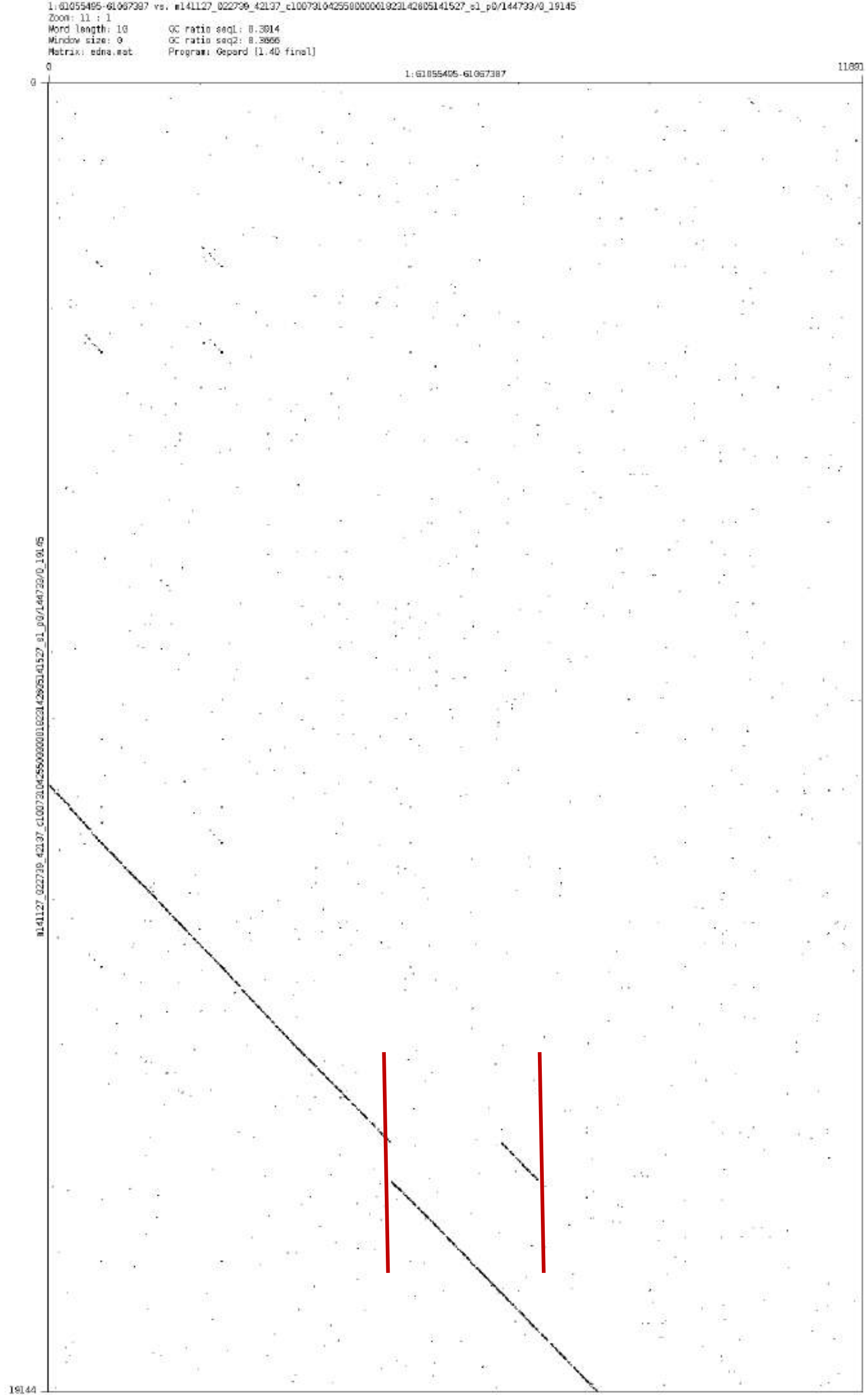

1:61055495-61067387 vs. m141127_022739_42137_c100731042550000001823142805141527_s1_p0/144733/0_19145
Zoom: 11 : 1
Word length: 10
GC ratio seq1: 0.3014
Window size: 0
GC ratio seq2: 0.3066
Matrix: edna.mat
Program: Gepard [1.40 final]
1:61055495-61067387
0
11881
19144

chr1:84056441-84057506

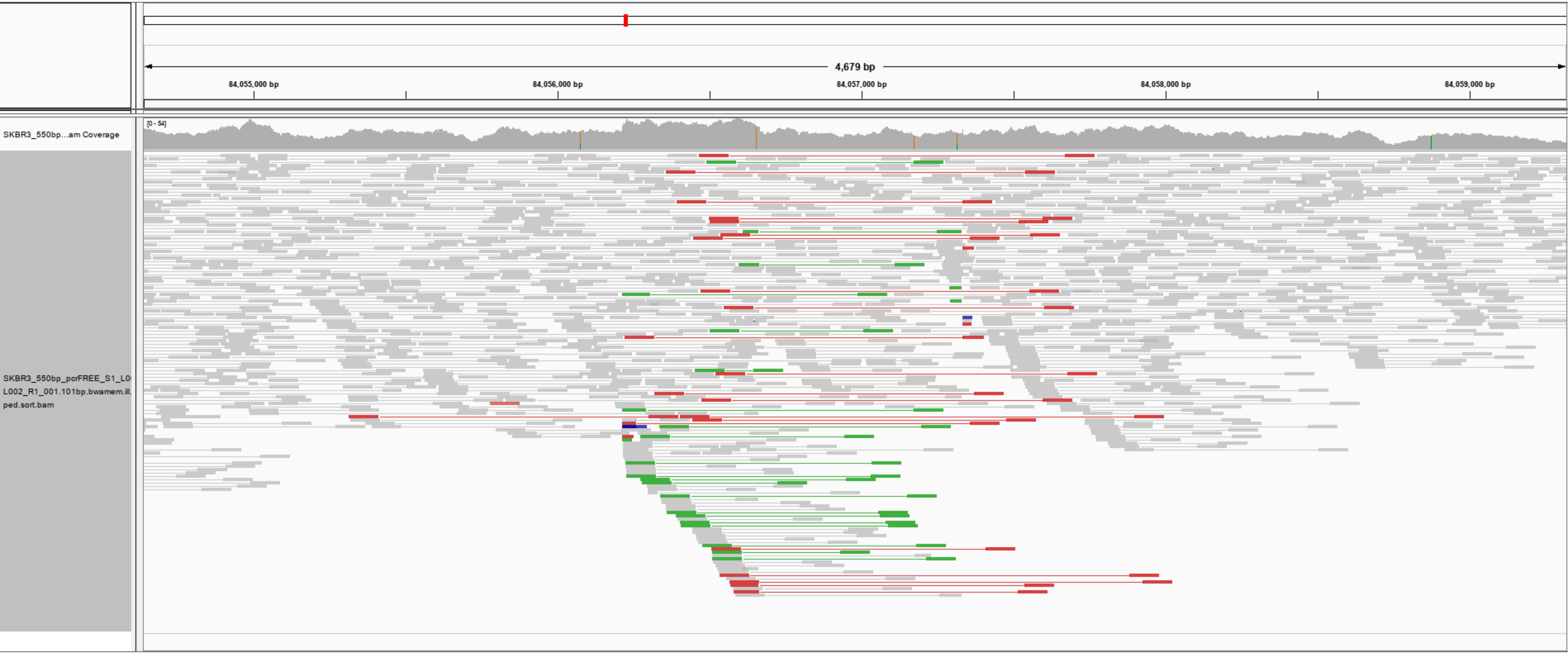

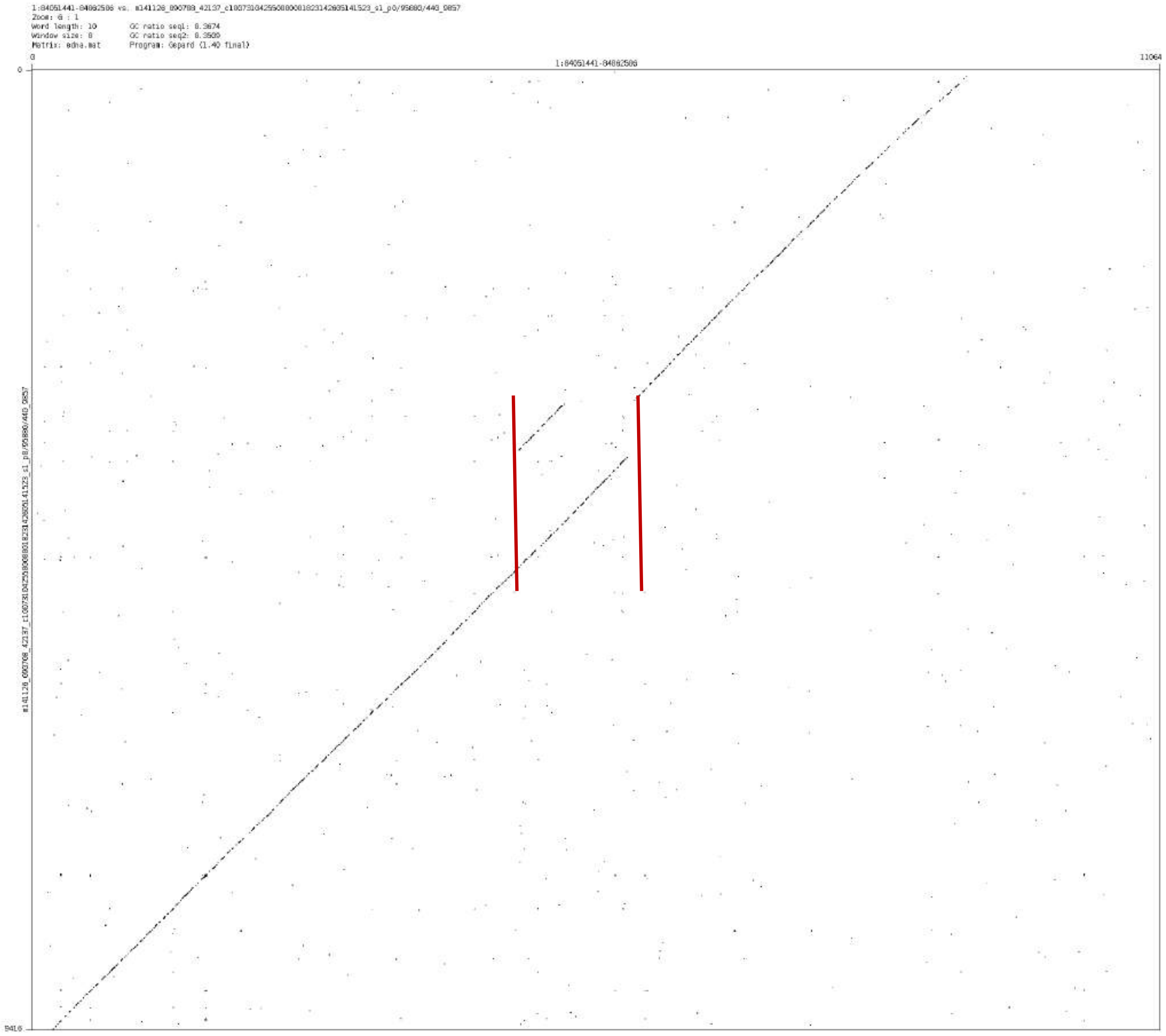
1:84051441-84062506 vs. m141126_090708_42137_c100731042550000001823142605141523_s1_p0/95880/440_9857
Zoom: 6 : 1
Word length: 10
GC ratio seq1: 0.3674
Window size: 0
GC ratio seq2: 0.3500
Matrix: edna.mat
Program: Gepard (1.40 final)
1:84051441-84062506
11064
9416

chr1:144898739-144906810

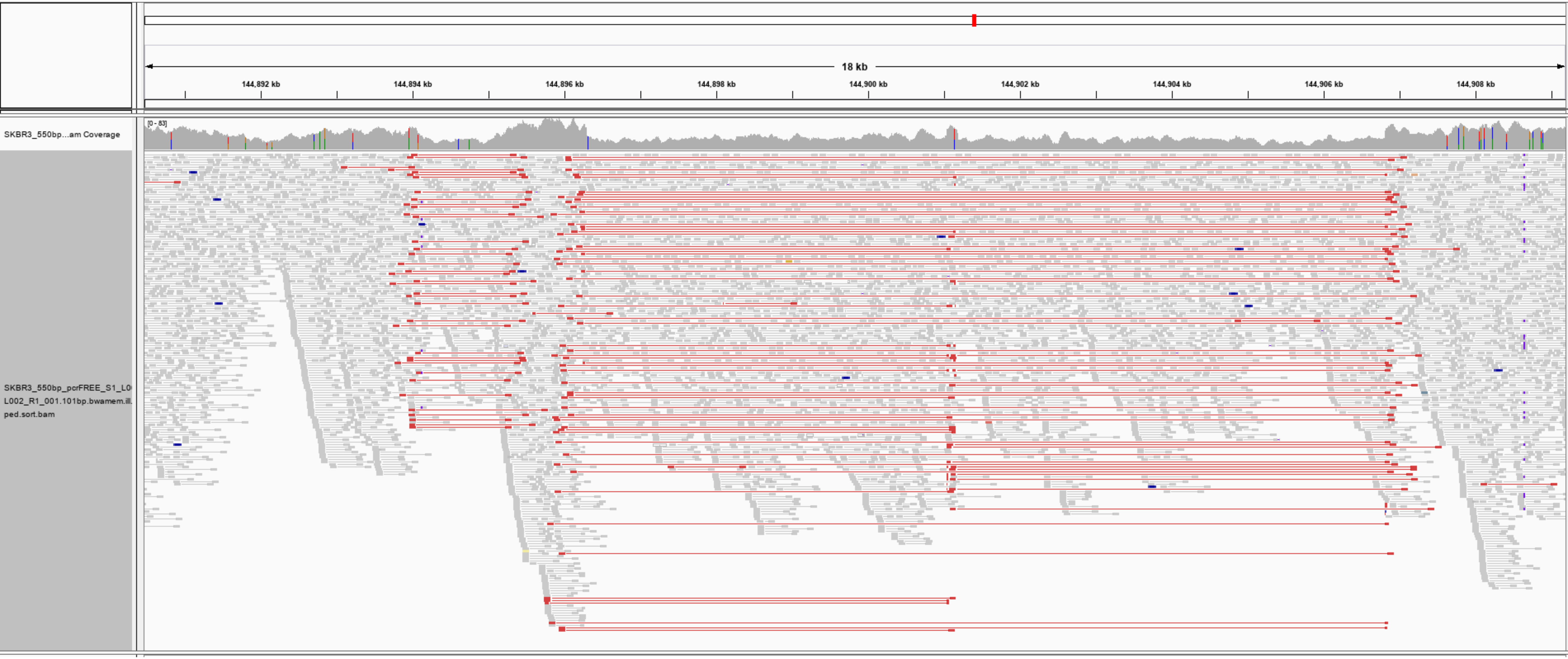

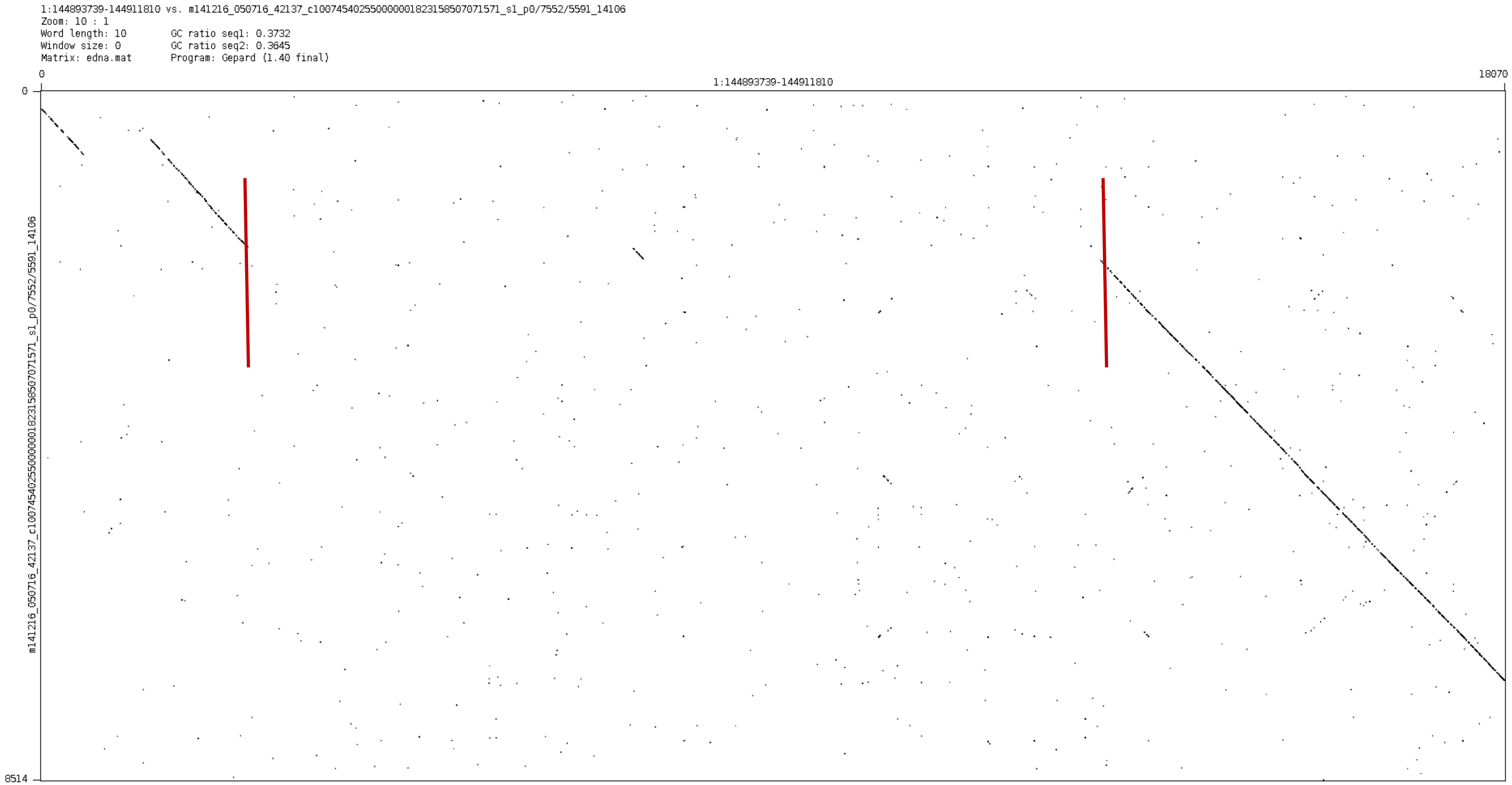
1:144893739-144911810 vs. m141216_050716_42137_c100745402550000001823158507071571_s1_p0/7552/5591_14106
Zoom: 10 : 1
Word length: 10
Window size: 0
Matrix: edna.mat
GC ratio seq1: 0.3732
GC ratio seq2: 0.3645
Program: Gepard (1.40 final)
0
1:144893739-144911810
18070
0
8514
m141216_050716_42137_c100745402550000001823158507071571_s1_p0/7552/5591_14106

chr1:187464829-187466729

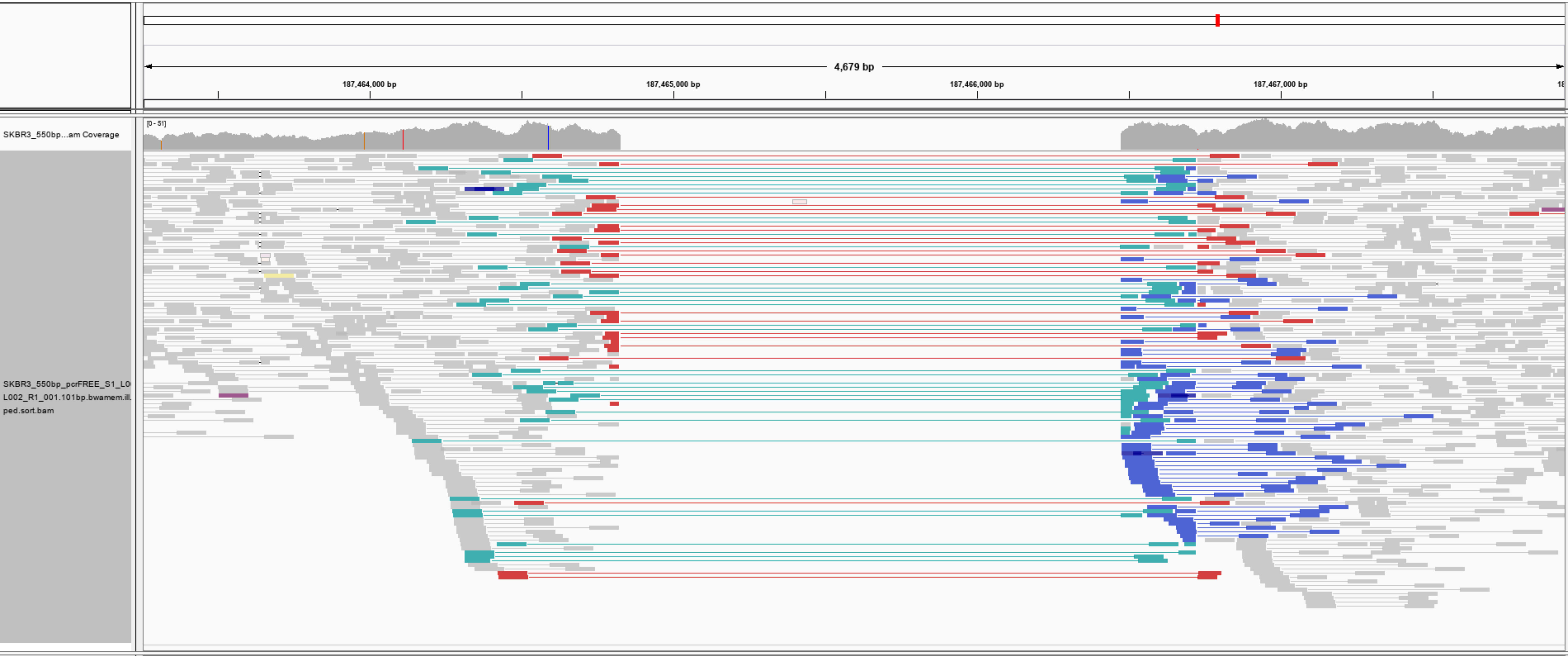

1:187459829-187471729 vs. m141127_131041_42137_c100731082550000001823142605141581_s1_p0/137037/91_6657
Zoom: 7 : 1
Word length: 10 GC ratio seq1: 0.3613
Window size: 0 GC ratio seq2: 0.3479
Matrix: edna.mat Program: Gepard (1.40 final)

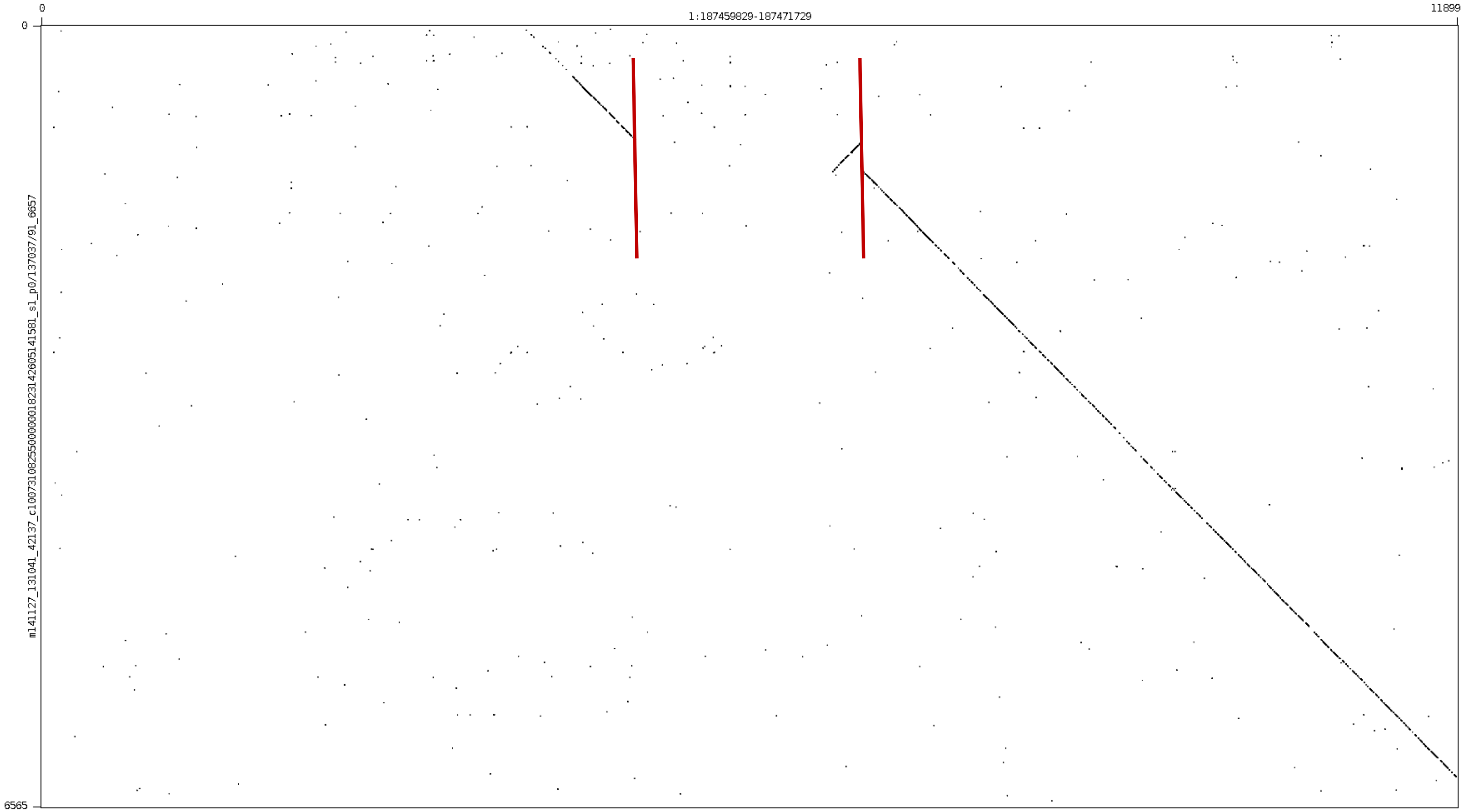

chr1:207292347-207293196

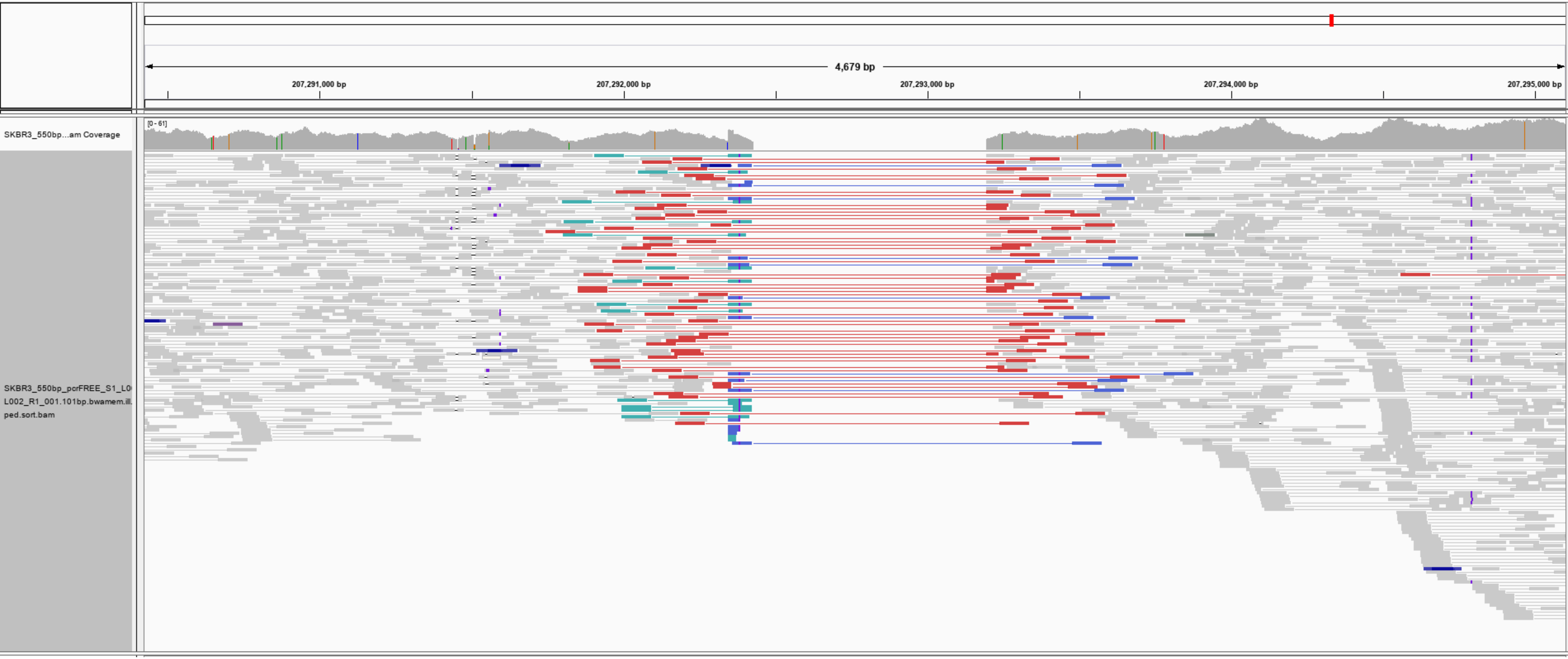

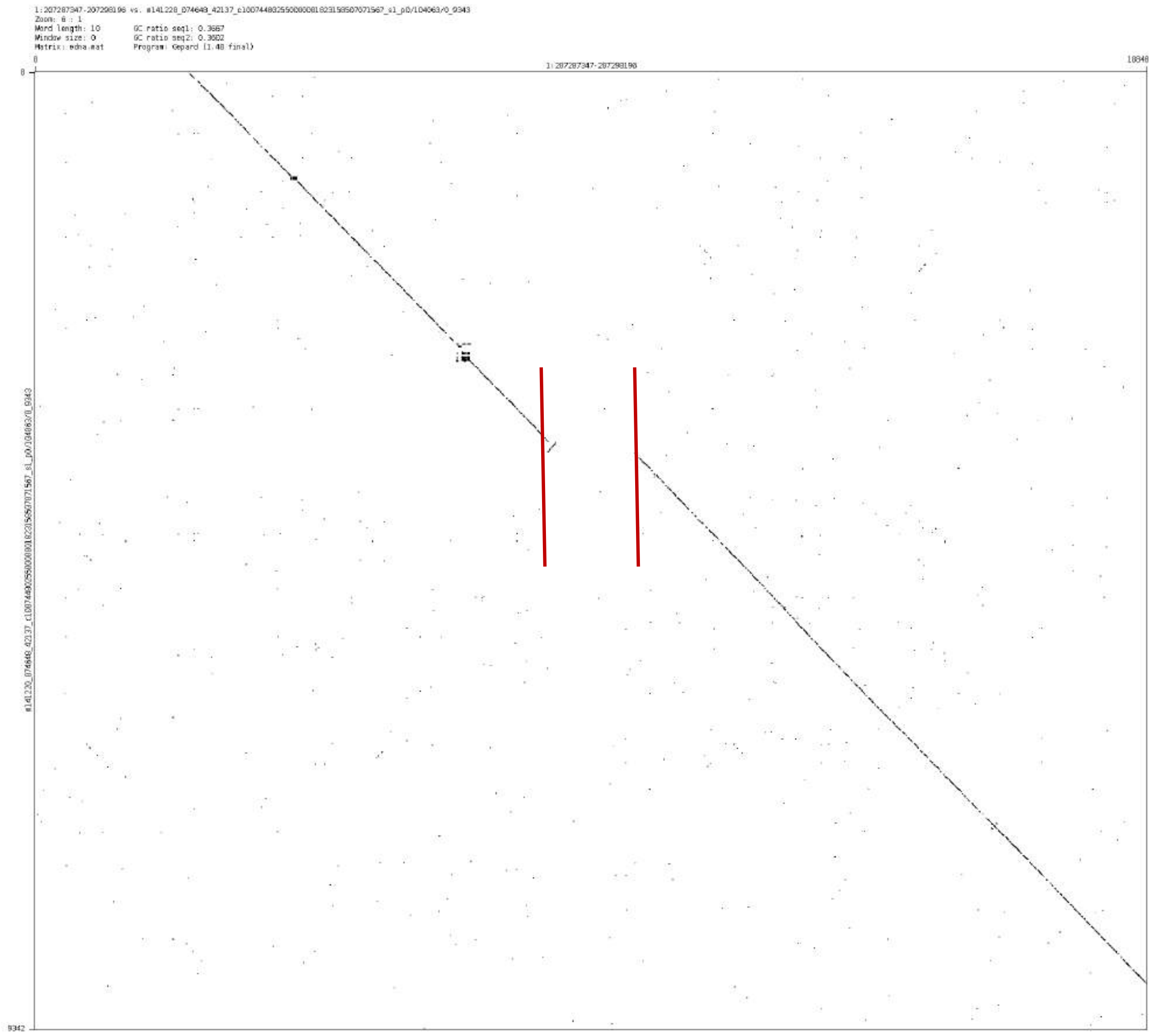
Zoom: 6 : 1
Word length: 10
Window size: 0
Matrix: edna.mat
GC ratio seq1: 0.3667
GC ratio seq2: 0.3602
Program: Gepard (1.40 final)
1:207287347-207298196
0
10848
9342

chr2:16406412-16407796

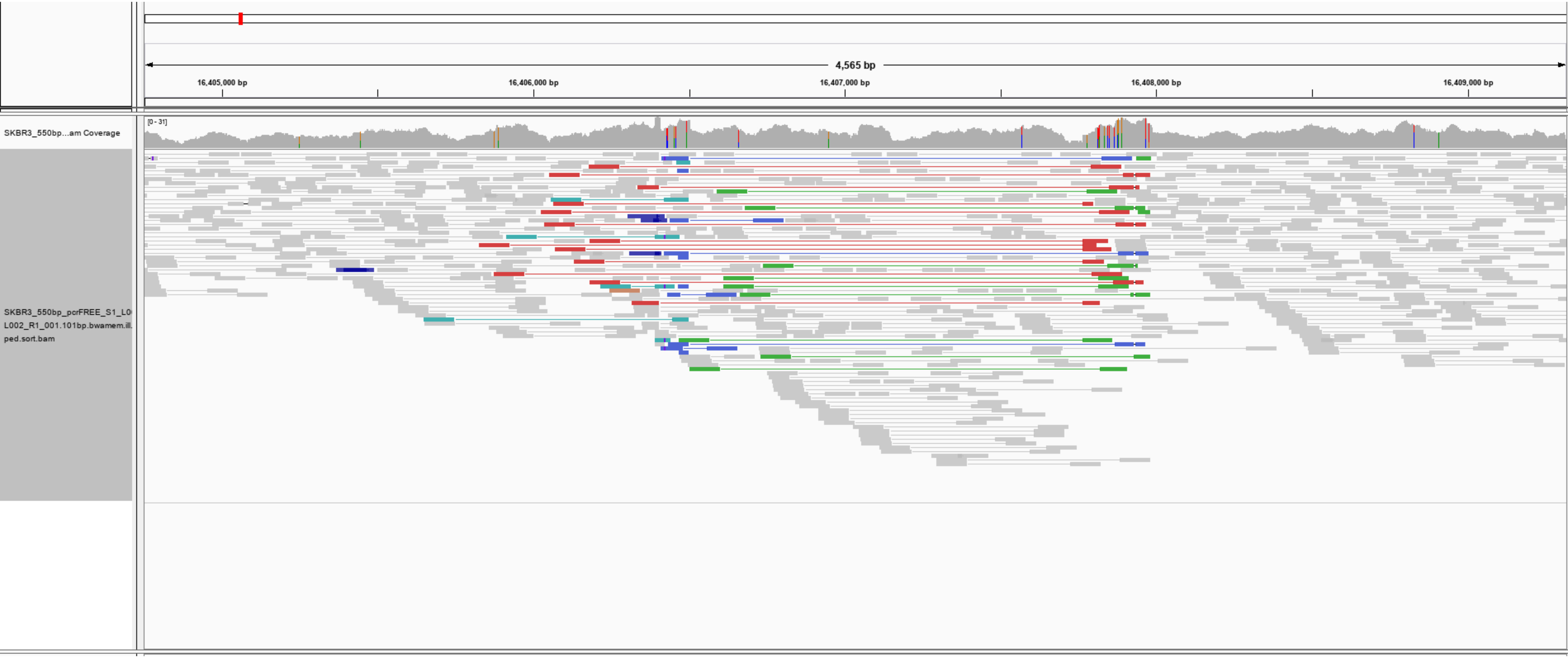

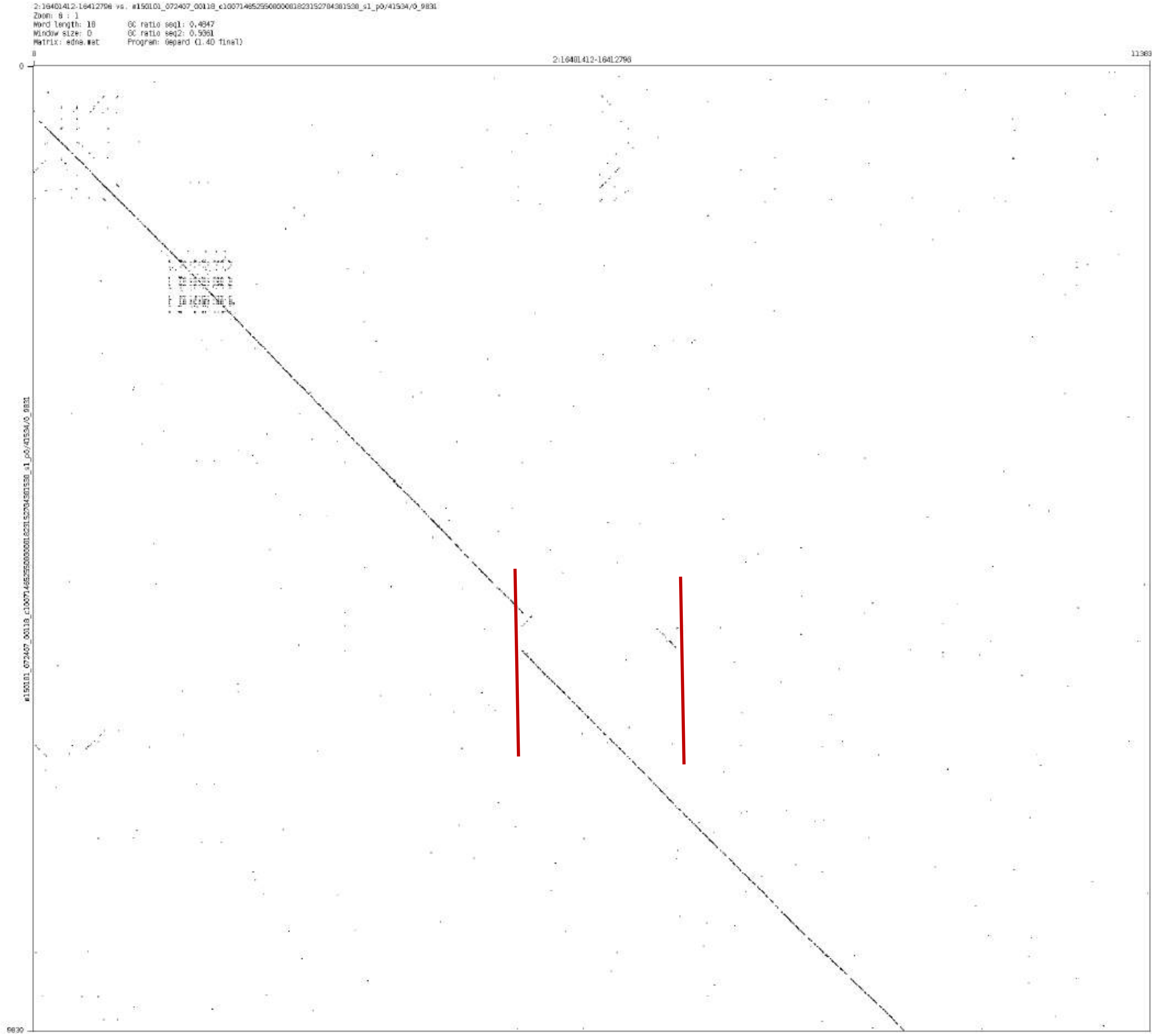
Window size: 0
Matrix: edna.mat
GC ratio seq1: 0.4847
GC ratio seq2: 0.5361
Program: Gepard (1.40 final)
2:16401412-16412798

chr2:120417142-120418497

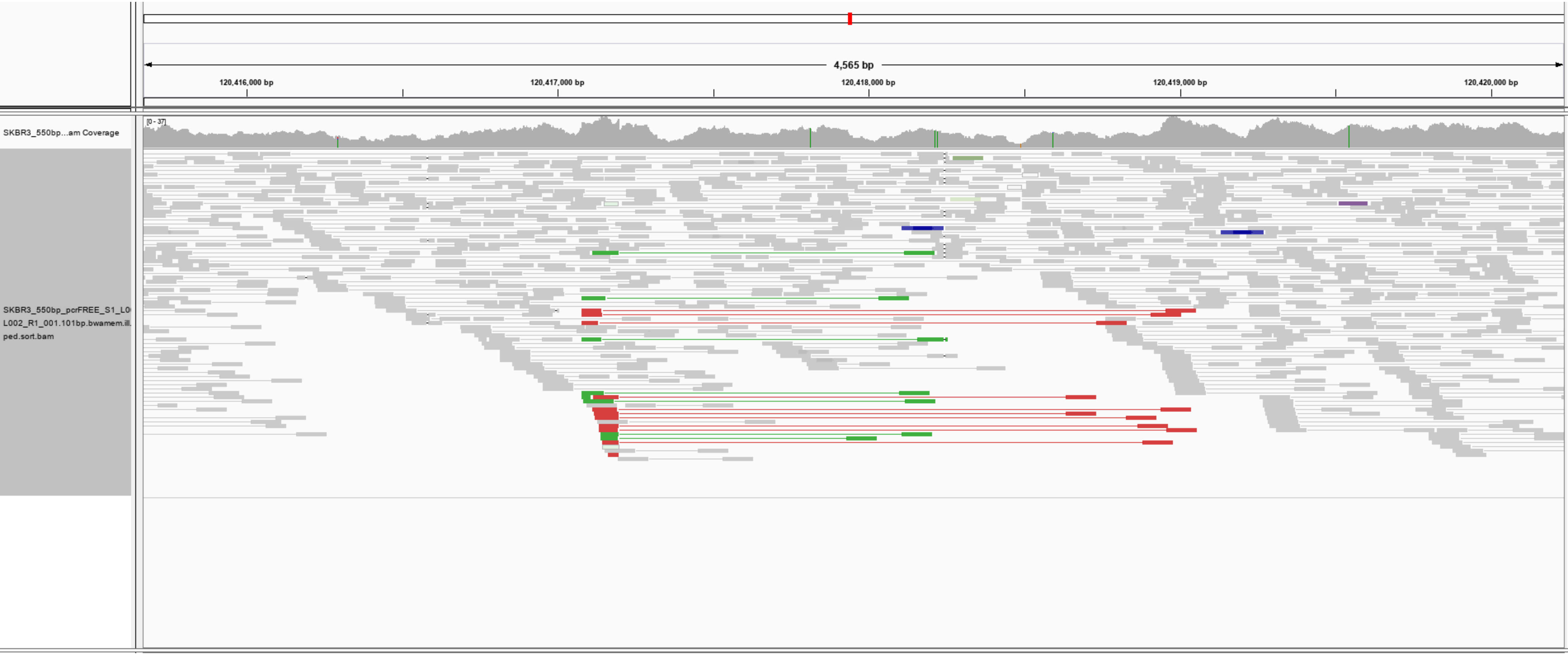

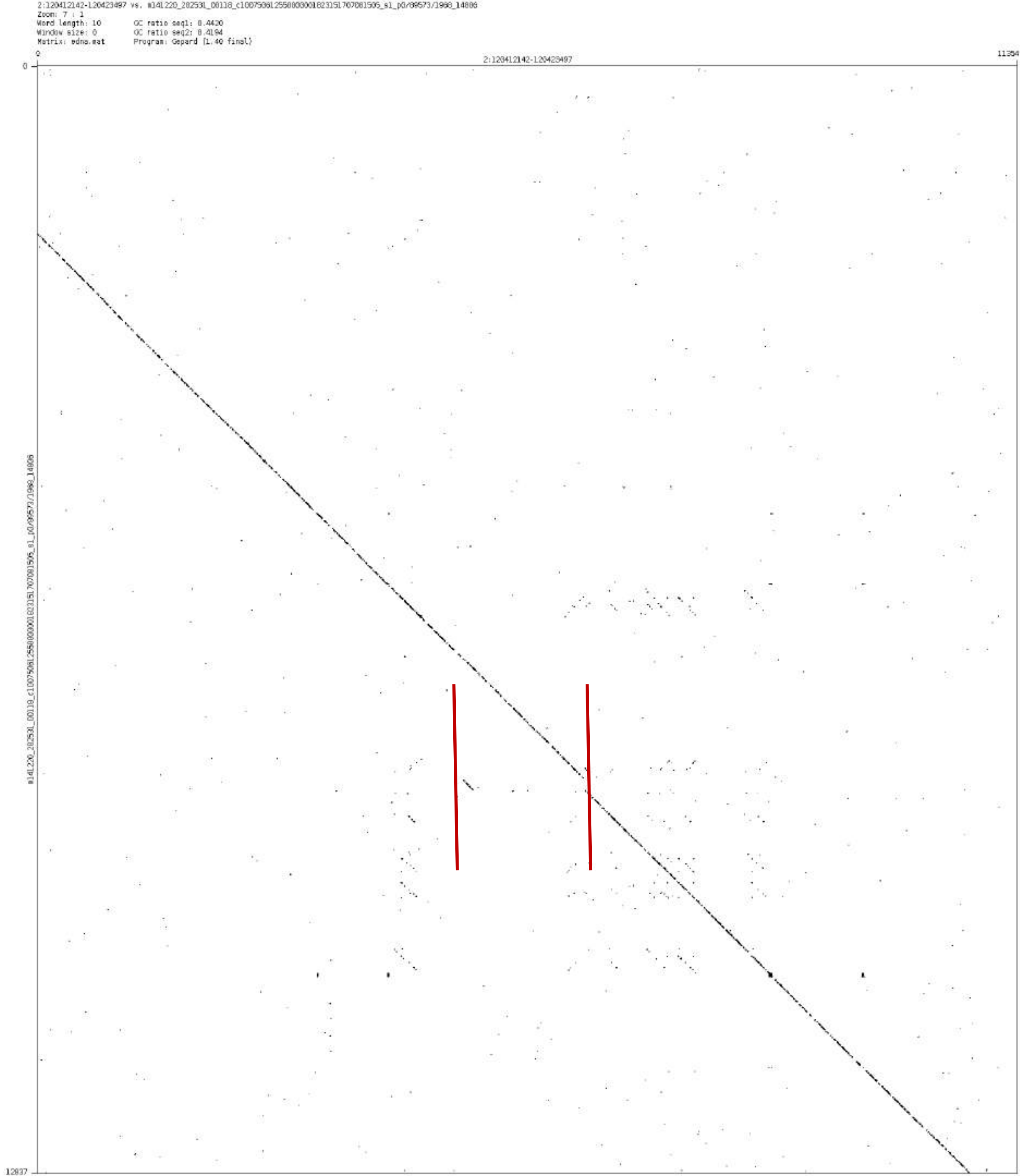
2:120412142-120423497 vs. m141220_202531_00118_c100750812558000001823151707081505_s1_p0/89573/1968_14806
Zoom: 7 : 1
Word length: 10
Window size: 0
Matrix: edna.mat
GC ratio seq1: 0.4420
GC ratio seq2: 0.4194
Program: Gepard (1.40 final)
0
2:120412142-120423497
11354
0
12837

chr2:225292798-225293577

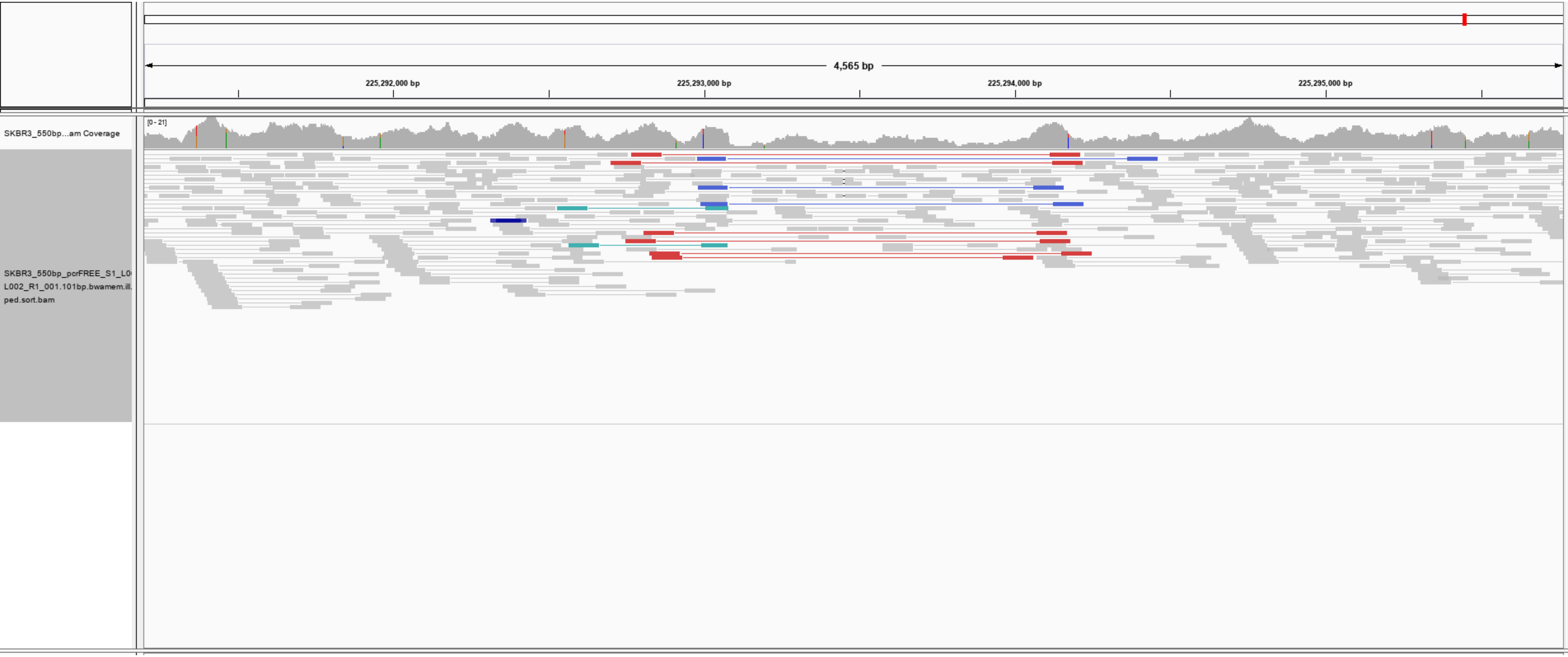

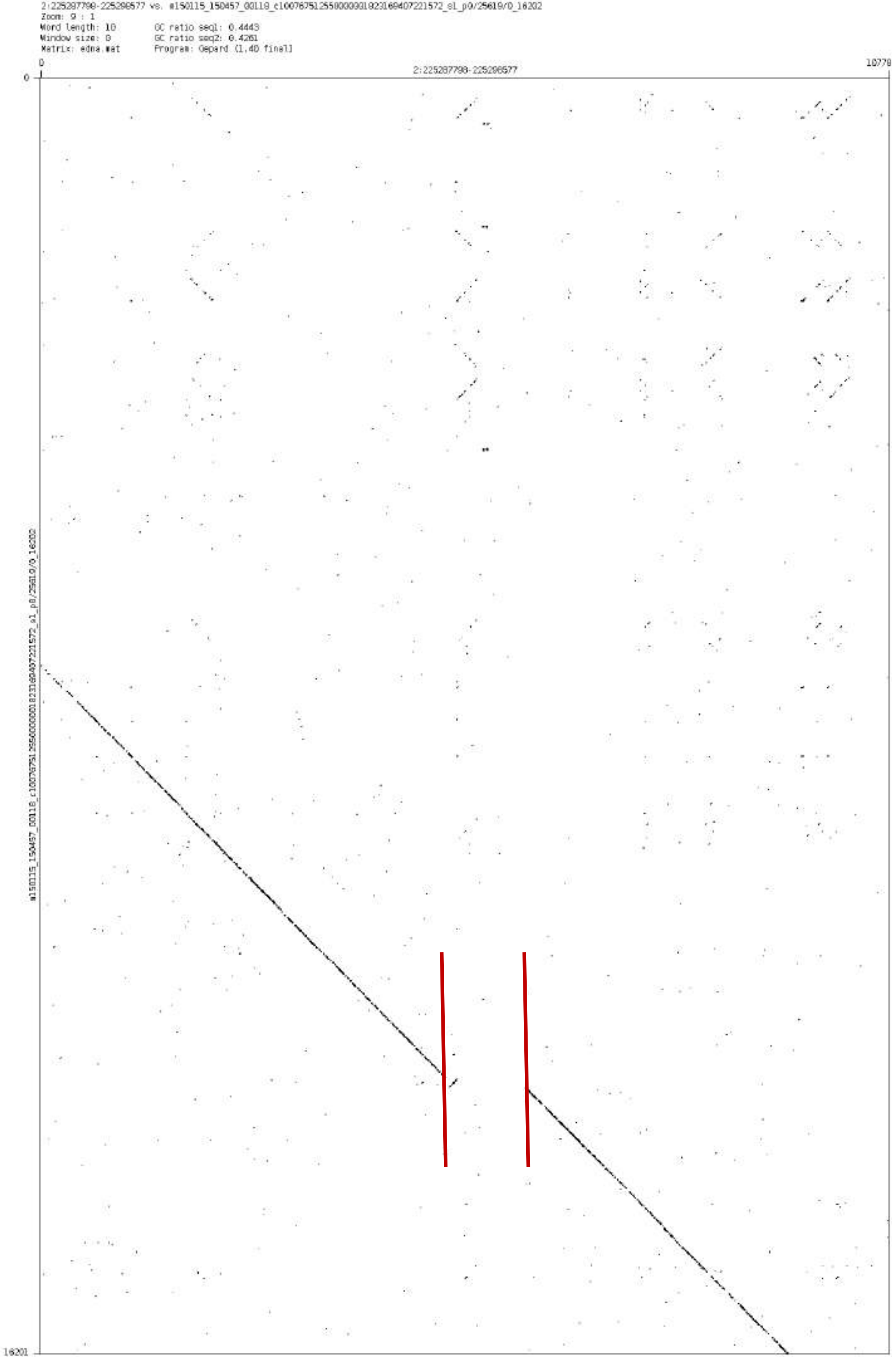
2:225287798-225298577 vs. m150115_150457_00118_c100767512550000001823169407221572_s1_p0/25619/0_16202
Zoom: 9 : 1
Word length: 10
Window size: 0
Matrix: edna.mat
GC ratio seq1: 0.4443
GC ratio seq2: 0.4261
Program: Gepard (1.40 final)
0
10779
2:225287798-225298577
0
16201

chr4:32065823-32071044

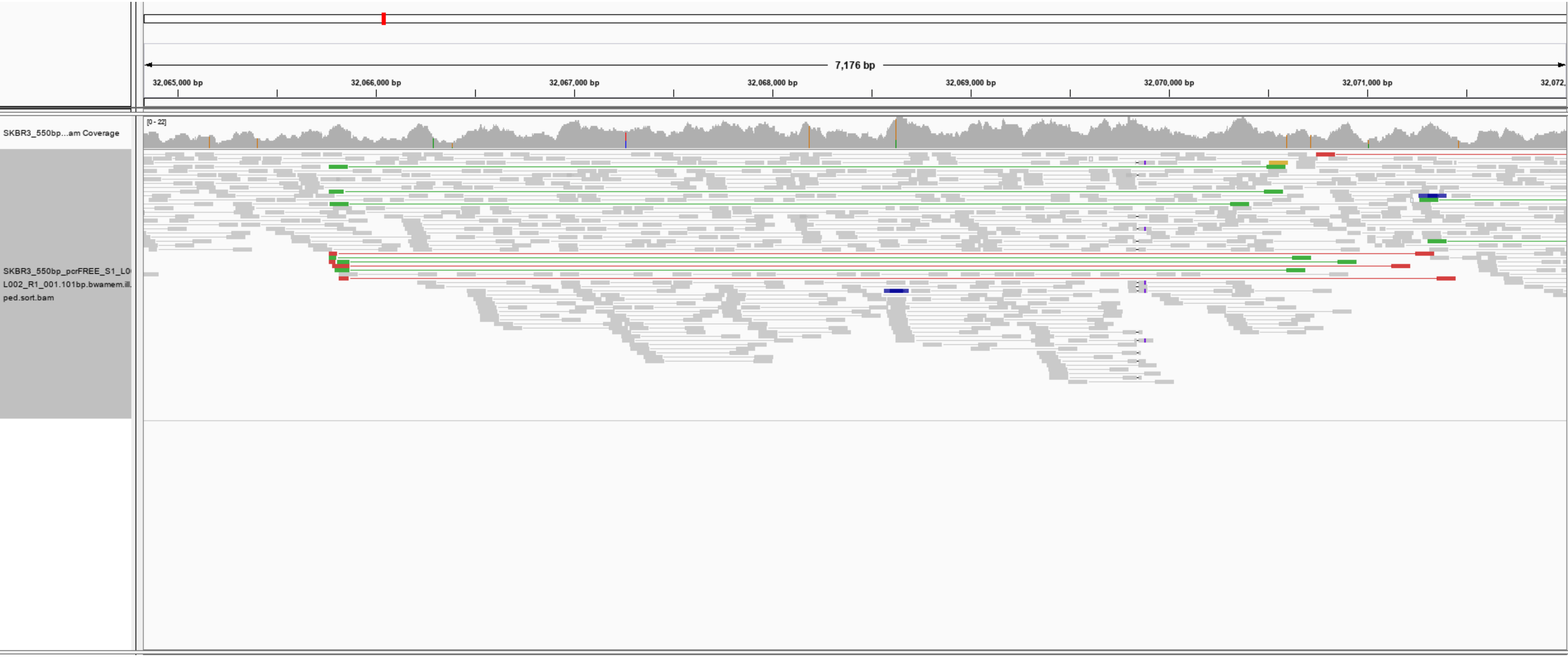

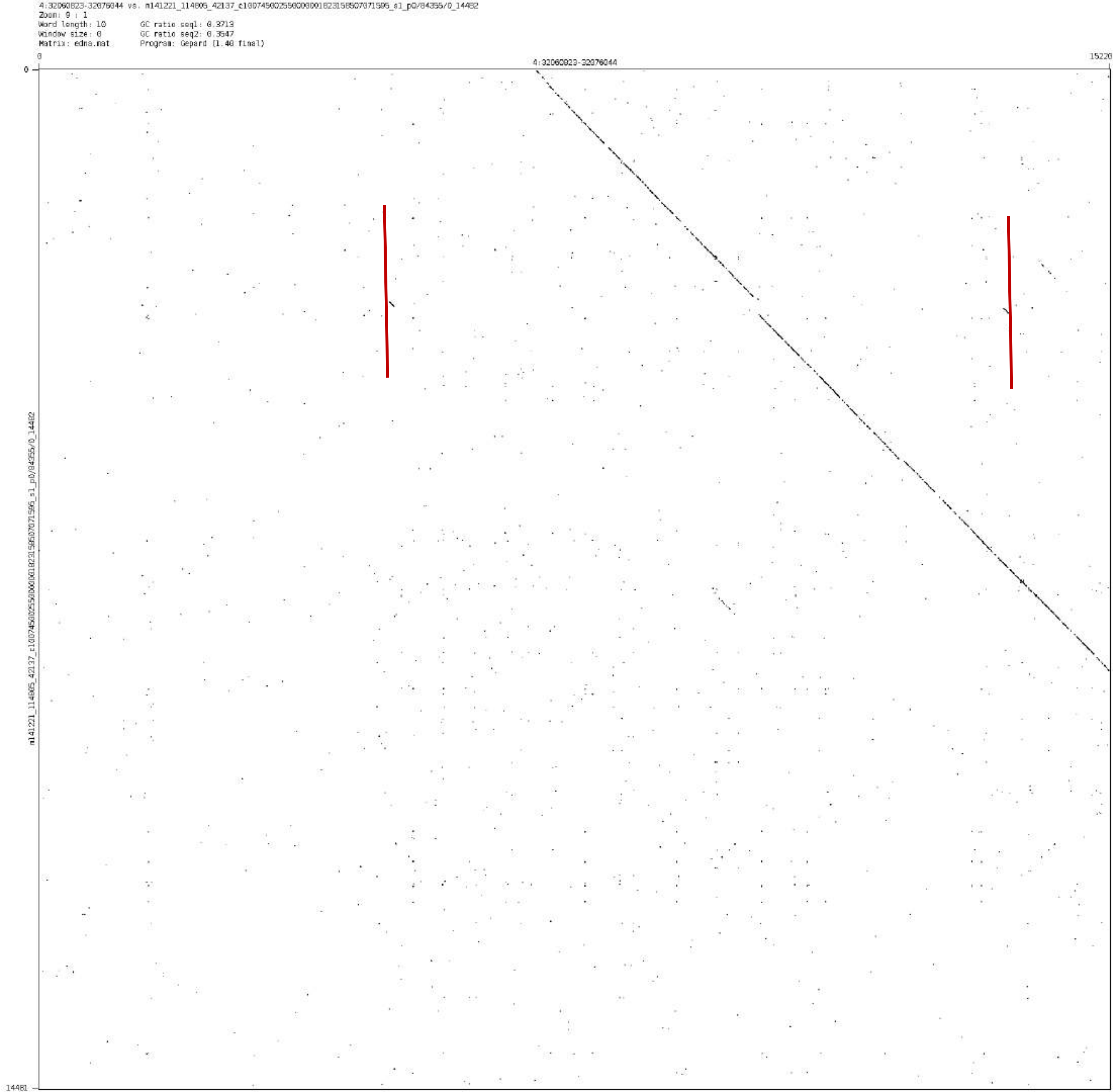
4:32060823-32076044 vs. m141221_114805_42137_c100745002550000001823158507071595_s1_p0/84355/0_14482
Zoom: 9 : 1
Word length: 10
Window size: 0
Matrix: edna.mat
GC ratio seq1: 0.3713
GC ratio seq2: 0.3547
Program: Gepard (1.40 final)
0
4:32060823-32076044
15220
0
m141221_114805_42137_c100745002550000001823158507071595_s1_p0/84355/0_14482
14481

chr4:93568459-93570189

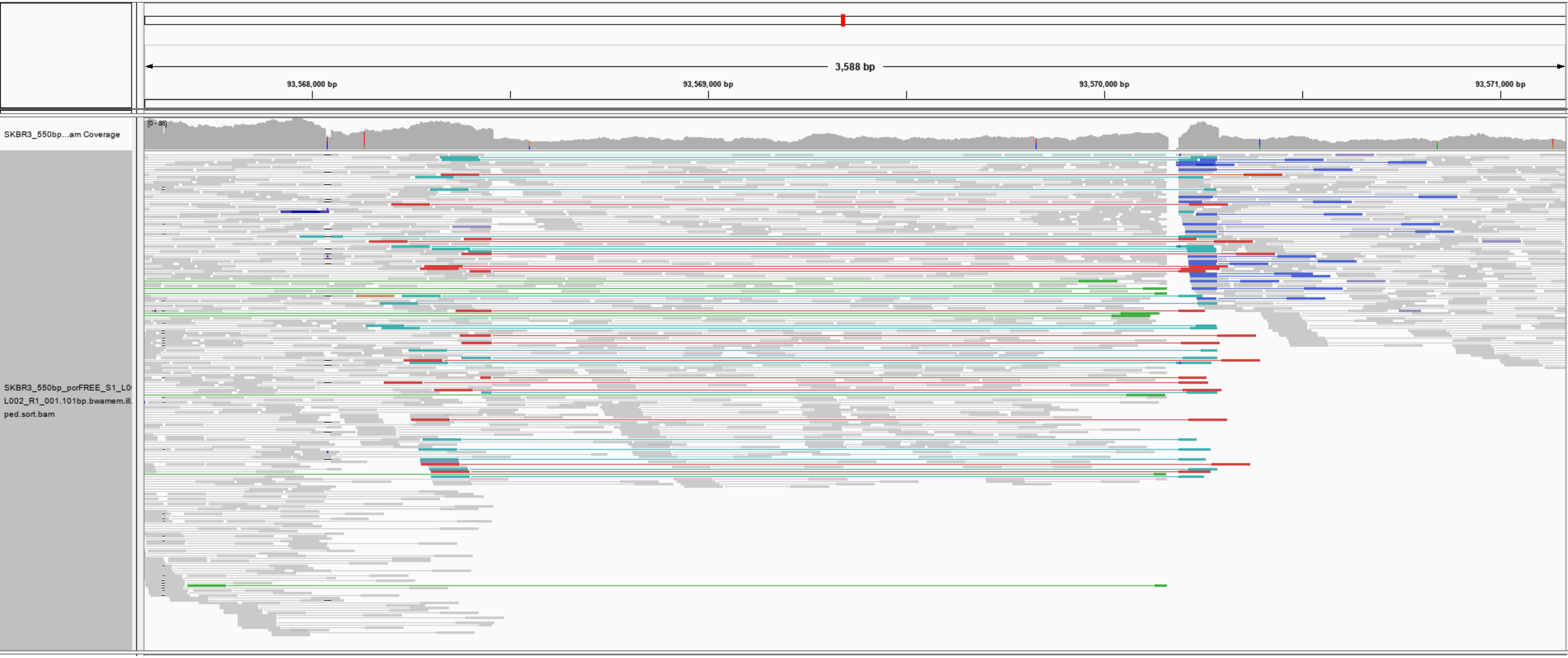

4:93563459-93575189 vs. m141212_181156_42137_c100744672550000001823159507071565_s1_p0/134761/21961_34542
Zoom: 7 : 1
Word length: 10 GC ratio seq1: 0.3373
Window size: 0 GC ratio seq2: 0.3263
Matrix: edna.mat Program: Gepard (1.40 final)

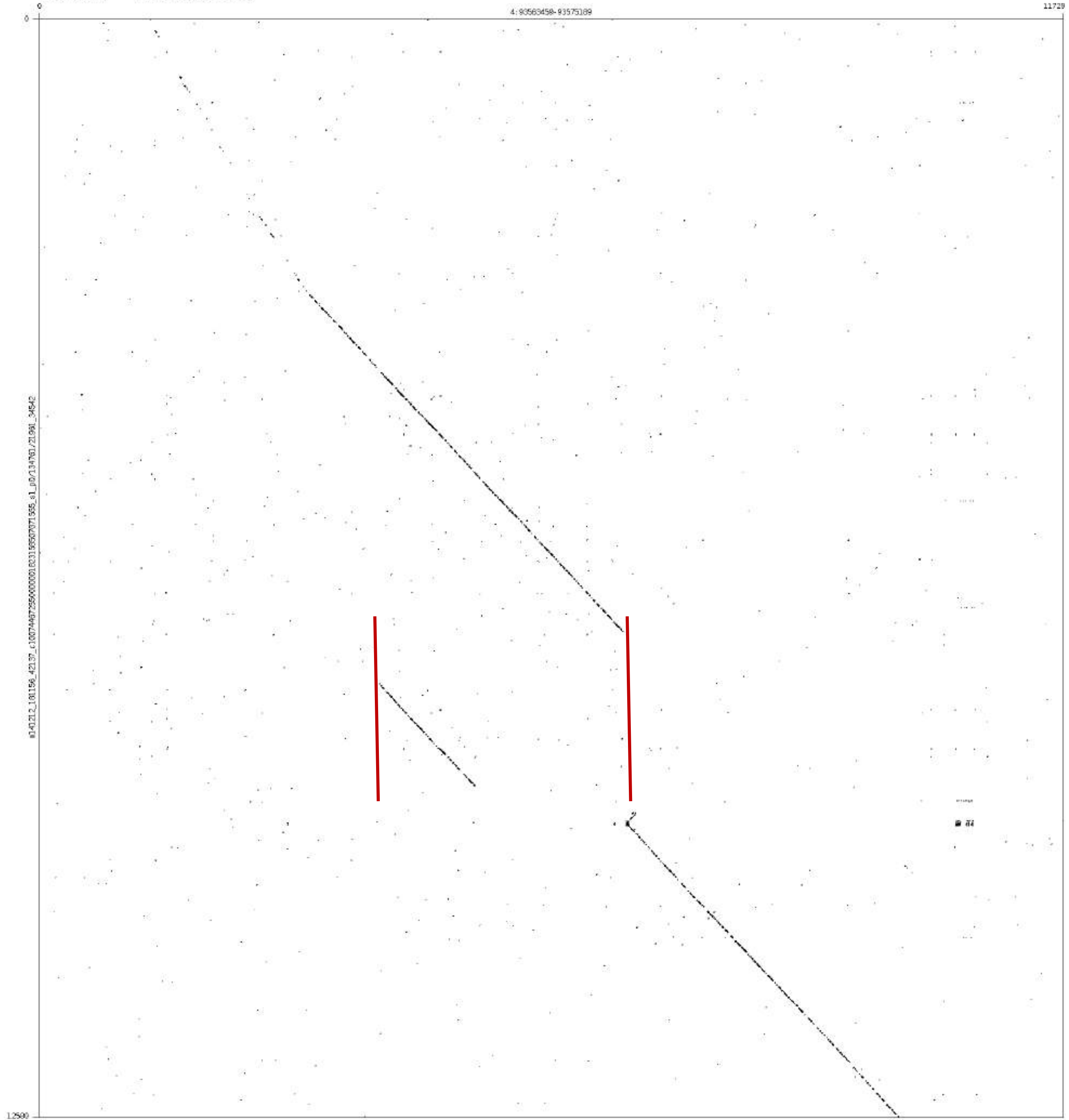

chr5:151511018-151516780

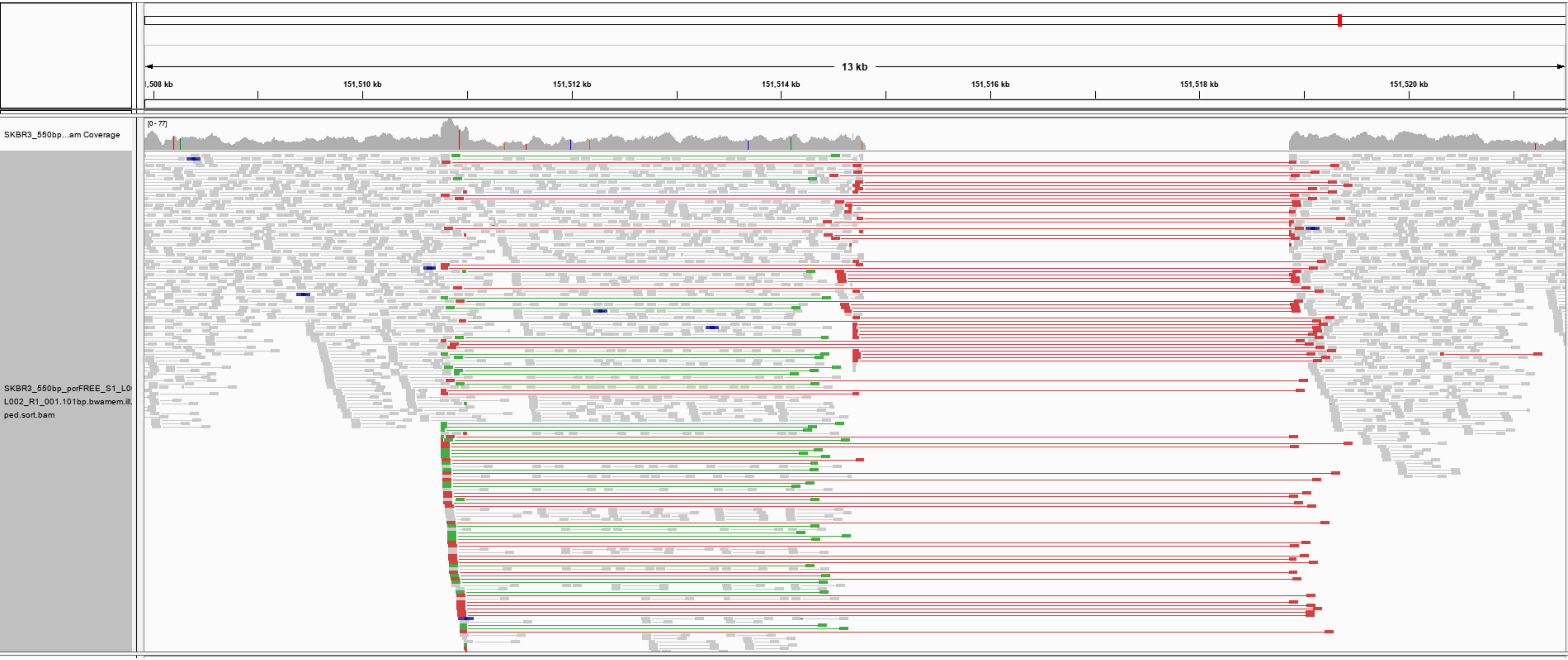

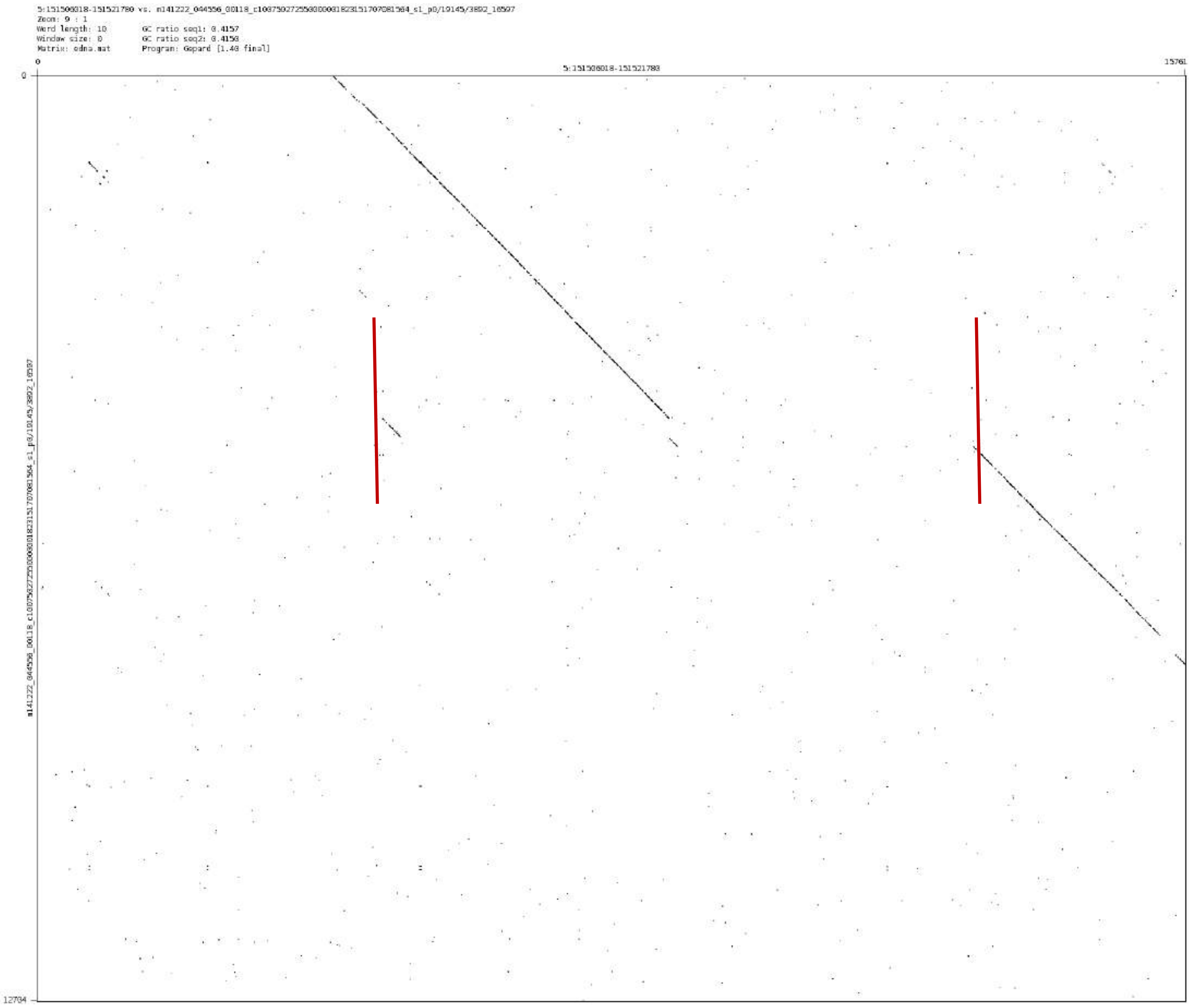
5:151506018-151521780 vs. m141222_044556_00118_c100750272550000001823151707081564_s1_p0/19145/3892_16597
Zoom: 9 : 1
Word length: 10
Window size: 0
Matrix: edna.mat
GC ratio seq1: 0.4157
GC ratio seq2: 0.4158
Program: Gepard [1.40 final]
0
5:151506018-151521780
15761
0
12704
m141222_044556_00118_c100750272550000001823151707081564_s1_p0/19145/3892_16597

chr7:154876776-154880629

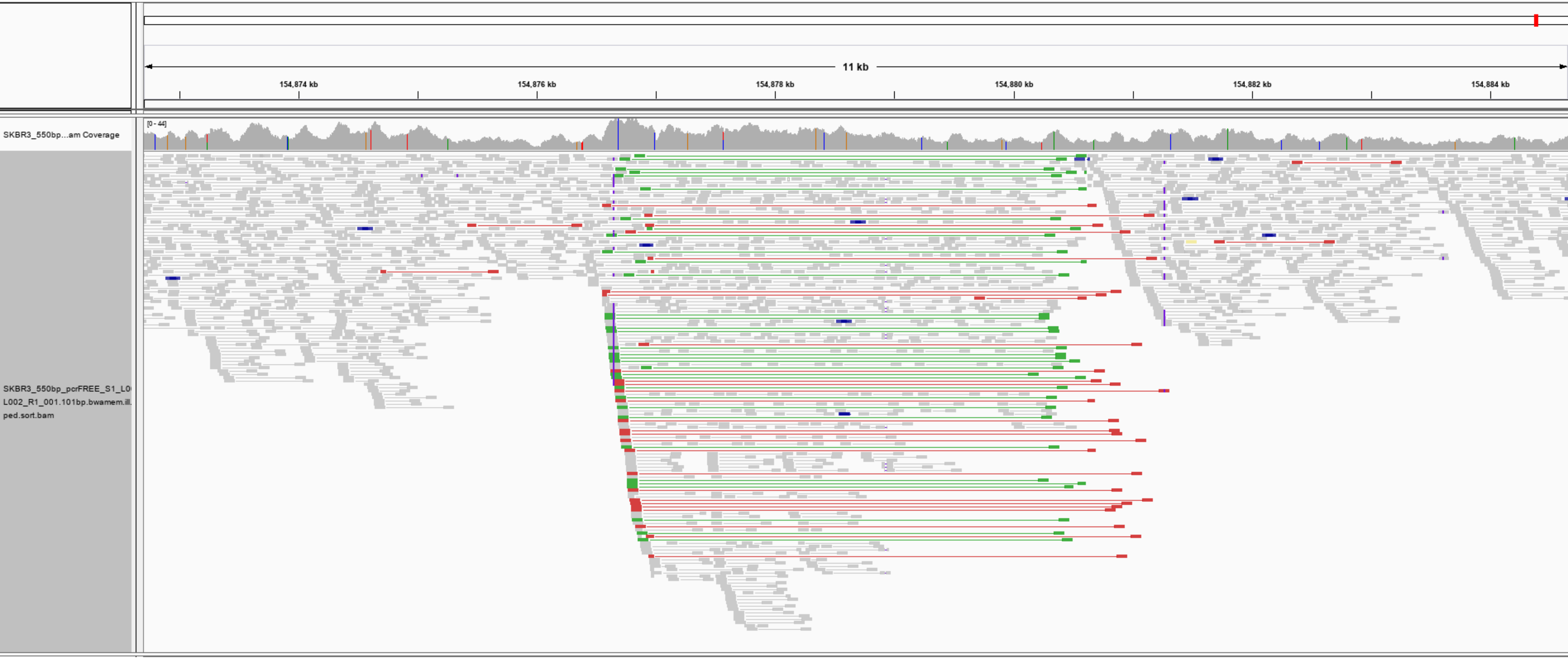

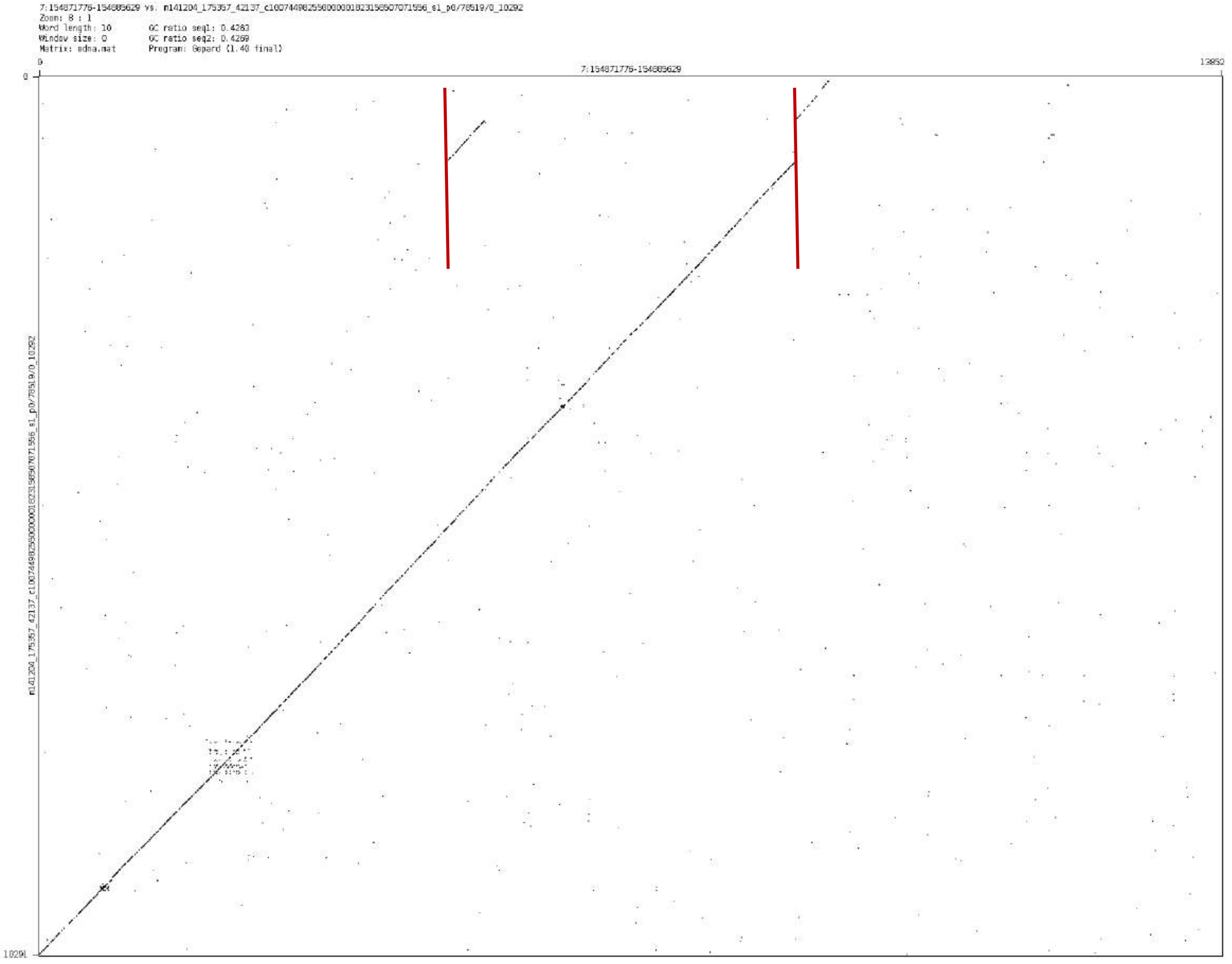
7:154871776-154885629 vs. m141204_175357_42137_c100744982550000001823156507071556_s1_p0/78519/0_10292
Zoom: 8 : 1
Word length: 10
GC ratio seq1: 0.4263
Window size: 0
GC ratio seq2: 0.4269
Matrix: edna.mat
Program: Gepard (1.40 final)
0
7:154871776-154885629
13852
0
m141204_175357_42137_c100744982550000001823156507071556_s1_p0/78519/0_10292
10291

chr9:89154436-89155939

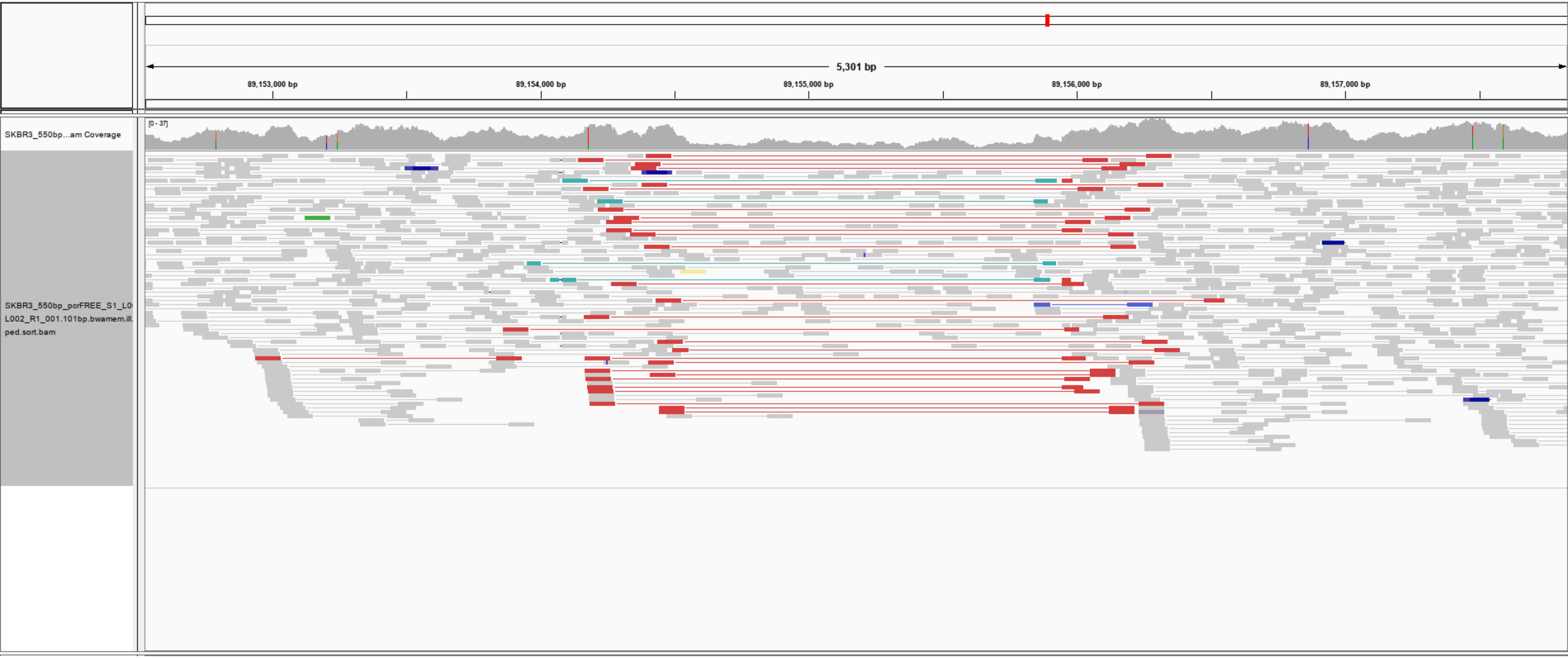

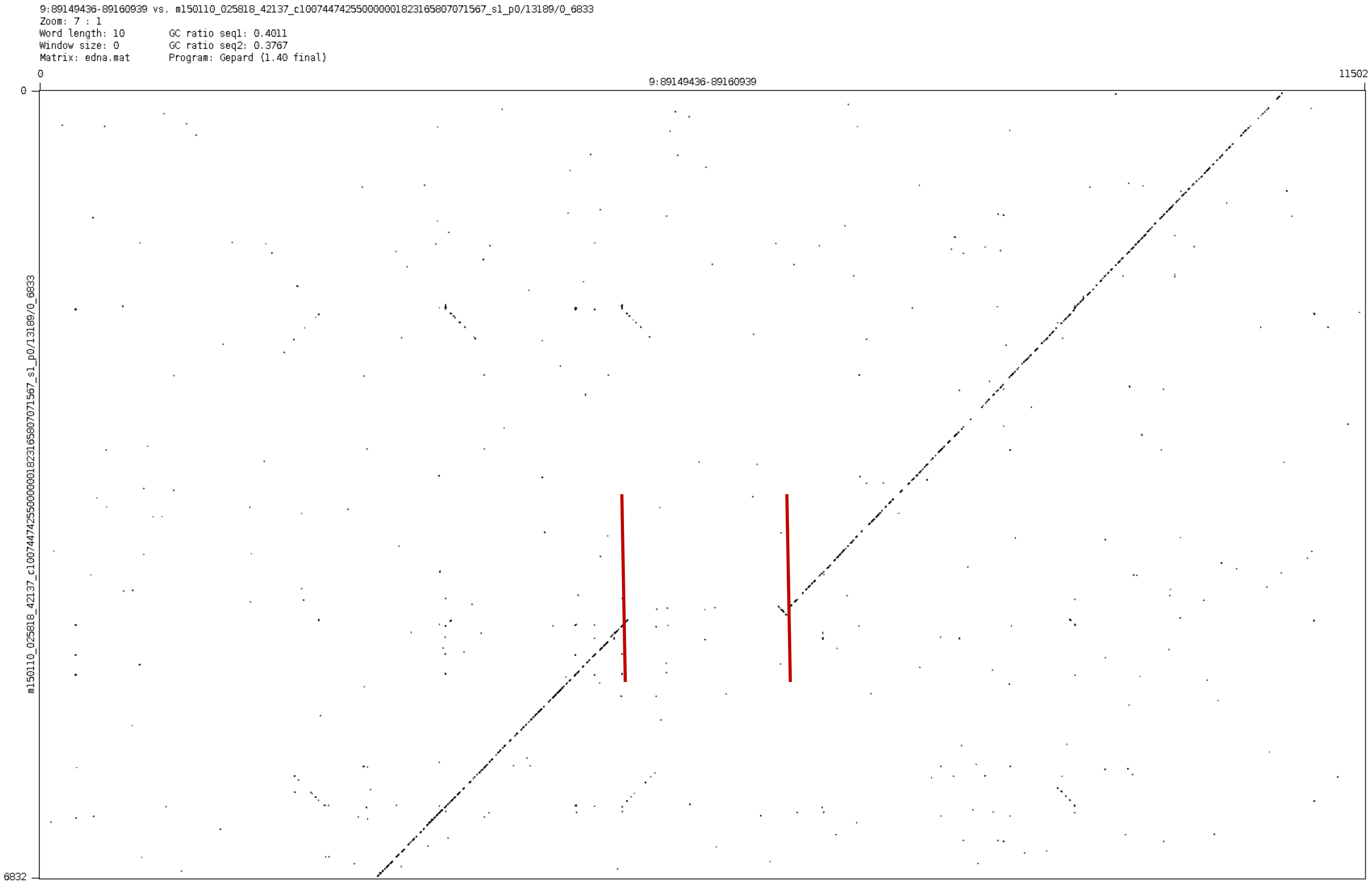
9:89149436-89160939 vs. m150110_025818_42137_c100744742550000001823165807071567_s1_p0/13189/0_6833
Zoom: 7 : 1
Word length: 10
GC ratio seq1: 0.4011
Window size: 0
GC ratio seq2: 0.3767
Matrix: edna.mat
Program: Gepard (1.40 final)
0
11502
9:89149436-89160939
0
6832
m150110_025818_42137_c100744742550000001823165807071567_s1_p0/13189/0_6833

chr9:112285937-112286755

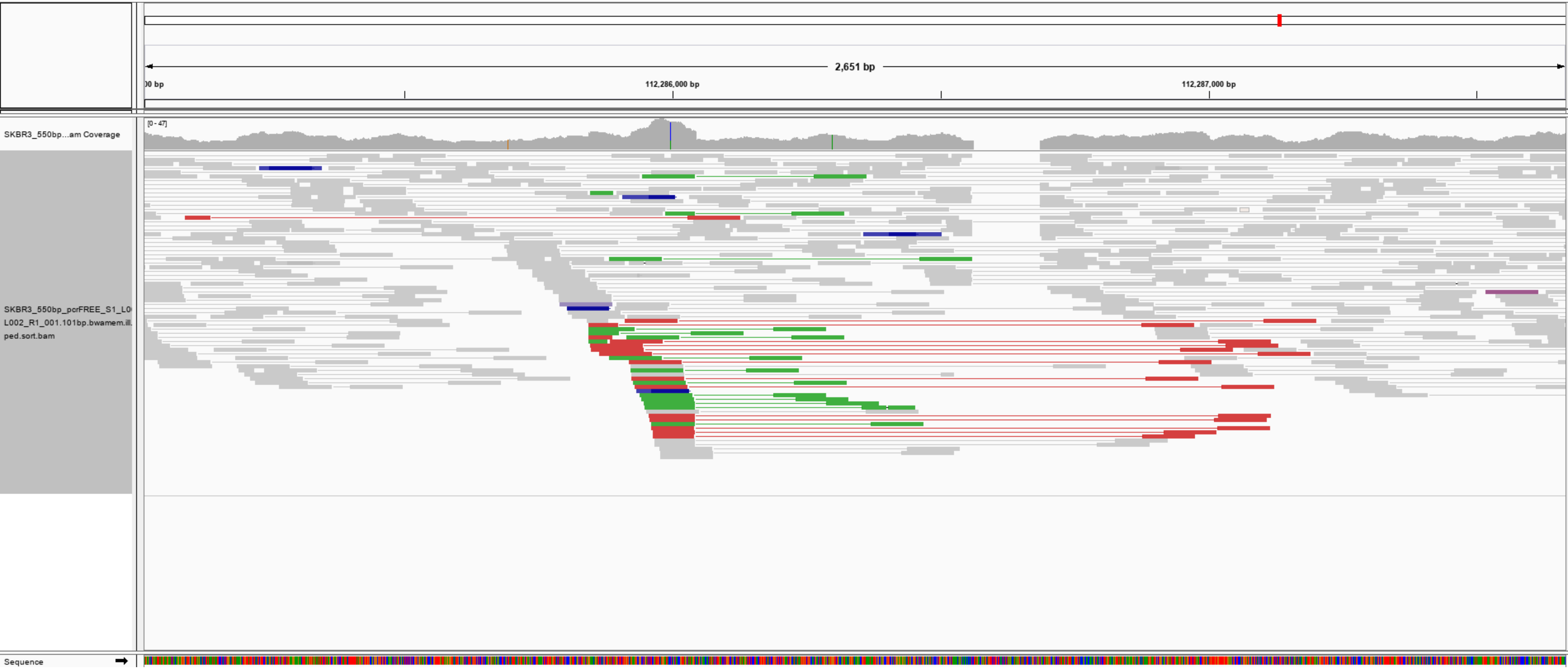

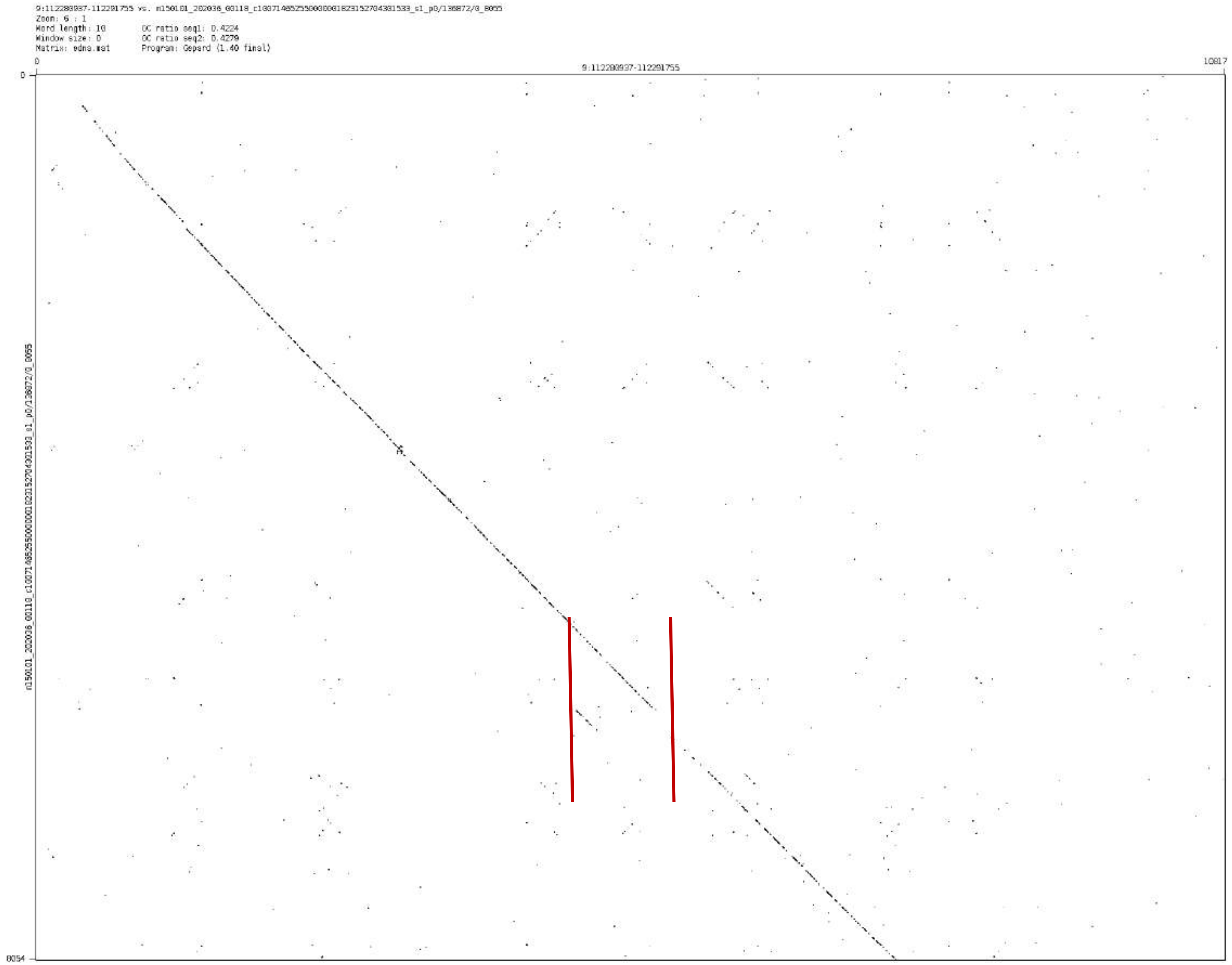
Zoom: 6 : 1
Word length: 10
Window size: 0
Matrix: edna.mat
GC ratio seq1: 0.4224
GC ratio seq2: 0.4279
Program: Gepard (1.40 final)
0
10817
8054

chr10:127191013-127197233

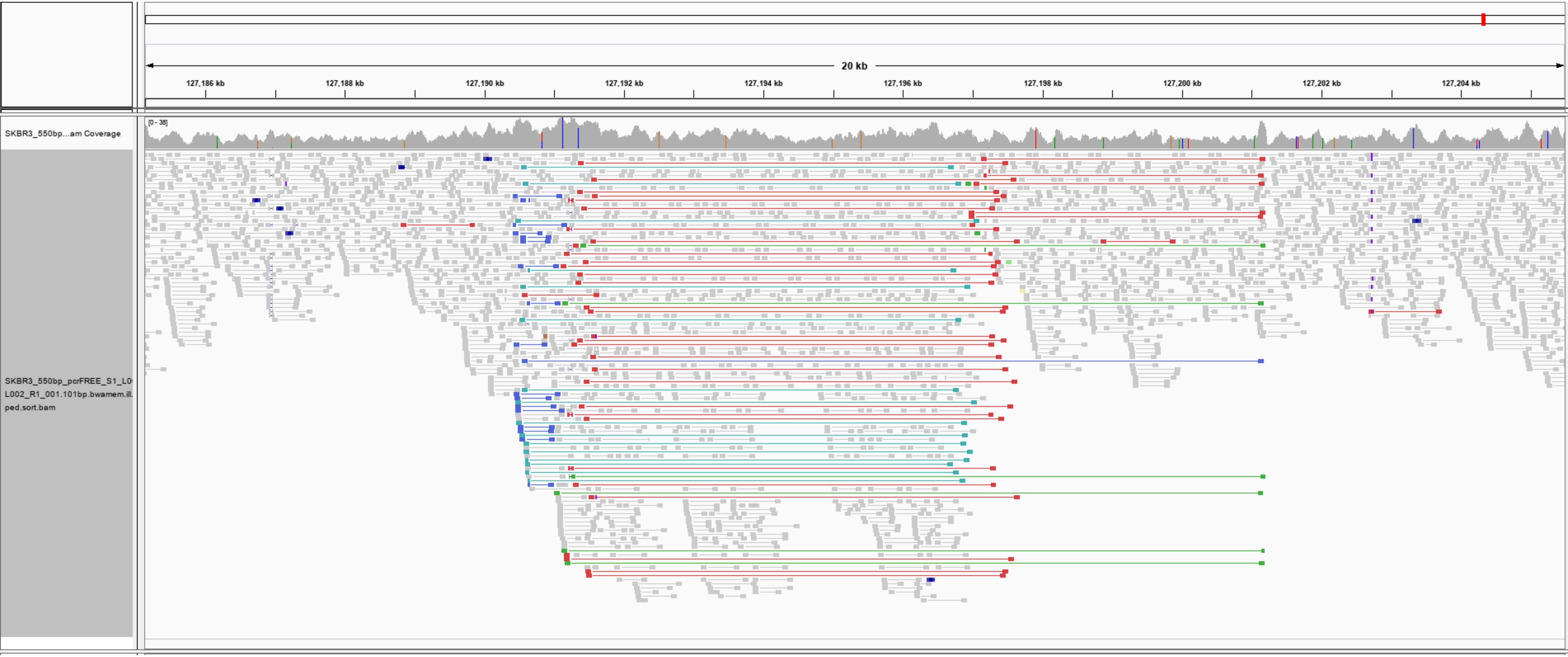

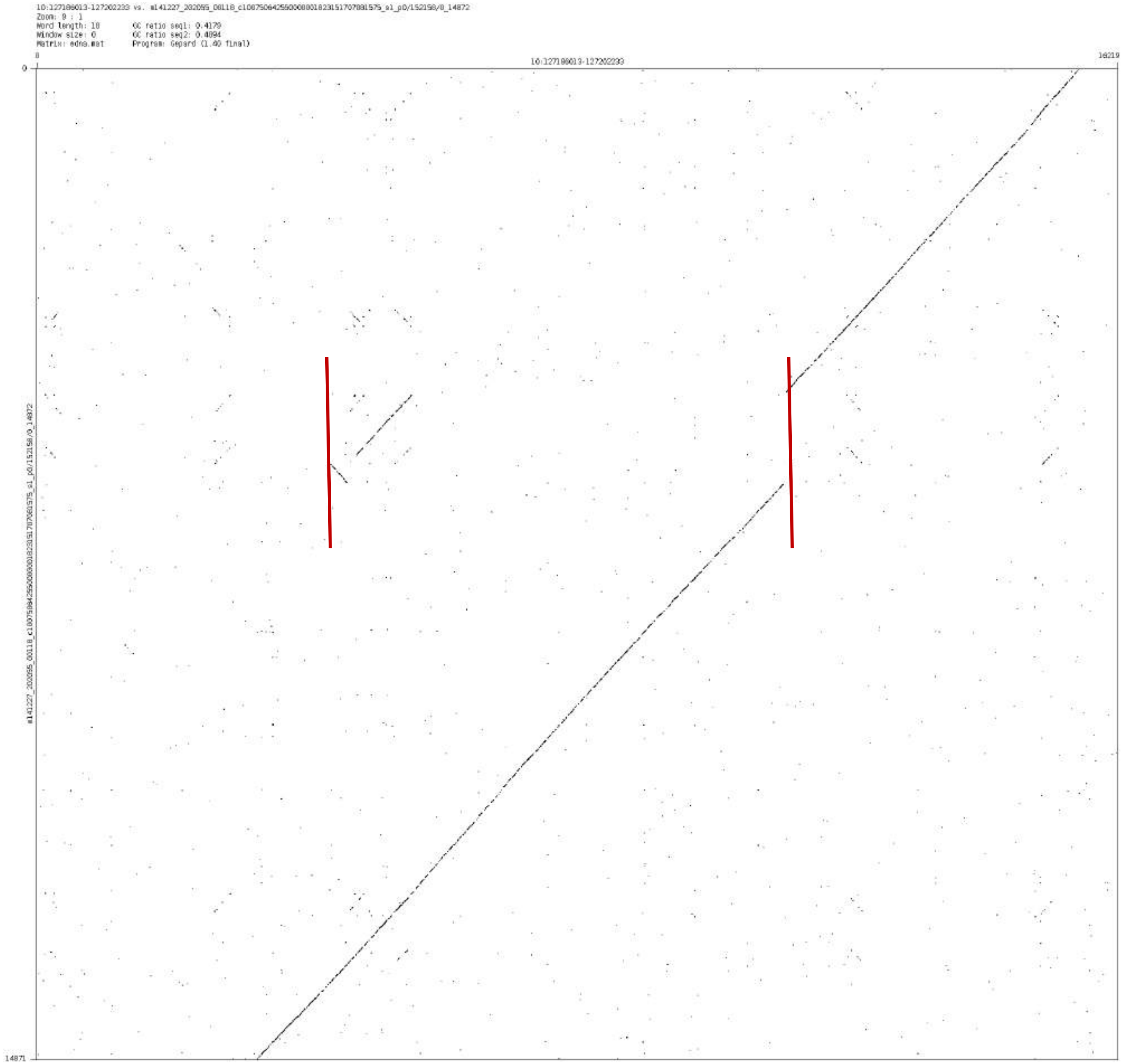
10:127186013-127202233 vs. m141227_202055_00118_c100750642550000001823151707081575_s1_p0/152158/0_14872
Zoom: 9 : 1
Word length: 10
Window size: 0
Matrix: edna.mat
GC ratio seq1: 0.4179
GC ratio seq2: 0.4094
Program: Gepard (1.40 final)
0
10:127186013-127202233
16219
0
14871

chr11:63698921-63701800

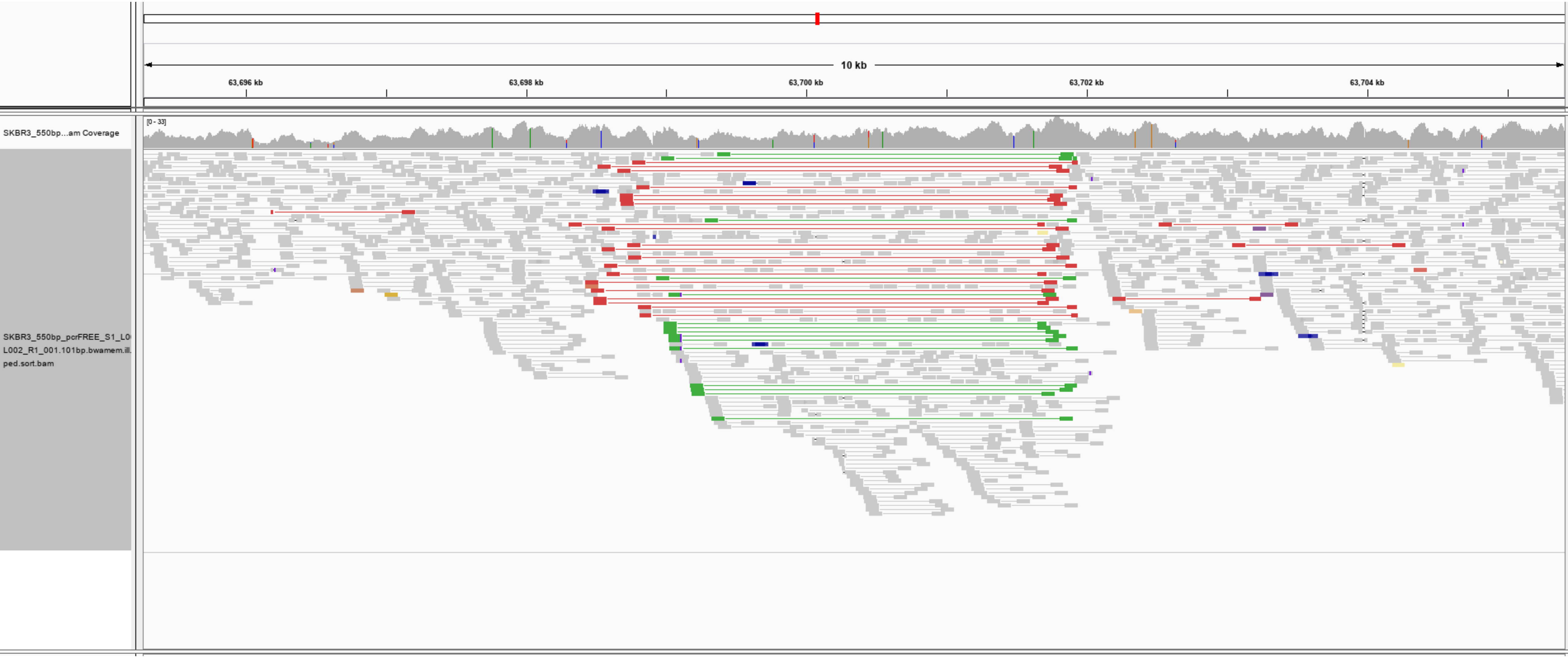

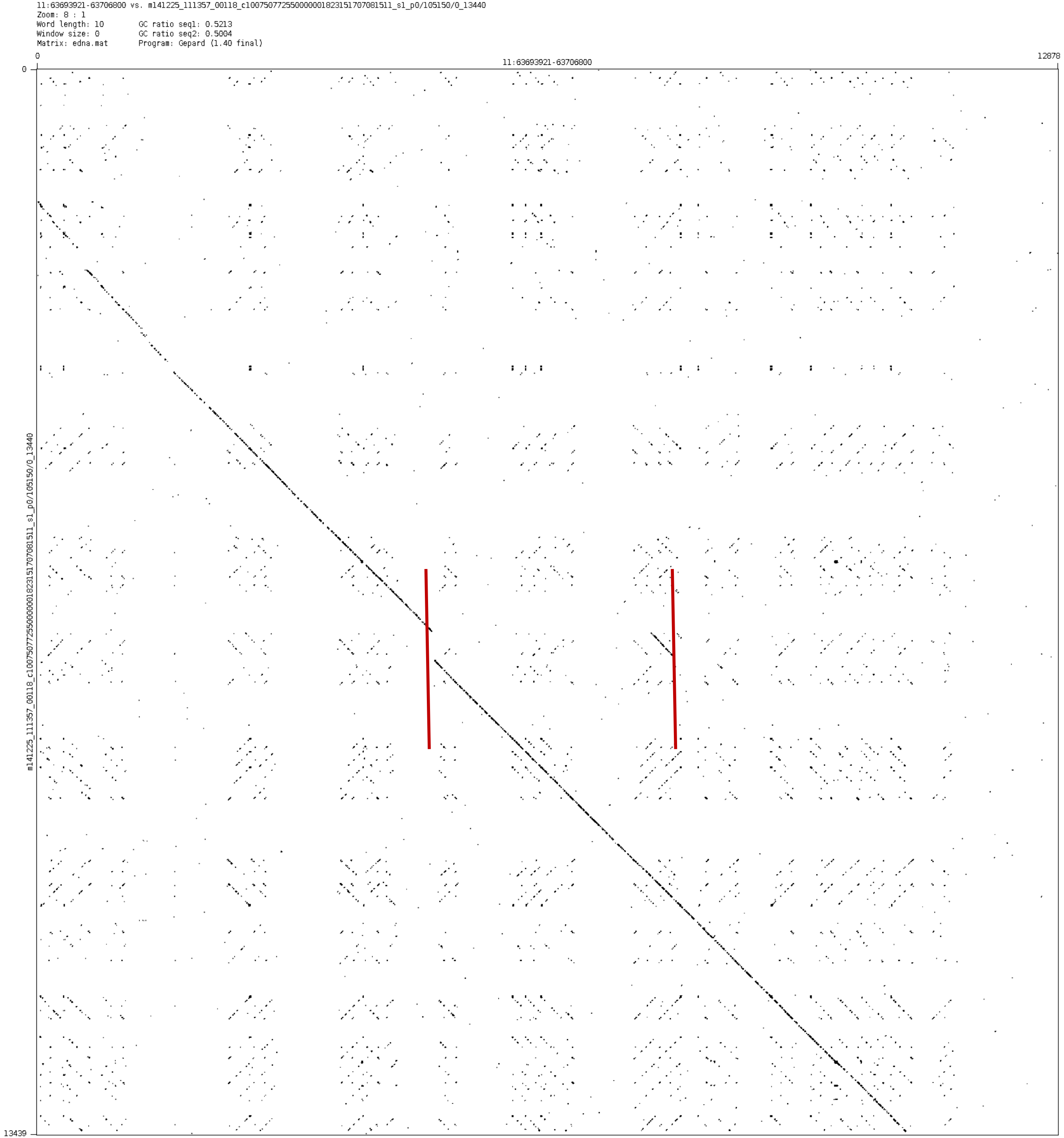

11:63693921-63706800 vs. m141225_111357_00118_c100750772550000001823151707081511_s1_p0/105150/0_13440
Zoom: 8 : 1
Word length: 10
GC ratio seq1: 0.5213
Window size: 0
GC ratio seq2: 0.5004
Matrix: edna.mat
Program: Gepard (1.40 final)
0
11:63693921-63706800
12878
0
m141225_111357_00118_c100750772550000001823151707081511_s1_p0/105150/0_13440
13439

chr11:78039089-78042394

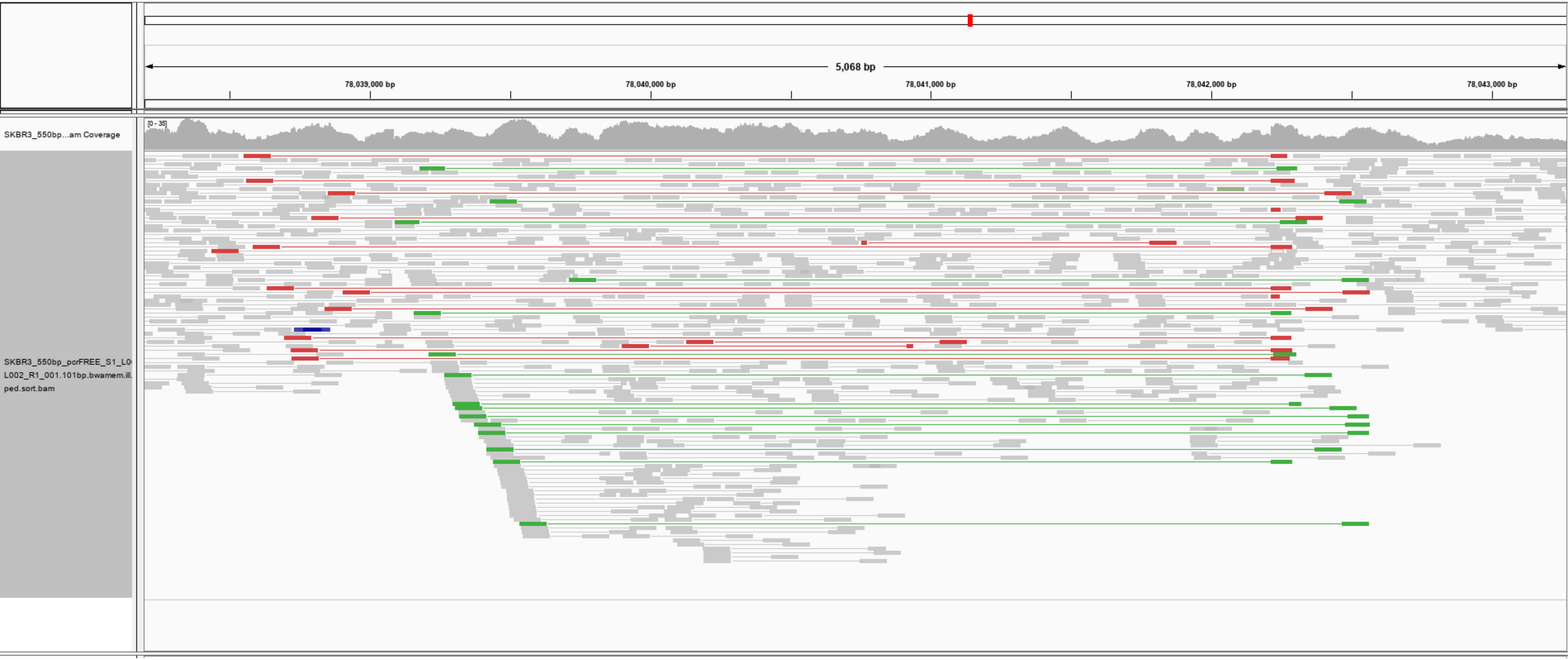

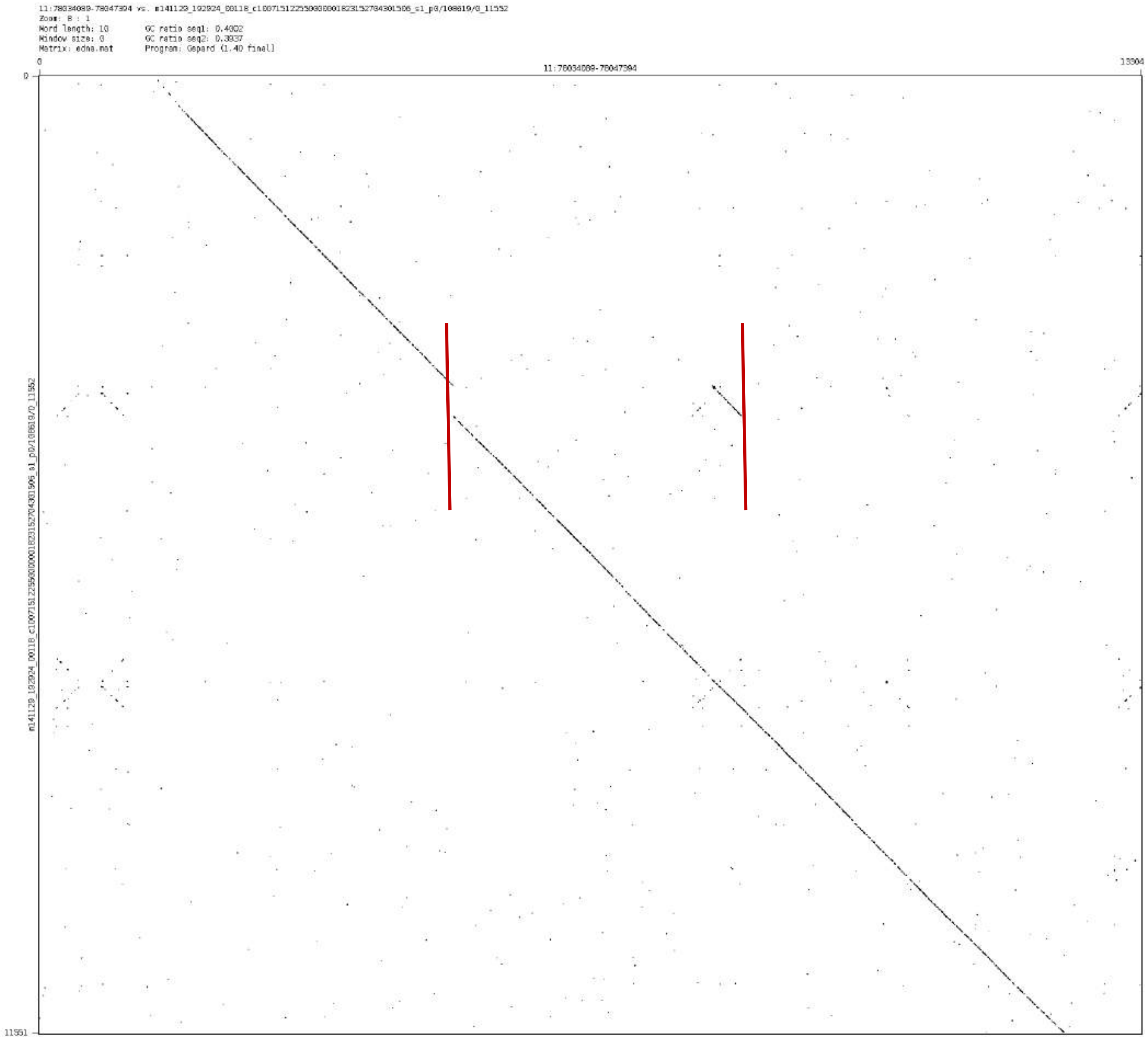

Zoom: 8 : 1
Word length: 10
Window size: 0
Matrix: edna.mat
GC ratio seq1: 0.4002
GC ratio seq2: 0.3937
Program: Gepard (1.40 final)
0
11:78034089-78047394
13304
0
11551

chr14:40113933-40114921

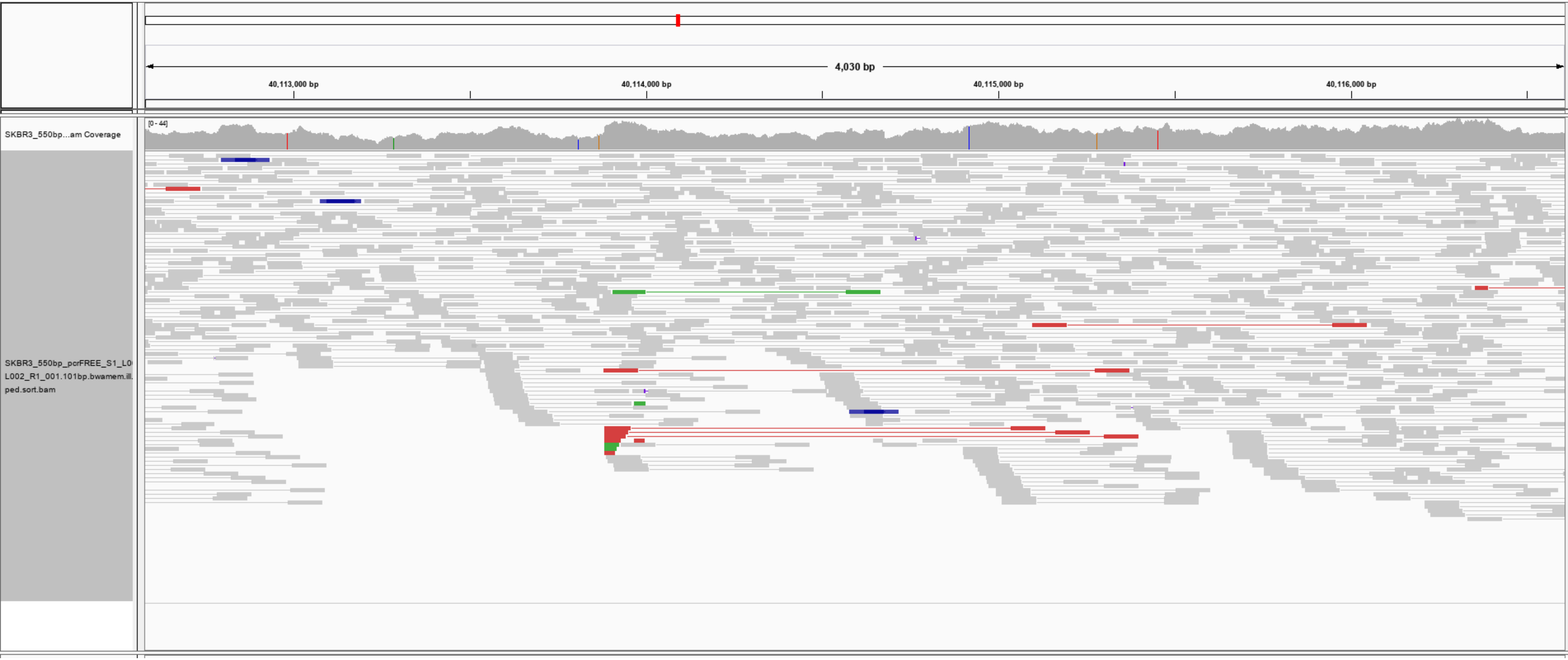

14_40113933_40114921.27

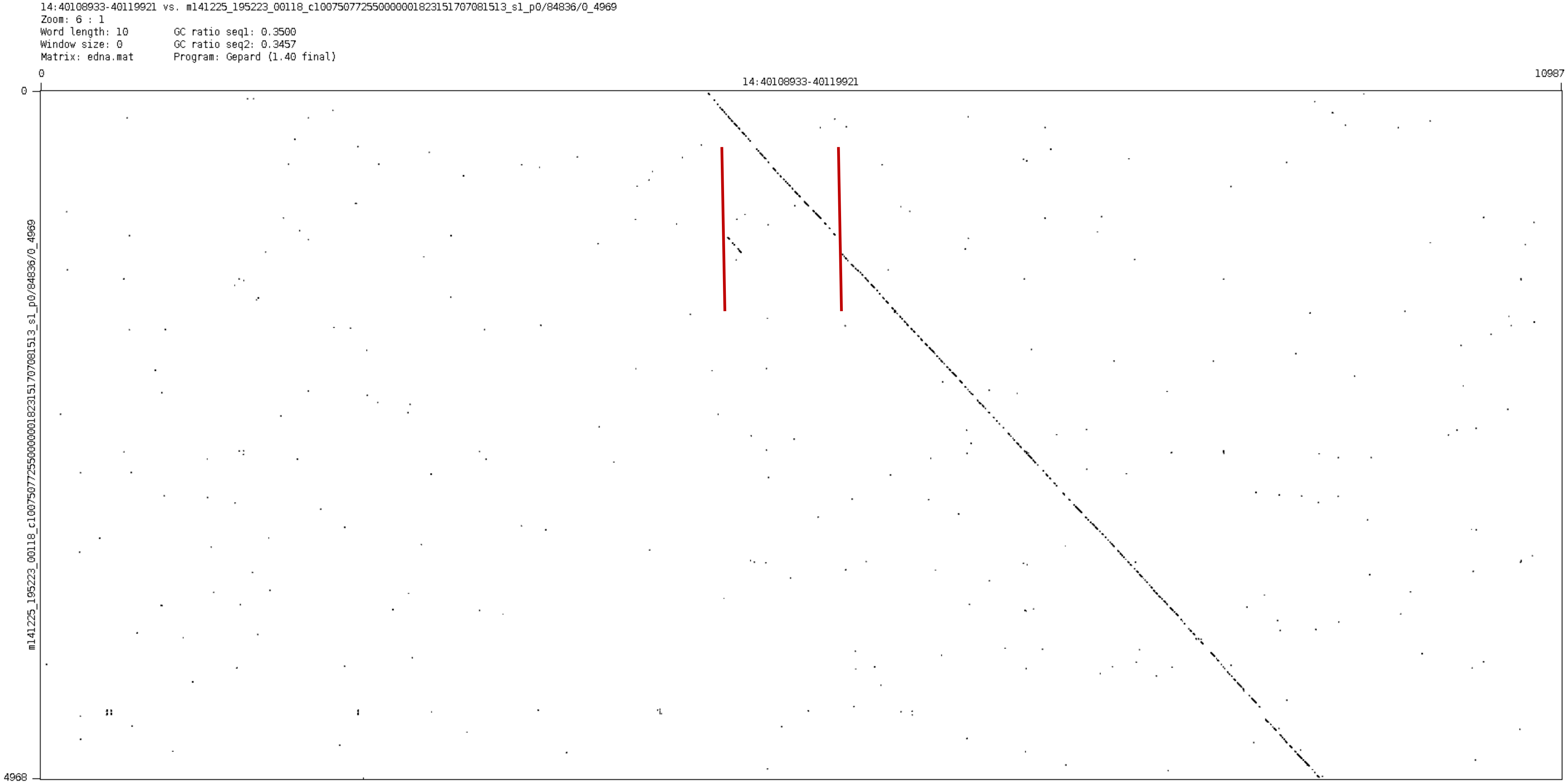

chr18:11509472-11511497

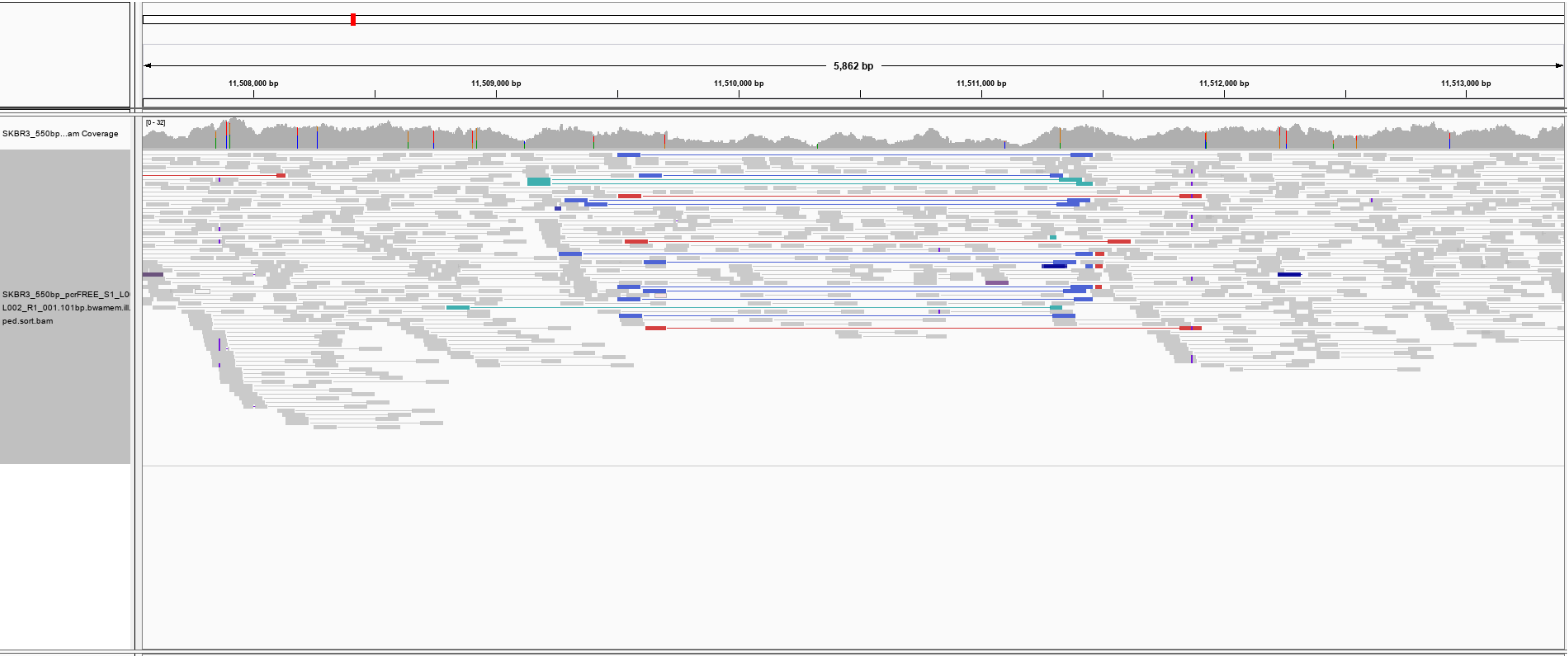

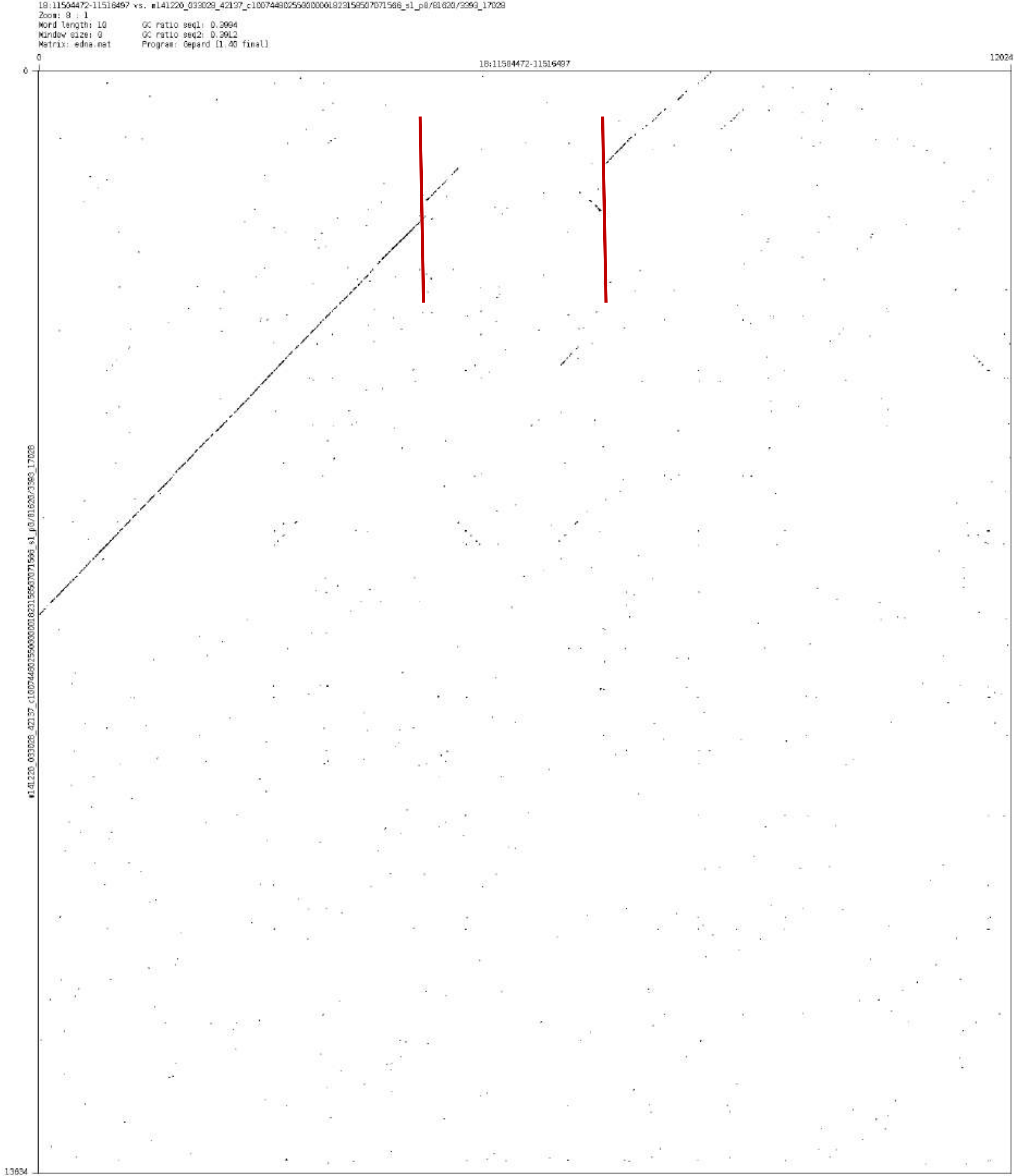
18:11504472-11516497 vs. m141220_033029_42137_c100744802550000001823158507071586_s1_p0/81620/3393_17028
Zoom: 8 : 1
Word length: 10
Window size: 0
Matrix: edna.mat
GC ratio seq1: 0.3994
GC ratio seq2: 0.3912
Program: Gepard [1.40 final]
0
18:11504472-11516497
12024
0
13634

chr18:69711908-69712979

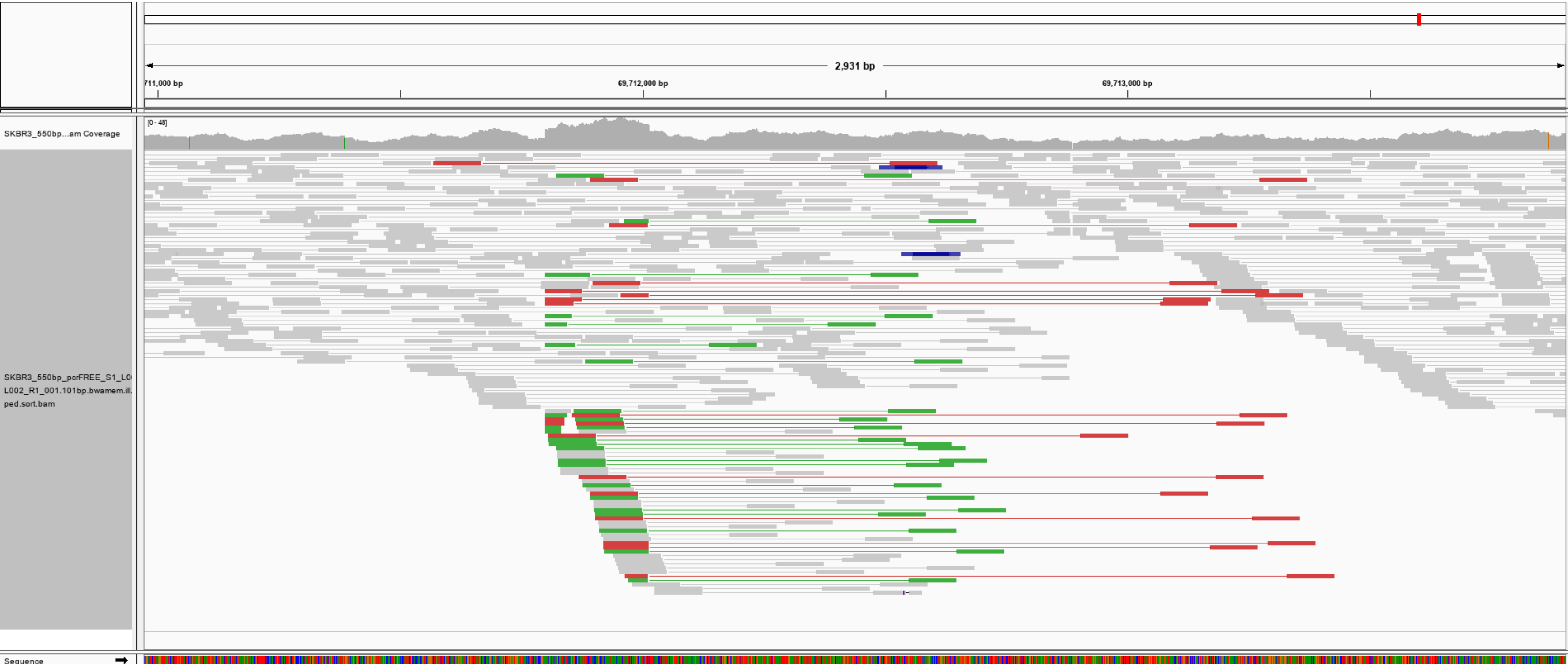

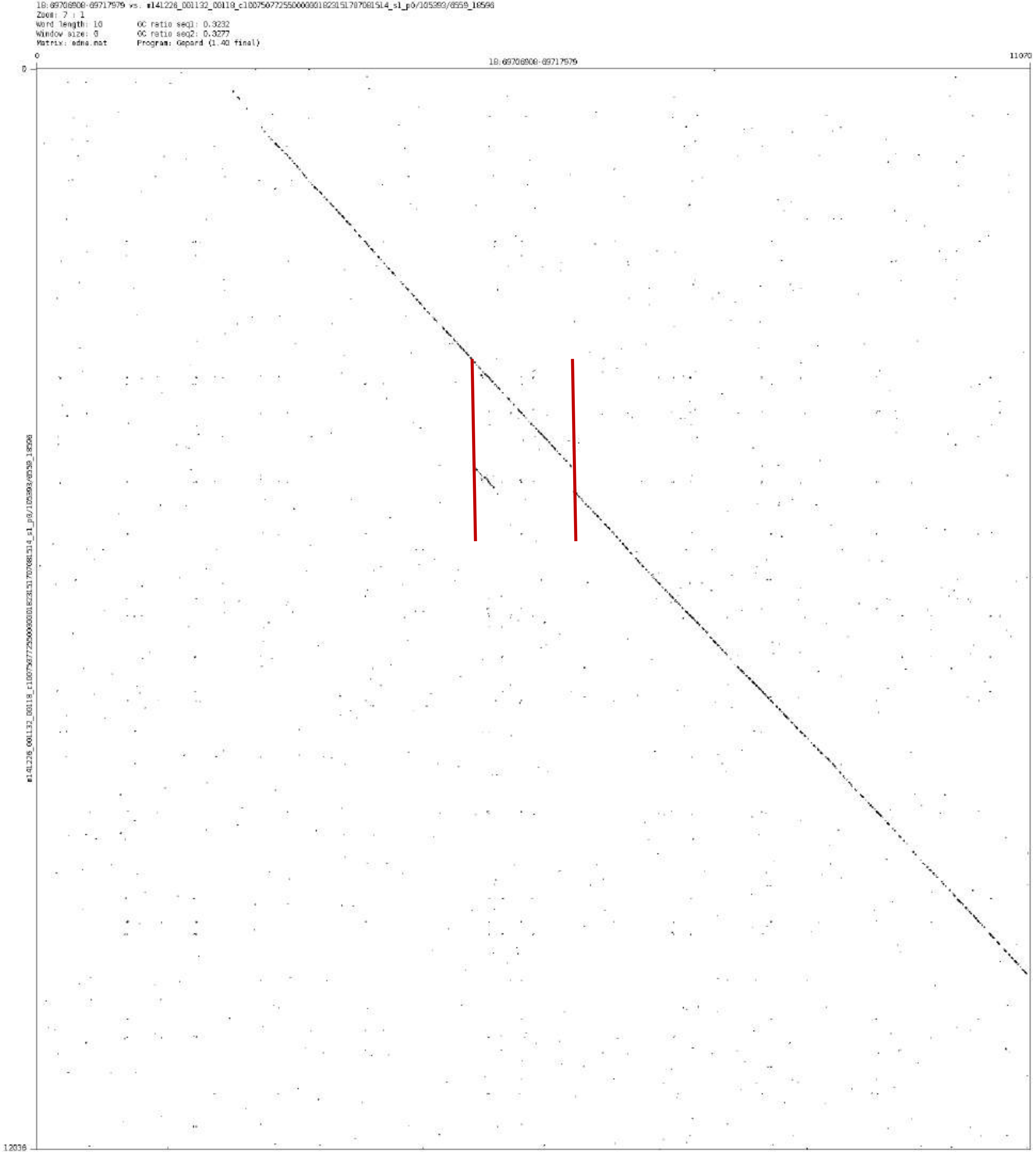
18:69706908-69717979 vs. m141226_001132_00118_c100750772550000001823151707081514_s1_p0/105393/8559_18596
Zoom: 7 : 1
Word length: 10
Window size: 0
Matrix: edna.mat
GC ratio seq1: 0.3232
GC ratio seq2: 0.3277
Program: Gepard (1.40 final)
0
11070
18:69706908-69717979
0
12036

chr21:22783456-22784516

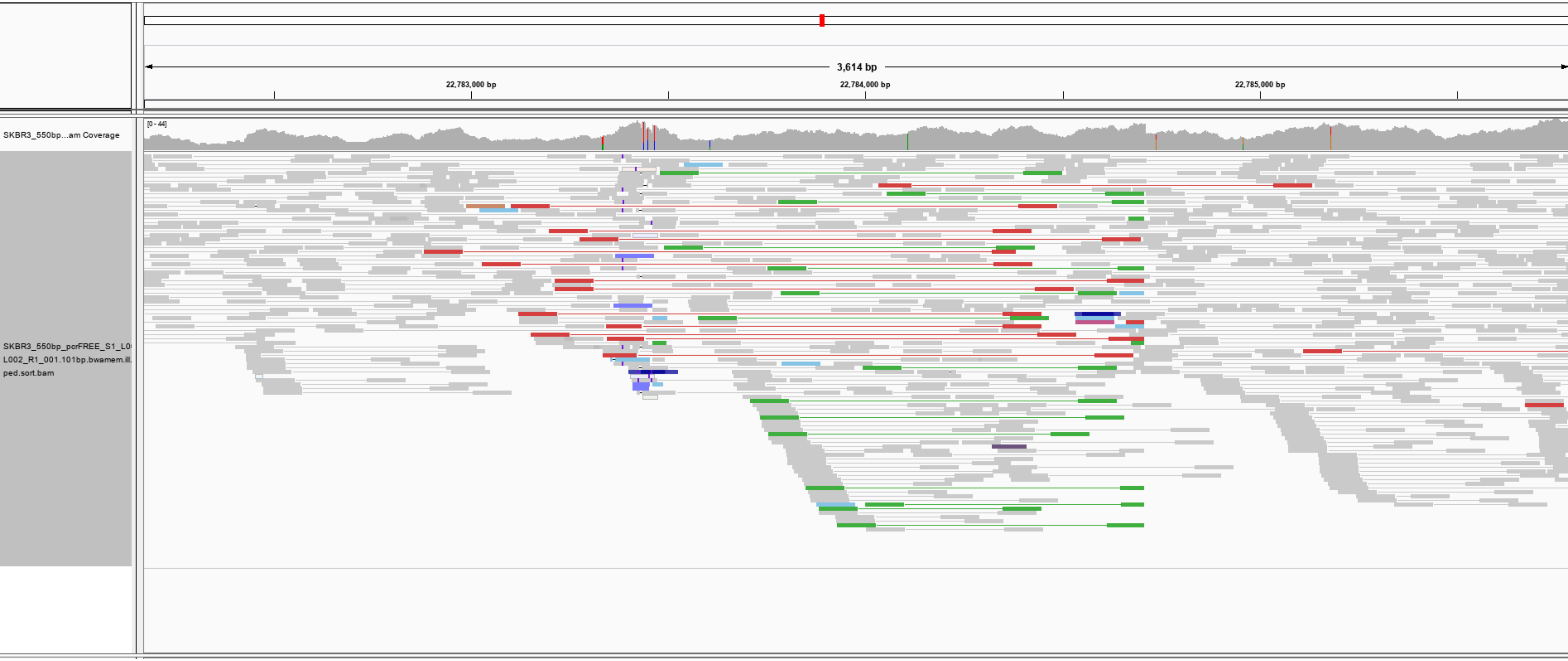

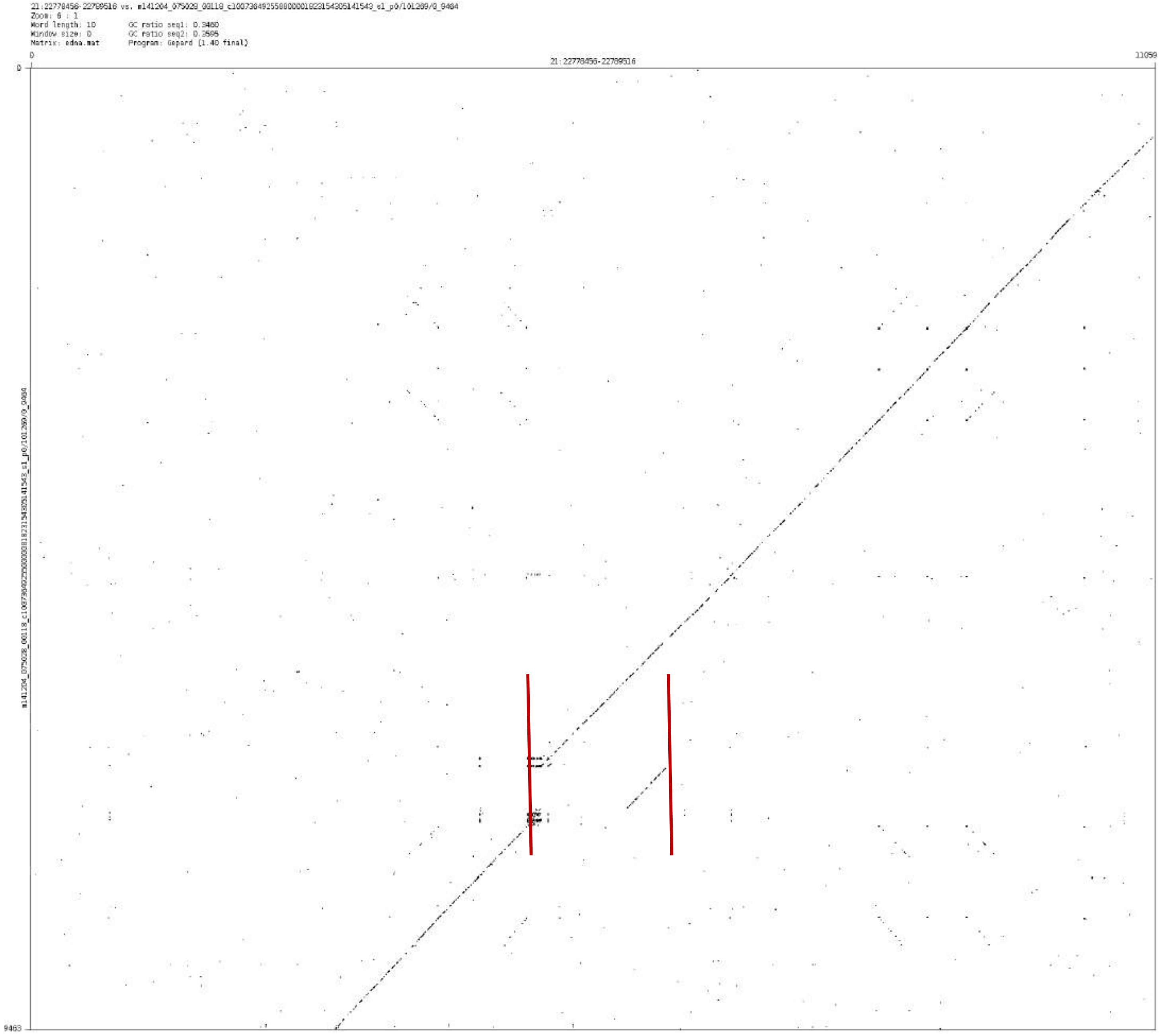
21:22778456-22789516 vs. m141204_075029_00118_c100738492550000001823154305141543_s1_p0/101289/0_9484
Zoom: 6 : 1
Word length: 10
Window size: 0
Matrix: edna.mat
GC ratio seq1: 0.3480
GC ratio seq2: 0.3585
Program: Gepard (1.40 final)
0
21:22778456-22789516
11059
0
9483
