# Supplementary material for: Mako: A Graph-based Pattern Growth Approach to Detect Complex Structural Variants
Source: Genomics Proteomics Bioinformatics. 2021 Jul 3;20(1):205–18. doi: 10.1016/j.gpb.2021.03.007 (PMC9510932; doi:10.1016/j.gpb.2021.03.007)

PCR results and visualization of CSV breakpoints validated through Sanger sequencing

PCR electrophoretic bands

M: DL5000 DNA marker. From bottom to top is: 100bp, 250bp, 500bp, 750bp, 1,000bp, 1,500bp, 2,000bp, 3,000bp and 5,000bp.
Red arrow: PCR product selected for sequencing with bright electrophoretic bands of targeted sizes

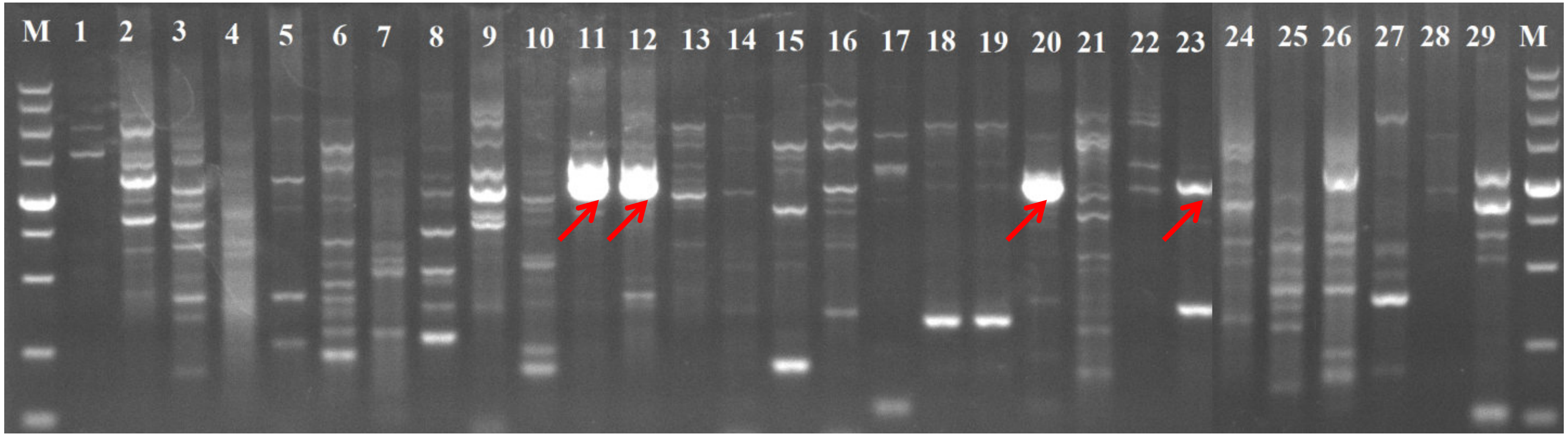

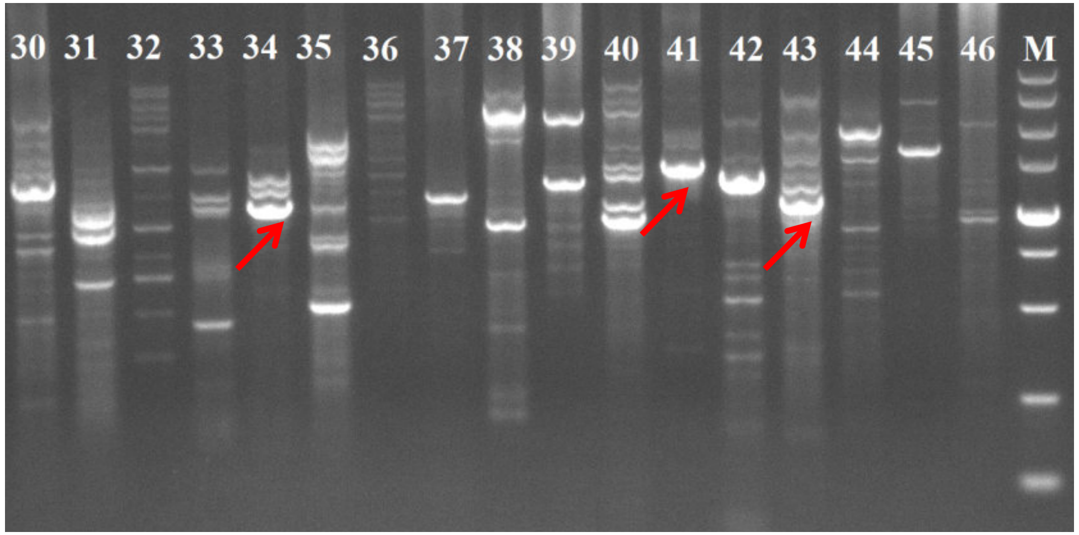
30 31 32 33 34 35 36 37 38 39 40 41 42 43 44 45 46 M

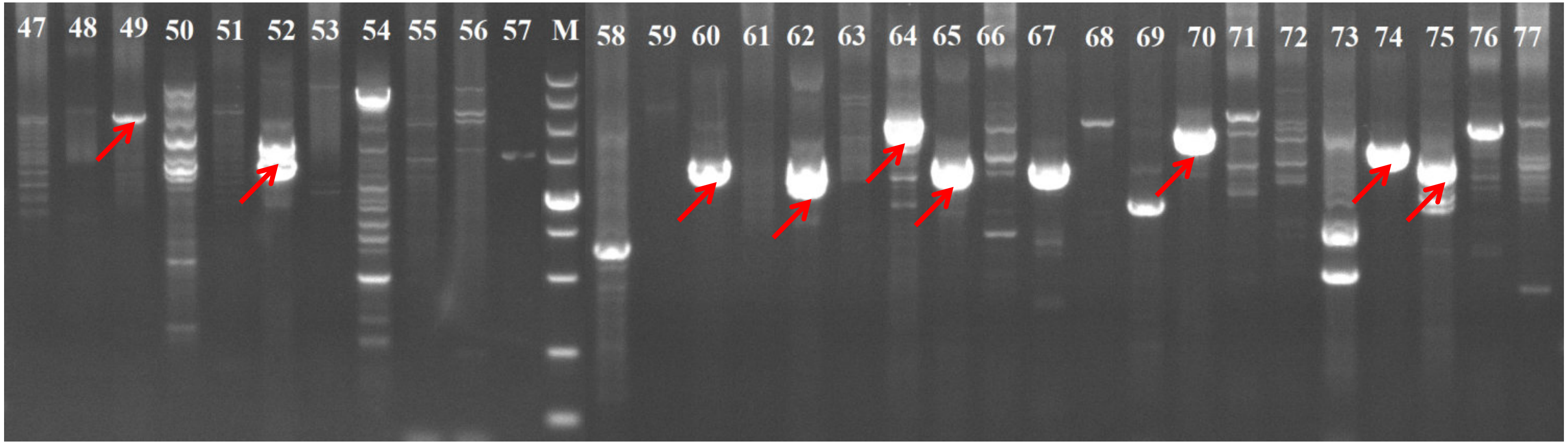
47 48 49 50 51 52 53 54 55 56 57 M 58 59 60 61 62 63 64 65 66 67 68 69 70 71 72 73 74 75 76 77

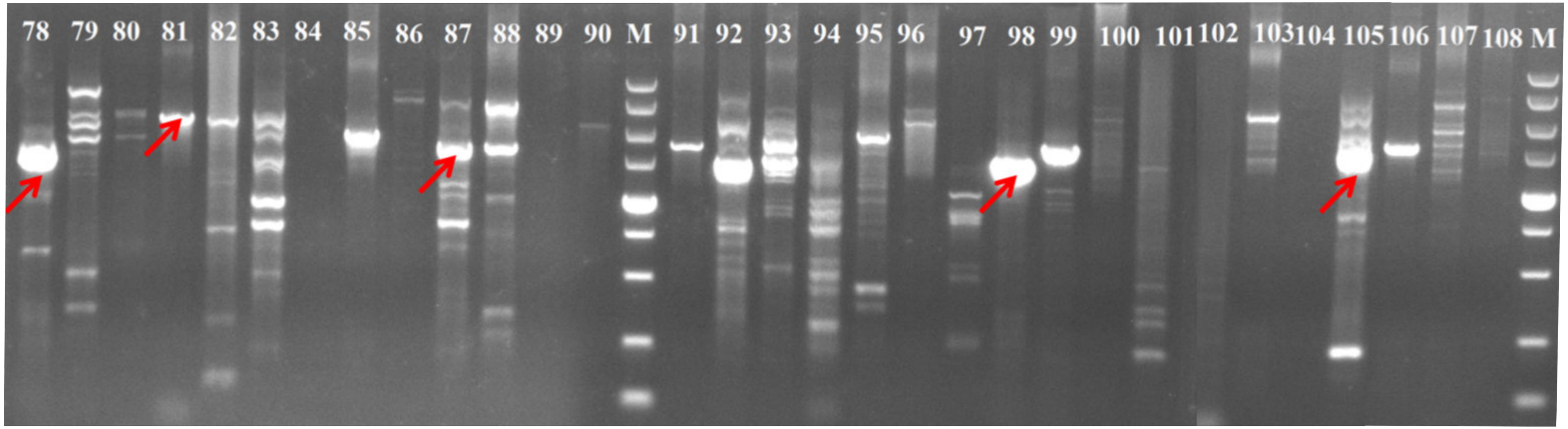

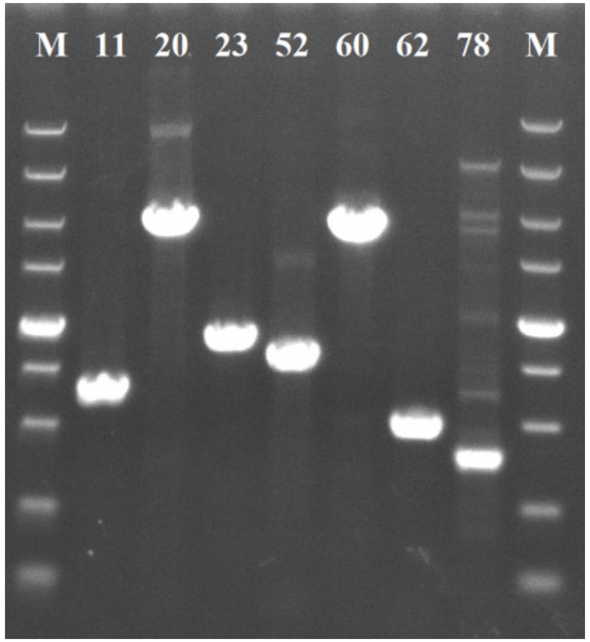

We have designed extra primers for events 11, 20, 23, 52, 60, 62 and 78.

Gepard visualization of validated CSVs

Mako chr1:81,194,398-81,195,874

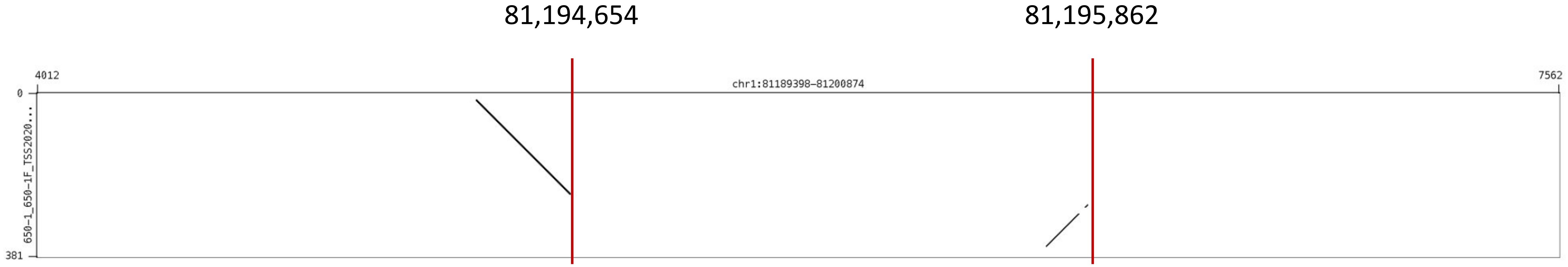

Mako chr2:119,659,504-119,661,322

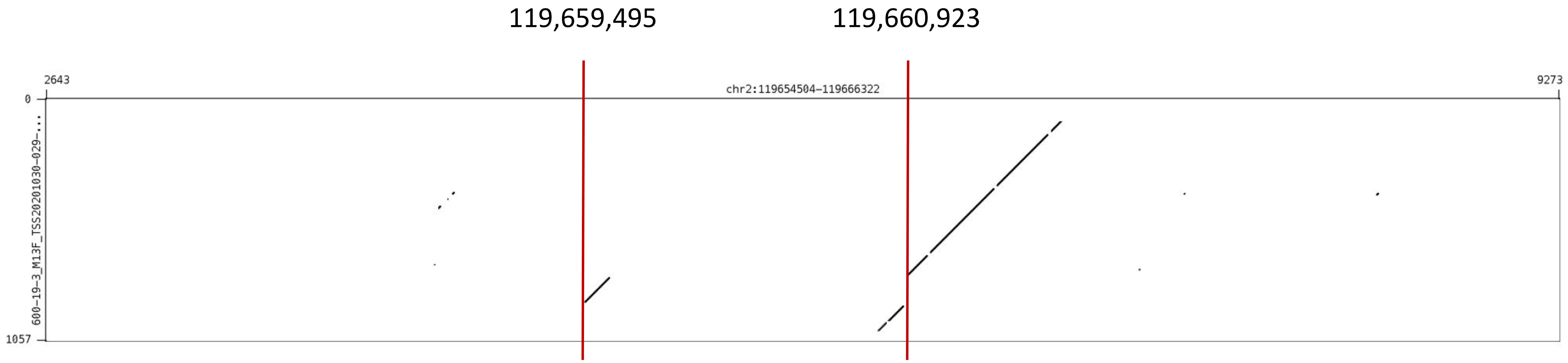

Mako chr3:146,667,093-146,677,284

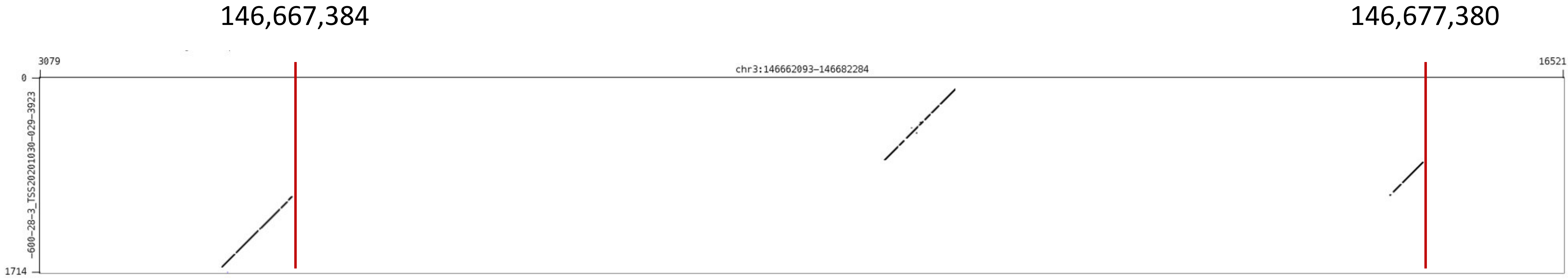

Mako chr5:141,480,327-141,483,116

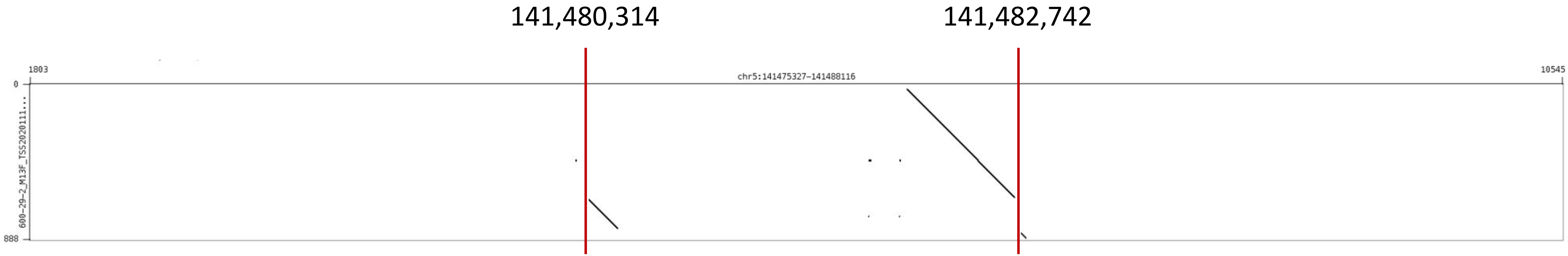

Mako chr7:1,940,931-1,941,009

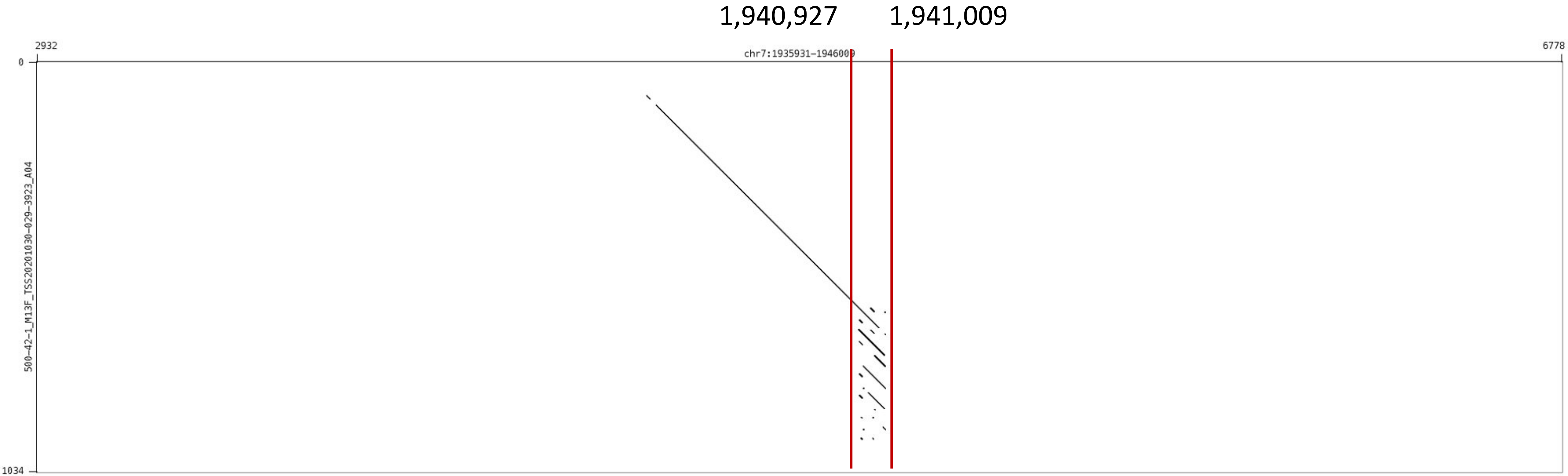

Mako chr9:29,591,409-29,593,057

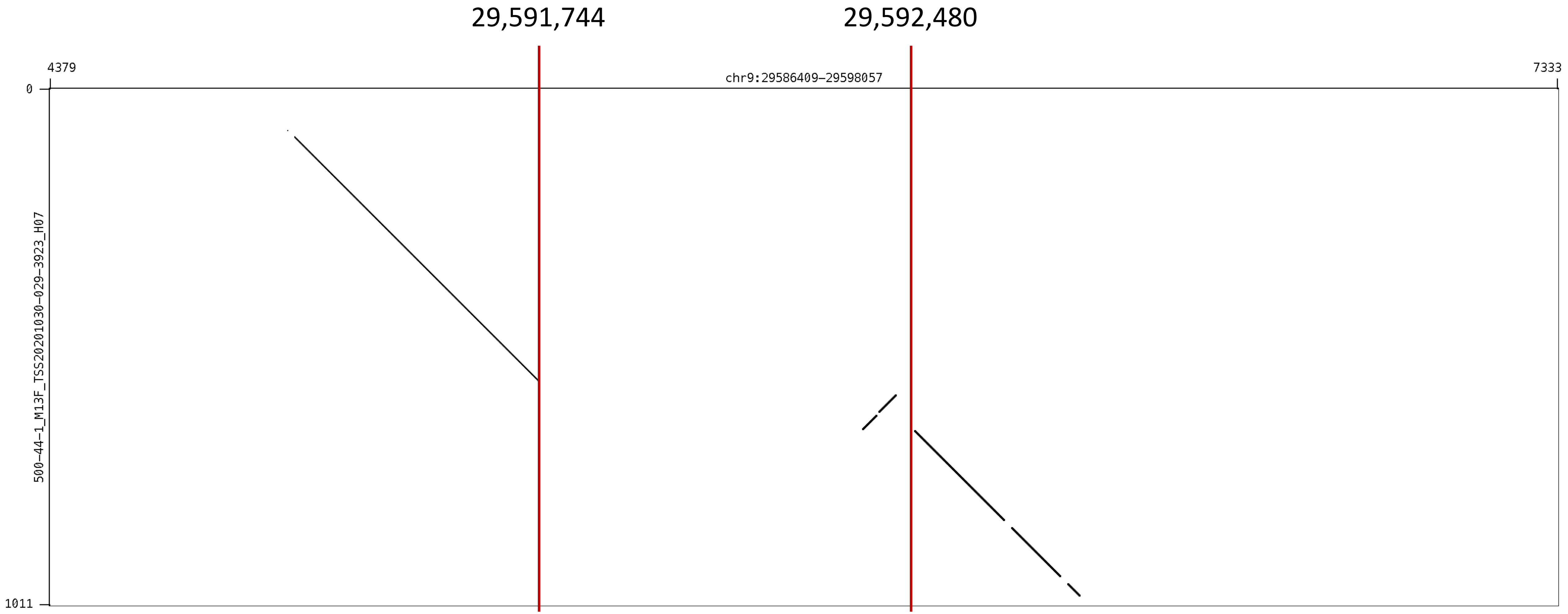

Mako chr10:14,568,488-14,568,677

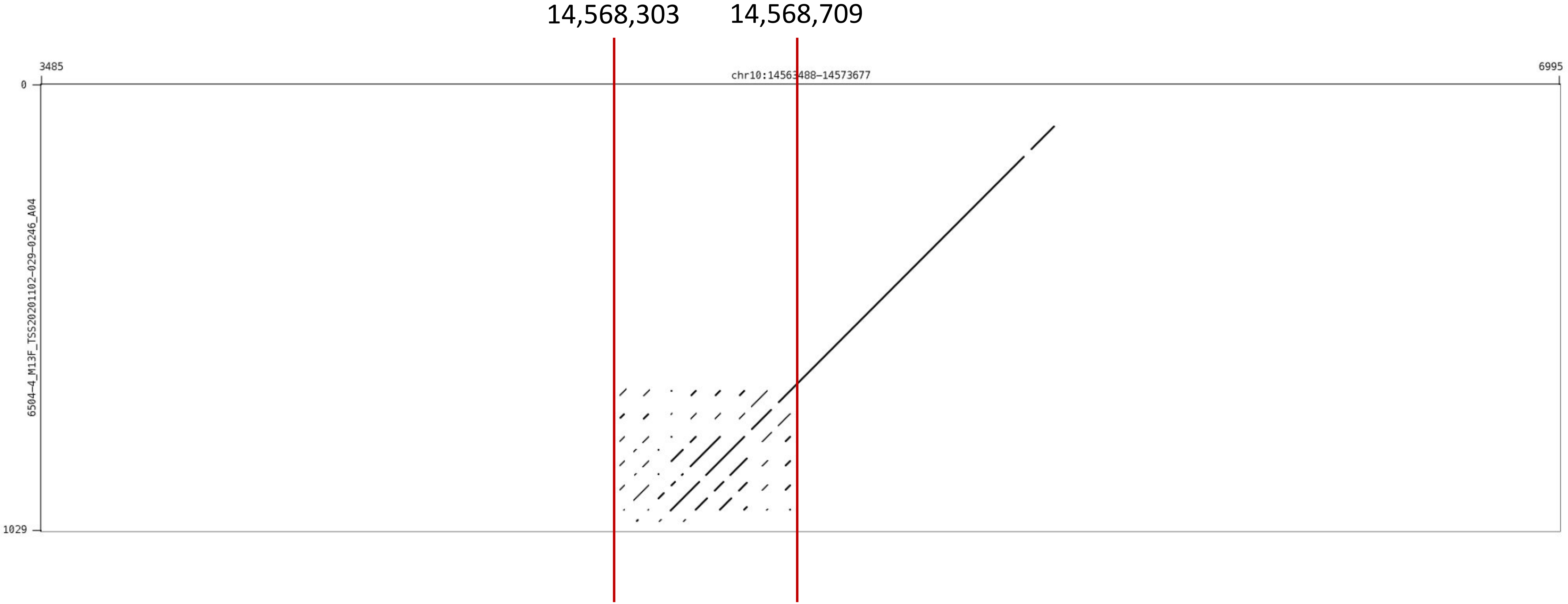

Mako chr12:71,315,482-71,316,928

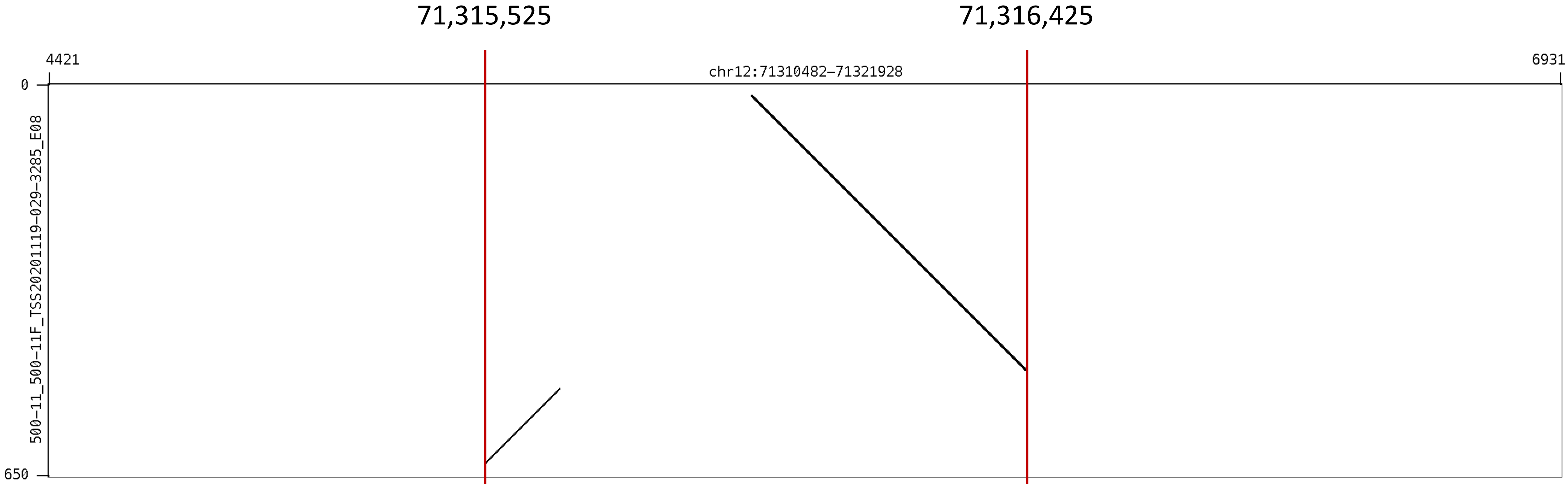

Mako chr12:77,989,900-77,994,324

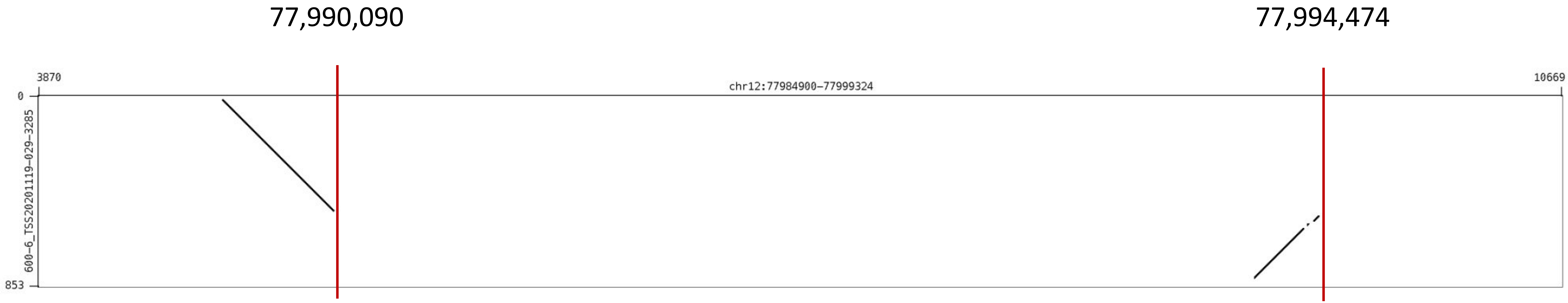

Mako chr13:74,340,759-74,342,810

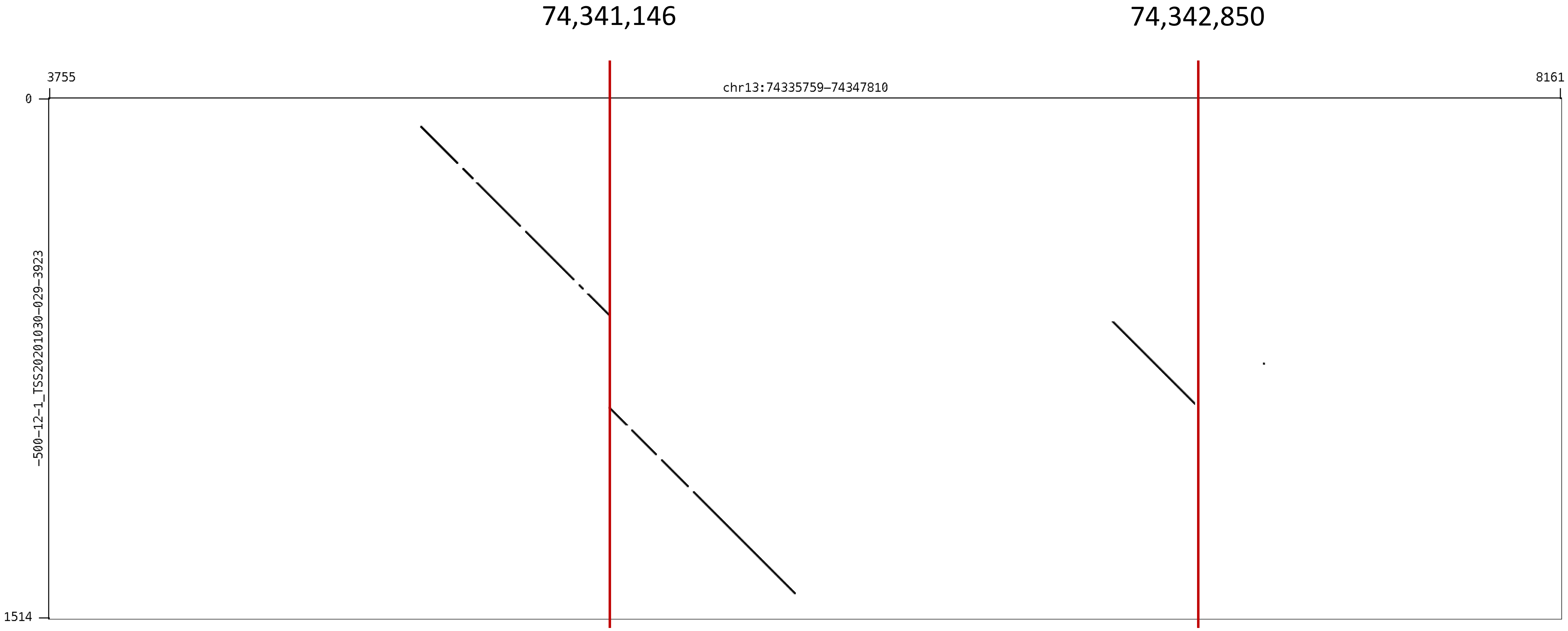

Mako chr16:78,004,459-78,007,456

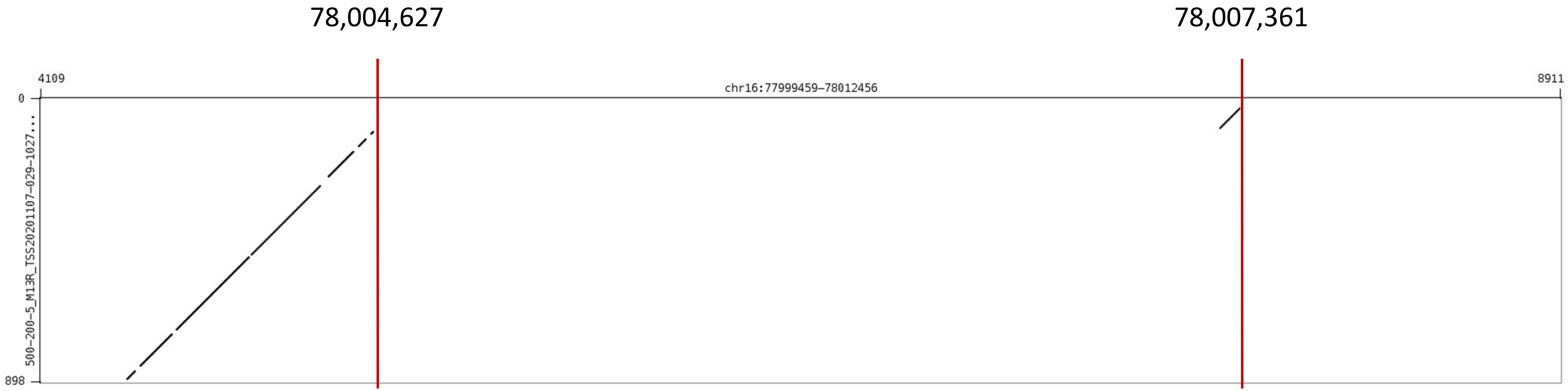

Mako chr17:34,854,438-34,855,851

34,854,671 34,855,859

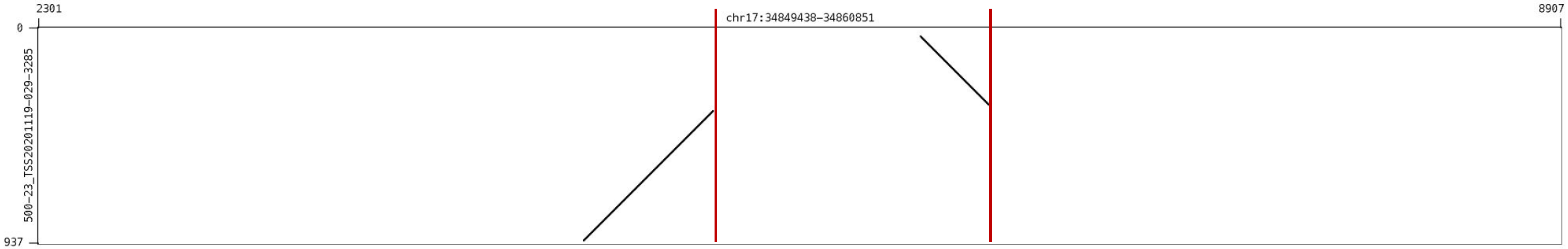

Mako chr17:48,538,270-48,540,171

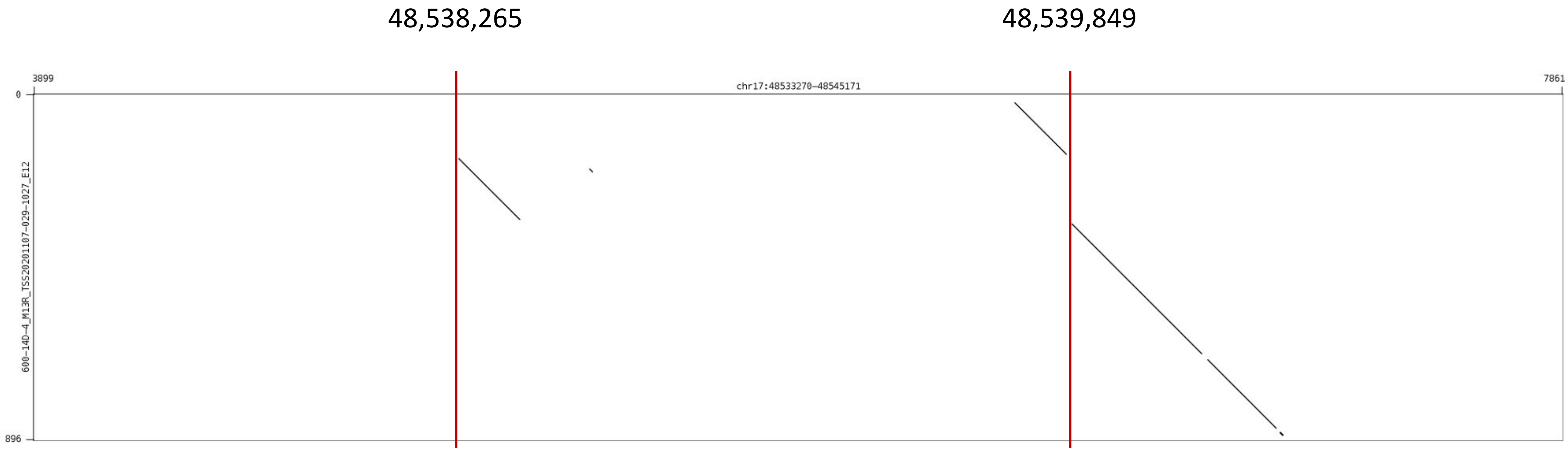

Mako chr18:72,044,575-72,045,937

72,044,564

72,045,636

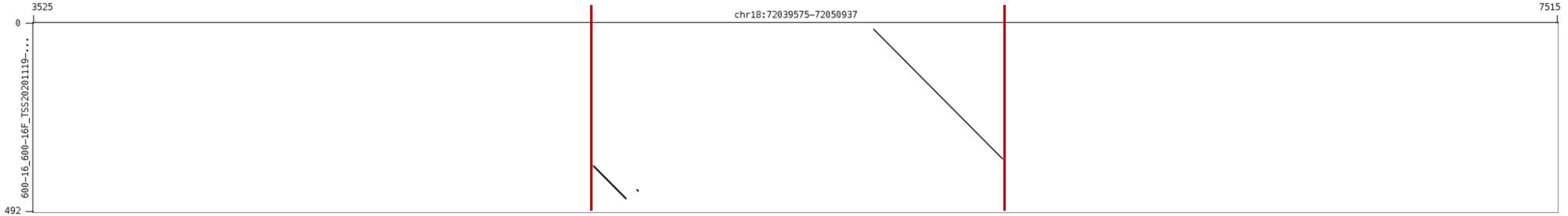

Mako chr21:26,001,844-26,002,990

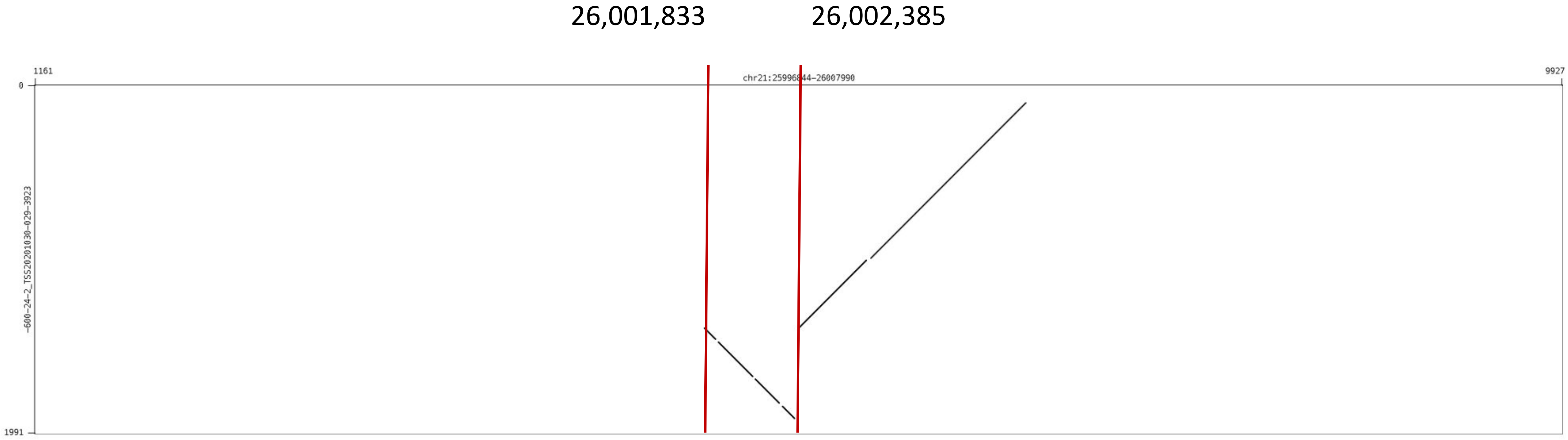

Supplement: Supplementary File S4 — PCR results and visualization of CSV breakpoint validated through Sanger sequencing [file mmc4.pdf]
