# Supplementary figures and images for: Mako: A Graph-based Pattern Growth Approach to Detect Complex Structural Variants
Source: Genomics Proteomics Bioinformatics. 2021 Jul 3;20(1):205–18. doi: 10.1016/j.gpb.2021.03.007 (PMC9510932; doi:10.1016/j.gpb.2021.03.007)

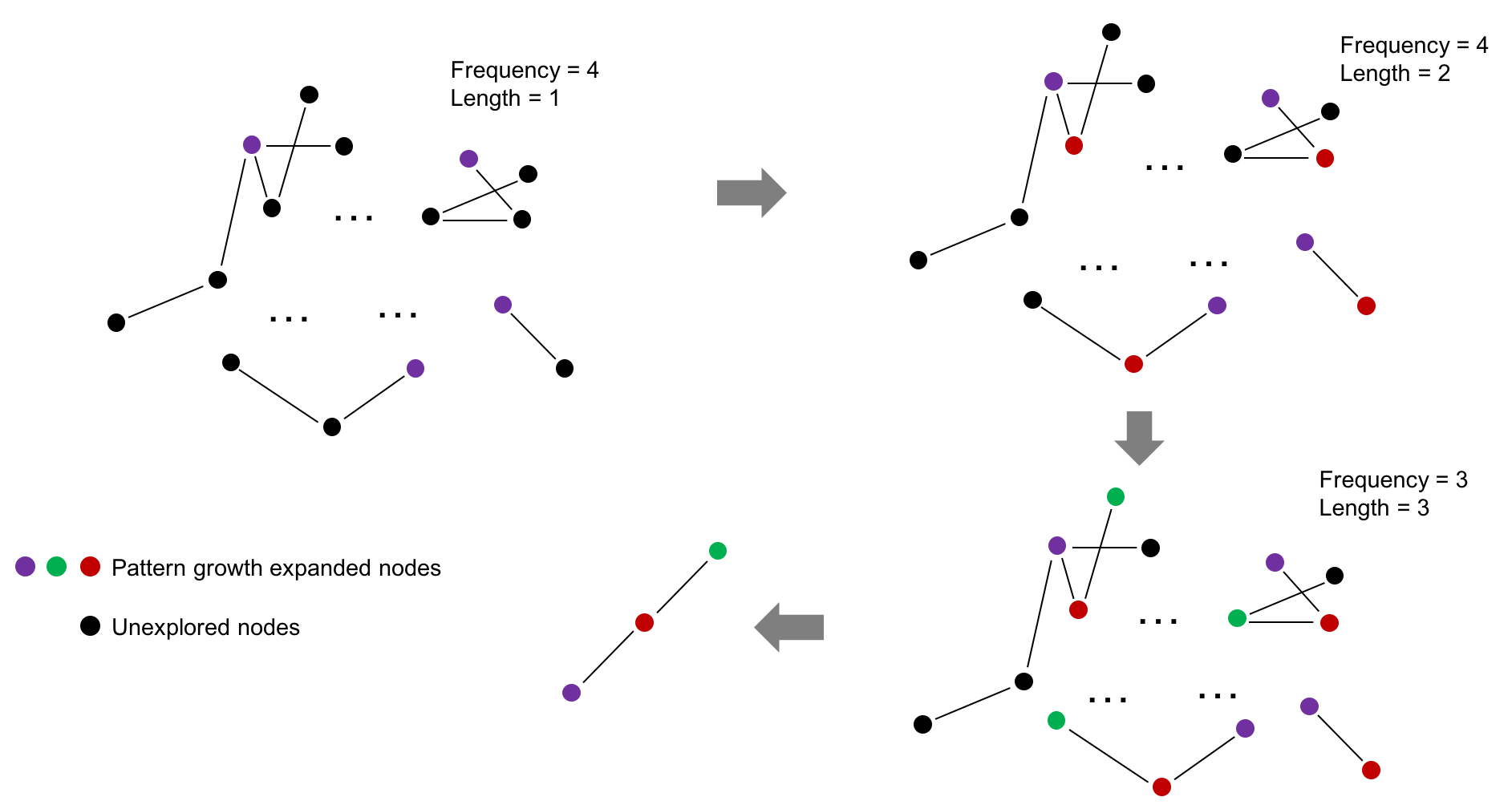

Supplement: Supplementary Figure S1 — A toy example to explain the pattern growth process [file mmc5.zip › Figure S1.png]

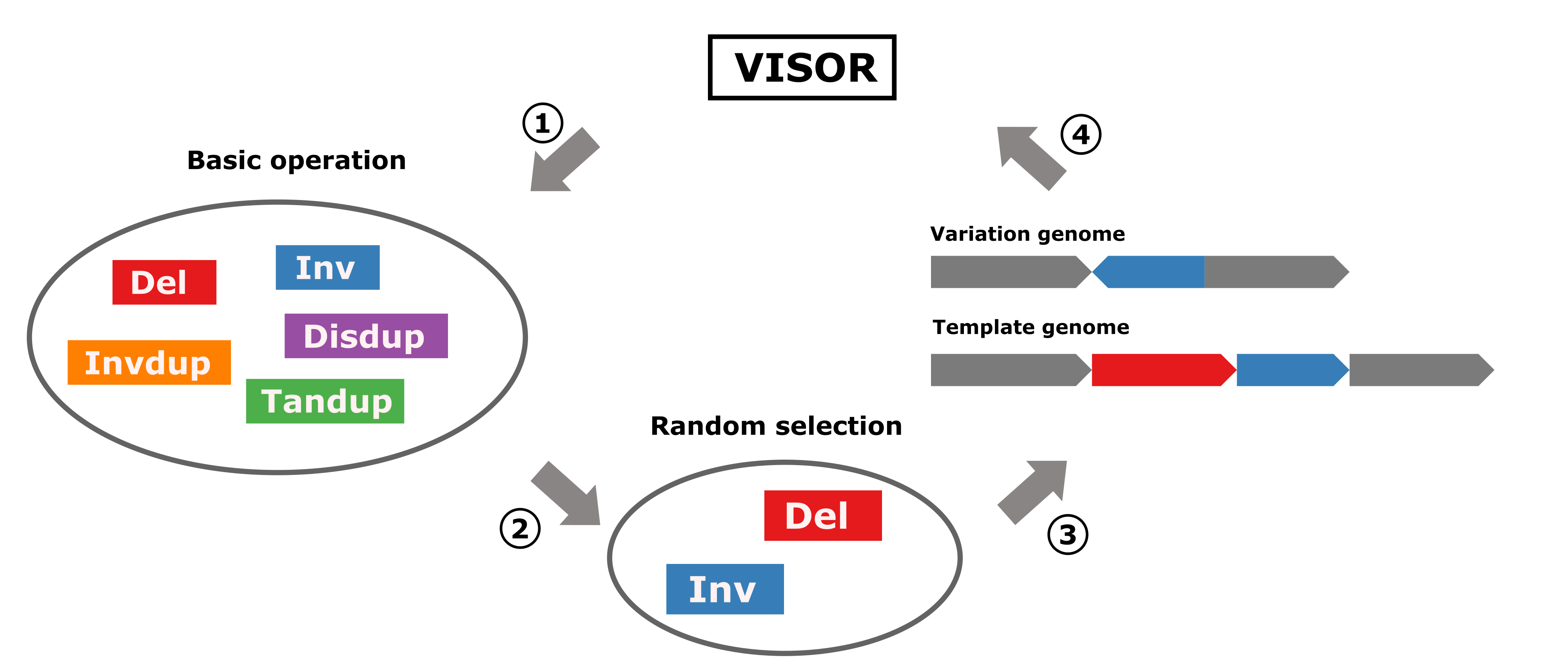

Supplement: Supplementary Figure S2 — Workflow of CSV simulation [file mmc6.zip › Figure S2.png]

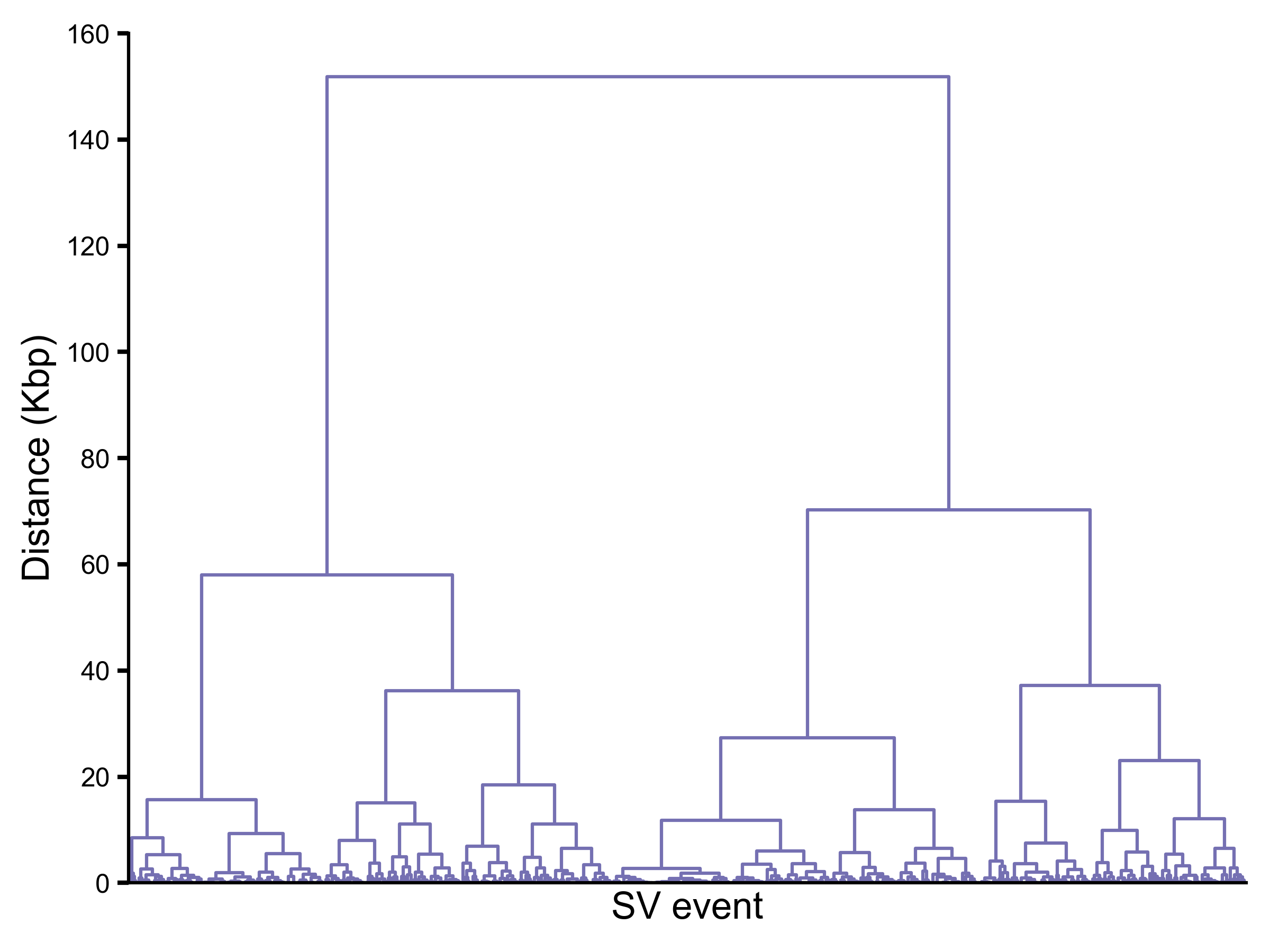

Supplement: Supplementary Figure S3 — Hierarchical clustering tree view of SVs from NA19240 chromosome 1 [file mmc7.zip › Figure S3.png]

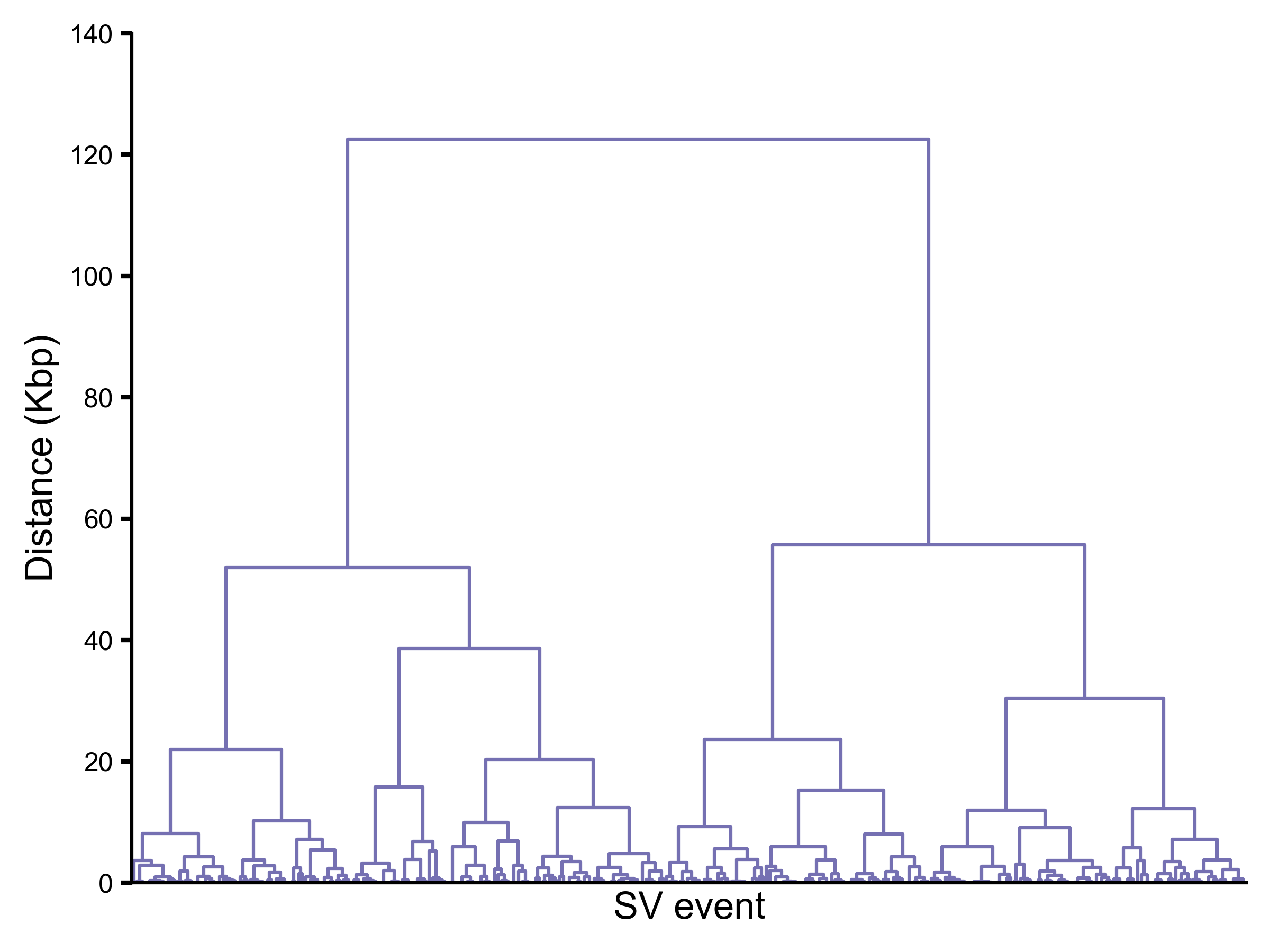

Supplement: Supplementary Figure S4 — Hierarchical clustering tree view of SVs from SKBR3 chromosome 1 [file mmc8.zip › Figure S4.png]

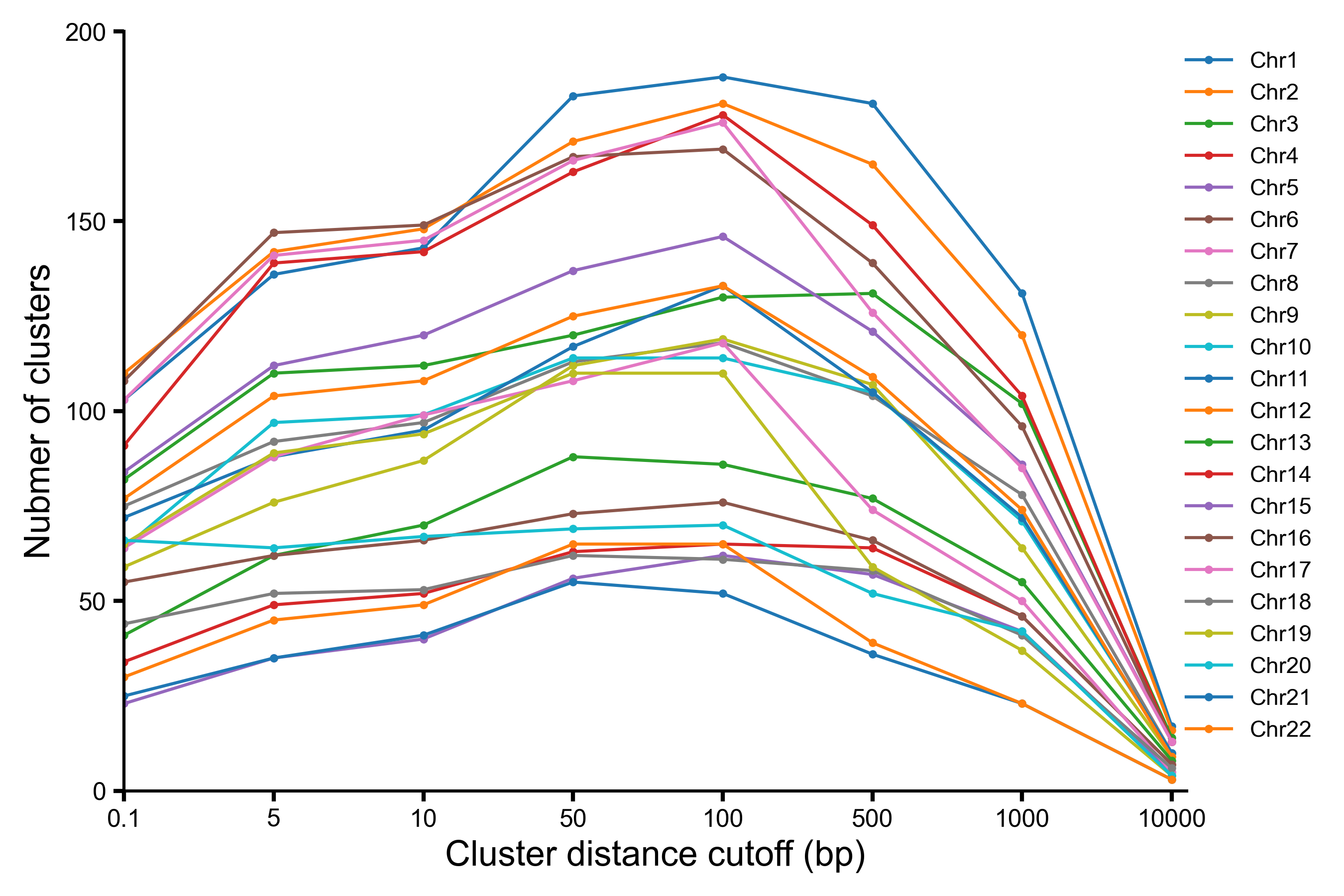

Supplement: Supplementary Figure S5 — The curve plot between cluster distance cutoff and number of clusters for SVs from NA19240 autosomes [file mmc9.zip › Figure S5.png]

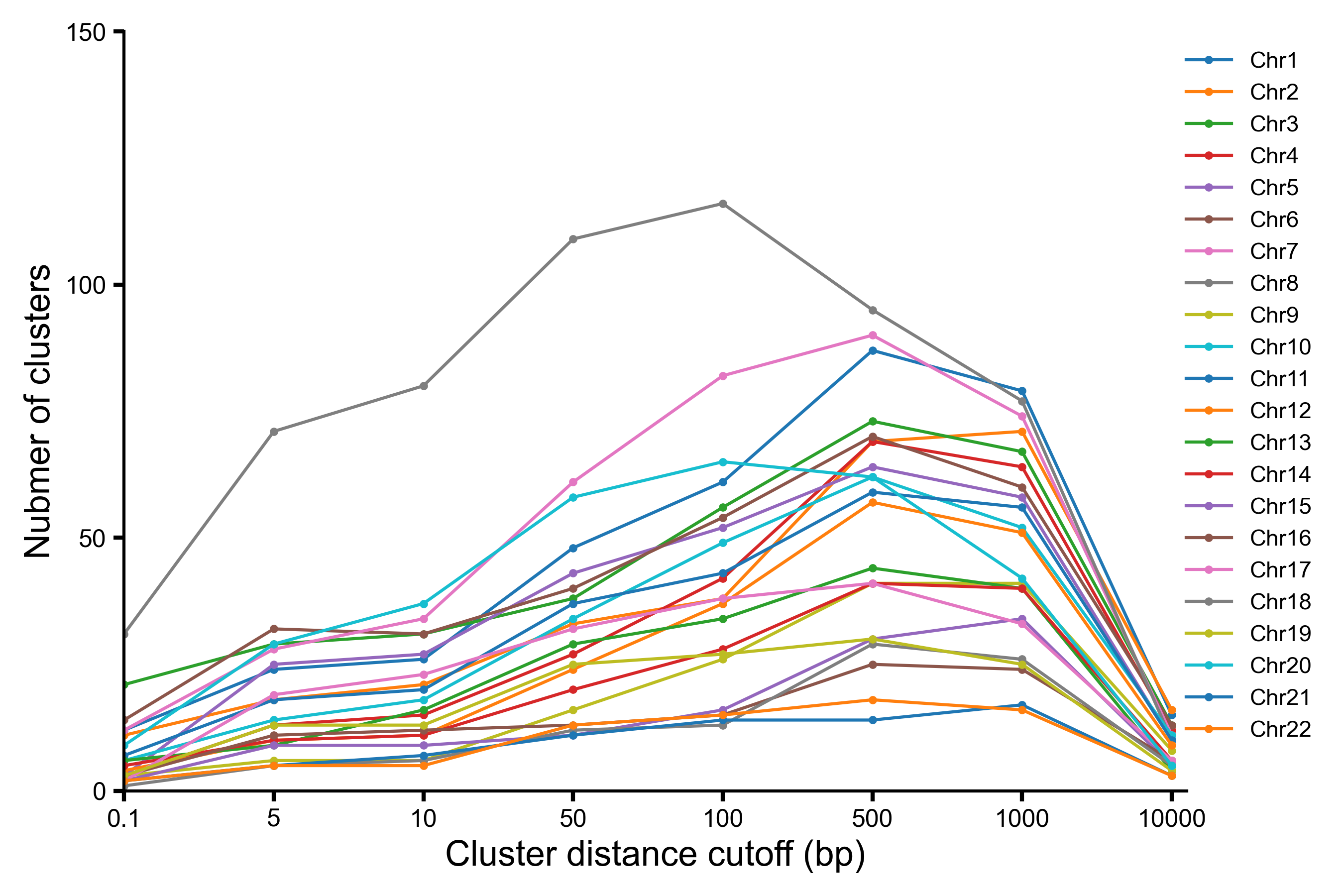

Supplement: Supplementary Figure S6 — The curve plot between cluster distance cutoff and number of clusters for SVs from SKBR3 autosomes [file mmc10.zip › Figure S6.png]

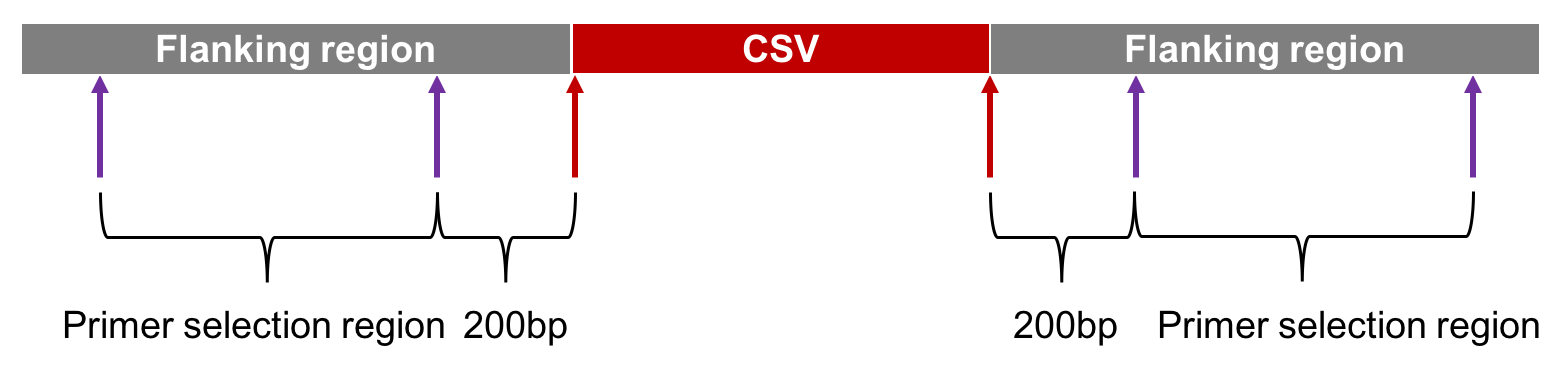

Supplement: Supplementary Figure S7 — Diagram of selecting primers for each CSV [file mmc11.zip › Figure S7.png]

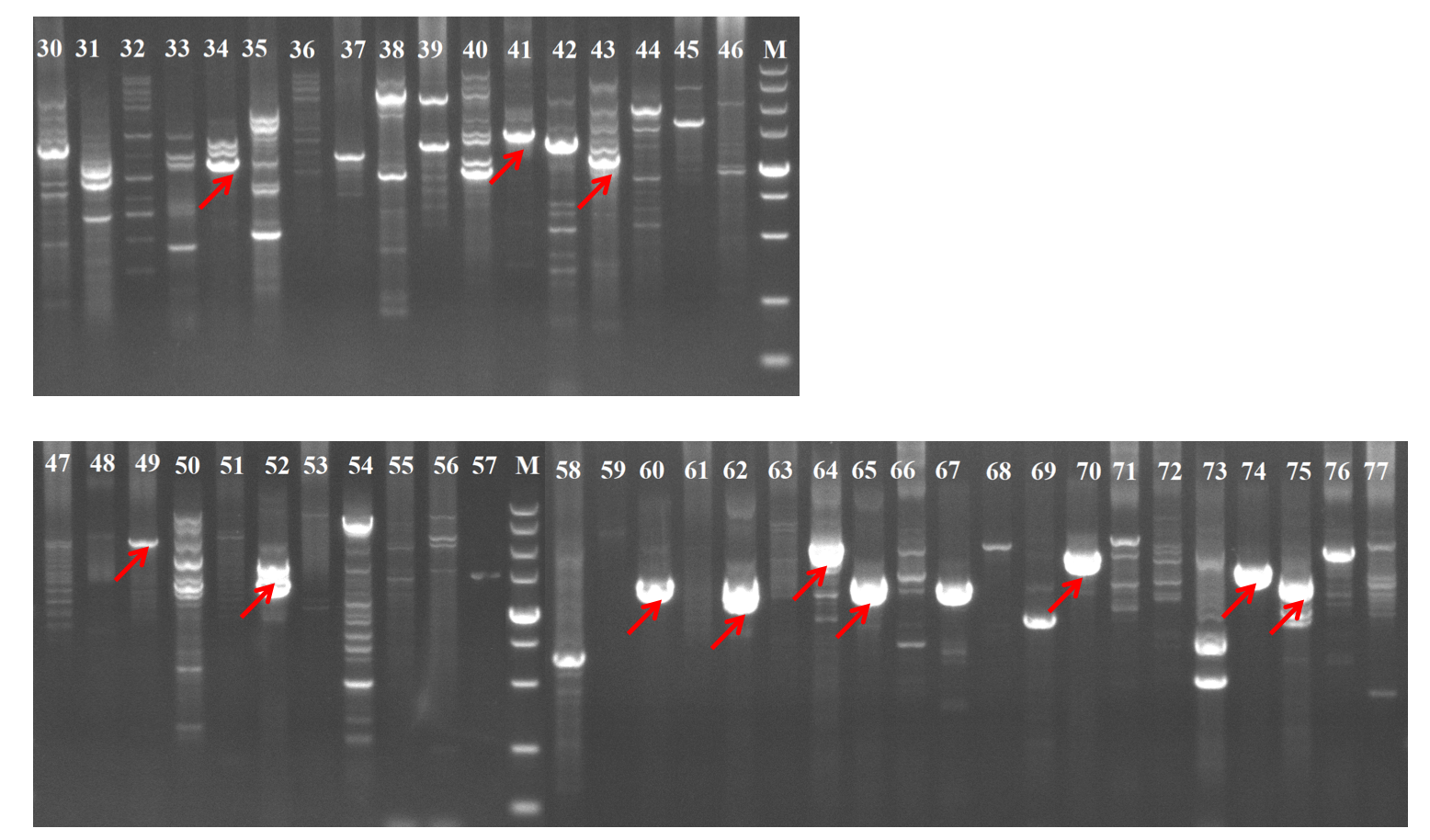

Supplement: Supplementary Figure S8 — Examples of PCR electrophoretic bands visualized under the UV light [file mmc12.zip › Figure S8.png]

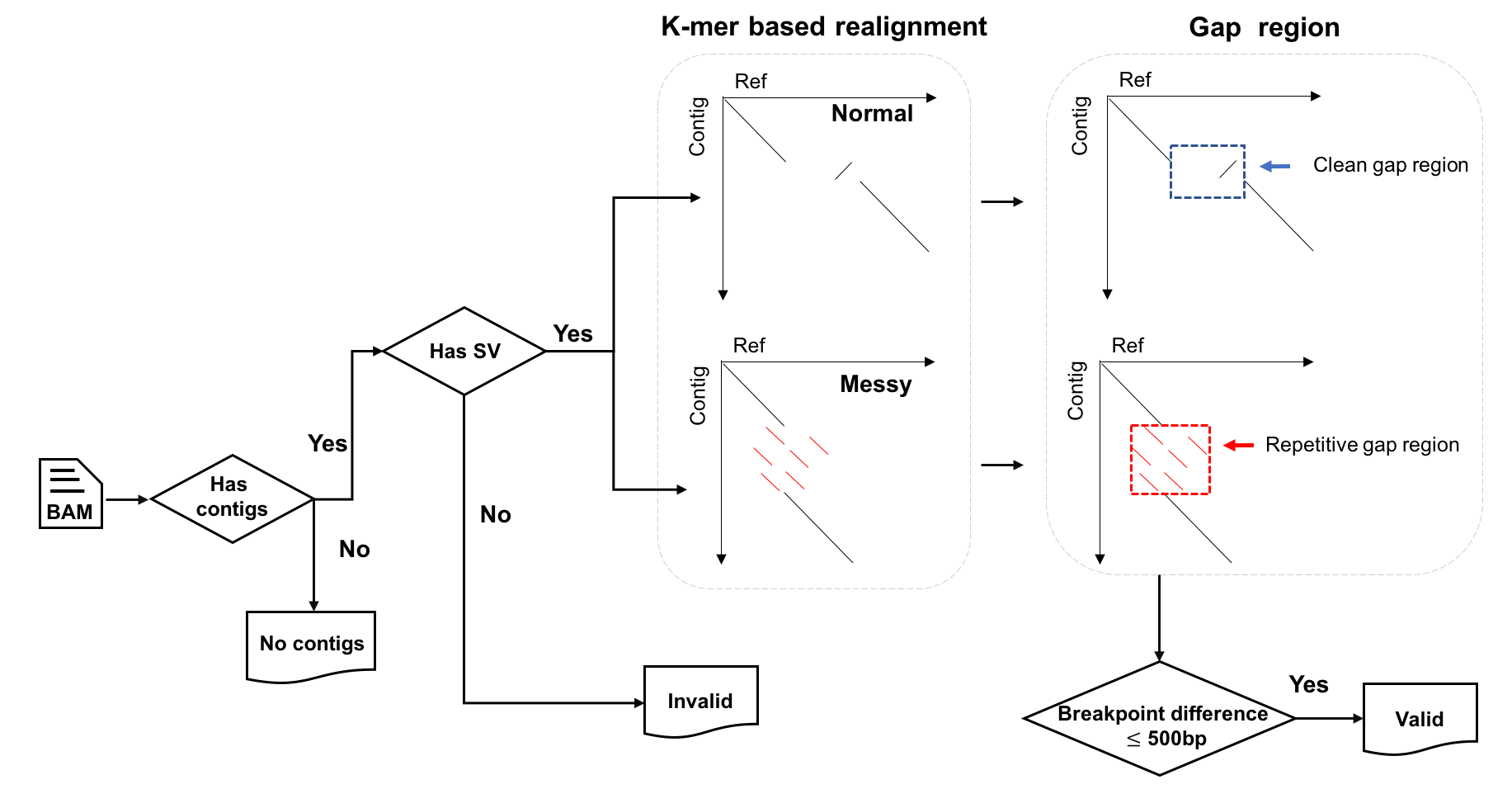

Supplement: Supplementary Figure S9 — Workflow of HiFi assembly K-mer validation [file mmc13.zip › Figure S9.png]

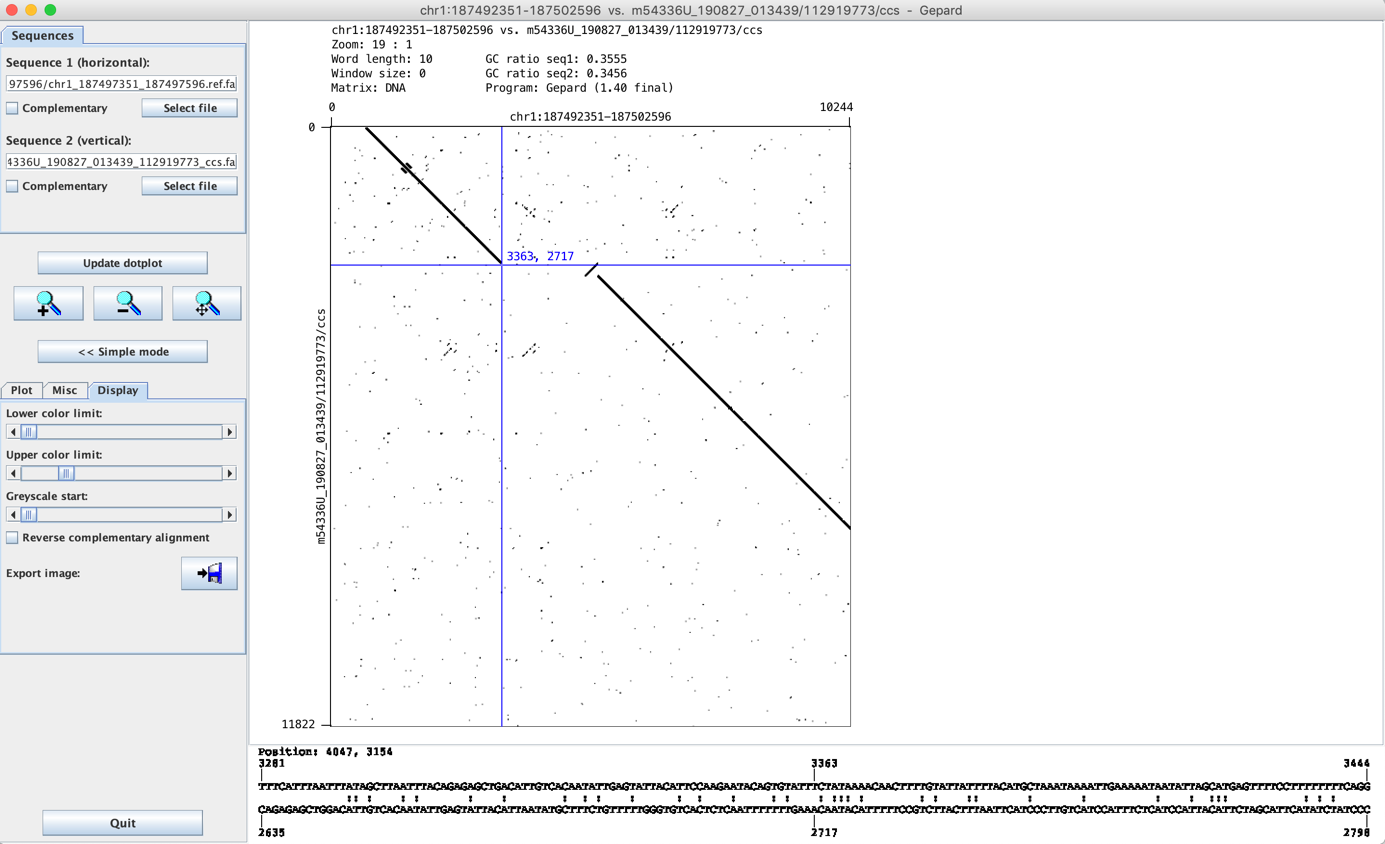

Supplement: Supplementary Figure S10 — A screenshot using Gepard to investigate a deletion associated with inversion event [file mmc14.zip › Figure S10.png]

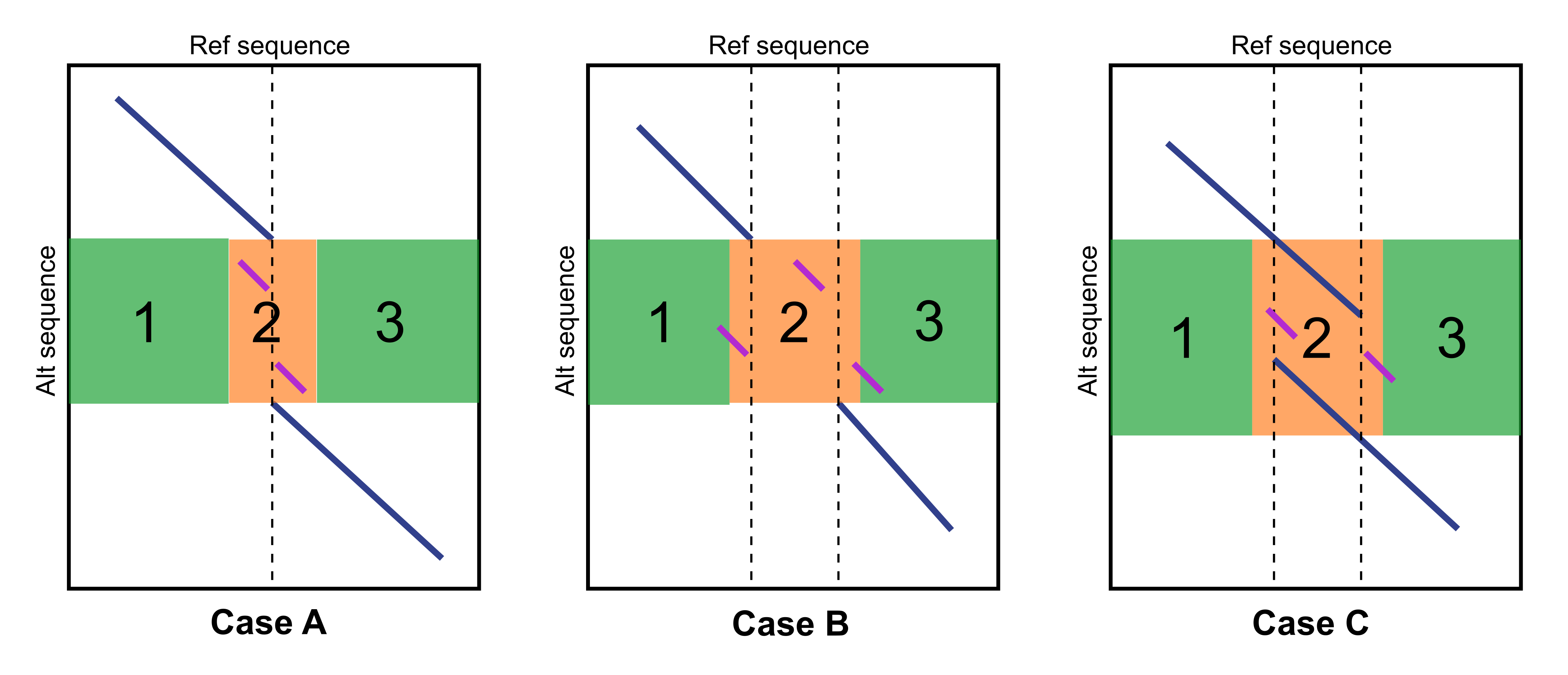

Supplement: Supplementary Figure S11 — Dotplot patterns used to identify CSVs at highly repetitive regions [file mmc15.zip › Figure S11.png]

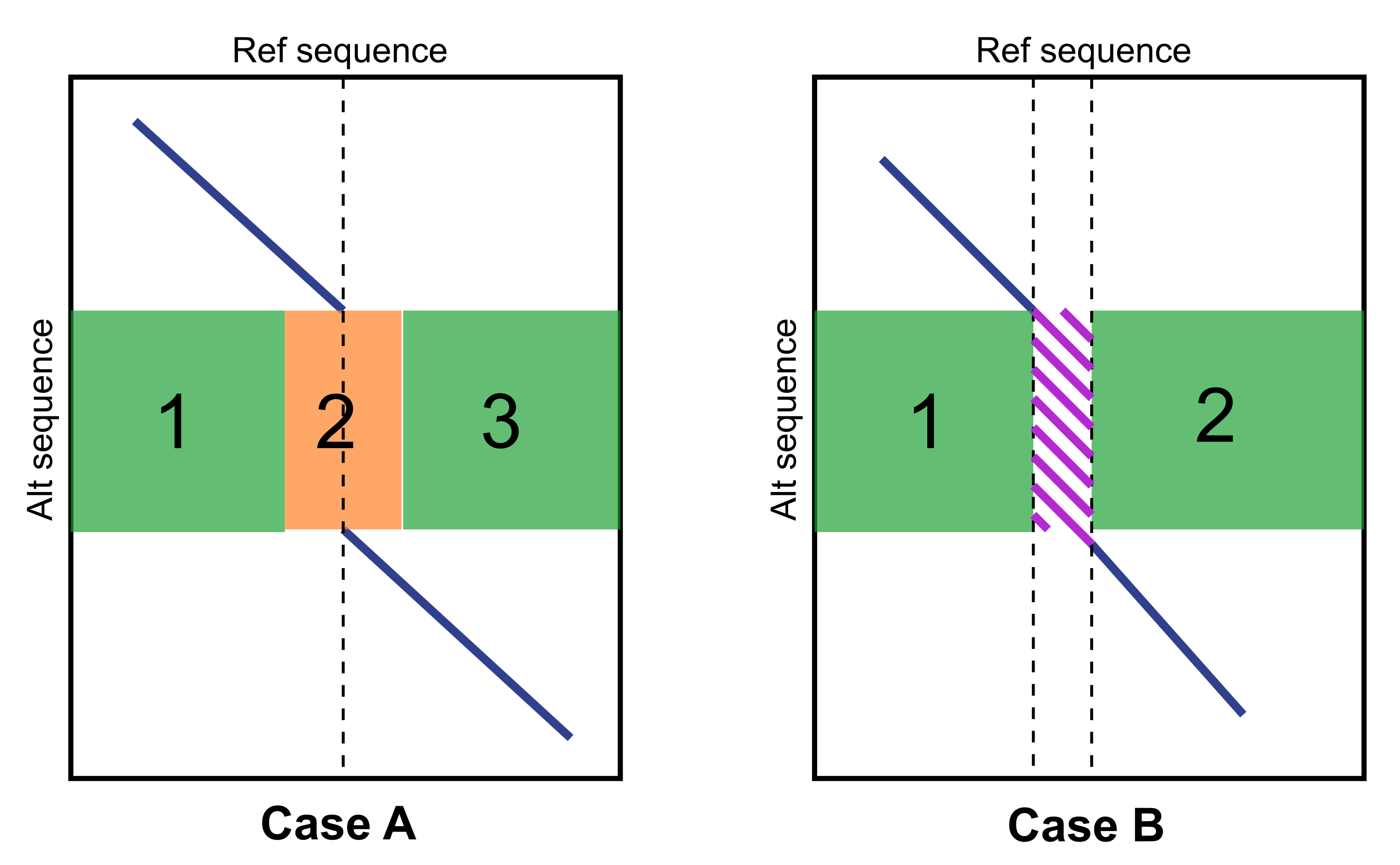

Supplement: Supplementary Figure S12 — Dotplot patterns used to identify SVs at highly repetitive regions [file mmc16.zip › Figure S12.png]

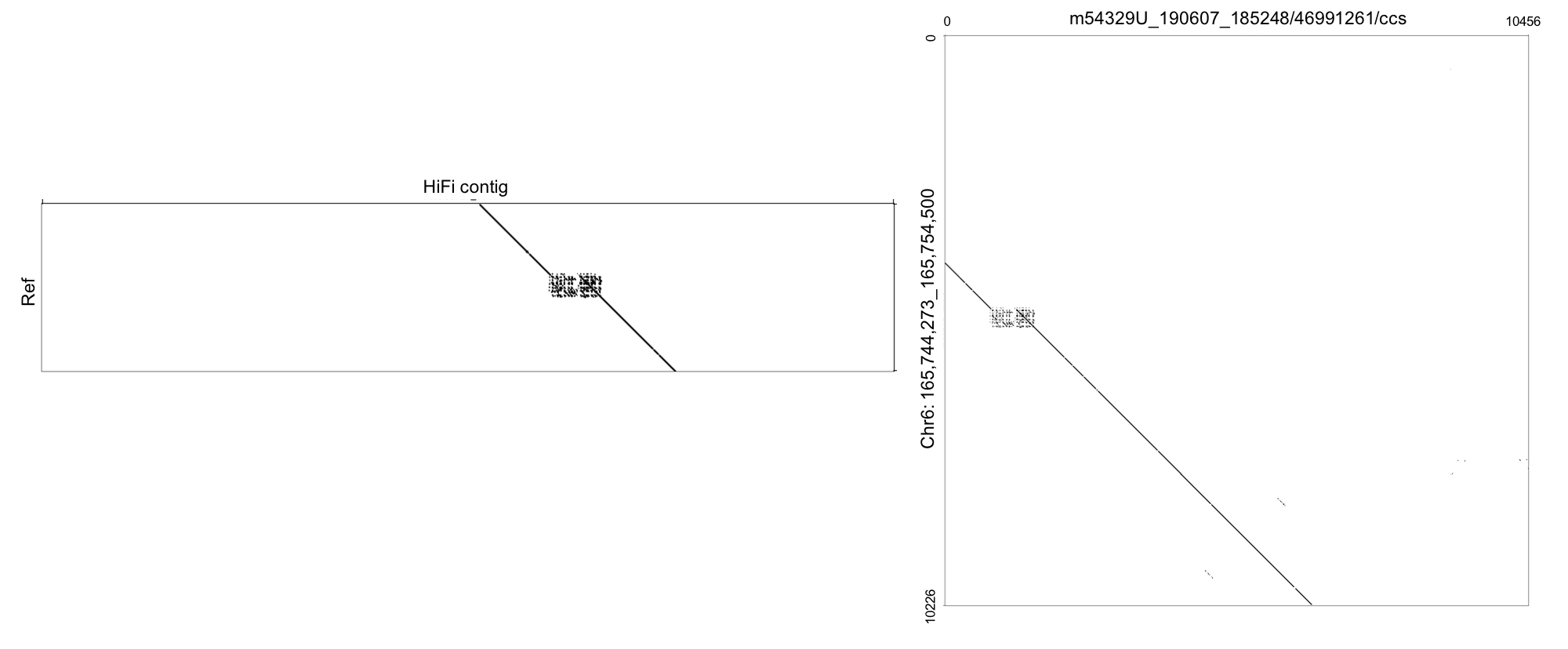

Supplement: Supplementary Figure S13 — Example call at high repetitive regions that labeled as NA by VaPoR at chr6:165,749,273-165,749,500 [file mmc17.zip › Figure S13.png]

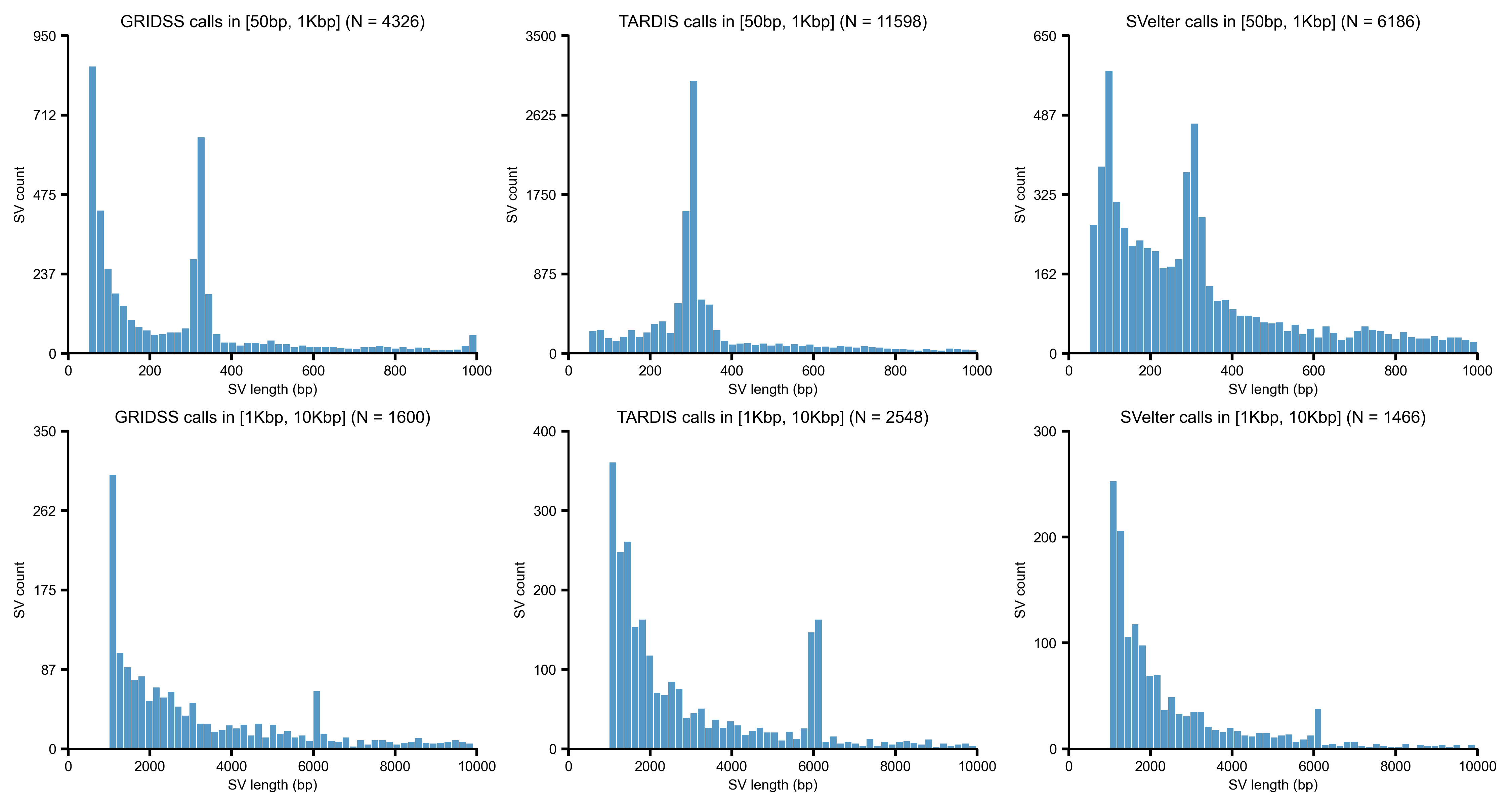

Supplement: Supplementary Figure S14 — Size distribution of SV in the range [50bp, 10Kbp] from NA19240 [file mmc18.zip › Figure S14.png]

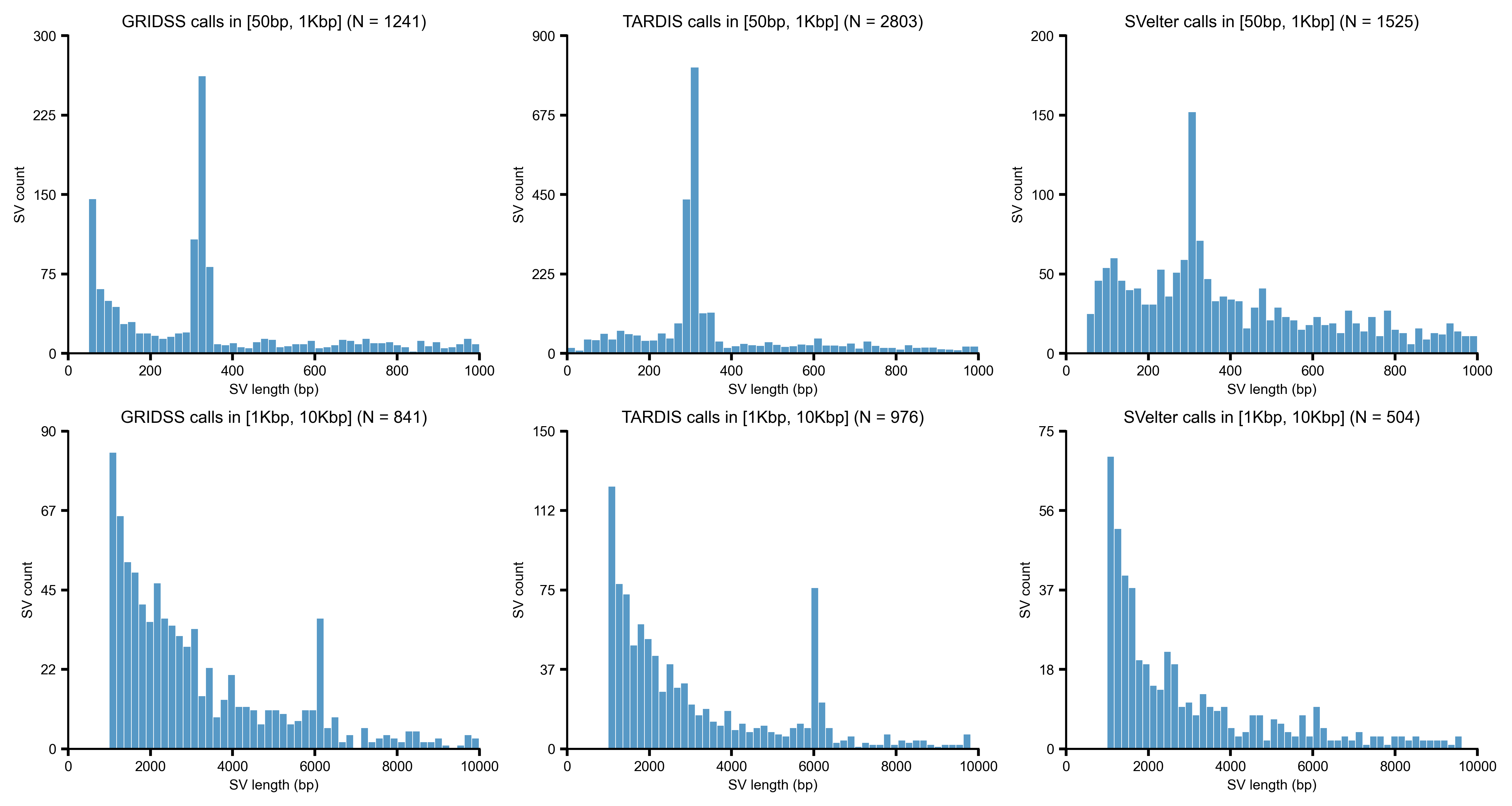

Supplement: Supplementary Figure S15 — Size distribution of SV in the range [50bp, 10Kbp] from SKBR3 breast cancer cell line [file mmc19.zip › Figure S15.png]

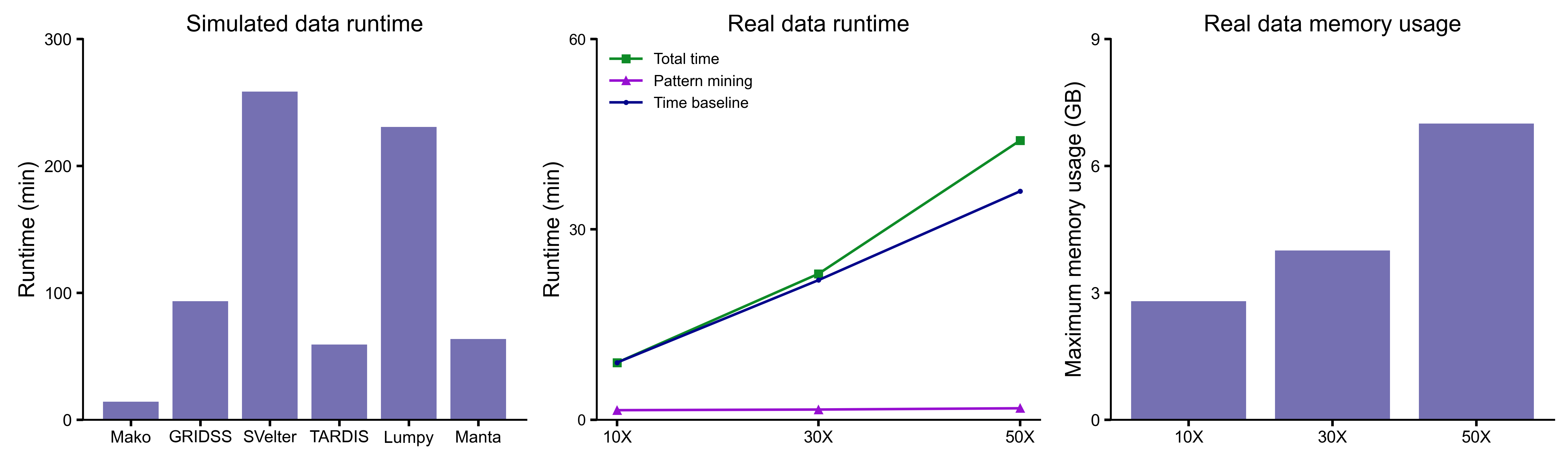

Supplement: Supplementary Figure S16 — Running time comparison between different methods A. Runtime comparison on simulated data at 30× coverage. B. Runtime of Mako on real data at different coverage. The time baseline is decided by copying the original BAM to another location. C. Memory usage of Mako on real data at different coverage. [file mmc20.zip › Figure S16.png]

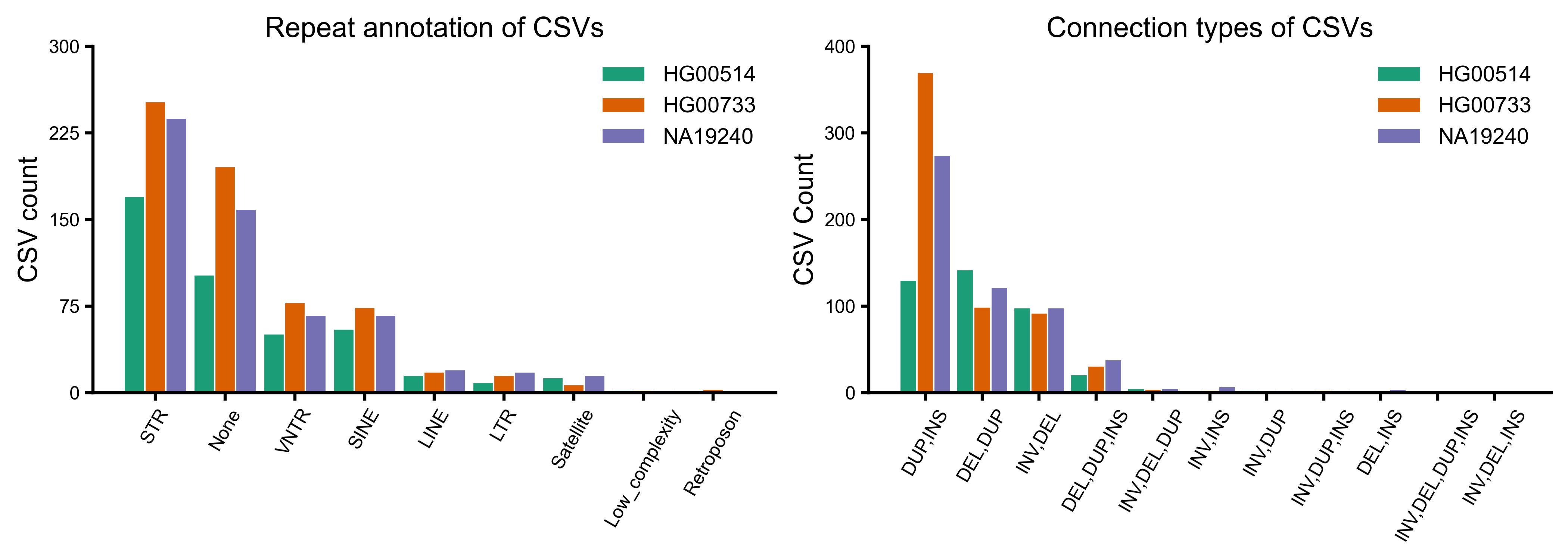

Supplement: Supplementary Figure S17 — Repeat annotation and connection types of Mako-detected CSVs from three samples A. Repeat annotation of CSVs detected from three genomes. B. Mako predicted CSV types of three genomes. [file mmc21.zip › Figure S17.png]

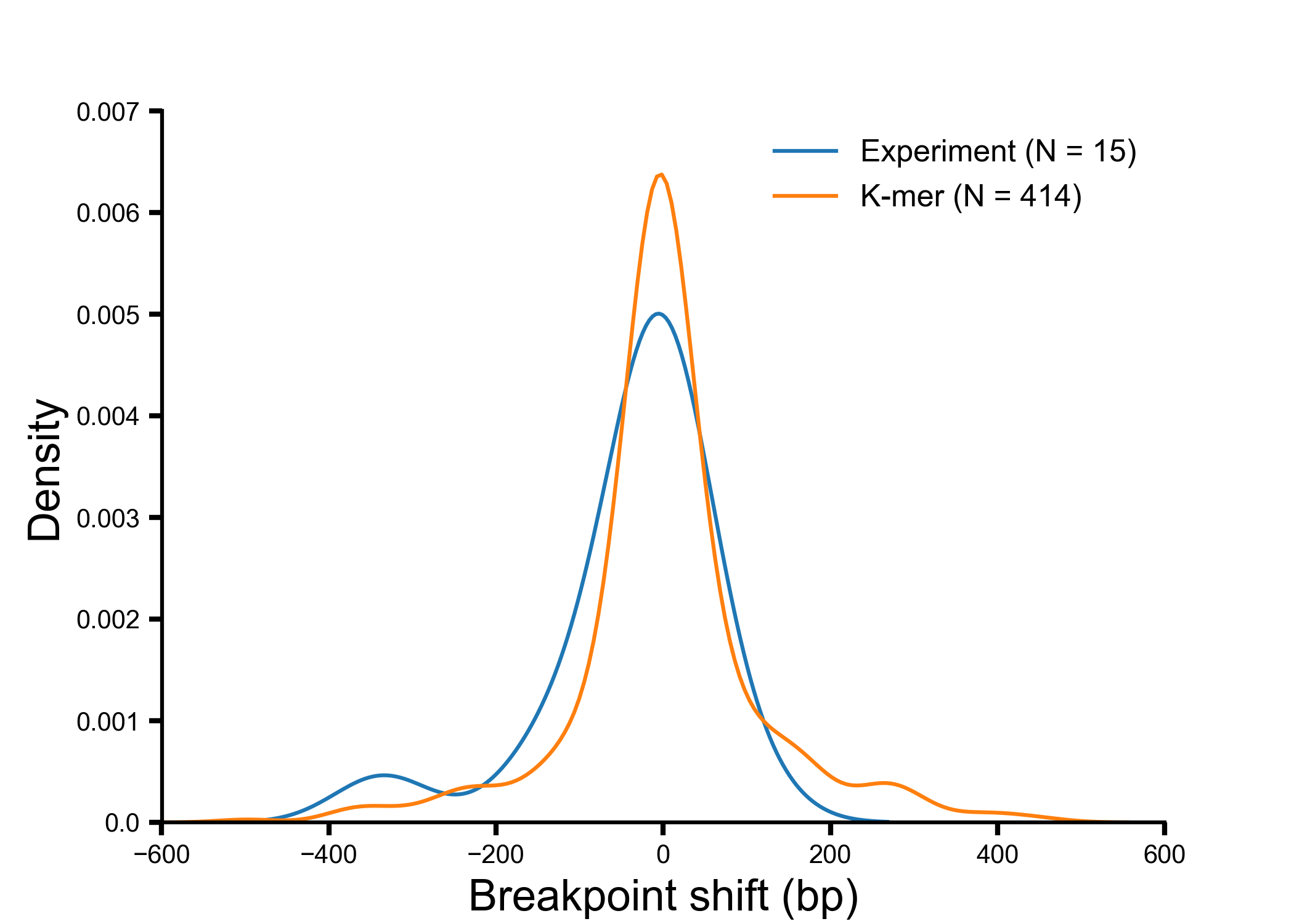

Supplement: Supplementary Figure S18 — Mako-detected CSV breakpoint resolution compared to HiFi contig (K-mer) and experiment [file mmc22.zip › Figure S18.png]

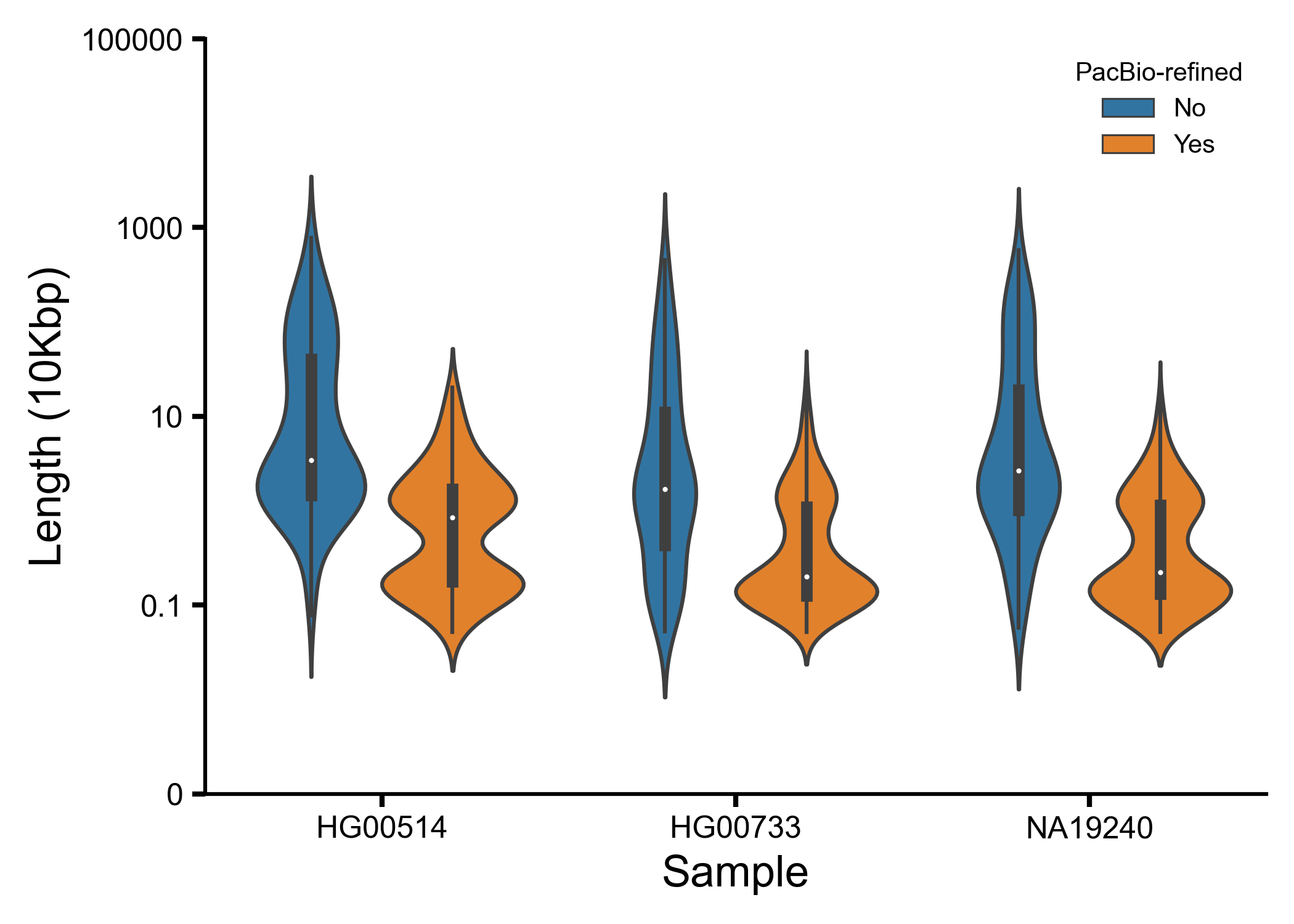

Supplement: Supplementary Figure S19 — Mako-detected CSV and PacBio HiFi read refined CSV sizedistribution [file mmc23.zip › Figure S19.png]

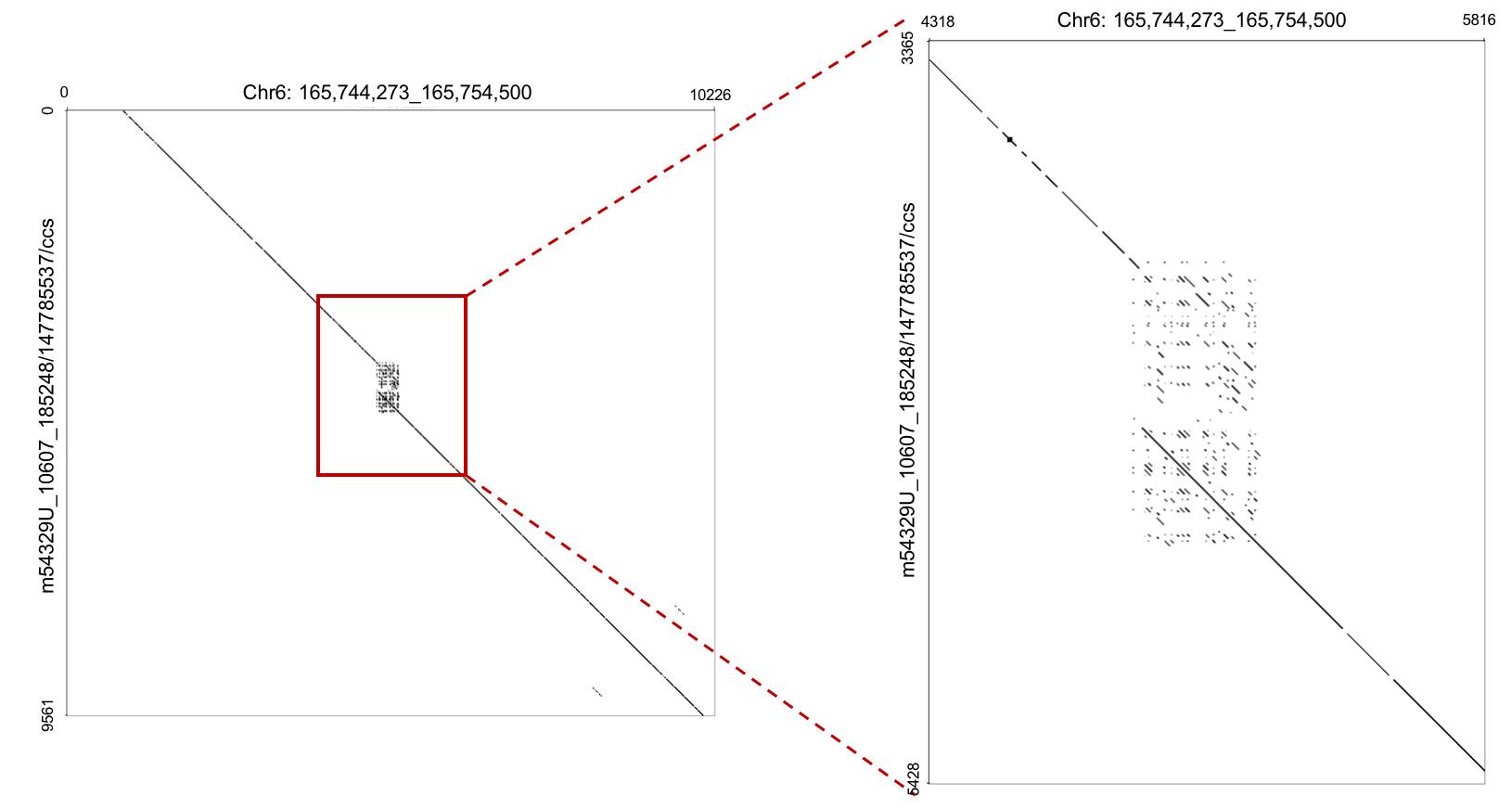

Supplement: Supplementary Figure S20 — Example of an insertion associated with duplication event (InsDup) at chr6:165,749,273-165,749,500 [file mmc24.zip › Figure S20.png]

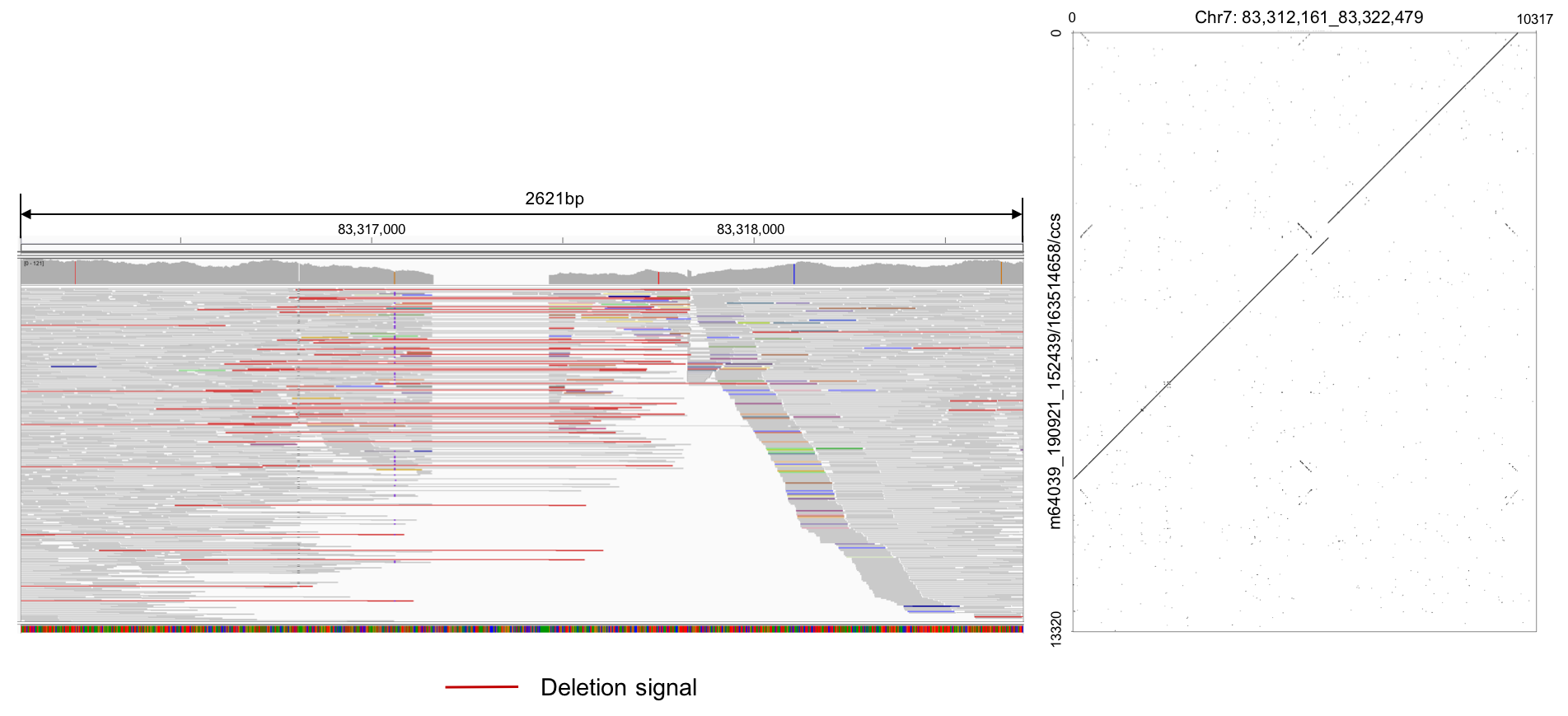

Supplement: Supplementary Figure S21 — The IGV view and sequence dot-plot of the adjacent segment swap from NA19240 at Chr7:83,316,809-83,317,466 [file mmc25.zip › Figure S21.png]

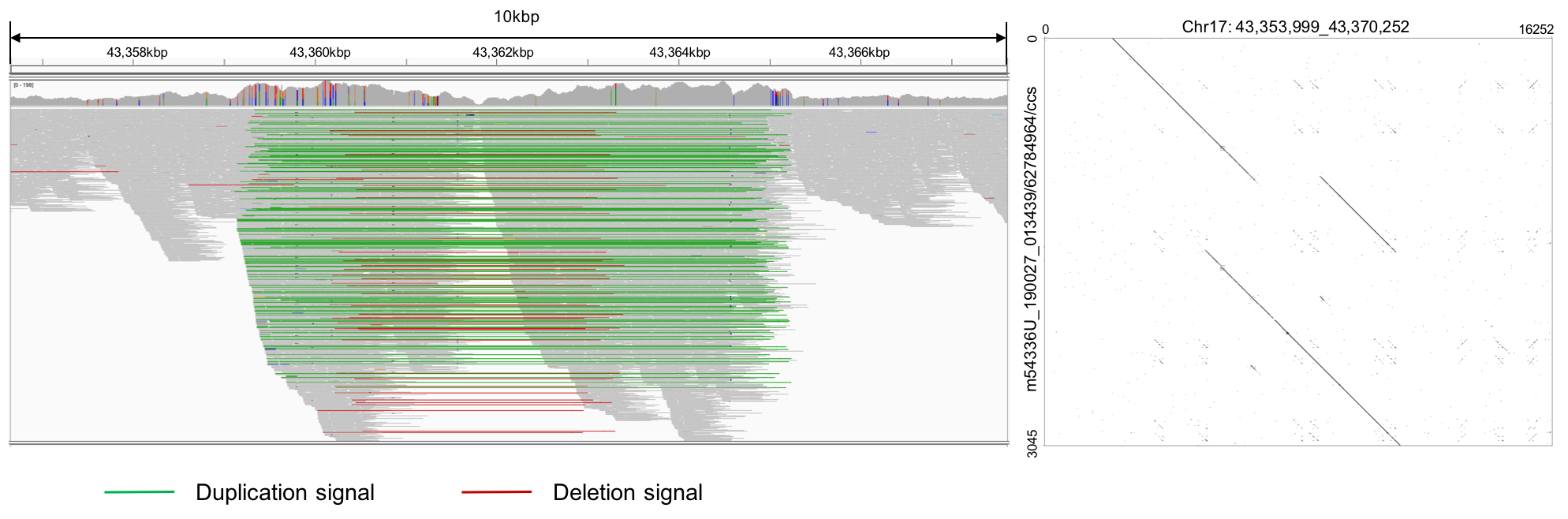

Supplement: Supplementary Figure S22 — The IGV view and sequence dot-plot of the tandem dispersed duplication from NA19240 at Chr17:43,359,104-43,365,253 [file mmc26.zip › Figure S22.png]

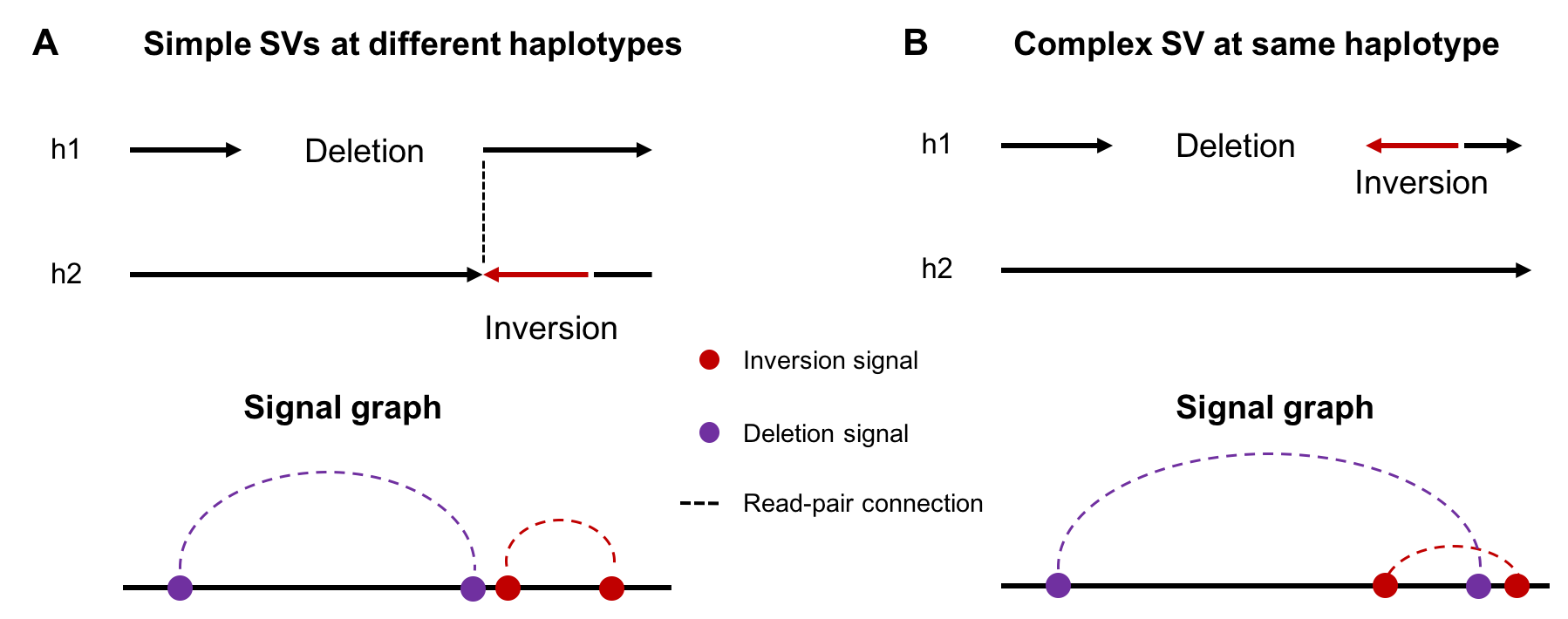

Supplement: Supplementary Figure S23 — Examples to show the difference of CSV breakpoints from single haplotype or two haplotypes A. Diagram of two simple SVs at different haplotypes. B. Diagram of complex SV at the same haplotype [file mmc27.zip › Figure S23.png]
